
JOURNAL INFORMATION
==============================
NLM Title Abbreviation: J Med Phys
NLM Journal ID: 9441104
Journal ID: JMP

Journal of Medical Physics / Association of Medical Physicists of India

ISSN: 0971-6203
EISSN: 1998-3913
Publisher: Wolters Kluwer -- Medknow Publications

ARTICLE INFORMATION
==============================
PMCID: PMC3146081
Article ID: JMP-31-154
Article version: 1
Subjects: Synopses of Papers Presented at Ampicon 2006

Synopses of Poster Papers

Print publication date: 2006 Jul-Sep
Volume: 31
Issue: 3
First page: 154
Last page: 224
Copyright: © Journal of Medical Physics
Copyright year: 2006
License: This is an open-access article distributed under the terms of the Creative Commons Attribution License, which permits unrestricted use, distribution, and reproduction in any medium, provided the original work is properly cited.
License URL: https://creativecommons.org/licenses/by/2.0/

Conference date: November 9-12, 2006
Conference name: 27th Annual Conference of Association of Medical Physicists of India
Conference Acronym: AMPICON-2006
Conference location: Hotel Mayfair Lagoon, Bhubaneswar
Conference sponsor: Hemalata Hospitals and Research Centre:
Conference theme:

==============================
[Downloaded free from http://www.jmp.org.in on Friday, July 29, 2011, IP: 130.14.254.25] || Click here to download free Android application for t

International Conference on Medical Physics
27th Annual Conference of AMPI (AMPICON - 2006)
November 9-12, 2006, Hotel Mayfair Lagoon, Bhubaneswar,
(Host: Hemalata Hospitals and Research Centre)

### SYNOPSES OF POSTER PAPERS

### P-01

### Effect of number of iterations on treatment time monitoring units, number of segments and dose to target and normal structures in IMRT

KR Muralidhar, P Narayana Murthy, Pramod Kumar Dixit, Tirumalai Swamy, Madhusudhan Sresty, Shiyama, Rajanesh.
*Indoamerican Cancer Institute and Research Center, Road No 14, Banjara Hills, Hyderabad - 500 034, India*
E-mail: krmuralidhar@rediffmail.com

**Introduction:** To study the effect of number of Iterations on treatment time monitoring units (MU), number of segments and dose to target and normal structures in IMRT.

**Methods and Results:** A patient with Ca. rectum case has been chosen for this study. Target volume 1 is created by 0.5 cm auto margin to tumor (rectum). The normal structures like bladder, left femoral head and right femoral head contours are drawn. Five intensity modulated beams, each of 6MV, are used with gantry angles 30, 130, 280, 315, 350. All beams are aimed to the center of target volume 1. Dose prescription is given in IMRT. For target and normal structures the respective weight, power and rank is given. The treatment planning system (CMS, XIO) consists of inverse planning facility. The convolution algorithm is used. Primus Plus Linear accelerator from siemens with MLC 58 leaves is used for Step and Shoots method. Studies have been done for 30, 40, 50, 60 and 64 iterations. For each iteration, the dose volume histogram, the IMRT optimization summary, dose volume histogram index, MLC segments were derived. Graphs are drawn on the number of iterations (on x-axis) vs. dose to target and normal structures (on y-axis) and also number of iterations vs. number of monitoring units. As the no. of iterations increases the no. of segments increased from 69 to 78 also monitoring units increased from 512.33 to 653.89.

**Conclusions:** As the number of iterations increases the treatment monitoring units and total number of segments increases. Even though the number of monitoring units increases from 512 to 654 i.e., 141MU as the number of iterations increases from 20 to 64, the dose to normal structures are in safe limits and dose conformity to the tumor is better.

Table 2: Iterations Vs No. of MU and segments

| *No. of* | *Monitoring units* | | | | | |
|---|---|---|---|---|---|---|
| *Iterations* | *AP* | *LAO* | *LPO* | *RAO* | *RPO* | *Total MU* |
| 20 | 87.67 | 98.64 | 108.49 | 94.03 | 123.5 | 512.33 |
| 30 | 75.17 | 106.2 | 109.03 | 108.66 | 134.07 | 533.13 |
| 40 | 76.07 | 116.1 | 126.54 | 111.91 | 156.8 | 587.42 |
| 50 | 81.74 | 125.89 | 124.9 | 113.9 | 161.24 | 607.67 |
| 60 | 78.92 | 117.09 | 132.98 | 126.32 | 165.17 | 620.48 |
| 64 | 86.94 | 102.08 | 158.63 | 139.24 | 167 | 653.89 |
| | | | Segments | | | Total |
| 20 | 14 | 14 | 13 | 15 | 13 | 69 |
| 30 | 12 | 15 | 12 | 16 | 14 | 69 |
| 40 | 12 | 15 | 15 | 16 | 16 | 74 |
| 50 | 13 | 16 | 15 | 16 | 16 | 76 |
| 60 | 12 | 16 | 15 | 17 | 16 | 76 |
| 64 | 13 | 14 | 17 | 18 | 16 | 78 |

Table 1: Iterations vs. dose to target and normal structures

| | *Target volume 1* | | | *Bladder* | | | *Right femur head* | | | *Left femur head* | | |
|---|---|---|---|---|---|---|---|---|---|---|---|---|
| *No. of iterations* | *Min dose* | *Max. dose* | *Mean dose* | *Min dose* | *Max. dose* | *Mean dose* | *Min dose* | *Max. dose* | *Mean dose* | *Min. dose* | *Max. dose* | *Mean Dose* |
| 20 | 2356 | 4496 | 4027 | 805 | 4083 | 2388 | 1830 | 3740 | 2417 | 932 | 3698 | 2195 |
| 30 | 2414 | 4498 | 4033 | 796 | 4096 | 2367 | 1837 | 3728 | 2405 | 940 | 3734 | 2185 |
| 40 | 2444 | 4483 | 4034 | 810 | 4063 | 2369 | 1830 | 3653 | 2386 | 939 | 3652 | 2177 |
| 50 | 2480 | 4488 | 4033 | 812 | 4092 | 2365 | 1810 | 3646 | 2385 | 949 | 3672 | 2180 |
| 60 | 2509 | 4578 | 4039 | 823 | 4106 | 2366 | 1763 | 3683 | 2368 | 941 | 3638 | 2154 |
| 64 | 2526 | 4553 | 4035 | 823 | 4129 | 2367 | 1780 | 3718 | 2387 | 922 | 3708 | 2151 |

### P-02

### Comparative study of superposition, fast superposition and convolution algorithms in open, 3DCRT and IMRT techniques for various diaganosis

KR Muralidhar, P Narayana Murthy, Madhusudhan Sresty, Tirumalai Swamy, Rajanesh, Pramod Kumar Dixit, Shiyama.
*Indoamerican Cancer Institute and Research Center, Road No 14, Banjara Hills, Hyderabad - 500 034, India*
E-mail: krmuralidhar@rediffmail.com

**Introduction:** Our aim is to compare the three algorithms namely convolution, superposition and fast superposition algorithms in open, 3DCRT and IMRT Techniques for various diagnosis. We have taken number of monitoring units, segments in IMRT techniques and dose conformity to the tumor for comparison.

**Methods and Results:** Convolution, superpostion and fast superposition algorithms were used in XIO planning system (Computerized Medical Systems, USA) to calculate doses in treatment plans using photon beams of 6 MV nominal energy from a Primusplus Linear accelerator (Siemens Medical Systems, USA). The three said algorithms were used for open, 3DCRT and IMRT for four kinds of diagnosis (Hypopharynx, lung, esophagus and prostate). The dose conformity, Monitoring units, No. of segments in IMRT is analyzed. The percentage of variation between these algorithms is derived on the basis of above three factors. The system (CMS, XIO Planning system) consists of inverse planning facility. The convolution algorithm is used. Primus Plus Linear accelerator from siemens with MLC 58 leaves is used for Step and shoots method. Prostate, lung, esophagus and hypopharynx cases were taken for our study. We planned with 2D, 3D, IMRT techniques. In each case we used three algorithms for comparison. First we planned each case with 2D, 3DCRT and IMRT, with convolution techniques. Second, we planned each case with 2D, 3D and IMRT, with superposition technique. Finally, we planned each case with 2D, 3D and IMRT, with fast superposition. The readings

The PDF is available for free download from a site hosted by Medknow Publications (www.medknow.com).

[Downloaded free from http://www.jmp.org.in on Friday, July 29, 2011, IP: 130.14.254.25] || Click here to download free Android application for t

Table 1: Percentage of variation with prescribed dose to all algorithms and variation between algorithms

| S. No. | Technique | Organ | Prescribed dose | Variations with respect to prescribed dose | | | Max % variation between algorithms |
|---|---|---|---|---|---|---|---|
| | | | | Convolution | Superposition | Fast superposition | |
| 1 | OPEN | Prostate | 4370 | -0.32 | -0.2 | -0.64 | 0.44 |
| 2 | 3DCRT | Prostate | 4370 | -0.71 | -.93 | -1.3 | 0.59 |
| 3 | IMRT | Prostate | 4370 | 1.37 | 1.39 | 0.8 | 0.59 |
| 4 | OPEN | Hypopharynx | 7332 | 2.89 | 3.36 | 3.31 | 0.47 |
| 5 | 3DCRT | Hypopharynx | 7332 | 2.34 | 2.68 | 2.55 | 0.34 |
| 6 | IMRT | Hypopharynx | 7332 | -0.38 | -0.91 | -1.1 | 0.72 |
| 7 | OPEN | Esophagus | 3962 | 2.47 | 1.91 | 1.61 | 0.86 |
| 8 | 3DCRT | Esophagus | 3962 | 2.8 | 2.62 | 2.42 | 0.38 |
| 9 | IMRT | Esophagus | 3962 | 0.96 | 0.58 | 0.4 | 0.56 |
| 10 | OPEN | Lung | 3066 | 1.3 | 1.47 | 1.17 | 0.3 |
| 11 | 3DCRT | Lung | 3066 | 0.91 | 0.91 | 0.55 | 0.36 |
| 12 | IMRT | Lung | 3066 | -0.61 | -0.55 | -0.49 | 0.12 |

obtained are tabulated.

**Conclusions:** Monitor units are almost equal for IMRT, 3DCRT and open techniques for each individual case. Segments are almost same in IMRT cases. The mean doses to tumor values are very near in case of convolution algorithm for head and neck and pelvic cases. Maximum Percentage of variation between three algorithms is 0.86% by taking mean dose of the tumor value as criteria. Fast superposition algorithm values are suitable for esophagus and lung.

**P-03**

**MDI of SSD and SAD technique developed in SNDH**

DV Shilvant, CJ Tamane*, SB Pangam, MD Raees, SK Aynoor.
*Departments of Radiotherapy and *Radiation Oncology, Seth Nandlal Dhoot Hospital (Charitable), A-1, A-2, MIDC, Chikalthana, Aurangabad-431210, (M.S.), India*
E-mail: sndh@dataone.in; deepakshilvant@gmail.com

**Introduction:** To develop Mechanical Distance Indicator (MDI) for both SSD and SAD Technique used in patient set up during radiotherapy treatment.

**Materials and Methods:** Role of ODI is vital during patient set up in radiotherapy unit. Obstruction of ODI due to design or unavailability of spares for short duration is the need for MDI conventionally called as 'T' rod. The available T rod and vertical scale mounted on couch had limitations. This can be used for SSD technique only and the couch vertical scale viewing is not comfortable during certain setup. The modified MDI developed in SNDH can be used for both SSD and SAD Technique with precision. The MDI was prepared with hollow SS Pipes, spring, Teflon bush and locking pin. The rod extreme end is fitted with Teflon smooth pointer tip and this assembly is movable. The scale is mounted on rod, thus precise displacement of required depth of maximum 10 cm is achieved. This function is utilized during SAD Technique. When spring is released, the measurement of T rod matches with the SSD of radiotherapy unit.

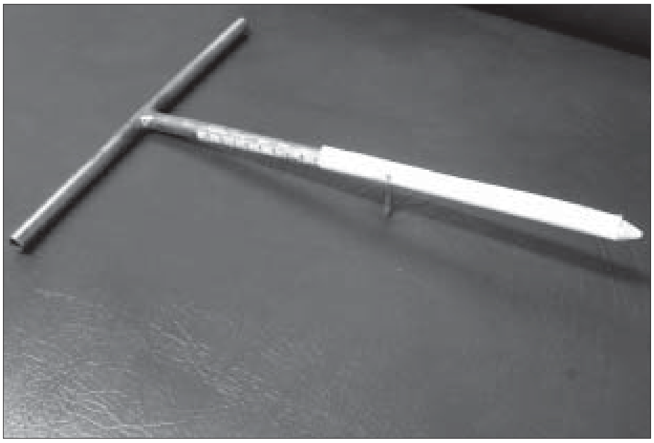

**Figure 1:** Mechanical distance indicator (MDI)

**Discussion:** Obstruction of ODI during patient setup is due to obesity. In case of 'Post Cricoid region setup', the chin obstructs the ODI and in oblique fields or with breast cone attachment, the ODI cannot be visible on patient surface. In SSD technique, the lasers can be used as an alternative, but it should be aligned properly with isocenter as during normal day workout it may get disturbed, hence can be used after confirmation. The available 'T rod' is rigid one and is normally used for SSD setup. As it touches the patient's skin, it is found difficult while removing after setup. The new modified MDI overcomes this problem because the tip of this device is made up of soft teflon pointer and as the end part is movable, it helps after patient set up. By just pushing, the device can be taken out without any injury to the patient's skin. In remote area hospitals, availability of ODI spares and the personnel who can replace the part is often uncertain. Therefore, without bothering the patient and without loosing the quality, the MDI developed in SNDH can be utilized to avoid down time and provide treatment to the heavy workload of patients. The same MDI can be used in SAD technique. When spring is in a released position, it matches the isocenter of the unit. Initially adjust the patient with isocenter, then with required depth, the tip can be pushed inside with matching scale on it and lift the couch up-word till the pointer touches the skin. Thus patient setup with SAD technique is completed. Then the device can be taken out as discussed above in SSD technique. The maximum setup depth of 10 cm is achieved with this device.

**Conclusion:**

- The MDI developed in SNDH is a precise lightweight instrument.
- The single device can be used in both SSD and SAD technique for maximum 10 cm-setup depth.
- This device is very useful for avoiding down time and treatment interruption.
- The simplicity of device demands a normal workshop facility for preparation of this inexpensive precise instrument.

**Acknowledgement:** I would like to acknowledge the kind cooperation from our hospital maintenance department. I am taking this opportunity to thank Mr. Dilip Jamdade, Supervisor of Sharda Engineering Works, Chikalthana, Aurangabad for the skillful work and crystallizing our ideas in the form of lucid device.

**P-04**

**New treatment approach for breast conservation therapy with mammosite applicator: Experience at Indo-American cancer institute**

KR Muralidhar, P Narayana Murthy, Pramod Kumar Dixit, Tirumalai Swamy, Madhusudhan Sresty, Shiyama, Rajanesh.
*Indoamerican Cancer Institute and Research Center, Road No 14, Banjara Hills, Hyderabad - 500 034, India*
E-mail: krmuralidhar@rediffmail.com

**Introduction:** This study, first of its kind in India, evaluates a new approach of doing breast conservation therapy with Mammosite

This PDF is available for free download from a site hosted by Medknow Publications (www.medknow.com).

[Downloaded free from http://www.jmp.org.in on Friday, July 29, 2011, IP: 130.14.254.25] || Click here to download free Android application for t

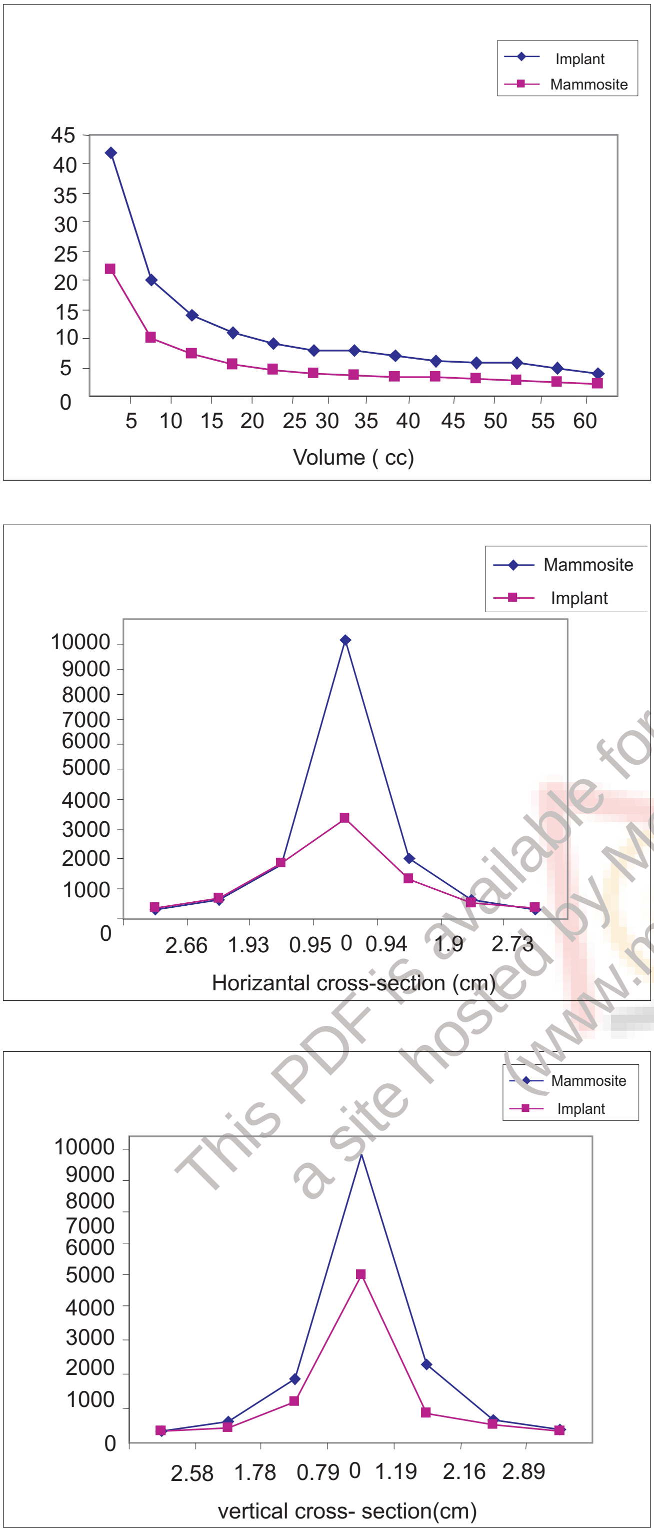

**Figure 1:** Dose volume histogram applicator to make breast brachytherapy simpler and more accurate. Advantages and disadvantages of this procedure are discussed.

**Materials and Methods:** A breast case planned with Mammosite was compared with interstitial brachytherapy. The criteria for comparison were the same for both methods. Optimal dose to the planning target volume (PTV) and sparing of the organs at risk (OAR). Computed Tomography scans of patients were obtained before initiation of treatment. A commercial three dimensional planning system was used for analysis of dosimetry including dose-volume histograms. These data were compared to the patient treated with interestial high-dose-rate brachytherapy. Vertical and horizontal cross-sectional doses of mammosite and implant cases were also compared.

**Results:** With Mammosite device, dose coverage to the tumor is better than interstitial brachytherapy. With Mammosite the dose to the lung is 50% lesser than Interstitial brachytherapy. The Dose difference between maximum and minimum is very high in Mammosite. The total treatment time to treat with implant is 460.5 seconds whereas with Mammosite, it is 246.1s to deliver 340cGy to PTV.

**Conclusions:** The dose to lung is lower with Mammosite compared with implant brachytherapy. Cross-sectional dose is better in implant cases. The total treatment time is reduced by Mammosite device. In terms of PTV coverage, there is an advantage in using Mammosite for particular selected breast cases.

This PDF is available for free download from a site hosted by Medknow Publications (www.medknow.com)

**P-05**

**Optimization of dose inhomogeneity in tangential breast fields using multi-leaf collimator and 3D-Treatment planning system**

K Krishnamurthy, SS Sivakumar, CA Davis, R Ravichandran, Kamal El Ghamrawy. *Department of Radiotherapy, National Oncology Center, Royal Hospital, Muscat, Sultanate of Oman*
E-mail: kammarikm@yahoo.co.in

**Introduction:** Radiation dose delivery to the chest wall after total mastectomy as well as in the treatment technique of conservative breast remains complex. This is due to the irregularity in contour at the chest wall level and also varying thickness of lungs underneath. X-Ray CT images with 3 dimensional (3D) planning softwares help in displaying the dose variations at different levels below skin. Physical or dynamic wedges are commonly applied to correct for entrance obliquities which also give rise to beam hardening effects in the beam. Despite all these planning efforts, there are about 10% increased dose hot spots encountered in final plans. With Intensity Modulated Radiotherapy (IMRT) and inverse planning it is possible to overcome this problem. To overcome the effect of formation of hot spots we have tried a manual forward planning method using added beams with multi-leaf collimator (MLC) of different weightages in addition to plain medial and lateral wedged tangent beams.

**Materials and Methods:** Computerised 3D Radiation Treatment Planning System (RTPS) Eclipse (version 6.5, Varian Ag, USA) is used for treatment planning. High energy linac Clinac 2300 CD (Varian Ag, USA) having 120-leaf millennium MLC is used for the breast field tangential treatments. 15 patient treatment plans were taken up to check and compare the validity of using additional MLC fields to achieve better homogeneity in dose distributions. X-ray CT images at different transverse sections away from mid plane, were taken for 3D planning. The MLC positions and beam weightages are decided by forward planning to arrive at homogeneity in dose.

**Results:** The dose volume histograms with the present type of optimization have shown better homogeneity inside the tumour volume. The resultant dose distributions with and without presence of MLC are compared objectively.

**Conclusion:** Our results show that 3D planning helps in identifying the hotspots at different planes and attenuating the beam using MLC vanes will help in overcoming dose inhomogeneity. The presence of wedges along with beam weighting and the use of MLC can be the solution in the absence of IMRT plans.

**P-06**

**Design, function and radiation safety aspects of delay tank system connected to radioactive iodine isolation wards at oncology center, Oman**

R Ravichandran, LS Arunkumar, R Sreeram, Ken Gorman, Amal Al Saadi. *Royal Hospital and DGEA, MOH, Muscat, Oman*

**Introduction:** For the management of differentiated carcinoma thyroid patients, large doses of Iodine-131 as high as 3.7 GBq (100 mCi) are administered at a time. The blood borne I-131 activity are excreted in

[Downloaded free from http://www.jmp.org.in on Friday, July 29, 2011, IP: 130.14.254.25] || Click here to download free Android application for t urine and therefore the isolation wards include toilets connected to delay tanks, which have regulated outflows into the sewerage of the hospital. Twin delay tanks are usually built and the volumes sufficient enough to have about 2 months collection, 2 months delay so as to achieve 56 to 105 days effective decay. Large volumes of the effluent tanks make the specific activity very low, before they are released to sewage treatment plants (STP). The functional and radiation safety aspects have been outlined in earlier reports.[1-4] The design and procedures followed at our new oncology center is highlighted.

**Materials and Methods:** Design: 2 underground concrete delay tanks of size 5 m x 4 m x 2 m each have storage capcity 30 Kilolitres (75%). They have 2 submergible pumps, float switches and ultrasonic monitoring systems. Using a Business Management System (BMS) different informations are monitored at control room of engineering department. Status of inlet valves, level indications, % volume of effluents are monitored and hooter alarm is included. Medical physics and engineering departments are responsible for release of waste effluents to STP of hospital.

**Functioning:** High and low level sensors at 180 cm and 60 cm heights from floor provide a total volume of 24 $m^3$ (60%) at the time of releasing. However dilution volume remains 30 Kilolitres. With collection rate of 200 Ltrs/day (2 patients), 1400 Ltrs/wk, for 2 months filling 11200 Ltrs and additional volume of 8000 Ltrs together makes one tank sufficient for total 16 patients/ 2 months. Registers are maintained to monitor weekly status of fulling of tanks.

**Procedures:** Entry valve of the filled tank gets closed, valve for the other tank opened; Radiation Safety Officer (RSO) indicates tentative date of release of the filled tank. On the date of release, RSO and Engineer inspects delay tank site, collects sample and after confirming the activity of effluents by external monitoring and sample counting (activity /ml) in well counter, the effluents are released. Probe 489-110D End Window GM Survey meter, Model 190 (Inovision, USA) is used for external monitoring. Medical Spectrometer with well counter (Atom Lab950PC, Biodex, USA) is used for well counting at I-131 window. Relevant documents are maintained in the departments.

**Results:** The I-131 patients admissions started during February, 2006. So far 8 patients were admitted. Tank No.2 and Tank No.1 were closed on 3, May, 2006 and 15, July, 2006 respectively. The effluents of Tank No. 2 were released on 3, July, 2006 after following all procedures. The calculated activity on the date of release was 3.7 MBq (100 uCi) in 30 Kilolitres, which corresponds to a specific activity of 0.12 Bq/ml. 200 sample taken from the tank by external monitoring did not show activity levels more than background. 1 ml samples counted with well counter registered counts comparable to counts/ml of the municipal water.

**Conclusion:** This paper illustrates the efficacy of the application of the method highlighted in earlier reports by us. The radiation safety regulations and procedures followed at different steps show the need for implementation of procedures in such situations. We hope that this report will be useful to new institutions starting these procedures.

### References

1. Ravichandran R, Jayasree U, Keshava SL, Devaru S. Proc. 23rd IARP Conf: Amritsar; 1997.
2. Ravichandran R, Pant GS. Radiation Safety for Unsealed Sources. Himalaya Pubs: 1998. p. 102-14.
3. Ravichandran R, Pant GS. Radiation Safety for Unsealed Sources. 2nd ed. 2000. p. 237-48.
4. Arunkumar LS. Guidelines on I-131 Therapeutic Uses. DGEA MOH document: Oman; 2006.

### P-07

### Monte Carlo simulation of a proposed design of blood irradiator for its safety assessment

Aruna Kaushik, K Biju, Kapil Deo Singh*, A Sharma, T Palani Selvam.
*Radiological Physics and Advisory Division, BARC, CT and CRS, Anushaktinagar, Mumbai - 400 094,*Health Physics Division, BARC, Trombay, Mumbai - 400 085, India*
E-mail: kaushik_aruna@rediffmail.com

**Introduction:** Blood and blood components are irradiated in a self-contained gamma irradiation facility called blood irradiator. Irradiation of blood is necessary to prevent occurrence of complications like Graft Versus Host Disease (GVHD), which occurs when viable T-lymphocytes in transfused blood or blood components engraft, multiply and react against the tissues of the recipient. GVHD may be seen when blood components are transfused to immunocompromised recipients, such as bone marrow transplant patients, patients with malignancies receiving aggressive chemotherapy and persons with congenital immune deficiency syndrome. A design of a compact single bag blood irradiator is proposed to contain three $^{60}Co$ pencils of total activity 10.175 TBq (275 Ci). Tungsten, lead and steel are used as the shielding material in a complex geometrical arrangement. There is a curved passage meant for the blood bag to go through it and get irradiated at the center. The paper aims at assessing the shielding adequacy of the proposed design of the blood irradiator using the Monte Carlo - based MCNP Code. We have also used an analytical method for comparison purpose.

**Materials and Methods:** The $^{60}Co$ sources used in the irradiator have a total length of 20.9 cm and an active length of 17.8 cm. The diameter of each source pencil is 1.15 cm. The shielding adequacy of the irradiator was assessed by using Monte Carlo as well as analytical methods.

**Monte Carlo code and Calculations:** The geometry of the proposed design of the irradiator was simulated using the Monte Carlo Code MCNP.[1] Techniques like importance sampling, photon splitting, Russian roulette and directional biasing were used so that sufficient numbers of scores were obtained to have a converged statistical estimate with an acceptable uncertainty. Hundred million histories were simulated. Energy fluence was estimated initially at points of interest, which was subsequently converted into exposure rate in units of Roentgen/hour (R/h). Analytical Method: Exposure rate, X(P) (in R/h), at any point P at a distance, R was computed using the following formula:

$$X(P) = \frac{2 \times K \times S_1}{4\pi} \int_{x=0}^{x=8.9} \frac{B_1 B_2 B_3 e^{-\mu R1} e^{-\mu 2R2} e^{-\mu 3R3}}{R^2} dx$$

where K is conversion factor to convert activity (in curie) to exposure rate (in R/h); $S_l$ is activity of the pencil per unit length (Ci/cm); $B_1$, $B_2$ and $B_3$ are dose build-up factors for various shielding materials; $m_1$, $m_2$ and $m_3$ are attenuation coefficients for various shielding materials; $R_1$, $R_2$ and $R_3$ are the distances traversed by radiation in the shielding materials.

**Results and Discussion:** As per American National Standard Institute (ANSI) Standards (ANSI N433.1, 1977), the design of the blood irradiator (Category I) should be such that the exposure rate from leakage radiation measured at any position 1 meter from the accessible surface of the irradiator shall not exceed 2 mR/h and at any position 5 cm from the accessible surface of the irradiator shall not exceed 20 mR/h. The proposed design of the blood irradiator was assessed from radiation safety standpoint. The radiation level all around the proposed design of the blood irradiator was observed to be higher than the permissible limit. The variation in the results obtained using both the Monte Carlo and analytical methods was of the order of a factor of two. The proposed design was modified by introducing 2 cm lead all around the cabinet and 3 cm thick lead plugs on the top and bottom of the pipe meant for the passage of the blood bag. Added shielding thickness reduced the exposure rate to values less than 20 mR/h at 5 cm and to 2 mR/h at 100 cm from the surface of the irradiator as per the requirement of ANSI Standard.

**Conclusions:** MCNP code proved to be a very useful tool in simulating the complex geometry of the proposed blood irradiator that included curvature of the pipe and different shapes of the shielding materials. The techniques like importance sampling and directional biasing were very helpful in solving this deep penetration problem.

**Acknowledgements:** Authors wish to thank Shri H. S. Kushwaha, Director, HS and EG for the encouragement during the work. Authors are also grateful to Shri Sunil Sunny, IGCAR, AERB, Kalpakkam for valuable technical discussions.

This PDF is available for free download from a site hosted by Medknow Publications (www.medknow.com)

[Downloaded free from http://www.jmp.org.in on Friday, July 29, 2011, IP: 130.14.254.25] || Click here to download free Android application for t

### References

1. Manual on MCNP. A General Monte Carlo N - Particle Transport Code. Los Alamos Laboratory: 1983.

### P-08
### Custom made patient positioning devices for radiotherapy set up in linear accelerator treatments

R Ravichandran, NK Babu, Bakshish Singh, S Namrata, BK Mohanti, Kamal El Ghamrawy. *Department of Radiotherapy, National Oncology Center, Royal Hospital, Muscat, Sultabate of Oman*

**Introduction:** Accurate patient positioning in a normal comfortable posture with ability to remain in that treatment position for a long duration forms the basic criterion for correct treatment dose delivery in radiotherapy. Further immobilization of the patient in the expected posture is achieved by orfit thermoplastic casts. Ravichandran *et al* emphasized the need for accurate positioning of the patient as a pre-requisite for successful radiotherapy. Patient support devices commercially available may not tailor to the individual patient needs and in many institutions in house fabrication of these devices are developed. Radiotherapy cancer treatment facility was started recently in Oman. In the initial period of one year we were in the process of procuring and establishing patient support aids for the type of patients encountered. We attempted to develop positioning devices in house, for radiotherapy of breast, pelvis and cranio-spinal areas using hard thermocole thick sheets. The efficacy of their application in the treatments are outlined.

**Materials and Methods:** Patient support devices are made with Styrofoam sheets supplied by M/s Arply Medical Systems, France. These boards are quite stable and maintain position without sagging, for a distributed load of more than 80 Kg. The density is $\rho$ = 34 Kg/m$^3$ which is less than that of water ($\rho$ = 1000 Kg/m$^3$) and therefore light to handle by the positioning technicians. Because of low density, these sheets do not provide appreciable scattering effect during irradiation; and also will have less scatter than that of carbon fibre breast boards which are commercially available. Transmission measurements (with a field size of 20 cm x 20 cm) using ion chamber showed an attenuation factor of 0.98 for 12 cm thickness, which corresponds to $\mu$ = 0.0017 cm-1 for 6 MV X-ray beam. Breast board (10$^o$ and 15$^o$ angulations) along with arm supports and mobile platform having longitudinal graduations were made using the Styrofoam sheets. A prone pelvic board was designed for the treatments of cancer cervix, prostate and bladder and they are used along with thermoplastic fixing. A supine pelvic board has knee rests for providing flat back properly resting offering comfortable position. A prone position face rest is constructed along with sloping chest rest, has been made for cranio-spinal axis irradiation in paediatric patients. The reproducibility of patient treatments was checked with repeated images acquired in high energy linear accelerator (Clinic 2300 CD, M/s Varian USA) amorphous silicon phosphor electronic portal imaging device (EPID) and anatomical matching method software in Varis patient record and verify system.

**Results:** Presence of angulated breast board on the treatment table helped in reducing the need for collimator rotation and better matching between tangents and supraclavicular fields. Randomly taken 25 medial and 25 lateral EPID images in 42 patients showed mean variations within 3 mm in tangent breast fields and rotation within 1.2 mm. The use of prone pelvic board helped in achieving better treatment plan improving the dose volume histogram (DVH) of bladder dose. The comparison of EPID beam edge plots with and without pelvic board has shown improved reproducibility of treatments with the application of the board. Using the prone face rest good positioning reproducibility could be achieved in paediatric patients.

**Discussion:** In the above work we have discussed the efficacy of patient support boards for achieving acceptable variations in repeated treatments. It is emphasized that the devices described here do not take away the purpose of thermoplastic immobilization requirement, but add to comfort to patient support below the patient. However the thermoplastic casts will be continue to remain for fixing the patient in planned posture. The cancer hospitals in developing countries have financial constraints and finding difficulty to meet the increasing demands for purchasing innumerable patient support aids. Thermoplastic moulds have become routine immobilization method adopted globally. These moulds along with this simple, light weight thermocole boards could be solution for small centers who can deliver accurate radiotherapy even with less expensive isotope machines.

### References

1. Ravichandran R, Sathiyanarayanan VK, Supe SS, Shariff MH, Kurup PGG. Accuracy of patient set up and on-line imaging in radiotherapy. Clin Radiother Oncol XI,1,1996:58-61.

### P-09
### Comparison of different calibration techniques for $^{192}$IR high dose rate remote after loading system

Rajib L Sha, C Ramkrishna Rao, P Krishna Reddy, B Mishra. *Dept of Radiation Physics, MNJ Institute of Oncology and RCC, Red Hills, Hyderabad - 500004, India*
E-mail: rajibsha@gmail.com

**Introduction:** Measurement of reference air kerma rate or strength of High Dose Rate (HDR) $^{192}$Ir source has to be essentially carried out to achieve desirable accuracy for clinical dosimetry. Strength of the source can be carried out directly by using re-entrant type. (Well type) chamber and dose rate / output by reference air kerma method. In MNJ Institute of oncology and RCC, both calibration techniques were employed, during source loading. The paper discusses the comparison of methodology and the results of the two calibration techniques with source certificate.

**Materials and Methods:** Reference Air Kerma method: Two equidistant (10 cm each) method for "in air" measurement of air kerma rate of the $^{192}$Ir HDR brachy therapy sources was measured with the help of 0.6cc farmer type ion chamber and PTW UNIDOS Secondary Standard Dosimeter. A special perspex jig made by MNJIO and RCC was used for mounting the ion chamber and sources at a fixed distance of 10 cm in a reproducible geometry. The jig was mounted on a rigid stand to achieve a measurement height greater than 1 m from the floor level and the measurement setup was arranged to maintain a minimum distance of 1 m from all sides of the room to minimize the scatter contribution. To get the maximum dose dwell position for both channels, three steps procedure followed such as 20 dwell positions, 3 dwell positions and 1 dwell position (first two steps in current mode and final step in current integrated mode) in 2.5 mm step size source movement. Finally reference air kerma rate was calculated by the following formula

Sr = Mr(nc) x Ktp x C.F.(Gy/c) x Inv sq factor x B (build up factor) x time correction

**Well Type Chamber:** A special re-entrant type chamber(PTW FREIBURG) made by Nucletron was used to measure the strength of the source directly, followed in the three steps procedure to get the maximum dose dwell position. All the three steps procedure was carried out by PTW UNIDOS SSD in current mode for 2.5 mm step size source movement.

Finally the strength of the source was calculated by using the following formula:

A = Mr (amp) x C.F.(Ci/amp) x $P_{ion}$ x Ktp

Where, $P_{ion}$ = the reciprocal of Ion collection efficiency factor $A_{ion}$.

$A_{ion}$ = 4/3 - (1/3) Q1/Q2

Where, Q1 and Q2 are the charge (or current) readings at nominal 300V and 150V respectively.

$A_{ion}$ was found 0.996, so $P_{ion}$ is nearly equal to 1.

Ktp = temp, pressure correction factor

**Results and Discussion:** From the Reference Air Kerma method, the reference air kerma rate was calculated as 4.0928043 x 10^4 microGy/hr which is equal to the strength of the source as 9.791Ci by using the convertion formula 1 mCi = 4.18 microGy/hr from literature. From the Well -type chamber method, the strength of the source was calculated as 9.808 Ci. From source certificate, the strength of the source was found 9.782 Ci after applying decay correction for 20 days (d.f. =0.821). Finally comparision was done between the above said

[Downloaded free from http://www.jmp.org.in on Friday, July 29, 2011, IP: 130.14.254.25] || Click here to download free Android application for t techniques with source certificate value. Reference Air Kerma method and well type method deviates 0.1%, 0.26% respectively with source certificate value and both calibration techniques deviates 0.173% from each other.

**Conclusion:** The comparison between Reference Air Kerma technique and Well type technique with source certificate for calibration of Ir-192 High Dose Rate remote after loading system was found within tolerance limit (< ±2%). Both the calibration methodology and results were found to be ideal and satisfactory for clinical use.

**P-10**

**Linearity of low monitor units for 6 and 15 MV beams**

Janhavi Bhangle, Shrikant Deshpande, VK Sathiya Narayanan. *Department of Radiation Oncology, Ruby Hall Clinic, Pune, India*

**Introduction:** Siemens ONCOR Impression Plus linear accelerator with photon beam energies of 6MV and 15 MV equipped with double-focused MLC has been installed in our clinic. Siemens ONCOR linear accelerators are designed to deliver intensity modulated fields using a multiple static field i.e., Step-and-Shoot technique. In this type of IMRT delivery, multiple segments are the result of the optimization process to conform the dose to the target volume. These segments are delivered in a stack arrangement. Each beam segment is delivered separately with the radiation beam turned off when the MLC moves to take a shape between segments. The composite of dose increment delivered to each segment delivers the planned intensity modulation. The accuracy of intensity modulation depends on the capabilities and limitations of the linear accelerator equipped with the multi-leaf collimator to deliver low MU segments. The operation of the linear accelerator delivering low MU segments needs to be investigated prior to commission the LINAC for IMRT. The linearity of dose in low MU region and its inter-segment variation for both 6 and 15MV photon energies were investigated in this work.

**Materials and Methods:** Dose monitor linearity and inter-segment response at small monitor units for 6MV and 15MV were examined using a FC65G ionization chamber connected to DOSE1 electrometer. The measurements were done at 100 cm SSD with the chamber at $d_{max}$ in the SP34 slab phantom. The collimator settings were 10 cm ×10 cm. For low MU linearity, ionization readings were recorded for 100MU, 50MU, 20MU, 10MU, 5MU and 2MU. These readings were normalized to 2MU and to 100MU reading and the corresponding percentage variation for all other MUs calculated. For inter-segment dose variation, the same set-up as described above was used. For the collimator setting of 10 cm ×10 cm the ionizations were recorded for 10MU. It is compared with the cumulative ionization of 5MU measured two times; 2MU measured five times, 1MU measured ten times. Similarly, ionization recorded for 15MU was compared with the cumulative ionization of 5MU measured three times; 3MU measured five times, 1MU measured fifteen times.

**Results and Conclusion:** For 6MV and 15MV, the deviations of relative ionization per MU for the range of monitor units examined from the ionization reading at 100MU and from that at 2MU were varying within 1.7%. For 6MV, the variation in the cumulative ionization recorded for 5MU measured two times was -1.002%. The variation in the cumulative ionization recorded for 2MU measured five times and that for 1MU measured ten times from the ionization recorded for 10MU was within 1.0%. Similarly, for 15MU the variation in the cumulative ionization recorded for 5MU measured three times was -1.45%, while that measured for 3MU five times and 1MU fifteen times was 0.31% and 1.012% respectively. For 15MV, the variation in the cumulative ionization recorded for 5MU measured two times from the ionization recorded for 10MU was 1.06%; for 2MU measured five times, the variation was 0.45%. But, for the cumulative ionization recorded for 1MU ten times the variation was found 16%. For 15MU, the variation for 5MU measured three times and 3MU measured five times; from the ionization recorded for 15MU was0.245 and -0.209% respectively. The variation in the cumulative ionization for 1MU measured fifteen times from that of 15MU was found to be 13.29%. For 6 MV beam, the linearity of the low MU segments such as 2 MU is within 2%.

**P-11**

**Quality assurance for double-focused multileaf collimator-based intensity modulated radiotherapy**

KR Muralidhar, P Narayana Murthy, Pramod Kumar Dixit, Tirumalai Swamy, Madhusudhan Sresty, Shiyama, Rajanesh. *Indoamerican Cancer Institute and Research Center, Road No 14, Banjara Hills, Hyderabad - 500 034, India*
E-mail: krmuralidhar@rediffmail.com

**Introduction:** To verify the geometric and dosimetric characteristics of Double-Focused Multileaf collimator. Materials and Methods: Intensity-modulated radiation therapy (IMRT) is an advanced mode of high-precision radiotherapy that utilizes the computer-controlled multileaf collimator (MLC) to deliver precise radiation dose to tumor by sparing critical organs and normal structures. The complexity of IMRT techniques demands a high level of quality control both in the operation of the equipment and in the delivery of treatment to individual patients. The purpose of this paper is therefore to apply the different techniques available, concentrating on the use of multileaf collimators and to consider the necessary quality control requirements for clinical applications. The present studies are on step and shoot method in siemens primus plus linear accelerator. The following are the some of the QA tests on dual-focused MLC.

1. Slit Pattern Test: To determine the dosimetric leaf position using dose profile analysis. In this 10 preprogrammed settings were used.
2. Abutment region test: To identify leaf position errors at different leave abutment positions. In this five preprogrammed settings were used.
3. MLC Alignment test: Misalignment of the MLC relative to the isocenter can be evaluated. In this film was irradiated with two complementary leaf patterns and calibrated with third image. The symmetry indicates perfect adjustment.
4. Communication and timing error test: In this study monitor unit (MU) per segment variation at a delivery rate of 300MU/Min was analyzed.
5. Diamond-Shaped pattern test: The MLC QA fixture in siemens primus linac gives the tool for regularly checking MLC Leaf positions. The fixture has a diamond-shaped pattern that was used with the field light to compare leaf positions.
6. Strip pattern test: It can easily validate the input parameters of IMRT dose calculation algorithm, that include small field dosimetry transmitted and leakage radiation, extra focal radiation and dose calculation resolution.
7. Star pattern test: It was used to determine the radiation isocenter. This test was done by collimator rotation, gantry rotation and table rotation.
8. Square test: This test shows the accuracy of collimator and leaf positions. It was done with four collimator angles.
9. Well pattern test: It can easily validate the input parameters of IMRT dose calculation algorithm, that include small field dosimetry transmitted and leakage radiation, extra focal radiation and dose calculation resolution.
10. Monitor unit linearity: To verify the monitor unit (MU) linearity over the range used in IMRT. Meter readings were taken for different MU. "Linearity (%) was calculated.
11. Monitor unit stability: To verity the monitor unit stability over the IMRT treatment conditions. Reading were measured for 20x5 MU in the first procedure. Next readings taken for 5x20 MU. % of difference was calculated.
12. Interleaf, Intraleaf transmission: Interleaf and intraleaf transmissions were measured by irradiating the EDR2 film with preprogrammed settings. RIT software was used.
13. Light Field projection: This test provides a quick visual assessment of the MLC to precision of about 0.5 mm, both on and off the axis and is useful when trouble shooting problems.
14. Intensity level verification: The delivery of different intensity levels has to be validated. To validate this feature triangular bar strips of different intensities were used. The triangular definition is used to

This PDF is available for free download from a site hosted by Medknow Publications (www.medknow.com).

[Downloaded free from http://www.jmp.org.in on Friday, July 29, 2011, IP: 130.14.254.25] || Click here to download free Android application for t verify the collimator rotation.

15. Isocenter verification: Digital reconstruction radiograph images and Electronic portal were used for verification of isocenter setup consistency between the treatment planning and the actual treatment.
16. Intensity pattern verification: Intensity patterns and their associated segments are created from the treatment planning system. Intensity patterns were verified with patient anatomy using double-exposed electronic portal images.
17. Point Dose verification: Patient plan was created on CT scanned phantom in TPS using the beam configuration of the patient. Point dose measurements were done in phantom and compared with TPS data.
18. Dose profile verification: This test was done by comparing the calculated and the film measured isodose plots for a single field from a multiple field in IMRT plan.
19. Tongue and groove effect: This test was done by double exposing a film with two matched MLC shapes.
20. Largest leaf extent verification: This study can obtain the largest extent of the leaves on the DRR in the BEV window.

**Results and conclusions:** All these tests were performed with ionization chamber and EDR2 films. Films were scanned with VXR-16 dosimetry pro film scanner. RIT software was used to analyse the exposed films. All results are with in the acceptable limits.

**P-12**

**Commissioning of Moduleaf Mini-MLC for Stereotaxy**

VK Sathiya Narayanan, Shrikant Deshpande, Janhavi Bangle, Vikram Maiya, Kumaraswamy, Sumit Basu. *Department of Radiation of Oncology, Ruby Hall Clinic Pune - 411001, India*

**Introduction:** Moduleaf is a miniMLC, has been commissioned for conformal beams and Intensity modulated stereotactic radiation therapy (SRT), conformal arc and IMRS for stereotactic radio surgery (SRS). Some of the essential dosimetric properties such as leakage from the leaves, interleaf transmission and penumbra have been measured and reported.

**Materials and Methods:** Thomas Bortfeld 1 defined that MLCs having leaf width below 2-5 mm could be called as MiniMLC and the one below 2.0 mm could be called as MicroMLC. Accordingly Moduleaf is a MiniMLC. Moduleaf rests on a vertical column stand, which can be lifted up and down through motor. The stand is on a trolley is on wheels and can be moved to any location. The Moduleaf is mounted on the Siemens Linear Acclerator called Oncor Impression Plus. One of the vital characteristics of a MiniMLC is the width of the radiation penumbra. The width of the penumbra was measured from the profiles of the radiation beam at $D_{max}$ in water. An ion chamber CC01 that has a volume of 0.01cc has been used. EDR2 films were exposed and by using the film dosimetry software called OmniproIMRT (Scanditronix-Welhofer) the size of the penumbra was measured. The inter leaf transmission is also evaluated using film dosimetry. The leakage from the leaves is measured by using a 0.6cc chamber (FC65G).

**Results and Discussion:** According to the AAPM Report no.54, the recommended limit for dose gradient in the beam penumbra i.e., from 80% to 20% should be less than or equal to 3.0. In other words the dose gradient should be less than or equal to 60%/3mm. From the ion chamber (CC01) profile measurements, the penumbra turns out to be 4.1 mm, whereas from the film densitometry measurements it is exactly 3.0 mm. The leakage was measured in a phantom using the FC65G (0.6cc) and it was 2.03%. The interleaf transmission was measured using the EDR2 films and found that the maximum is below 1% in addition to the averaged leakage measured with the ion chamber mentioned above. The output factors were measured for various field sizes starting from 5x5 mm to 120x100 mm, the normalization done for 10x10 cm. The output factor for 5x5 mm is as low as 0.1676, which shows steepness of the change in the output for the change in field sizes for small fields. The MiniMLC leaves are driven by the gear and motor arrangement with a leaf positioning accuracy of 0.5 mm. The maximum field size that can be covered is 12x10 cm, which makes it suitable for treating SRT and SRS. The drift of the reticule center when the isocentric accuracy test, is performed is closer to 1.0 mm. The star shot clearly displays below 1.0 mm. The error in matching of the leaves shape (optical field) with the printouts from the Treatment Planning System is below 1.0 mm. The weight of the Moduleaf is 39 Kg. The clearance from the isocenter when the Moduleaf is mounted is 30 cm. The maximum leaf over travel is 5.5 cm and the maximum leaf speed is 1.5 cm/sec. This is an important parameter while delivering step and shoot type IMRT and IMRS. While commissioning IMRT, the calculated and delivered fluence were compared and they have correlation coefficient higher than 0.97.

**P-13**

**Monte Carlo calculations of $^{137}$CS dosimetry parameters according to TG - 43 u1 formalism**

Sridhar Sahoo, T Palani Selvam, S Kannan. *Radiological Physics and Advisory Division, Bhabha Atomic Research Centre, Mumbai, India*

**Introduction:** The purpose of the work is to calculate dosimetry data for an indigeniously developed $^{137}$Cs LDR source (BRIT, India) in compliance with AAPM TG - 43U1. The Monte Carlo code (MCNP) was used for this purpose. The calculated dose parametres are compared with published data of different $^{137}$Cs source models.

**Materials and Methods:** Radioactive source: The $^{137}$Cs source (CSA-1) produced by BRIT, INDIA has an active length of 15 mm and total length 21 mm with activity 120 mCi. The sources has a sheathing of 0.1 mm platinum and is encapsulated with SS 0.5 mm wall thickness. The source capsule has a thickness of 2 mm SS at one end and 4 mm (2 mm ceramic spacer and 2 mm SS) at other end. Monte Carlo calculations: In the Monte Carlo calculations the source was immersed in a cylindrical water phantom of 40 cm diameter and 40 cm height. Angular dose profiles as a function of radial distance, r (= 0.25-10 cm) and polar angle, q (= 0°-180°) are calculated. In the calculation water kerma was approximated to absorbed dose. Air kerma strength per mCi, $S_k$/mCi, was calculated in 5 m. dia vacuum sphere. From the calculated data, dose rate constant, L, radial dose function, g (r) and

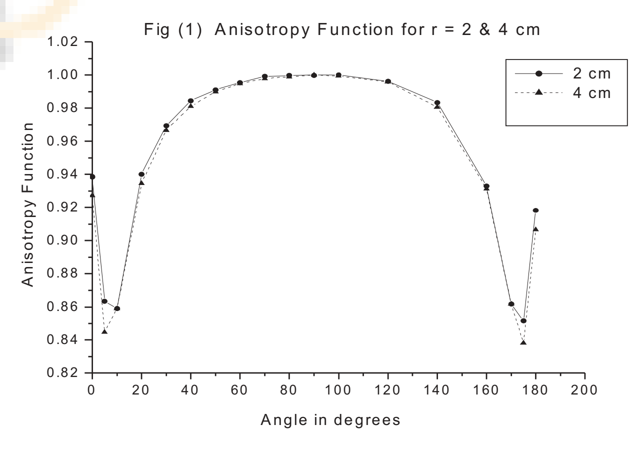

**Figure 1:** Anisotropy function for r = 2 and 4 cm

Table 1: Values od dose rate constant, L, for different $^{137}$Cs sources

| *Source* | *Dose rate constant, (cGy. $h^{-1}.U^{-1}$)* |
|---|---|
| CSA-1 (BRIT) this work | 0.945 ± 0.0005 |
| CDCS - M | 0.946 ± 0.007 |
| CDCS - J | 0.979 |
| CSM 2 | 0.807 ± 0.003 |
| CSM 3 | 0.902 ± 0.003 |
| CSM 11 | 1.096 ± 0.002 |
| Selectron / LDR | 1.107 ± 0.003 |

This PDF is available for free download from a site hosted by Medknow Publications (www.medknow.com)

[Downloaded free from http://www.jmp.org.in on Friday, July 29, 2011, IP: 130.14.254.25] || Click here to download free Android application for t anisotropy function, F (r, q) were calculaetd as per the AAPM TG - 43U1 formalism.

**Results and Conclusion:** The value of $S_k$/mCi is 2.89 ± 0.03 % cGy. $cm^2$. $h^{-1}$. $mCi^{-1}$. The calculated value of L is 0.945 ± 0.05 % cGy.$h^{-1}$.$U^{-1}$. Dose rate constants for the different designs of the $^{137}Cs$ sources are given in Table 1. The differences in the value of L are attributed to geometry factor. The anisotropy function, F (r, q) exhibits deviations from isotropy at positions on the long axis. Near longitudinal axis of the source, deviations up to 9% are observed for different q at same radial distance. This is due to oblique filtration of the source photons in the angle 0° - 20° and 160° -180°. Deviations up to 2% are observed for same polar angle at different radial distances. Anisotropy function F (r, q) versus q, for 2 and 4 cm is plotted in Figure 1. The radial dose function, g (r) data were calculated and fitted to a fifth order polynomial $g(r) = a + b_1r + b_2r^2 + b_3r^3 + b_4r^4 + b_5r^5$ between r=0.5 cm and r=10 cm and the cofficients obtained are a = 0.99847, $b_1$ = 0.0079, $b_2$ = -0.00817, $b_3$ = -0.0017, $b_4$ = -1.73523e-4 and $b_5$ = 6.4259e-6. The differences in radial dose function are in the range of 0.01-0.5% as compared with results of CDCS-M type source.

### P-14

### Analysis of East Indian kidney stones

TR Rautray, V Vijayan*, S Panigrahi. *Department of Physics, NIT, Rourkela - 769 008, India, *Institute of Physics, Bhubaneswar - 751 005, India*

**Introduction:** Urinary stone formation has been known to be one of the oldest diseases leading to hospitalization and surgery. In India it affects nearly 2 million people each year and lifetime risk is about 20% in those having this type of disorder. Different kinds of stones require different conditions for their nucleation and growth.[1] Urinary calculi are made of crystalline materials. It contains mainly calcium oxalate, calcium phosphate or mixed of others with matrix materials, depending upon the physiological derangement. The invariable presence of trace elements in urine and in renal calculi suggests that they may have some function in urolithiasis. However, although many studies have demonstrated that trace elements can influence the nucleation, aggregation and growth of calcium oxalate (CaOx) crystals in synthetic solutions and urine, whether they directly or indirectly cause or inhibit the formation of stones in the urinary tract remains open to debate. PIXE technique was used for the elemental analysis of east Indian kidney stones for better understanding of formation mechanism of kidney stones.

**Materials and Methods:** The east Indian human kidney stone samples of both sexes were collected from Kalinga Hospital, Bhubaneswar, Orissa, India in the age group of 26 to 42 years. The kidney stone samples, after cleaning and drying, were crushed to make pellets of size 13 mm diameter. PIXE analysis was carried out using the 3 MV Pelletron accelerator at Institute of Physics, Bhubaneswar, India. The targets were bombarded with 3 MeV proton beams with 20 nA beam current. A Si (Li) detector with a resolution of 170 eV at 5.9 keV with beryllium window placed at 900 to the beam direction was used to detect the characteristic X-rays emitted from the targets.

**Results and Conclusion:** Major/trace elements like P, S, Cl, K, Ca, V, Cr, Mn, Fe, Cu, Zn, Sr, Zr and Mo were estimated in the mixed and calcium oxalate stones. While S and V were below detection limit in case of the mixed stones, P, K, Sr and Zr were found to be below detection limit in case of the calcium oxalate stones. Elements like P, Cl, Ca and Fe are found to be the major elements present in the mixed stones, whereas elements like Ca and Fe are established as the major elements in the calcium oxalate stones. P (9046 ppm) was present in the mixed stones, while P was below detection limit in case of the CaOx stones. The concentration of calcium in the CaOx stone is about twice more than that in the mixed stones. There is always a significant obligatory loss of calcium in the urine (as there is in the faeces), even on a low calcium intake, simply because maintenance of the plasma ionised calcium and, therefore, of the filtered load, prevents total elimination of the calcium from the urine. So, concentration of Ca in both the types of kidney stones is very high. Concentration of Fe in the mixed stones if much higher than the concentration of Fe in the CaOx stones. Iron is not actively excreted from the body in urine or in the intestines. Iron is only lost with cells from the skin and the interior surfaces of the body

### P-15

### Optimization procedure in IMRT using objective function

Pramod Kumar Dixit, KR Muralidhar, Rajeensh Kumar, Madhusudhan Sresty, S Shiyama, Thirumalai Swamy, AK Raju. *Indo American Cancer Institute and Research Center, Banjara Hills, Road No 14, Hyderabad - 300 034, India*
E-mail: dixit.pk@gmail.com

**Introduction:** The purpose of this paper is to optimize the doses from all the beams in an IMRT procedure using the objective function with optimal parameters, so that goal of radiation therapy can be achieved.

**Materials and Methods:** IMRT is one of the best conformal procedures in radiation therapy for to deliver maximum dose to tumor and avoiding dose to the critical organs and normal tissues. The non-uniformity of dose delivery in IMRT is achieved by inverse planning which uses special mathematical function in an iterative procedure. As per the inverse planning dose specification each beam will split into hundreds or more than hundreds of beamlets of varying intensities, which will give non-uniform fluence map to achieve the desired dose. Then from the DVH it can be checked whether the nonuniform intensity of beamlets are able to achieve the prescribed dose in inverse planning or still better optimization is required. Optimization procedure in IMRT depends on the optimizing parameters. Here we have taken a function called objective function to explain the optimization procedure. The parameters used in this function are, dose volume constraint, weightage and power. When these parameters changed this effects the PTV and OAR dose and affects the optimization procedures. Images from CT are transferred to XIO, Computarized planning system. The tumor (prostate) and OARs like bladder, rectum, Rt femur and Lt Femur are contured. Planning was done using convolution algorithm with five beams. The prescribed dose for PTV is 4500cGy.The inverse planning dose specification for this plan is:

| Oar | Dose | % volume |
|---|---|---|
| Bladder | 2700 | 70 |
| | 3150 | 30 |
| Rectum | 2700 | 70 |
| | 3150 | 30 |
| Rt.Femur | 1800 | 70 |
| | 3150 | 30 |
| Lt.Femur | 1800 | 70 |
| | 3150 | 30 |

The objective function we have taken is defined as:

$$F_k(X) = W_k \sum_{J=1}^{N_k} (C_+ \{D_{kj}(X) - D_k^{max:}\})^2$$

$C_+\{a\}=\{a$, for $a \geq 0$, 0 otherwise. $F_{k:}$ is the objective function, Wk: is the weight for different organs which has to be defined, $C_{+:}$ is the constraint (for dose and volume), which has to be defined. $D_{k,j:}$ Dose at Voxel j in OAR k. $D_k^{max}$ = Tolerance dose. a: a value to be chosed by the system. Five plans are made. The parameters for all the plans are:

| | Plan 1 | | Plan 2 | | Plan 3 | | Plan 4 | | Plan 5 | |
|---|---|---|---|---|---|---|---|---|---|---|
| | wt | power | wt | power | wt | power | wt | power | wt | power |
| Bladder | 100 | 2 | 500 | 2 | 100 | 2 | 100 | 3 | 100 | 2 |
| Rectum | 100 | 2 | 400 | 2 | 100 | 2 | 100 | 3 | 100 | 2 |
| Rt.Femur | 100 | 2 | 450 | 2 | 100 | 2 | 100 | 3 | 100 | 2 |
| Lt.Femur | 100 | 2 | 450 | 2 | 100 | 2 | 100 | 3 | 100 | 2 |
| Target | 100 | 2 | 100 | 2 | 100 | 3 | 100 | 2 | 100 | 4 |

**Result:** Plan 1: 96% of tumor is covered by 97% of the prescribed dose. Bladder: 27% vol >4000cGy, Rectum 26% Vol>4000cGy, Rt and Lt femur 30% vol>3000cGy. Plan 2: Here 95% of target volume is getting only 92% of the prescribed dose. This gives less coverage compare to plan2. All OAR doses have no sharp change compare to plan 2. Plan 3:95% of PTV getting 98% of dose. Bladder: 28% vol

[Downloaded free from http://www.jmp.org.in on Friday, July 29, 2011, IP: 130.14.254.25] || Click here to download free Android application for t

>4000cGy, 28%vol >4000cGy Plan 4:95 % getting 80 % of prescribed dose. Bladder: 0% vol>4000cGy, Rectum 0% vol >4000cGy, Rt and Lt femur: 3% vol>3000cGy Plan 5: 95% getting 99% of the prescribed dose (4500cGy) with mean dose of 4530 cGy, all other organs are getting higher dose.
**Conclusions:** The plan is made with some fixed gantry angles. Number and value of gantry angles creates optimization problems called beam angle optimization problems. The parameters used in the objective function decide the beam intensity I(x, y) at each point (x, y) on the MLC aperture called fluence map optimization. Once I (x, y) was calculated, one must convert them to leaf closing and opening, called leaf sequencing. When weight of OAR increases dose to OARs observed. By increasing power to OAR still dose can be restricted effectively but PTV coverage goes down. Similarly increasing weight and power to PTV, coverage to PTV increases but dose to OAR increases significantly. The optimizing parameters should be used such that the goal of radiation therapy can be achieved.

**P-16**
**Calibration of well chamber for low dose rate brachytherapy applications**
M Suresh, P Narayana Murthy*, AK Mahant. *Gutnur Cancer Care Centre, Acharya Nagarjuna University, Guntur, *RSS, RSSD, BARC, Mumbai, India*

**Introduction:** In the treatment of cancers, radioisotopes play vital role. For LDR brachytherapy treatment using Ir-192 and Cs-137 are most useful for most type of cancers. Before induction of these radioisotopes into patient, it should be calibrated with well chamber. A new well ionization chamber to be calibrated with standard laboratories. In this work, we calibrated our new well chamber with BARC

**P-17**
**Quality assurance tests with multileaf collimator for commissioning IMRT using sliding window technique**
SS Sivakumar, K Krishnamurthy, R Ravichandran, CA Davis, S Kannadhasan, JP Binu Kumar, Kamal El Ghamrawy. *Dept. of Radiation Oncology, National Oncology Centre, The Royal Hospital, Muscat, Sultanate of Oman*

**Introduction:** Intensity Modulated Radiotherapy (IMRT) using Multileaf Collimator can be delivered either using Step and Shoot technique or Sliding window technique. In Sliding window technique leaf pairs move dynamically with different speed while the beam is on. Accuracy in leaf positioning is crucial in IMRT. Thus prior to clinical commissioning of dynamic MLC related IMRT, performing quality assurance tests initially and periodically is essential. Varian 2300CD with 120 Millennium MLC is in clinical use for 3D CRT in our center since December 2004. DMLC position accuracy, dynamic leaf tolerance, reproducibility in standard patterns and dosimetric verifications are few recommended tests for IMRT implementation. The paper outlines the various mechanical and dosimetric quality assurance tests completed and their validation.
**Materials and Methods:** Stability of the leaf pairs is evaluated by Picket Fence and Garden Fence tests. Garden Fence test is performed with and without errors and also at four different gantry angles to evaluate the influence of gravitation on leaf pairs. Varian recommended standard patterns are generated using DMLC files. Intensity patterns are compared for leaf movement with and without beam interruptions. Kodak X Omat film is used to record the intensity patterns delivered by the dynamically moving leaf pairs. The film is placed at 100 cm SSD with 1.5 cm solid water phantom buildup material. The density patterns in films are evaluated using Omnipro IMRT software (M/s Scanditronix Wellhofer) and Vidar Scanner. Using Shaper software a sweeping field with gap width of 1cm is created and stability of the machine output at four different gantry angles is checked to verify gravity effect. Variation in the output for change in the gap width is also evaluated. Dosimetric leaf gap (DLG) separation and average leaf transmission factor is determined. Farmer Chamber FC65 (0.6 cc) with solid water phantom is used for ionization measurements along with Dose-1 electrometer.

**Results and Discussion:** Picket Fence and Garden Fence Tests show positional errors of leaf pairs with in acceptable limits. Errors introduced intentionally in Garden Fence test by editing leaf motion sequences confirmed their detectability of errors in individual leaf position as low as 0.05 cm. Intensity patterns with and without beam interruptions compared by visual inspection and using IMRT OmniPro software were acceptable. Ion chamber measurements show consistency in the machine output at all four different gantry angles. A change of 1.56% in the output is observed, which corresponds to a 0.2 mm gap width variation. Measured average leaf transmission is 1.96% and dosimetric leaf gap separation is 0.19 cm, which are in agreement with previous reports published for same machine.

**References**

1. Arnfield MR, Wu Q, Tong S, Mohan R. Dosimetric validation for multileaf collimator-based intensity-modulated radiotherapy: A review. Med Dosim 2001;26:179-88.
2. Van Esch A, Bohsung J, Sorvari P, Tenhunen M, Paiusco M, Iori M, *et al.* Acceptance tests and quality control (QC) procedures for the clinical implementation of intensity modulated radiotherapy (IMRT) using inverse planning and the sliding window technique: Experience from five radiotherapy departments. Radiother Oncol 2002;65:53-70.
3. DeMarco JJ, Solberg TD, Agazaryan N. A seed specific dose kernel method for low-energy brachytherapy dosimetry. J App Clin Med Phys 2003;4:66-74.
4. Venencia CD, Bese P. Commissioning and quality assurance for intensity modulated radiotherapy with dynamic multileaf collimator: Experience of the Pontificia Universidad Catolica de Chile. J App Clin Med Phys 2004;5:37-54.

This PDF is available for free download from a site hosted by Medknow Publications (www.medknow.com).

**P-18**
**Tissue equivalence of polymer gel dosimeters**
P Sellakumar, E James Jebaseelan Samuel*, Sanjay S Supe**. *Bangalore Institute of Oncology, Bangalore, *Vellore Institute of Technology, Vellore, **Kidwai Memorial Institute of Oncology, Bangalore, India*

**Introduction:** To evaluate the tissue equivalence of different polymer gel dosimeters in photon energy ranging from 10 keV to 20 MeV in terms of effective atomic number, mass attenuation co-efficients and energy absorption co-efficients.
**Materials and Methods:** For this study we chose 14 different types of gel dosimeters whose chemical compositions are given in table. The effective atomic number of each gel was calculated using the expression $Z_{eff} = w_i Z_i$, where $w_i$ and $Z_i$ are the fractional weight and atomic number of the $i^{th}$ element respectively. The mass- attenuation co-efficients (μ/r) and energy absorptions co-efficients ($\mu_{en}$/r) for each gel dosimeters were calculated using XCOM database for energy ranging from 10 keV to 20 MeV. The ratio of mass- attenuation co-efficients and energy absorptions co-efficients for each gel dosimeters to that of water were also calculated.
**Results:** The study showed that the effective atomic numbers of different gel dosimeters were in agreement with the effective atomic number of water with the variation of ±1.75%. The mass- attenuation co-efficients and energy absorptions co-efficients of different gel dosimeters were corresponded well with the values of water. Below 60 keV the coefficients relative to water were not in agreement with water (the maximum variation is 7.4%)
**Conclusion:** The study of tissue equivalence of polymer gel showed that the non-water equivalence of polymer gel below 60 keV which need appropriate corrections for accurate measurements.

**P-19**
**Effect of mobile phone on response of radiotherapy dosimeters**
RH Chilkulwar, Neeraj Dixit, SD Sharma, S Kannan. *Radiological Physics and Advisory Division, Bhabha Atomic Research Centre, CT and CRS Building, Anushaktinagar, Mumbai - 400 094, India*

**Introduction:** Workers of the radiotherapy (RT) department in the

[Downloaded free from http://www.jmp.org.in on Friday, July 29, 2011, IP: 130.14.254.25] || Click here to download free Android application for t

Table 1: Gel dosimeters and their chemical composition

| *Composition* | *BANG-1* | *BANG-2* | *PABIC* | *PAG* | *MAGIC* | *VIPAR* | *ABAGIC* | *PAGAT* | *HEAG* | *MAGAS* | *MAGAT* | *nMAG* | *nPAG* | *NIPAM* |
|---|---|---|---|---|---|---|---|---|---|---|---|---|---|---|
| Water | 0.89 | 0.88 | 0.8726 | 0.88 | 0.828 | 0.8723 | 0.825 | 0.89 | 0.89 | 0.83 | 0.83 | 0.86 | 0.87 | 0.89 |
| Gelatin | 0.05 | 0.05 | 0.049 | 0.06 | 0.08 | 0.0491 | 0.08 | 0.05 | 0.05 | 0.08 | 0.08 | 0.08 | 0.06 | 0.05 |
| BIS | 0.03 | 0.03 | 0.0392 | 0.03 | - | 0.0393 | 0.045 | 0.03 | 0.03 | - | - | - | 0.03 | 0.03 |
| Acrylamide | 0.03 | - | - | 0.03 | - | - | 0.045 | 0.03 | - | - | - | - | 0.03 | - |
| Acrylicacid | - | 0.03 | - | - | - | - | - | - | - | - | - | - | - | - |
| Ascorbic acid | - | - | - | - | 0.000352 | - | 0.00352 | - | - | 0.00880 | - | - | - | - |
| $CuSO_4.5H_2O$ | - | - | - | - | 0.00002 | - | 0.00002 | - | - | - | - | - | - | - |
| Hydroquinone | - | - | - | - | 0.002 | - | 0.002 | - | - | - | - | - | - | - |
| PEGDA | - | - | 0.0392 | - | - | - | - | - | - | - | - | - | - | - |
| N-Vinyl pyrolidone | - | - | - | - | - | 0.0393 | - | - | - | - | - | - | - | - |
| MAA | - | - | - | - | 0.09 | - | - | - | - | 0.09 | 0.09 | 0.06 | - | - |
| NaOH | - | 0.01 | - | - | - | - | - | - | - | - | - | - | - | - |
| THP | - | - | - | - | - | - | - | 0.01906 | - | - | 0.01906 | 0.0038 | 0.00953 | 0.01906 |
| NIPAM | - | - | - | - | - | - | - | - | - | - | - | - | - | 0.03 |
| HEA | - | - | - | - | - | - | - | - | 0.03 | - | - | - | - | - |
| $Z_{eff}$ | 7.413 | 7.454 | 7.404 | 7.405 | 7.374 | 7.391 | 7.362 | 7.631 | 7.418 | 7.372 | 7.596 | 7.441 | 7.589 | 7.624 |

hospitals are using mobile phone. Mainly two types of technology in mobile communication like GSM (Global System for Mobile communications) and CDMA (Code Division Multiple Access) are available. Most of the mobile phone operates at 800 and 1800 MHz GSM in the country. The handset emits electromagnetic energy when it is either receiving or making call. Mobile phone handsets are low-powered RF and microwave transreceiver emits maximum powers in the range of 0.2 to 0.6 watts. Although the microwaves emitted by mobile phones are of relatively low power, they are still capable of interfering with the operation of other sensitive electronic devices. This is an important issue during clinical dosimetry using therapy dosimeter.[1] We have experimentally determined the effect of mobile phones on the response of RT dosimeters. This paper presents the result of the measurements.

**Materials and Methods:** Measurements for interference of active mobile phones were carried out in four different dosimeters namely "Unidose" with 30 cc chamber, "Multidose (PTW Freiburg, Germany) with 30 cc chamber, SSD-92 (CD-High Tech, Bangalore, India) with 0.6 cc cylindrical chamber and Max-4000 (SI, USA) with HDR-1000 well chamber. A GSM mobile phone of model Nokia 1100 with MTNL Dolphin connectivity was used in this study. This mobile phone was capable to send and receive the data in addition to voice signals. Only voice communication mode was used in this study.[2] Firstly, the phone was placed in close proximity of the dosimeter system with both orientation i.e., antenna side and keypad side of the phone to the chamber and reading of the electrometer was recorded. Interference was observed by keeping the mobile phone in close proximity to ionization chamber, coaxial connecting cables and electrometer separately in all possible orientation of the phone. Each set of measurement was repeated for the distance of 5 cm and 1 meter between the center of the antenna of the phone and the center of the dosimetry system. The measurement was carried out by dialing local telephone line for about 1 min. Experimental efforts were also put to minimize the response of the dosimeter system due to interference by covering the system with RF shield.

**Results and Discussion:** Measured typical values of the CD SSD-92 dosimeter is shown in Table 1. Incidentally, ionization chambers do not contribute in the erroneous response of the system since no ionization is possible in the chamber due to RF and microwave radiation.

Table 1: Recorded display of CD SSD electrometer

| *Mobile phone orientation* | *Maximum measured value (MU/min) at* | | | | |
|---|---|---|---|---|---|
| | Front | Rear | Side-1 | Side-2 | Top |
| Antenna side | 7.2 | 10.3 | 1.0 | 1.3 | 13 |
| Keypad side | 3.0 | 9.0 | 1.7 | 0.9 | 9.0 |

The connecting coaxial cables are RF shielded thus did not contribute to the dosimeter response. PTW Unidose, Multidose and Max-4000 electrometers were found immune to electromagnetic interference. Secondary standard dosimeter manufactured by CD High-Tech indicated erroneous reading when exposed to RF and Microwave radiation emitted by the mobile phone. Experimentally it is observed that the erroneous response of the CD SSD electrometer got reduced to background when it is covered by metal box.

**Conclusion:** This study showed that mobile phones can introduce significant electromagnetic interference in RT dosimeters. The level of erroneous response can vary significantly among different models and also with time during call. The response is not decreasing with time because the electronics of the dosimeter system treat the RF and microwave signals from the mobile phones similar to the input signals from its chamber. Simple measures based on radiation-protection priciples i.e. time, distance and shielding can be taken to prevent this interference.

**References**

1. Djajaputra D, Nehru R, Bruch PM, Ayyangar KM, Raman NV, Enke C A. Cell-Phone interference with pocket dosimeter. Phys Med Biol 2005;50:N93-9.
2. Hietanen M, Shibakov V, Hallfors S. Safe use of mobile phones in hospitals. Health Phys 2000;79:S77-84.

**P-20**

**Intensity-modulated radiotherapy in carcinoma of the cervix: Dosimetric advantage over 3D conformal radiotherapy**

KR Muralidhar, Madhusudhan Sresty, Thirumalai Swamy, AK Raju, Subramanyam, Madhavi. *Indo American Cancer Institute and Research Center, Banjara Hills, Road No 14, Hyderabad - 300 034, India*
E-mail: krmuralidhar@rediffmail.com

**Introduction:** To compare intensity-modulated radiotherapy (IMRT) with Three-Dimensional Conformal Radiotherapy (3D-CRT) treatment plans in Carcinoma of the Cervix.

**Materials and Methods:** Five patients with different stages (IB, IIA, IIB, III and IV) were selected. 3D-CRT and IMRT plans (5040) cGy were made for each of them and compared with respect to Dose-Volume Histograms. Planning CT was acquired with the patient in the treatment position. All patients underwent contrast-enhanced CT with 3mm slice thickness. CT data sets were transferred to the treatment planning system (XIO, Computerized Medical Systems, Inc, St. Louis MO). Clinical target volume was contoured consisting of the upper vagina, parametria, uterus (if present), presacral and pelvic Lymphnode regions. The clinical target volume was expanded by 1 cm to create a Planning Target Volume (PTV). Using planning system, 5 field, 6MV, Coplanar IMRT plans were generated for the selected patients. The

This PDF is available for free download from a site hosted by Medknow Publications (www.medknow.com)

[Downloaded free from http://www.jmp.org.in on Friday, July 29, 2011, IP: 130.14.254.25] || Click here to download free Android application for t

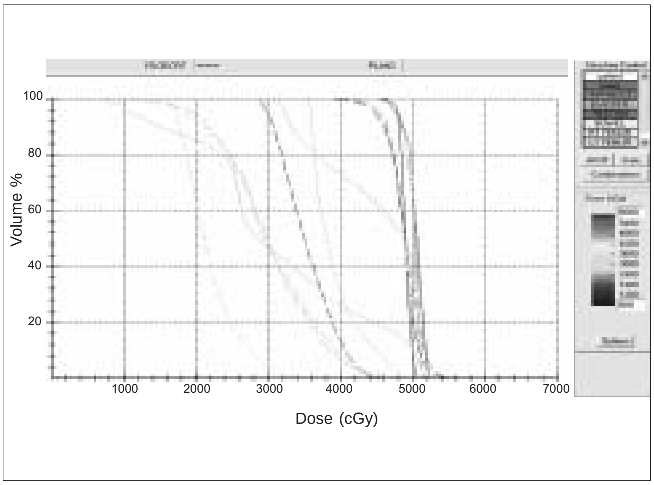

**Figure 1:** Dose-volume histogram: IMRT Vs 3DCRT

| | *Prescribed dose (cGy)* | *Volume (95%) dose below prescribed* | *Min. dose* | *Max. dose* |
|---|---|---|---|---|
| PTV | 5000 | 5% | 4570 | 5362 |
| | *Dose limit to dose limit (cGy)* | *Volume above* | *Min. dose* | *Max. dose* |
| Bladder | 4000 | 35% | 2605 | 4455 |
| Rectum | 4000 | 32% | 4058 | 4968 |

Treatment planning parameters prescribed dose to target volume and constraints of target and normal structures were defined. The IMRT fields were equal spaced at 72 degrees. The prescribed dose was 50 Gy in 25 fractions in all patients. Four field box techniques with 15MV were used for 3D-CRT. The dose-volume histograms were compared.

**Results:** In IMRT Mean dose of bladder reduced by 15.92%, Rectum reduced by 18.6%. Doses to Left Femur head and Right Femur head were also reduced by 35% and 36.12%. 95% volume of Nodal regions and PTV received the prescribed dose of 95%.

**Conclusions:** IMRT is useful in treating all stages of Carcinoma of the Cervix because of its dosimetric advantages. IMRT planning's result in excellent PTV coverage with considerable sparing of normal tissues.

**P-21**

**TLD intercomparision and quality assurance in the department of radiation oncology, MGIMS- Our experience**

N Balasubramanian, Rajeev Shrivastava. *Department of Radiation Oncology, Mahatma Gandhi Institute of Medical Sciences, Sevagram - 442 102, India*

**Introduction:** Accuracy of beam calibration is very important for the success of a radiation therapy treatment. The accuracy of dose delivery should be within ± 5%. Nowadays radiation oncologists insist the physicist community that the accuracy should be within ± 2% to achieve treatment goal. To meet this requirement, all cancer hospital should have a quality assurance programme of beam calibration. Their dosimetry system should be checked periodically and regularly calibrated. Periodic check source measurements are recommended to identify the characteristic performance (any drifts) of the dosimetry system. secondly, take the dosimetry system to the near by cancer hospital to check its sensitivity and performance. The calibrated beam output with the dosimetry system agrees with the clinical output of that hospital, then the dosimetry system works well. thirdly, participate voluntarily thermoluminescence dosimeters (TLD) postal-dose quality audit programme (TLD intercomparison) offered by iaea/who through radiation standard section of Bhabha Atomic Research Centre, Mumbai. The first two methods need considerable money, manpower and co-operation between two hospitals. But the third method is completely free. The evaluated results from TLD intercomparison give definitely boost our confidence in our dosimetry system. Effects have made in the department of radiation oncology, Mahatma Gandhi Institute of Medical Sciences (MGIMS) to participate regularly in this quality audit programme. The aim of this paper is to bring out integrity of our dose-measuring device and analyze the techniques used for the clinical purpose through the results of the TLD intercomparison at our department for last 09 years.

**Materials and Methods:** The accuracy of the dose delivery system depends on the dose measuring device i.e., dosimeter along with a valid calibration factor. In our department, we have dosimeter / electrometer comprises of electrometer model CNMC 11 and a 0.125CC PTW ionization chamber. Our dosimeter system is calibrated once in three years from radiation standard section, radiation safety systems division of BARC, Mumbai, which has been recognized secondary standard dosimetry laboratory (SSDL) by IAEA. In our department we follow trs-398 protocol for the calibration of beam output. Department of Radiation Oncology, MGIMS voluntarily participate in the IAEA/who TLD postal dose quality audit programme regularly. A set of three TLDS sent to us by BARC. we are requested to expose the two TLD capsules (during window period mentioned) to an absorbed dose to water of 2GY, established by measurements and calculations done by us in our regular day to day procedures used for delivering dose to tumor of the cancer patients. One capsule is a control one and should not be irradiated. The irradiated two capsule along with control capsule return back to BARC within a week for evaluation. This control capsules is kept against background, unexpected fading and accidental exposure. BARC collects the exposed TLD capsules from various centers in our country and forwarded to IAEA, dosimetry and medical radiation physics division, division of human health, vienna for evaluation.

**Results and Discussion:** IAEA evaluate the TLD capsules from various centers in our country and send the evaluated report to respective user hospital through BARC. the result helps us to identify our dose measuring system as well as human errors like calculational errors, dosimetric setup measurements etc. In 9 years, we had participated three times in the TLD intercomparison programmes, the percentage of deviation of dose estimated by either IAEA or BARC were 0.42, 1.78 and -0.2 for 1.25mev mean energy of co-60 photon beams in the years of 2000, 2002 and 2006 respectively. The cross calibration with IAEA/BARC and the difference (deviation) observed in our results were well within the limits say ± 2% rather than ± 5%, which are considered to be clinically acceptable. Conclusion: Our Dosimetry Quality Assurance Programme shows that the Dosimetry system set up at the Department of Radiation Oncology, MGIMS is adequately accurate. Through TLD intercomparison programme we have verified the beam output independently. Hence, this study helps not only to verify beam calibration output but also to provide a quality of radiotherapy treatment

Table 1: Shows the result of the percentage of deviation of our hospital from TLD intercomparison

| *Participated year* | *Teletherapy unit.* | *Intended/ quoted dose by mgims* | *Estimated dose/measured dose for two Tl dosimeters From Iaea/Who Or Barc.* | | *Mean value* | *% of deviation = 100 *(Qd-Ed)/Ed.* |
|---|---|---|---|---|---|---|
| 1999 | Theratron Phoenix Co-60 Machine | 2gy | 2.015 | 1.968 | 1.9915 | 0.42 |
| 2002 | Theratron Phoenix Co-60 Machine | 2gy | 1.97 | 1.96 | 1.965 | 1.78 |
| 2006 | Theratron Phoenix Co-60 Machine | 2gy | 2.01 | 2.00 | 2.005 | -0.24 |

This PDF is available for free download from a site hosted by Medknow Publications (www.medknow.com)

[Downloaded free from http://www.jmp.org.in on Friday, July 29, 2011, IP: 130.14.254.25] || Click here to download free Android application for t to needy cancer patients. Definitely one can improve the dose delivery to cancer patients, if concern authority implemented the following suggestions. Participating in this TLD postal dose quality audit programme should be mandatory instead of voluntary and the frequency of this postal dose quality audit should be increased considerably say once in a year.

**P-22**

**Goal achievement with implant brachytherapy as with intensity modulated radiotherapy**

NVN Madhusudhana Sresty, A Krishnam Raju, KR Muralidhar, S Tirumalaiswami, S Shiyama, Promod Dixit, Rajaneesh Kumar, PV Naidu. *Indo American Cancer Institute and Research Centre, Hyderabad, India*

**Introduction:** Currently, Intensity Modulated Radiotherapy is considered as the superior technique in external beam therapy planning to achieve very good uniformity, conformity and desired doses to the critical organs. Because of this advantage in many cases IMRT technique will be used to deliver boost doses. Implant Brachytherapy is being practiced in many centres since long time. With a good Brachytherapy implant, dose escalation is possible. In our Institute both techniques are in practice. The purpose of this study is to compare the differences in dose coverage, uniformity, conformity, patient average dose and doses to the critical organs in Implant Brachytherapy and Intensity Modulated Radiotherapy.

**Materials and Methods:** The institute is equipped with Varian Brachyvision treatment planning system, Siemens CT simulator and CMS XIO planning system.3 patients of ca cervix and 2 patients of soft tissue sarcoma treated with HDR Implant Brachytherapy had been studied. Post implant CT scans of 5 mm thickness were obtained.CT data had sent to both Brachyvision TPS for Brachytherapy planning and XIO planning system for IMRT planning. Target volume and other critical structures were drawn by clinician. Many plans obtained in both planning systems. In Brachyvision geometric and volume optimizations are available to achieve goal. Graphical optimization is used to reduce doses to critical structures and to increase the target coverage. Similarly external planning was done with IMRT technique by using convolution algorithm. In XIO planning system by changing the parameters rank, power and weight in IMRT prescription one can achieve the goal. DVH of target, bladder, rectum and patient were evaluated. Homogeneity and conformity indices also calculated for all plans.

**Results and Discussions:** Average homogeneity 0.285 in IMRT, 0.249 in Implant is almost equal. Patient mean dose 12.5% in IMRT, 12.9% in Implant is also comparable. Average Conformity is better in IMRT (1.28) compared with Implant (1.67). Dose coverage is good in implant than in IMRT. In Implant, on average, 95% volume is covered by 100% isodose line. But, in IMRT, on average,95% volume is covered by 95% isodose line only. There is an increment of up to 10% dose to critical structures in implant over IMRT on average. But, one can reduce the doses to critical structures easily by compromising the coverage of 100% isodose line in implant.

**Conclusion:** Homogeneity and patient mean dose of implant is comparable to IMRT. Dose coverage is good in implant and conformity is better in IMRT. Though there is an increment of dose to the critical structures in Implant, still they are within acceptable limits and also they can easily be reduced by compromising the target coverage dose from 100% to 95% or 90%. So, by calculating LDR equivalent to HDR dose per fraction, total dose can be delivered with very less number of fractions in Implant therapy. IMRT is more complex and expensive. Biological evidence of IMRT is still questionable! So, for boost doses, practice of Implant therapy is effective, cheaper and reliable.

**P-23**

**Entrance and exit dose measurements by using FBX dosimetry in head and neck cancer patients to measure radiological depth of the tumour**

A Balraj, MK Semwal*, N Chacravarty*, PK Thakur, S Bhatnagar, PB Vidyasagar**. *Dept of Radiation Oncology, Malignant Diseases Treatment Centre, Command Hospital (SC) Pune - 411 040, *Dept of Radiation Oncology, Army Hospital (R and R) Delhi Cantt - 110 011, **Dept of Physics, University of Pune, Pune - 411 007, India*

**Introduction:** The prescribed dose delivery to the tumour depends upon various factors. One of the important factors is depth of the tumour from the skin of the patient. The treatment depth in head and neck cancer is always uncertain due to the anatomy of that region. The FBX aqueous chemical dosimetry system has been found useful for depth dose measurements in external beam therapy. Therefore, FBX solution prepared in-house was used to measure the entrance and exit dose by *in-vitro* and *in-vivo* method thereby measuring the radiological depth of the tumour, which varies from physical depth. Using this radiological depth, tumour dose can be delivered more accurately. In head and neck cancer patient treatment setup, the depth of the tumour is measured from skin to the midline of the body which is maximum at the inferior border and minimum at the centre and in-between at the superior border of the field. Skin separation is always taken to measure the depth assuming that skin to skin is occupied by tissue. But in reality, air gaps are present in mouth and trachea. The variation of dose distribution in air and tissue, may lead to treatment time calculations error, hence leading inaccurate dose delivery. The aim of this study is to measure the radiological separation of the treatment field by measuring entrance and exit dose with help of FBX Dosimetry and thereby delivering prescribed dose to the tumour.

**Materials and Methods:** FBX solution was prepared by standard methods as per the literature (Gupta *et al* 1982) by using Ferrous ammonium sulphate (0.20 mM) Benzoic acid (5.0 mM) Sulphuric acid (0.08N) and Xylenol orange (0.20 mM). Stoppard cylindrical polypropylene tube of 5.0 cm length, 1.3 cm outer diameter amd 0.1 cm wall thickness were filled with fresh FBX solution and used as dosimeters both *in-vitro* and *in-vivo*.

***In-vitro* Setup:** Theratron 780-E Co-60, machine beam out put was measured at 10 cm depth of water for various fields' size (actual patient treatment field size) with 80 cm SSD by using 0.6cc farmer type ion chamber and CNMC model II secondary standard dosimeter. From this the output at 0.5 cm depth (D max) was calculated by using PDD values. Now the FBX filled vials were irradiated (for dose of D max) for various fields size (actual patient treatment field size) keeping the vials from outside on the side surface of the water phantom. The optical densities (OD) of the irradiated vials were measured using Photochem micro 5 filter colorimeter at 540 nm. The dose (D Max) versus the OD thus obtained in this geometry was used to determine the entrance dose, i.e., dose at D max. The calibration curve obtained was used to measure the entrance dose with the patient setup. The same principle was used for the exit dose measurements. Here the vial and the ion chamber were kept at the bottom of the water phantom with different depth of water. The vials were irradiated with different field size. Both OD and ion chamber readings were plotted and calibration curve obtained was used for measuring exit dose in patient setup.

***In-vivo* Setup:** All head and neck cancer patients were treated with parallel opposing fields. Thermoplastic mould was used for all patients for better immobilization during treatment. For each patient six vials filled with FBX solution were placed at the entrance and exit beam points of superior, inferior borders and in the centre point of the field. The vials were placed on the thermoplastic mould and the patients were treated as per the treatment dose schedule. After the treatment the vials were removed and OD was measured. With already available in-vitro calibration curves the entrance and exit dose were obtained.

**Results and Discussions:** This study has been done on 30 head and neck cancer patients. It was found that the entrance dose for both in-vitro and *in-vivo* varies with percentage of 2.2%, 4.3% and 5.8% in centre of the field, superior border and inferior border respectively. Where as the exit dose for both in-vitro and *in-vivo* varies greater then the entrance dose variation in all points. The greater variation in the exit dose clearly shows the air gap present in the intra field. With entrance and exit dose the radiological separation was calculated which is smaller than the physical separation. Dose delivery as per radiological separation may be more accurate than the dose delivered due to

This PDF is available for free download from a site hosted by Medknow Publications (www.medknow.com).

[Downloaded free from http://www.jmp.org.in on Friday, July 29, 2011, IP: 130.14.254.25] || Click here to download free Android application for t physical separation.

### P-24

### Monte Carlo aided dosimetry of caesium-137 intracvaitary brachytherapy source

K Devan, D Manigandan, G Bharanidharan, S Gopi*, KV Subbaiah**, P Aruna, S Ganesan. *Division of Medical Physics and lasers, Department of Physics, Anna University, Chennai - 600 025, *BIBI General Hospital and Cancer Center, Hyderabad - 500 024, **Safety Research Institute, Indira Gandhi Center for Atomic Research, Department of Atomic Energy, Kalpakkam - 603 102, India*
E-mail: kdevn@yahoo.co.in

**Introduction:** Among several treatment modalities for the cervix cancer, Intracavitary brachytherapy is being considered as one of the most frequently used treatment modality with Cesium 137 and other radioactive isotopes. Various models of Cesium 137 source designs and models are available in market. In India, the Cesium 137 source designed by Board of Radiation and Isotope Technology (BRIT) supplied by Bombay Atomic Research Center (BARC), Bombay is being used in various cancer hospitals for the treatment of the later. The kit contains long intrauterine tandem (3 sources), medium intrauterine tandem (2 sources) and vaginal ovoids (2 sources). The purpose of this work is to estimate the dose distribution for the BRIT source using Monte Carlo Simulation (MCNP4B) and to compare it with Quimby's dose distribution. Also to compare the dose delivered to patients.

**Materials and Methods:** The Cesium 137 source which is available in BIBI cancer Hospital, Hyderabad was used in this study. It is 1.5 cm active length, total length 2.1 cm and external diameter 0.3 cm. The sheathing thickness is 0.05 cm of stainless steel. The air-kerma rate constant for Cesium 137 is taken as 0.078uGym$^2$Bq$^{-1}$h$^{-1}$ (exposure rate constant of 3.32 Rcm$^2$mCi$^{-1}$h$^{-1}$). The activities of the sources in long tandem are 31.1899, 23.993 and 20.394 mgRaEq. The activities of the sources in ovoids are 20.394 mgRaEq each. Computation of dose rate was done using the Monte Carlo N-Particle transport code System (MCNP) version 4B computer program. MCNP4B is a general-purpose code for calculating the time-dependant, continuous-energy transport of neutrons, photons and/or electrons in three-dimensional geometry. Point detectors (F5 tally) and ring detectors (F5a tally) were used to compute flux at various points of interest, where 'a' can be either X or Y or Z-axis, the coordinates of the detector location. Each photon history originates at a random position and direction inside the source core. The simulations were done for 10$^6$ photon histories to obtain statistically acceptable results. Each photon history was traced down to 1 keV, a default cutoff energy set by MCNP.

**Results and Discussion:** For a particular patient case, loaded with long tandem with ovoids, the dose rate calculated by Monte Carlo at left Point-A is 8.369 and right Point-A is 81.12 cGy/hr when compared to a value of 102.46 and 91.50 cGy/hr respectively. The average rectal dose estimated using Monte Carlo is 87.79 cGy/hr and average bladder dose is 143.41 cGy/hr using Monte Carlo simulation when compared to a value of 99.08 cGy/hr and 173.44 cGy/hr respectively. It is found that the dose rate values calculated by manual method are higher than that are calculated by Monte Carlo simulation. It is also found that the manual method also over estimates the rectal and bladder dose. From this study, it is clear that the patients receive unnecessary over dose.

**Conclusion:** The study was done for all intracavitary brachytherapy applications such as long tandem with ovoids, medium tandem with ovoids, long tandem only, medium tandem only and ovoids only. Five patient details for each case were studied. Totally 25 cases were investigated and found that in all the cases, the dose rate calculated by manual method over estimated the dose rate when compared to the Monte Carlo computed values of dose rate. Thus it is found that all the patients treated by manual calculation method were subjected to less treatment time because of under estimated dose rate, actually the treatment time should be large in order to compensate the less dose rate value calculated by Monte Carlo method.

### P-25

### Partial wedge field dosimetry for indigenously developed mini phantom and water phantom

S Senthilkumar, N Sivagami Sundari*, EH Annex*, S Vasanthamalai, S Jebasingh. *Dept. of Radiotherapy, Govt Rajaji Hospital and Madurai Medical College, Madurai - 20, *Dept. of Basic Sciences, A. K. College of Engineering, Krishnankovil, India*

**Introduction:** Goal of Radiotherapy is to treat the cancer patient with homogenous photon beam. When the patient's body surface is uniform then the photon beam will be attenuated uniformly. If the

This PDF is available for free download from a site hosted by Medknow Publications (www.medknow.com)

Table 1: Dose rate in Rad/mg h (Quimby's table) for BRIT $^{137}$Cs source

| | Y=0.5 | 0.75 | 1 | 1.5 | 2 | 2.5 | 3 | 4 | 5 |
|---|---|---|---|---|---|---|---|---|---|
| Z=0 | 20.4 | 10.9 | 6.69 | 3.27 | 1.89 | 1.23 | 0.86 | 0.49 | 0.31 |
| 0.5 | 16.9 | 9.3 | 5.95 | 3.03 | 1.79 | 1.18 | 0.84 | 0.48 | 0.31 |
| 1 | 8.06 | 5.64 | 4.11 | 2.41 | 1.56 | 1.07 | 0.77 | 0.46 | 0.3 |
| 1.5 | 3.25 | 2.92 | 2.48 | 1.75 | 1.25 | 0.92 | 0.7 | 0.43 | 0.29 |
| 2 | 1.62 | 1.62 | 1.54 | 1.23 | 0.97 | 0.75 | 0.6 | 0.39 | 0.27 |
| 2.5 | 0.9 | 0.99 | 0.99 | 0.88 | 0.74 | 0.62 | 0.51 | 0.35 | 0.25 |
| 3 | 0.56 | 0.66 | 0.68 | 0.64 | 0.57 | 0.5 | 0.42 | 0.31 | 0.22 |
| 3.5 | 0.37 | 0.44 | 0.48 | 0.48 | 0.45 | 0.4 | 0.36 | 0.28 | 0.21 |
| 4 | 0.24 | 0.32 | 0.35 | 0.36 | 0.36 | 0.33 | 0.29 | 0.24 | 0.19 |
| 4.5 | 0.15 | 0.24 | 0.26 | 0.29 | 0.28 | 0.27 | 0.25 | 0.21 | 0.17 |

Table 2: Dose rate in Rad/mg h (Monte Carlo MCNP4B) for BRIT $^{137}$Cs source

| | Y=0.5 | 0.75 | 1 | 1.5 | 2 | 2.5 | 3 | 4 | 5 |
|---|---|---|---|---|---|---|---|---|---|
| Z=0 | 17.61 | 9.36 | 5.73 | 2.74 | 1.59 | 1.02 | 0.71 | 0.40 | 0.25 |
| 0.5 | 14.81 | 8.04 | 5.06 | 2.54 | 1.50 | 0.99 | 0.69 | 0.39 | 0.25 |
| 1 | 7.24 | 4.96 | 3.55 | 2.05 | 1.31 | 0.89 | 0.64 | 0.38 | 0.24 |
| 1.5 | 3.10 | 2.66 | 2.21 | 1.51 | 1.06 | 0.77 | 0.58 | 0.35 | 0.23 |
| 2 | 1.62 | 1.53 | 1.39 | 1.08 | 0.83 | 0.64 | 0.50 | 0.32 | 0.22 |
| 2.5 | 0.98 | 0.97 | 0.92 | 0.78 | 0.64 | 0.52 | 0.43 | 0.29 | 0.20 |
| 3 | 0.65 | 0.66 | 0.64 | 0.58 | 0.50 | 0.42 | 0.36 | 0.25 | 0.18 |
| 3.5 | 0.47 | 0.48 | 0.47 | 0.44 | 0.39 | 0.35 | 0.30 | 0.22 | 0.17 |
| 4 | 0.35 | 0.36 | 0.36 | 0.34 | 0.32 | 0.28 | 0.25 | 0.20 | 0.15 |
| 4.5 | 0.27 | 0.28 | 0.28 | 0.27 | 0.26 | 0.24 | 0.21 | 0.17 | 0.13 |

[Downloaded free from http://www.jmp.org.in on Friday, July 29, 2011, IP: 130.14.254.25] || Click here to download free Android application for t

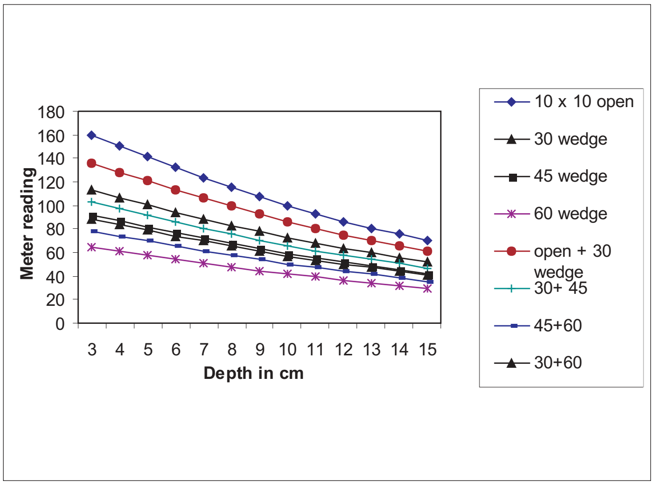

**Graph 1:** Mini - Phantom measurement for combined wedge effect

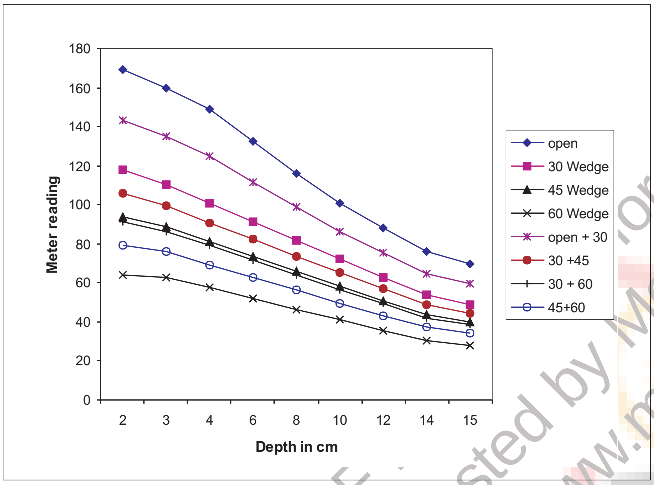

**Graph 2:** Water phantom measurement for combined wedge effect beam travels through non-uniform or irregular surface of the human body the radiation beam is also attenuated non-uniformly and tumor volume will get non-uniform dose. To deliver uniform dose to the tumor, surface of the patient's body must be uniform. If the body surface of the patient is non-uniform, it is cumbersome to irradiate the tumor with uniform dose. Patient's body is always oval or circular in shape. If the irradiated body is square or rectangular shape like a water phantom, we can deliver maximum dose to the tumor volume. In cobalt -60 photon beam isodose plot is flat in shape. If irradiated with Co-60 beam, tumor will get non-uniform dose, some areas will get hot spot and some areas will get cold spot. To avoid these cold and hot spots within the tumor volume, modification of the Co-60 beam is a mandatory one to modify the photon beam. Different types of beam modifying devices are used in the radiotherapy department such as wedge filter, tissue compensator, beam flattening device and filters, etc. All radiotherapy departments have pre angled wedges like 15°, 30°, 45° and 60°. In our hospital we have 30°, 45° and 60° wedges for different field sizes (6x15, 8x15 and 10x15). These wedges will not satisfy the 100% uniform dose to the tumor volume. When we planned for radiotherapy using above wedges, it is difficult to irradiate with maximum dose to the tumor volume for all patients unless if we have all angled wedges. In this work we studied different combination of wedges to get the maximum dose to the tumor volume and verified the dosimetry date using water phantom measurement and indigenously developed PMMA Mini phantom measurements and also with isodose chart method for all combination of wedges.

**Materials and Methods:** Mini Phantom made up of PMMA material and cylindrical in shape with 4 cm diameter. It has the provision to insert the 0.6cc ion chamber inside the mini phantom and also it has base plate, stand and phantom holder. The Mini phantom set up placed over the cobalt machine couch in vertical position. Central axis of the photon beam made to coincide with the axis of the mini phantom and also center to the ion chamber with the help of laser alignment. Collimator opened for the field size of 10 cm x 10 cm and SSD set at 80 cm to the center of the chamber. Machine exposed for 1 minute and meter readings were noted then 1 cm slice of the PMMA material was placed over the mini phantom and then machine energized for 1 minute and meter readings were noted. The same setup was repeated for 15 cm slice of the PMMA material to measure the depth dose up to 15 cm depth. Then the pre angled 30° wedge filter was inserted in the path of the cobalt-60 beam without changing the machine setup and also same procedure was repeated for 45°, 60° Wedge filters up to 15 cm depth. To get the intermediate wedge effect we used combination of different wedge angels for the same treatment condition. First open beam irradiated the chamber for half a minute without changing the machine setup and electrometer reading then 30° wedge filter was inserted in the path of the beam then the machine exposed for half a minute and the meter readings were noted and tabulated. Same procedure was repeated for different wedge combinations like 30°+45°, 30°+60°, 45°+60°, etc. For water phantom dosimetry measurement the above set of mini phantom experiment repeated for open and all wedge fields.

**Result and Discussion:** The dosimetry data for open field and different combination of wedge values were tabulated and plotted in graph and all the graph lines are linearly decreasing from higher level to lower level when the depth is increasing. Graph also shows decreasing due to more attenuation of the radiation in the phantom material. Graph 1, 2 clearly shows the 45° wedge values are similar to the combination 30° + 60° wedge values. From this we can easily understand that the partial wedges give the same effect of the pre angled wedge effects. Open and 30° wedge gives 15° wedge effect and 30° + 45° wedge gives the effect of 37.5° wedge effect and the 30° + 60° gives the effect of 45° wedge effect for both the mini phantom and water phantom.

**Conclusion:** Goal of Radiotherapy is to deliver maximum uniform dose to the tumor volume. For that we used the available wedges and used the concept of partial wedges to get the maximum dose in different combination of wedges. Dosimetry done for different wedge combination and the dose calculated using the TRS 398 water phantom and indigenously fabricated mini phantom to verify the dose for different depth and different field size. Both the water phantom and mini phantom values are with in + 2. Combination effect of the wedge values also lies with in the tolerance for both the mini phantom and water phantom measurement and also for different field size and different depths.

### P-26

### Calibration of beam data parameters using indigenously developed mini phantom values compared with ESTRO mini phantom values

S Senthilkumar, EH Annex*, N Sivagami Sundari*, S Vasanthamalai, J Jebasingh. *Dept of Radiotherapy, Govt Rajaji Hospital and Madurai Medical College, Madurai - 20, *Dept. of Basic Sciences, A. K. College of Engineering, Krishnankovil, India*

**Introduction:** Accurate dosimetry involves a large number of steps, starting with the calibration of a dosimeter through the determination of the absorbed dose at a point of interest in the patient. Mini-phantoms are playing an important role to measure the basic scatter parameters of high-energy photon beams. The ESTRO published in 1997 the in air measurement in high energy photon beams using mini-phantoms for the measurement of head scatter components and volume scatter, output ratios, wedge factors, phantom scatter correction factors ($S_p$), total scatter correction factors ($S_{C,P}$), collimator scatter correction factors($S_{--C}$) and wedge factors etc. The shape of the mini-phantom is square or circular with the cross section perpendicular to its axis and it is made by PMMA or polystyrene a water equivalent. In this present work we fabricated an indigenous mini-phantom which is low cost,

This PDF is available for free download from a site hosted by Medknow Publications (www.medknow.com)

[Downloaded free from http://www.jmp.org.in on Friday, July 29, 2011, IP: 130.14.254.25] || Click here to download free Android application for t easy to handle. Mini-phantoms are fabricated with two different water equivalent material 1) PMMA 2) Bee's wax and also in different shapes for both material (with top flat surface and dome surface). For measuring different depth dose, different SSD, we fabricated mini-phantom with slices of 1 cm thick and same diameter of the mini-phantom to analyze the beam parameters of Co-60 beam for different depth and different field sizes and also open and wedge fields. Finally these values were compared with the ESTRO Booklet No: 6 published values.

**Materials and Methods:** Different types of build-up cap are used to measure the photon beam parameters; the basic columnar Mini-Phantom is cylindrical in shape with 40 mm diameter and 250 mm in length. Different Mini phantoms are fabricated with different material (PMMA and Bees wax) and different surface (flat and dome) with 15 slices also fabricated for 10mm thickness for depth dose measurement. Mini phantoms are kept in vertical direction and irradiated parallel to its long axis. The ion chamber is placed 10 cm below the surface of the mini phantom. The photon beam travels through the long axis of the columnar Mini Phantom for 10 cm depth. So it is deep enough to stop all the contaminating electrons in the provided buildup depth. Long axis of the Mini Phantom has the smallest cross section with respect to the photon beam and the ion chamber is placed along the cylindrical axis of the columnar Mini Phantom. Ion chamber has the diameter of 0.7 cm and 1.15 cm wall thickness and the columnar Mini Phantom has a diameter of 4.0 cm. Minimum field size of our cobalt 60 machine is 5 X 5 $cm^2$, so the small size Mini Phantom will be irradiated fully to measure head scatter correctly. When measuring Head-scatter ($S_h$) factor using a Mini Phantom, any large objects near the Mini Phantom would produce secondary scatter radiation, so that should be kept away from the mini phantom. That's why the Mini Phantom and its accessories are fabricated with PMMA material. 0.6cc cylindrical ion chamber used as a detector and it is inserted in the Mini Phantom then both are holed in a mini phantom holder. Mini Phantom long axis is aligned parallel to the central axis of the photon with the help of laser alignment of the cobalt 60 room. The center of the 0.6cc ion chamber active volume is set at a (SAD) source-axis-distance, usually 80cm for cobalt-60 machine.

Water phantom 30x30x30 $cm^3$ is used for the $O_R$ measurement under the full scatter condition. 0.6cc ionization chamber is placed in the water phantom at the central axis of the photon beam at the reference depth of $Z_R$=10 cm. The source to detector distance is kept at 80 cm distance. i.e., SAD = 80 cm, The ionization chamber exposed half an hour for warm-up and chamber readings are corrected for stem effect and cable effect. Collimators X, Y are set for minimum Equivalent Square field 5x5 $cm^2$ and exposed for a minute, meter readings are noted. The same procedure is repeated for all the equivalent square fields up to 35x35 $cm^2$. To measure $O_R$ for rectangular field measurement the X collimator jaws fixed at 5 cm and Y jaws moved from 5 cm to 35 cm then the Y jaws fixed at 5 cm and X jaws moved from 5 cm to 35 cm. A 25 cm long 40 mm diameter mini phantom with ion chamber inserting hole of diameter 15 mm and 170 mm long is used for $O_O$ measurement. Ion chamber is placed in side the phantom parallel to the central axis of the beam and mini phantom placed at the reference depth of 10 cm ($Z_R$). The source to detector distance is kept at 80 cm. The meter readings are corrected for stem effect and cable effect. In case of mini phantom these quantities are minimum due to its orientation in the beam. Collimators X and Y are set for minimum equivalent square field of 5x5 $cm^2$ and exposed for a minute then meter readings are noted. Same procedure is repeated for all the equivalent square fields up to 35x35 $cm^2$. To measure the Oo of rectangular field, the X collimator jaws fixed at 5 cm and Y jaws are moved from 5 cm to 35 cm then the Y jaws fixed at 5 cm and X jaws moved from 5 cm to 35 cm. Oo is an asymmetric function of X and Y collimator settings. Phantom scatter ratio (Sp) measured the dose values of ratio between the full scatter condition in water phantom and in air measurement in a mini phantom.

**ESTRO Booklet No:** 6 explain the phantom scatter ratio for monitor unit calculations and the ratio between water phantom and mini phantom. Values of ' Sp ' differences will cancel out each other. The ' Sp ' value measurement for square fields carried out in the Co-60 photon beam from the field size 5x5 $cm^2$ to 35x35 $cm^2$ at SSD of 80 cm. The ionization chamber is placed at the reference depth of 10 cm for both the phantom setup. To calculate the monitor unit in a wedged photon beam is based on the use of correction factor called the wedge factor, wedge filters should be introduced in to the central axis of the beam under the reference conditions and open beam for the same number of monitor units. Water phantom is placed over the treatment couch under the reference conditions (depth $Z_R$, field size $C_R$ and source to chamber distance SCD). In our hospital we have 30°, 45° and 60° wedges. First open beam reading are taken for one minute then the 30° wedge introduced and exposed for the same. The same setup is repeated for 45° and 60° wedges for different field sizes also for mini phantom then the values were tabulated. Collimator exchange effect measurements were carried out in the large water phantom and mini phantom with different wedge factor under the reference condition. X-jaws of collimator fixed and the Y-jaws were moved from minimum to maximum field and meter readings were noted then the Y-jaws fixed and X-jaws were moved from minimum to maximum field then the values were tabulated.

**Result and Discussion:** Theratron phoenix Co-60 machine output ratio ($O_R$) measurement carried out in a water phantom under the full scatter condition. Readings include head scatter and phantom scatter variations for all square field sizes (C) and the reference field size ($C_R$) and these values are compared with the ESTRO Booklet No:6 published data for MDS Co-60 Theratron 780 values. Both the values are almost same in the smaller square field and the output ratio ($O_R$) increases with increase in field size. When the field size is increasing water phantom scatter contribution also increase, so the beam quality is decreasing. Variation observed in the larger filed size. In MDS Theratron 780 having more scatter contribution than the Theratron Phoenix machine. The output ratio $O_O$ measured in indigenously fabricated phantoms made of PMMA and Bees Wax material. These values are varied as the field size increases. Our mini phantom values are slightly higher than the MDS Co-60 Theratron 780 values in the higher field size. Normalized output ratio of our mini phantom values are compared with ESTRO Booklet 6 published data for MDS Co-60 Theratron 780 mini phantom values. PMMA and Bees Wax mini phantom gives the same output ratio with small variation observed in higher field size. The deviation between the two mini phantom values are less than 0.1% and also compared with the ESTRO Booklet 6 published data for MDS Co-60 Theratron 780 mini phantom values also less than 0.1%. CEE (Collimator Exchange Effect) of the theratron phoenix machine variation was observed and it was almost same for all fields except in the large field size. It is almost negligible in the Co-60 unit. This small variation is due to the collimator distance from the source, X jaws are near to the source than the Y jaws. Both $O_O$ and $O_R$ depends upon the orientation of the collimator for rectangular beams. Both the X-Jaws and Y-Jaws curves are superimposed up to 20x20 $cm^2$ field size. The deviation observed in the larger field size. It is similar to water phantom data. Our Co-60 Machine has almost negligible Collimator Exchange Effect. The variation of the wedge factor with different field size gives the same result using mini phantom and large water phantom. The variation with field size of the relative wedge factor can be obtained by the ratio of output for open beam and wedged beam for a normalized field size of 10x10 cm. Water phantom and Mini phantom are reflecting the same wedge factor. In Mini phantom dome surface and flat surface doesn't show any considerable change in the measurements at the same time the normalized value remain same for different water equivalent materials (PMMA and Bees Wax). Phantom scatter correction factor ($S_P$) are calculated from the water phantom and mini phantom output ratios $O_R$ and $O_O$ for a Co-60 beam for each field sizes are tabulated. Phantom scatter correction factor ($S_P$) varies due to the beam quality with field size. Our calculated values are compared with Storch and Van Gasteren's ' $S_P$' values also compared with ESTRO booklet No:6 $S_P$ values. The results were tabulated and our experimental $S_P$ values lies +0.2% of existing values.

### P-27

**Patient specific quality assurance for the delivery of steriotactic radiosurgery and steriotactic radiotherapy**

KR Muralidhar, P Narayana Murthy, Pramod Kumar Dixit,

This PDF is available for free download from a site hosted by Medknow Publications (www.medknow.com)

[Downloaded free from http://www.jmp.org.in on Friday, July 29, 2011, IP: 130.14.254.25] || Click here to download free Android application for t

Tirumalai Swamy, Madhusudhan Sresty, Shiyama, Rajanesh.
*Indoamerican Cancer Institute and Research Center, Road No 14, Banjara Hills, Hyderabad - 500 034, India*
E-mail: krmuralidhar@rediffmail.com

**Introduction:** A patient specific quality assurance program has been developed to facilitate the clinical implementation of Steriotactic Radiosurgery and Steriotactic Radiotherapy.

**Materials and Methods:** The measurements presented have been conducted with the use of the Siemens, Primus linear accelerator. This linear accelerator generating a 6 MV X-ray beam with dose rate of 200mu/min.The micro-Multileaf collimator is an accessory for the Siemens linear accelerator. The micro-MLC consists of 96 tungsten leaves that shape a treatment field upto 10x10 cm. In this 14 inner leaf pairs having thickness of 2.1 mm and outer leaf pairs having thickness of 3.6 mm. It is four bank mMLC with leaf thickness of 3.8 cm each. Maximum distance over center line is 2 cm. Maximum leaf travel distance is 8.8 cm. Steriotactic Radiosurgery and Steriotactic Radiotherapy plans presented have been generated using commercially available 3D planning system (Accusoft from Direx Medical Systems, UK). Measurements have been conducted with a MED-TEC Benchmark IMRT Phantom, LA. USA. Steriotactic Radiosurgery / Steriotactic radiotherapy Patient specific QA requires a method of absolute dose measurements. This Methodology include dosimetric task that is performed prior to the treatment of each patient. The ionization chamber is the standard dosimeter for calibration and absolute dose measurements for radiation therapy. A 0.1cc ionization chamber (scanditronix wellhofer) has been used for absolute dose measurements in the MED-TEC IMRT Phantom. The MED-TEC IMRT phantom is computed tomography (CT) compatible with visible fiducial markers. The phantom was scanned on Siemens CT scanner and the images were transferred to treatment planning system. Treatment planning system has an option of mapping the complete patient treatment on to any phantom. Adjustments of the leaf positions can be made with leaf position values if needed. After mapping the patient treatment plan onto the MED-TEC Phantom, the dose distribution in the phantom can be viewed. The point dose can be measured at the center of the target (isocenter). The planned treatment plan on phantom then transferred to work station (acculeaf). X, Y, Z co-ordinates from the planning system was used to keep the center of the chamber at the isocenter. In individual field measurements all fields are individually delivered and measured in the MED-TEC IMRT phantom with an SAD setup shown in Figure 1, at 10 cm depth and 0 degree gantry angle. For composite field measurements the composite plan is delivered on to the MED-TEC IMRT phantom. Absolute dosimetry for each composite plan is performed using an ionization chamber, with ion chamber positioned at isocenter. The dose calculated in TPS then compared with measured [Tables 1 and 2].

Table 1: Percentage of difference between measured and TPS calculated data in individual field measurements

| Field no. | Calculated dose (cGy) | Measured dose (cGy) | % difference |
|---|---|---|---|
| 1 | 40 | 39 | 2.5 |
| 2 | 40 | 38.9 | 2.75 |
| 3 | 40 | 39.2 | 2 |
| 4 | 40 | 39.7 | 0.75 |
| 5 | 40 | 39.2 | 0.8 |
| Total | 200cGy | 196 | 2 |

Table 2: Percentage of difference between measured and TPS calculated data in composite field measurements

| Patient No. | Calculated dose (cGy) | Measured dose (cGy) | % difference |
|---|---|---|---|
| 1 | 200 | 194 | 3 |
| 2 | 200 | 195 | 2.5 |
| 3 | 200 | 198 | 1 |
| 4 | 200 | 194 | 3 |
| 5 | 200 | 196 | 2 |

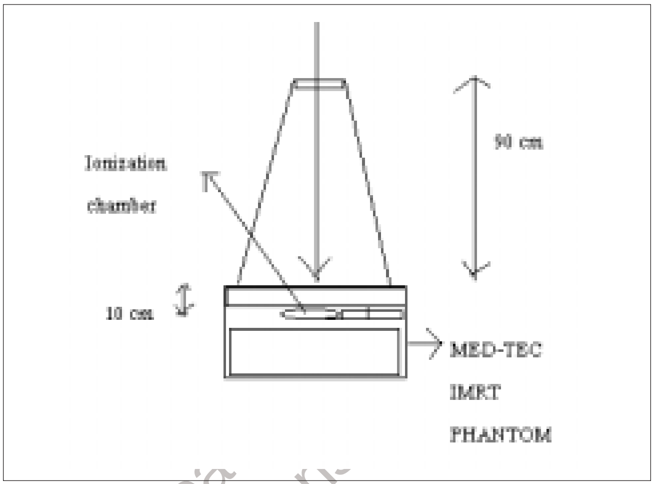

**Figure 1:** Experimental setup for single field SRS/SRT measurement

**Results and conclusions:** Measured and calculated values are compared in the Individual field measurements and in the composite field measurements. Dose differences between measured and calculated were obtained. Excellent agreement between the measured and calculated values has been observed. 2.75% variation between measured and calculated values was observed for Individual field measurements and less than 3% variation was observed for composite field measurements.

### P-28

### Mega voltage cone beam CT for IGRT: A preliminary report

VK Sathiya Narayanan, Kumaraswamy, Shrikant Deshpande, Sumit Basu, Janhavi Bhangle, Vikram Maiya, Nirmal Kumar Babu.
*Department of Radiation Oncology, Ruby Hall Clinic, 40-Sassoon Road, Pune - 411 001, India*

**Introduction:** To commission the Mega Voltage Cone Beam CT by performing acceptance test procedure and to measure the actual dose delivered and also to use the images for patient alignment to deliver the treatment.

**Materials and Methods:** An Oncor impression plus linear accelerator with its 6 MV beam and the 1024 x 1024 amorphous silicon flat panel (AG9) are basically used for this purpose. The 6MV beam is used with the shorter dose pulse which will be 6-10 times of the usual one with 250 pulses per second. The gantry is made to make an arc of 200° degree from 270° to 110°. The flat panel acquires an image at each degree of the gantry rotation and all the 200 images are used to reconstruct the 3D volumetric image. The images produced are co-registered or fused with the planning CT. This acquisition and the registration are done at the therapist workspace. The planning CT images with all the target volumes and critical structures are imported in the therapist workspace earlier and once the megavoltage image is acquired, the co-registration takes place with the maximization of the mutual information algorithm (MMI) and from this the table shift to be done is calculated. The alignment and the resulting table shift can also be calculated by manually aligning the images. The images are available in axial, sagittal and coronal views for this purpose. The total time of acquiring, comparing and aligning with the planning CT images is below 5 minutes. The protocol we used in this situation was with 8 MU in the 200° arc and the dose delivered to the patient was checked with an 30cc extradin ion chamber. The charge collected was compared with the 8MU delivered in the fixed gantry position.

[Downloaded free from http://www.jmp.org.in on Friday, July 29, 2011, IP: 130.14.254.25] || Click here to download free Android application for t

**Results and Discussion:** The preliminary study of the Mega Voltage Cone Beam images for aligning the patient performed on 4-5 patients has given us the large amount of information, which is being analyzed objectively. In all the patients, the shift that needs to be performed turned out to be with in 3-5 mm. This may be due to the prior information available to the technologists that this patient will be subjected to the Mega Voltage Cone Beam and hence very careful positioning has made the errors low. The dose delivered to the patient was checked suing an 30cc ion chamber and it was within 1.2%. Our preliminary experience shows that the Mega Voltage Cone Beam CT guided alignment and treatment delivery meets the requirements of the situation and can be safely used.

**P-29**

**A comparitive study between CT based computerized template treatment planning and standard template planning in interstitial brachytherapy: Our experience**

NVN Madhusudhana Sresty, KR Muralidhar, A Krishnam Raju, S Thirumalai Swamy, S Shiyama, Pramod Kumar Dixit, Rajaneesh Kumar, PV Naidu. *Indo American Cancer Institute and Research Centre, Hyderabad, India*

**Introduction:** Interstitial Brachytherapy is one of the major treatment modality for the treatment of gynecological cancers. Many institutes are practicing template Brachytherapy treatments. But very few centers are equipped with CT simulator, 3D planning system for CT based 3D computerized planning. In some of the centers usual practice is to calculate the doses and treatment time with an already created standard template using planning system. In reality, template configuration will change from partially to large extent after the application in the patient's body. The purpose of this study is to find out the difference in many parameters like target coverage, patient dose, doses to the critical structures etc between the two methods.
**Materials and Methods:** Our institute is equipped with Varian Brachyvision treatment planning system Siemens CT simulator and Syed-Neblett applicator. 5 patients of ca cervix treated with HDR implant Brachytherapy has been studied. Post implant CT slices of 5 mm thickness were taken. CT data are sent to Brachyvision treatment planning system. Clinician drew target volume and other critical structures like bladder and rectum etc. Localization of the needles had done by focusing and rotating the sagittal, frontal and transversal views of the plane of each needle. By proceeding in this way, one can localize all the needles very accurately. Then plan was optimized and executed. In Brachyvision one can download standard template for planning. Making use of this facility fixed template was inserted and adjusted by viewing in all the planes. Length of each needle and step size was fixed as in CT based planning. Planning was done and optimized. For the best comparison results, plans were obtained without any optimization also.
**Results and Discussions:** Unoptimised plans comparison: In CT based plans 100% dose is covered by 99.7%, 92%, 100%, 98% and 100% volume where as in standard template plans this is 63.6%, 87%, 92%, 80% and 58% volume respectively for the 5 patients. In 2 patients, CT based planning showed upto 6% less patient dose and 15% more patient dose in one patient compared with standard plan. In remaining 2 patient negligible differences are obtained between two plans. Target mean dose is up to 1.5 times more on average, in all cases in CT based 3d planning compared with standard template. 2 patient plans showed 9% and 28% less bladder dose, one patient plan showed 52% more bladder dose compared with standard plan. For 2 patient plans negligible variation is observed. 3 patient plans showed 38%, 14% and 7% more rectal doses and one patient plan showed 56% less rectal dose compared with standard template plan. Optimized plans comparison: In CT based plans 100% dose is covered by 98.6%, 99%, 100%, 98% and 100% volume where as in standard optimized implant plans 96%, 90%, 98%, 90% and 94% respectively. In 4 patients, CT based planning showed 26%, 5%, 15% and 10.5% less patient dose, in one patient 3% more patient dose compared with standard plan. Target mean dose is more up to 50% on average in 2 patients and 29% less an on average in 2 patients, in CT based 3D planning compared with standard template. Negligible variation found in one patient. CT based plan showed less bladder doses up to 15% on average in 3 cases and 9% more dose in one case. Rectum doses are less up to 35% on average in 3 cases and one plan showed 29% more when compared with standard plan.
**Conclusion:** Standard template plan users cannot optimize the doses because of the lack of 3D treatment planning system. So, for the good comparison purpose, unoptimized plans are important though we have studied and compared optimized plans also. If we analyze the results big differences in target coverage, patient mean dose, target mean dose and doses to critical structures between CT based 3D planning and standard template planning are observed. Though we have not calculated, one can easily estimate large differences in dose uniformity and conformity between two methods. Treatment goals are not achievable by delivering doses with fixed template planning. With needles itself this much variation was found, then one can easily estimate the difference in the doses if the implant (Head and Neck, breast etc.) is with wires where the chances of bending is more. So, CT based 3D planning are only reliable because of the ability to identify the exact implant. This remarkable variation to be given a thought for the benefit of the patient treatement. Institutes who are practicing implant therapy should try to procure necessary equipments for the prefect planning and execution.

**P-30**

**Inclusion of BRAGG-gray stopping-power ratios in the Monte Carlo-based EGSnrc user-code SPRRZnrc**

T Palani Selvam, DWO Rogers*. *Radiological Physics and Advisory Division, Bhabha Atomic Research Centre, Mumbai - 400 094, India*
**Carleton University, Ottawa, K1S 5B6, Canada*

**Introduction:** Spencer-Attix (S-A) cavity theory is considered more accurate than Bragg-Gray (B-G) cavity theory, but the latter is still applied in cavity-based radiation dosimetry studies. The Monte Carlo-based EGSnrc user-code SPRRZnrc which already calculates S-A stopping power ratios, is therefore modified to include the B-G stopping-power ratio. The coding is done using the MORTRAN programming language. In order to verify that the existing coding of the S-A stopping-power ratio is undisturbed we repeated many published studies using the modified SPRRZnrc code. Only limited results are presented in this note.
**Materials and Methods:** B-G stopping-power ratio calculation in the SPRRZnrc code The B-G stopping-power ratio of medium $m_1$ to medium $m_2$ is given by:

$$(\bar{S}/\rho)^{m_1}_{m_2} = \frac{\int_0^{E_{max}} \varphi_{P,m_1} [S/\rho]_{m_1} dE}{\int_0^{E_{max}} \varphi_{P,m_1} [S/\rho]_{m_2} dE} \qquad (1)$$

where $\varphi_{P,m1}$ is the differential fluence spectrum of primary charged particles in medium $m_1$ in the region of interest and $[S/\rho]_{m1}$ and $[S/\rho]_{m2}$ are the unrestricted mass collision stopping-powers of media $m_1$ and $m_2$ respectively. In a photon beam, $\varphi_{P,m1}$ includes photon-generated electrons and positrons. The knock-on electrons ($\delta$-rays) produced by these charged particles are not part of $\varphi_{P,m1}$. In a charged particle beam, the $\delta$-rays are not part of $\varphi_{P,m1}$. The basic approach of scoring the total dose deposited in two different media, as represented by the numerator and denominator in equation (1) is as follows. The transport of particles takes place in the transport medium, $m_1$, not in the detector medium, $m_2$ i.e., there is no cavity in the calculation. Photons and charged particles are transported down to the energies of AP=PCUT (photon kinetic energy) and AE=ECUT (total energy of primary charged particles) respectively. Energy deposition by the primary charged particles from different energy deposition events is scored using an "on-the-fly" technique. A detailed report describing mathematical background, implementation of the B-G theory, preparation of cross-section data sets and input to the code and various tests carried out, is available from the authors on request.

[Downloaded free from http://www.jmp.org.in on Friday, July 29, 2011, IP: 130.14.254.25] || Click here to download free Android application for t

Table 1: Comparison of Spencer-Attix and Bragg-Gray water-to-air stopping-power ratios calculated based on new version of the EGSnrc user-code SPRRZnrc and those calculated by Malamut *et al* for broad parallel beams of monoenergetic photons on a uniform water medium.

| MeV | Malamut et al Med. Phys. 18 1222-1228 (1991) ICRU Rep 35 stopping powers used | | | new SPRRZnrc code (present study) ICRU Rep 37 stopping powers used | | |
|---|---|---|---|---|---|---|
| | Bragg-Gray | Spencer-Attix | % diff | Bragg-Gray | Spencer-Attix | % diff |
| 1 | 1.132 | 1.136 | 0.4 | 1.132 | 1.135 | 0.3 |
| 5 | 1.101 | 1.107 | 0.6 | 1.095 | 1.100 | 0.5 |
| 10 | 1.074 | 1.079 | 0.5 | 1.064 | 1.069 | 0.5 |
| 20 | 1.042 | 1.046 | 0.4 | 1.031 | 1.035 | 0.4 |
| 40 | 1.007 | 1.011 | 0.4 | 0.999 | 1.002 | 0.3 |

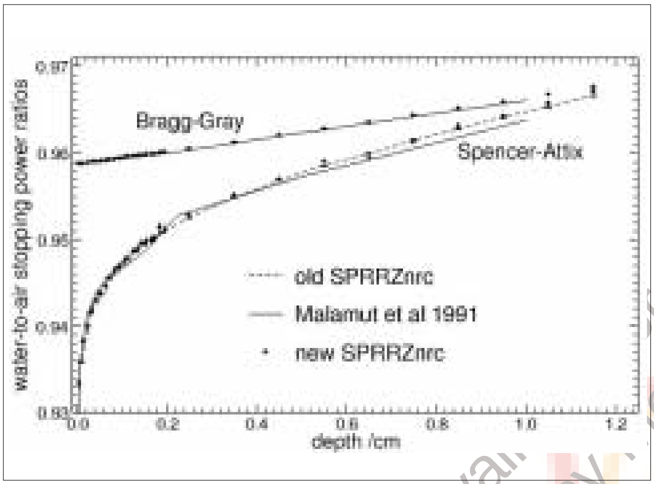

**Figure 1:** Water-to-air S-A and B-G stopping-power ratios for a broad parallel beam of 20 MeV electrons incident on a water phantom

**Results and Conclusion:** Table 1 compares the differences between S-A and B-G stopping-power ratios for different broad parallel monoenergetic photon beams as published by Malamut *et al* using ICRU Report 35 stopping powers to the present differences calculated using ICRU Report 37 stopping powers. The AE=ECUT value used in the calculations is 521 keV. The differences shown in the table should be independent of the stopping powers used. The values presented for 40 MeV are at 15 cm depth in water whereas for the remaining beam energies the values are at 10 cm depth. The differences between the B-G and S-A stopping-power ratios that are based on ICRU Report 35 stopping-powers are within 0.1% of the corresponding differences that are based on ICRU Report 37 stopping-powers. This implies that the new version of the SPRRZnrc code calculates the B-G stopping-power ratios correctly for photon beams. Figure 1 presents, as a function of depth, the values of water-to-air S-A and B-G stopping-power ratios calculated in the present work and previously published by Malamut *et al* for a broad parallel beam of 20-MeV electrons incident on water. The S-A stopping-power ratios are shown based on the new and old versions of the SPRRZnrc code. The present values of B-G stopping-power ratios are in good agreement with those calculated by Malamut *et al*, which are based on ICRU Report 37 electron collision stopping powers. This once again confirms that we have implemented B-G theory correctly. The new and old versions of the SPRRZnrc codes produce the same values of S-A stopping-power ratios. This confirms that the existing S-A coding is undisturbed. The results suggest that this new version of the SPRRZnrc code may safely be used for calculating B-G and S-A stopping-power ratios.

**P-31**

**A semi-analytic model to calculate 2-D dose-rate distributions around $^{60}$CO Hdr brachy sources**

T Palani Selvam. *Radiological Physics and Advisory Division, BARC, CT and CRS, Anushaktinagar, Mumbai - 400 094, India*
E-mail: pselvam66@yahoo.co.in

**Introduction:** $^{60}$Co sources are still in use in some countries mainly for treating cervical cancers. Recently, Monte Carlo-based dosimetry study has been reported for three $^{60}$Co HDR sources used with the Ralston remote afterloader (Shimadzu Corporation, Japan). The present study is aimed at calculating 2-D dose-rate distributions in water around $^{60}$Co HDR sources based on a semi-analytic model.

**Materials and Methods:** Radioactive sources: The three source designs used with Ralston remote afterloader incorporate two $^{60}$Co cylindrical active pellets. The pellets are encapsulated in a 3 mm in diameter stainless steel cylinder. Each source pellet is 1 mm long and 1 mm diameter. In type 1 and type 3 sources the pellets are spaced 9 mm and 11 mm apart, respectively whereas in type 2 source the pellets are in contact.

**Description of semi-analytic model:** A general expression for calculating 2-D dose-rate distributions in water D(Y,Z), in units of $cGyh^{-1}U^{-1}$ for a cylindrical source is given below:

$$D(Y,Z) = [\mu_{en}/\rho]^{wat}_{air} \, B\exp(-\mu r) \, G(Y,Z) \quad (1)$$

where ($[\mu_{en}/\rho]^{wat}_{air}$= 1.112) is the ratio of mass-energy absorption coefficient of water to that of air and $B\exp(-\mu r)$ is the combined energy-absorption-build-up and attenuation factor in water at r from a point $^{60}$Co source and G (Y,Z) is geometry factor that takes into account activity distribution of the source. The values of $B\exp(-\mu r)$ are calculated by using the MCNP code for r = 0.5-15 cm by assuming a point photon

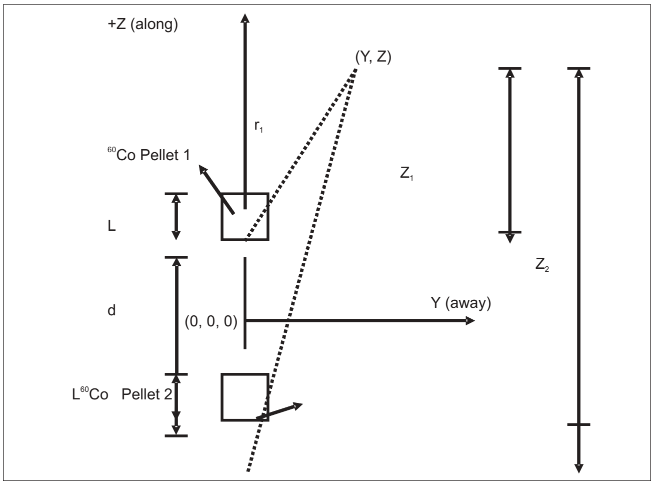

**Figure 1:** Coordinate system used for analytical calculations of D(Y,Z) for type 1 and type 3 $^{60}$Co sources. The values of d are 0.9 and 1.1 cm, respectively, for type 1 and type 3 sources and active length L = 0.1 cm for both the sources

This PDF is available for free download from a site hosted by Medknow Publications (www.medknow.com)

[Downloaded free from http://www.jmp.org.in on Friday, July 29, 2011, IP: 130.14.254.25] || Click here to download free Android application for t source of energy 1.25 MeV suspended at the centre of a 20-cm-radius water phantom. Equation (1) is applicable for source capsules incorporating single $^{60}$Co active pellet (like type 2 source). For capsules incorporating two $^{60}$Co active pellets (each pellet having an active length L) separated apart by a distance, d (like type 1 and type 3 sources), equation (1) is modified as follows:

$$D(Y,Z) = \frac{1}{2}\,[\mu_{en}/\rho]_{air}^{wat}\,\{Bexp\,(-\mu r_1)\,G(Y,Z_1) + Bexp\,(-\mu r_2)\,G\,(Y,Z_2)\} \quad (2)$$

For a given Z, the parameters, $Z_1$, $Z_2$, $r_1$ and $r_2$ are calculated as follows:

when Z = 0; $Z_1 = Z_2$ and $r_1 = r_2$

when 0 > Z d” d/2 + L/2; $Z_1 = d/2 + L/2 - Z$ and $Z_2 = d + L - Z_1$

when Z > d/2 + L/2; $Z_1 = Z - (d/2 + L/2)$ and $Z_2 = Z_1 + d + L$

$r_1 = \sqrt{Y^2 + Z_1^2}$ and $r_2 = \sqrt{Y^2 + Z_2^2}$

The coordinate system used is shown in Figure 1 along with description of the parameters, $Z_1$, $Z_2$, $r_1$, $r_2$, d, Z, Y and L.

**Results and Conclusion:** When Z = 0 and Y = 1 cm, equations (1) and (2) represent expressions for $\Lambda$. Thus, equation (1) gives $\Lambda$ = 1.092 for type 2 source and equation (2) gives $\Lambda$ = 0.874 and 802, respectively, for type 1 and type 3 sources. These values are in excellent agreement with the published values that involved accurate modeling of the sources. A comparison of D(Y,Z) data for type 1, type 2 and type 3 $^{60}$C sources obtained in the present work against the published Monte Carlo-based data suggests that the proposed model can safely be used for Z = 0 to 1.5 cm and for a any Y value between 0.5 cm and 10 cm. The agreement in these regions is because of the fact that the encapsulation effect cancels out as dose rate distribution is normalized to unit air-kerma strength. For Y values close to source axis due to dominance of self-attenuation by the source our model predicts inaccurate results when Z > 1.5 cm. To address this issue a function describing anisotropy should be included in the model. This work is in progress. The proposed model in the present form enables to calculate two important dose parameters such as $\Lambda$ and radial dose function, g(r) accurately. The proposed model may serve as a powerful tool for medical physicists to verify these parameters in the treatment planning system.

### P-32

### Characteristics study of radiochromic films by using radiotherapy beams

BK Padmanaban, S Prabakar*, S Ashok Kumar, S Gopi**. *Dept. of Radiation Physics, BIBI General Hospital and Cancer Center, Hyderabad - 500 024, *Department of Physics, Bharathiar University, Coimbatore - 641 046, India, **Dept. of Radiation Oncology, Pomona Valley Hospital and Cancer Care Center, California, USA*

**Introduction:** Photon, electron and proton radiations are used extensively for medical purposes in diagnostic and therapeutic procedures. Dosimetry of the radiation sources can be performed with radiochromic films, devices that have the ability to produce a permanent visible color change upon irradiation. Radiochromic film dosimeters now available in the formats which accurate dose measurements range from less than 1 Gy upto many kGy. In this project, we studied the characters of the radiochromic film and the corresponding optical densities are measured.

**Materials and Methods:** The characteristics of the radiochromic films i.e., optical density, dose fractionation, post irradiation coloration properties, dose rate, uniformity in film response, ambient and readout light source effect, ultraviolet dosimetry, polarization effect, energy dependence of radiochromic film, dosimetry in water phantom orientation of exposure effect, heat treatment processes for radiochromic films are studied by keeping the radiochromic film in perspex sheets and water phantoms at a depth of 5 cm from SSD of 80cm/100cm with a field size of 10 cm$^2$. The optical densities of the films are measured by "Radiochromic Film Reader" supplied by Far West Technology Inc".

**Results and Analysis:** From the results, we analyzed that the

- The optical density property of radiochromic film is true and the radiochromic film is turned into blue color when the film is irradiated.
- The results of dose fractionation effect of the radiochromic films are shown that the film is independent of the dose fractionation.
- After irradiation of the radiochromic film for the particular dose causes post exposure density growth which manifests itself as a significant increase in the optical density.
- The effect of dose rate of delivery on the response of radiochromic film has been found that the film is relatively dose-rate independent.
- An ideal film dosimeter will provide a uniform response unless the optical structure of the film active layer and other components defects in the film structure as well as scratches or other foreign materials such as dust particles.
- Ambient and readout light sources for densitometry can also have an effect on the Radiochromic film if there is a UV component to the source.
- The radiochromic films are sensitive to ultraviolet radiation in varying degrees producing coloration. Both sunlight and fluorescent light sources can effect the coloration of radiochromic film due to the UV content in their beam intensity.
- Various layers in the construction of radiochromic films contain mylar protective coatings, radiosensitive gels and glue. These types of constructions can produce effects based on the polarization qualities of the readout light sources used for optical density evaluation
- The radiochromic film is energy independent. The film is irradiated with different qualities of beams with different energies with

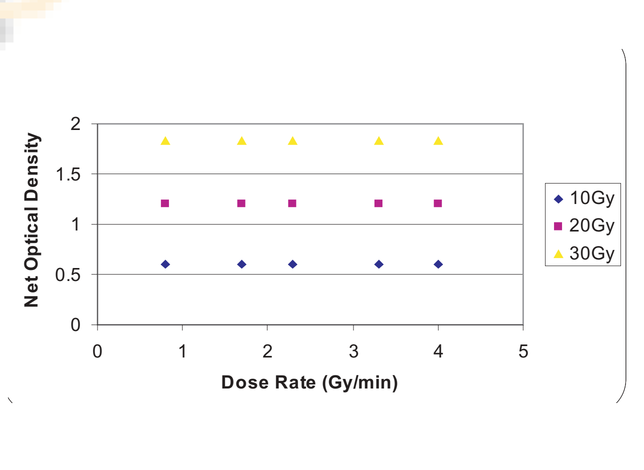

**Graph:** Dose rate dependence of a radiochromic film at typical medical linear accelerator dose and level deliveries

Table: Dose fractionation effects for GAFCHROMIC MD-55-2 radiochromic film

| Sl. No | Different beam quality | Total dose | No. of measurements | | Optical density value | |
|---|---|---|---|---|---|---|
| | | | For 1 fraction (Film 1) | For 5 fraction at 30 minutes gap (Film 2) | For 1 fraction (Film 1) | For 5 fraction at 30 minutes gap (Film 2) |
| 1 | 6 MV Photon | 4000cGy | 10 | 10 | 1.14 | 1.14 |
| 2 | 18 MV Photon | 4000cGy | 10 | 10 | 1.15 | 1.14 |
| 3 | Co-60 Beam | 4000cGy | 10 | 10 | 1.14 | 1.15 |
| 4 | 12 Mev Electron | 4000cGy | 10 | 10 | 1.15 | 1.15 |

This PDF is available for free download from a site hosted by Medknow Publications (www.medknow.com)

[Downloaded free from http://www.jmp.org.in on Friday, July 29, 2011, IP: 130.14.254.25] || Click here to download free Android application for t constant doses. The optical density of the film remains same.

- It is noted that there is no measurable changes in the optical density properties within the radiochromic film due to the effects of water over a period of 24 hours.
- Variations in measured radiation doses can occur due to the orientation of the film placed in the radiation field and the isotropic nature of the radiation.
- The chemical reactions occur within the active structure of the film by post-irradiation heat treatment process. Post-irradiation heat treatment of radiochromic films is a well-established and recommended process. The amount of heating and temperature of optimal response is dependant on the film product in use.

**Conclusions:** The Radiochromic film has become an essential part of radiation dosimetry. Properties such as its relatively energy-independent response to radiation and high spatial resolution make it ideal as a medical radiation dosimeter. Other advantages of the Radiochromic film include its high precision, accuracy, large measurement range, dose-rate independences, ease of handling and no processing requirement. Since this dosimeter possesses a number of unique features, it is anticipated that with use of this dosimeter in water, Medical Physicists have already developed a number of important applications including proton dosimetry, SRS and SRT, HDR Brachytherapy and IMRT for Radiochromic film.

**P-33**

**A comparative study and out-come of radical dose to carcinoma of esophagus by external beam radiotherapy alone and external beam radiotherapy + intra luminal brachytherapy**

Mukund B Sarje, Vandana Jain, KK Singh, Rahul Umbarkar, Ajit Kulkarni, Rajeev Shrivastava, RS Shinde, PM Dighe. *Pravara Rural Hospital, Loni, Maharashtra, India*

**Introduction:** The purpose of this study is to achieve dose uniformity by both External Beam Radiotherapy (EBRT) and External Beam Radiotherapy + Intra Luminal Radiotherapy (EBRT + ILRT) and outcome of it for the cases of carcinoma of esophagus with various combination of THERAPLAN-TPS planning for external beam radiotherapy (EBRT) and HDR- Intraluminal Radiotherapy (ILRT) with various combination and optimization modes of Gamamed-HDR/PDR Brachy therapy system.

**Materials and Methods:** 30 patients were considering for discussion from 1997 to 2005. All of them were diagnosed cases of Carcinoma of Esophagus of different regions from Upper 1/3 to Lower 1/3. 10 patients were female with age group ranging from 40 to 65 years with mean age of 51 and remaining 20 patients were male with age group ranging 30 to 83 with mean age of 54. Study shows the site distribution was cervical and upper 1/3(7), middle 1/3(12) and lower 1/3(11). 18 patients with 40cGy EBRT followed by 4 fractions or 2 fractions (#) of ILRT by Gamamed-12i HDR/PDR Brachytherapy with Ir-192 source of 10 Ci. Strength with different diameter of esophageal applicators (1.0 cm, 1.2 cm, 1.4 cm) or by Ryle tubes of different diameter (0.6 cm and 0.8 cm) were treated with ABACUS Gamamed 12i treatment planning system with iterative correction method, so that homogeneous dose distribution can occurred. All these patients having target volume greater than 5 cm were treated by taking Barium sallow and taking AP-LAT. radiograph for deciding length and width of target for radiotherapy. All were treated by external beam radiation by Co-60. Analysis has been done with all 30 patients giving external beam RT alone with dose of 40Gy/25#s (180cGy/#) or 40Gy/20# (200cGy/#) using two fields AP/PA. Then after patients were planned for three fields, one anterior and two posterior oblique to save the spinal cord. 10 Gy dose is delivered by three fields by giving weightage to reduce the dose to spinal cord while delivering homogeneous and uniform dose to target volume. Then patient was examine for fitness test if patient is fit then decided for HDR (ILRT) Brachytherapy, otherwise continued with same field up to 60Gy / 66Gy. If patient is fit for brachytherapy, after gap of 15 days patient is allowed for Brachytherapy. IN Gamamed 12i HDR- ILRT. In our hospital total dose of 16 Gy.in 4 fractions (4 Gy/#) or 10 Gy in 2 fractions (5 Gy/#) were delivered. First esophagus applicator applicator (of different diameter 1.0 cm, 1.2 cm, 1.4 cm) or Ryle.

**P-34**

**Analysis of various optimizations with the help of dose volume histogram of a surface mould brachytherapy in a hard palate**

DK Ray, A Kumar, T Maji, P Chaudhuri, P Kumar, S Bhattacharya. *Department of Radiotherapy, Chittaranjan National Cancer Institute, 37, S. P. Mukherjee Road, Kolkata - 700026, India*
E-mail: dkray_cnci@rediffmail.com

**Introduction:** Over the years, tools of brachytherapy application and treatment planning have been evolved. The calculation tools changed from manual look-up tables to computerised dosimetry systems and the standard approach to brachytherapy source distribution became more quimby like. Since the ultimate aim of radiation therapy is to deliver the dose as homogeneously as possible within the clinical target volume. The dwell time programming features of High Dose Rate (HDR) Brachytherapy offers optimization of dwell times for desired dose distribution. Most frequently planning is performed with the computer algorithm that uses various optimization methods like Geometry optimization (Distance, Volume), Dose Point Opmization (Distance, Volume), Specific Points (Patient, Applicator), Manual dwell weight and Manual dwell times. Dose Volume Histogram (DVH) is extremely useful for evaluating dose distribution and treatment plan for individual patient. In the present study of a surface mould brachytherapy plan of a recurrent Hard Palate, the various optimization procedure were analyzed with the help of Dose Volume Histogram (DVH), isodose distribution in each planes and careful observation of dwell times in individual catheter and the comparative results have been presented.

**Materials and Methods:** The palatal surface mould was prepared by our dentist with dental material, as it has to be fixed below hard palate in the mouth. Five flexible catheters, single plane were placed 1 cm apart to each other in hemispherical curved plane. The thickness of mould towards the target was 2 mm. The radio opaque clip was placed inside the mould to delineate target volume for treatment planning. Two radiographs were taken from AXIM II radiotherapy simulator for variable angle reconstruction of catheters and markers. The reconstruction and treatment planning were carried out in 3D PLATO Treatment Planning System and the treatment was delivered by HDR Microselectron Brachytherapy Machine. After reconstruction of catheters and marker points, the dwell positions were placed within the length of planning target volume. The dose points were selected at 1cm towards target side from all dwell positions and 5 mm lateral from first and last catheters dwell positions. The plan was optimized by Dose Point Optimization (Distance and Volume) and Geometric Optimization (Distance and Volume). The DVH of all four optimizations

Table 1: DVH parameters for various optimizations

| *DVH parameters* | *Dose point optimization* | | *Geometric optimization* | |
|---|---|---|---|---|
| | *Distance* | *Volume* | *Distance* | *Volume* |
| U.I | 0.925 | 0.950 | 0.917 | 0.917 |
| V.G.R | 0.471 | 1.02 | 1.04 | 0.618 |
| $V_{100}$ | 16.75 cc | 17.53 cc | 16.68 cc | 16.46 cc |
| $V_{150}$ | 9.39 cc | 9.65 cc | 9.30 cc | 9.18 cc |
| $V_{200}$ | 6.12 cc | 6.23 cc | 5.98 cc | 5.87 cc |

Table 2: Catheters time in various optimizations

| *Catheter no.* | *Catheter time (Sec)* | | | |
|---|---|---|---|---|
| | *Dose point optimization* | | *Geometry optimization* | |
| | *Distance* | *Volume* | *Distance* | *Volume* |
| 1 | 0.7 | 15.9 | 34.4 | 38.7 |
| 2 | 78.3 | 35.9 | 31.3 | 29.9 |
| 3 | 0.0 | 59.5 | 31.2 | 28.0 |
| 4 | 88.6 | 45.8 | 30.8 | 27.7 |
| 5 | 0.9 | 15.4 | 34.9 | 36.8 |

[Downloaded free from http://www.jmp.org.in on Friday, July 29, 2011, IP: 130.14.254.25] || Click here to download free Android application for t were generated with 50,000 sample points. The prescription dose to the target volume was 350 cGy per fraction. Uniformity Index (U.I), Volume Gradient Ratio (VGR), Volume covered 100% of Prescription Dose ($V_{100}$), Volume covered 150% of Prescription Dose ($V_{150}$) and Volume covered 200% of Prescription Dose ($V_{200}$) were calculated from DVH. The U.I quantifies how well a target volume is covered by dose distribution and the VGR quantifies the extent by which the implant histograms differs from the point source and specifically the height of the difference and whether this difference is mainly concentrated in the region enclosed by $D_{95}$ and $D_{105}$.

**Results:** The DVH parameters U.I, VGR, $V_{100}$, $V_{150}$ and $V_{200}$ are shown in Table 1. The total dwell times for all dwell positions in individual catheter are shown in Table 2. U.I and volume covered by different isodose lines are almost equal in all four optimized planning. However, V.G.R in Dose Point optimization -distance and Geometric optimization-volume is less than one. Catheters time in Geometric optimization (distance and volume) are almost homogeneous but in Dose Point optimization - distance catheters time of every alternate catheters are negligible that cause unequal dose distribution especially very high dose near dwell positions.

**Conclusion:** The analysis of DVH parameters is not alone sufficient but observation of catheter time and isodose lines in different planes are also essential before finalisation of brachytherapy planning.

### References

1. Anderson LL. A “natural” volume - dose histogram for brachytherapy. Med Phys 1986;13:899-903.

### P-35

### Experimental verification of dependence of wedge factor on field size and energy in universal wedges

DK Misra, DK Ray, T Maji, P Chaudhuri, TP Sinha*. *Department of Radiotherapy, Chittaranjan National Cancer Institute, 37, S.P. Mukherjee Road, Kolkata - 700 026, *Department of Physics, Bose Institute, 93/ 1, A.P.C. Road, Kolkata - 700 009, India*
E-mail: dkray_cnci@rediffmail.com

**Introduction:** The wedge filter is the most commonly used beam modifying device. This is a wedge shaped absorber that causes a progressive decrease in the intensity across the beam, results in a tilt of isodose curves from their normal positions. Wedge filters are of two types:

1. Individualized wedge, which requires separate wedge for different field sizes.
2. Universal wedge, which is a single wedge, serves for all the field sizes.

In the present study the wedge transmission factors of universal wedges were measured for various field sizes for 6 and 18 MV X-ray beams and dependence of wedge factor on field sizes and energies were studied and their results have been presented.

**Materials and Methods:** We have dual energy Linear Accelerator of Siemens (Model-KD-2, Mevatron) having 6 and 18 MV X-rays and six electron energies ranging from 6 to 18 MeV. The output of 6 and 18 MV X-rays were measured with and without wedge filter for field sizes 5x5 cm$^2$ to 20x20 cm$^2$ at 10 cm water equivalent depth in solid phantom (PMMA Sheets) with 0.6 cc Farmer Ionization Chamber and PTW, UNIDOS Secondary standard Dosimeter. We have universal wedges supplied with the Accelerator having wedge angle 15, 30, 45 and 60 degrees. The maximum wedge field size is 25x30 cm$^2$ for 15, 30 and 45 degree wedges and that of 20x25 cm$^2$ for 60 deg wedge. The output was measured for 200 MU. The wedge factors obtained from the ratio of outputs with the wedge and without wedge for various field sizes.

Table 1: Wedge factors and % variation of wedge factors with respect to 10x10 cm$^2$ field size for 30 deg wedge

| *Field size In cm x cm* | *Wedge factors* | | | |
|---|---|---|---|---|
| | *6MV* | | *18 MV* | |
| | *Wedge factor* | *Variation (in %)* | *Wedge factor* | *Variation (in %)* |
| 8x8 | 0.5190 | + 0.04 | 0.5938 | + 0.08 |
| 10x10 | 0.5192 | 0.0 | 0.5943 | 0.0 |
| 12x12 | 0.5201 | - 0.17 | 0.5960 | - 0.29 |
| 15x15 | 0.5253 | - 0.61 | 0.5993 | - 0.84 |
| 20x20 | 0.5259 | - 0.67 | 0.6020 | -1.3 |
| 25x25 | 0.5262 | - 0.70 | 0.606 | -1.9 |

Table 2: Wedge factors for 60 deg wedge and percentage variation of wedge factors with respect to 10x10 cm$^2$ field size.

| *Field size In cm x cm* | *Wedge factors* | | | |
|---|---|---|---|---|
| | *6 MV* | | *18 MV* | |
| | *wedge factor* | *Variation (in %)* | *wedge factor* | *Variation (in %)* |
| 5x5 | 0.3325 | +1.9 | 0.415 | +1.19 |
| 8x8 | 0.336 | + 0.88 | 0.416 | + 0.95 |
| 10x10 | 0.3396 | 0.0 | 0.420 | 0.0 |
| 12x12 | 0.3392 | - 0.06 | 0.422 | - 0.48 |
| 15x15 | 0.3410 | - 0.59 | 0.425 | -1.19 |
| 20x20 | 0.3414 | - 0.71 | 0.428 | -1.9 |

**Result:** The wedge factors for various field sizes for 6 and 18 MV X-rays for 30 deg and 60 deg are shown in Tables 1 and 2 respectively. From Tables 1 and 2 it is observed that wedge factor varies with field size. The wedge factor increases with increasing field size. The maximum variation of wedge factor for 30 degree wedge with field size was - 0.7% to + 0.04% and -1.9% to + 0.08% for 6 and 18 MV respectively. The maximum variation of wedge factors for 60 degree wedge with field size was - 0.71% to + 1.9% and - 1.9% to + 1.19% for 6 and 18 MV respectively. It is also observed that variation of wedge factor with field size was more in 18 MV than that in 6MV. Further it is also clear from the Tables 1 and 2 that the wedge factor increases with the energy and decreases with the wedge angle for a given field size.

**Conclusion:** We had used universal wedge for study and found that wedge factor varies with field size and energy. Therefore it is suggested that the user should measure wedge factor for various field sizes and for all available photon energies for use in treatment time calculation.

### P-36

### Measurements of electron output by 0.6 cc farmer chamber and plane parallel plate markus chamber: A comparative study

Rajib Das, DK Ray, DK Misra, S Pal*. *Department of Radiotherapy, Chittaranjan National Cancer Institute. 37, S P Mukherjee Road. Kolkata - 700 026, India. *Variable Energy Cyclotron Centre, 1/AF, Bidhannagar, Kolkata - 700 064, India*

**Introduction:** The electron beams of Linear Accelerator are used in Radiation Therapy for treating superficial tumors with a characteristically sharp drop-off dose beyond the tumor. Since the electron output depends on head design of individual Linear accelerator, therefore the output of different energies for each accelerator is measured separately and periodically. The TG-51 Protocol recommends the use of Plane-Parallel Plate Ionization Chamber for calibration of electron beams with energies 6 MeV or less and either Plane-Parallel Plate or cylindrical Ionization chambers for higher energy electron beams. In the present study the output of electron beams of different energies were measured by plane-parallel Plate Ionization chamber as well as 0.6 cc Farmer cylindrical ionization chamber for verification purpose and their comparison has been presented.

**Materials and Methods:** A Markus Parallel plate Ionization chamber PTW (active volume 0.055 cc) and 0.6 cc Cylindrical Farmer Ionization chamber, PTW were used to measure output of electron energies 6,

This PDF is available for free download from a site hosted by Medknow Publications (www.medknow.com)

[Downloaded free from http://www.jmp.org.in on Friday, July 29, 2011, IP: 130.14.254.25] || Click here to download free Android application for t

Table 1: The % deviation of output measurement for various electron energies

| Energy (MeV) | Measured output by Markus chamber (cGy/min) | Measured output by 0.6cc Farmer chamber (cGy/min) | % deviation of farmer chamber with Markus chamber |
|---|---|---|---|
| 6 | 0.997 | 1.029 | 3.2 |
| 8 | 1.043 | 1.048 | 0.48 |
| 10 | 1.113 | 1.128 | 1.3 |
| 12 | 0.855 | 0.865 | 1.2 |
| 15 | 1.1015 | 1.107 | 0.45 |

8,10,12 and 15 MeV of linear Accelerator (Siemens, KD-2) at this Institute. All the output were measured for 15x15 electron applicator at the depth of maximum dose for SSD 100 cm in Solid Phantom (PMMA Sheet) and Secondary Standard dosimeter (UNIDOS, PTW) was used for measurement. TRS 381 Protocol was followed for output calibration. The required Dosimetric parameters like dmax, Rp, R50 were obtained from Central axis PDD of various electron energies by Radiation Field Analyser MP3S, PTW. The formula used for output measurement was-

Dw, dmax = $M_{Rcorr}$ X $N_{Dair}$ X $S_{w, air}$ X Ps X $P_Q$ X $h_m$

Where $M_{Rcorr}$ = $M_R$ X $K_{TP}$X $K_S$

$M_{Rcorr}$ = Corrected meter reading for temp-pressure and polarization.

$N_{Dair}$ = Absorbed dose to air calibration factor.

$h_m$ = Electron fluence correction, plastic to water.

Ps = Ion recombination factor

$P_Q$ = Overall perturbation factor

**Results and Discussion:** The % deviation of measured output for various electron energies by 0.6 cc Cylindrical Farmer Ionization chamber and Markus Parallel plate Ionization chamber is shown in Table 1. The % deviation of the measured output was maximum for 6 MeV electron beam whereas % deviation was less than 1.5% for all other energies greater than 6MeV. More deviation for 6MeV is due to the fact that Plane Parallel Plate Chamber with a thin window and a small electrode separation offers the least perturbation to the electron field whereas for Cylindrical Ionization Chamber with large cavity size gives rise to significant perturbation at low energies. This also confirms the earlier result by different authors. Therefore 0.6 cc Cylindrical Ionization Chamber can also be used for output calibration of all electron energies used in Radiotherapy greater than 6 MeV.

### References

1. Technical Report Series No. 381.
2. Technical Report Series No. 398.
3. AAPM Task Group 51.

### P-37

### The difference in the bladder and rectum doses with different applications of high dose rate intracavitary brachytherapy in carcinoma of the uterine cervix treated at a rural centre of Maharastra, India

Vandana Jain, MB Sarje, KK Singh, R Umberkar, R Shrivastava, SM Jain#. *Department of Radiotherapy and #Microbiology, Rural Medical College, PIMS, Loni-413736 (MS), India*

**Introduction:** To report the difference in the bladder and rectum doses with different applications in the same patient of the carcinoma of the uterine cervix treated by multiple fractions of high-dose-rate (HDR) intracavitary brachytherapy (ICBT).

**Materials and Methods:** Between Jan. 2003 to Dec. 2004, a total of 60 cases of the carcinoma uterine cervix were selected randomly for the retrospective analyses. The patients completed their External beam Radiotherapy (EBRT) treatment and HDR- ICBT in time. All 60 cases were grouped in six groups according to the treating radiotherapist who did the HDR-ICRT application. Three radiotherapists were considered for this study, named A, B and C. 10 cases for each radiotherapist in which all three applications were done by same radiotherapist. And 10 cases for each radiotherapist with shared applications in the same patient (A+B, A+C and B+C).

**Results:** There were 10 cases in each six groups. The bladder and rectal doses were calculated in reference to point "A" dose and grouped in three groups. Bladder and rectum doses were limited to 80% of prescribed point "A" dose, as per ICRU-38 recommendations. Dose less then 80% (<80%), 80-100% and above 100% (>100%). In total 180 applications for 60 patients were calculated for the above analyses. The bladder dose for 30 applications for 10 patients by A, B and C radiotherapists were below 80% in 23, 9 and 13 applications, 80-100% in 5, 6 and 12 applications and above 100% in 2, 15 and 5 applications respectively. The rectal doses for 30 applications for 10 patients by A, B and C radiotherapists were below 80% in 24, 19 and 23 applications, 80-100% in 5, 9 and 6 applications and above 100% in 1, 2 and 1 applications respectively. The shared applications for 10 patients by A+B radiotherapists were 16+14 respectively. The shared applications for 10 patients by A+C radiotherapists were 14+16 respectively. The shared applications for 10 patients by B+C radiotherapists were 13+17 respectively. Details of all applications are shown in Table 1.

**Discussion and Conclusion:** There is a lot of difference in the bladder and rectal doses with the application by the different radiotherapists, even in the same patient with multiple fractions of HDR-ICRT. Applications by 'A' radiotherapist were within the limits in the self as well as in the shared groups more number of times, by 'B' radiotherapist was more times exceeding the limit and by 'C' radiotherapist doses were in between the A and B. For the rectal and bladder doses most important factors are patients age, disease stage, duration between EBRT and HDR-ICRT and patient anatomy, but these differences can be minimized to some extent by careful application, proper packing and proper fixation.

### P-38

### An in-house laser CT scanner for reading Normoxic and Fricke gels

B Paul Ravindran, S Brindha, S Timothy Peace, L Ebenezer, S Ebenezer, Suman Babu. *Dept of Radiotherapy, Christian Medical College, Vellore - 632 004, India*

**Introduction:** To construct an in-house single slice laser CT scanner and investigate its performance for evaluating normoxic and Fricke gel dosimeters in radiotherapy application. Advanced radiotherapy techniques based on increased complexity of radiation delivery methods necessitate verification of computer calculated dose distribution by an accurate dosimetric method. Gel dosimeters evaluated with MRI has been used for mapping dose distributions in 3D. Due to the practical problems of availability, cost and artifacts, optical scanning of gel has been used by several researchers as an accurate and practical alternative to MRI.

**Materials and Methods:** The scanner was constructed with a translate-rotate motion. This scanner consisted of an aquarium with a turntable with angular graduation, a red diode laser (~633 nm) as light source and light dependent resistors (LDR) as detectors. The position of the detector was determined by a 10-turn 20K linear potentiometer. A regulated 10 Volt DC was applied to the potentiometer and the LDR. The change in resistance was measured as the variation in the voltage and fed to the PC through a DT 9812 ADC (Data Translation, USA). A cylindrical container of diameter 9.5 cm and height 15 cm was used as the gel container.

**Gel preparation and Data Reconstruction:** The gel used for this study was the normoxic PAG gel named as PAGAT with 5% gelatin, 3.5% BIS, 3.5% AA, 88% distilled water and 10 mM THP as the antioxidant. The gel was scanned before and after irradiation. The ratio of the post and pre scan data were obtained and the CT reconstruction of the optical attenuation data was performed with Matlab

This PDF is available for free download from a site hosted by Medknow Publications (www.medknow.com).

[Downloaded free from http://www.jmp.org.in on Friday, July 29, 2011, IP: 130.14.254.25] || Click here to download free Android application for t

**P-39**

**Effective atomic numbers for dosimetric materials**

BR Kerur, SB Kaginelli, MT Lagare, S Anil Kumar#. *Department of Physics, Gulbarga University, Gulbarga - 585 106, India. #Radiation Safety and System Division, Bhabha Atomic Research Centre, Mumbai - 400 085, India*

**Introduction:** The increasing application of low energy photons/X-rays in a wide variety of fields necessitates the under standing and accurate measurement of photon attenuation coefficients. In medical physics, it is important to measure the amount of radiation delivered by the ionizing radiation. On the other hand, the concept of z-dependence of photon attenuation has led to many applications in radiation studies. For example, the range of tissue substitutes currently available is the result of demands by experimentalists for materials closely stimulating a wide variety of body tissues. During the first half of last century, phantoms with relatively simple geometries are often deemed adequate to represent volumes of muscle for experimental dosimetry. Since than, a steady improvement in dosimetric technique and the requirement for more realistic dose data, has lead to the development of complex phantoms containing simulated muscle, bones, lungs and other tissues and organs. In this regard the effective atomic number has proved to be a convenient parameter for interpretation of X-ray attenuation by a complex medium like a biological tissue and particularly for the calculation of dose in radiography radiation dosimetry etc. The mass attenuation coefficient is proportional to electron density and when the photon energy is lower than about 15 keV, these cross sections values have great importance in many applications. Hence the atomic number or the effective atomic number must be explicitly considered in estimating the mass attenuation coefficients. The X-ray mass attenuation coefficient m/r for any material are usually estimated from the sum of the appropriately weighted contributions from the constituent elements. This is based on the assumption that contributions of each element to the attenuation is additive and the law is know as Bragg's additivity law or more commonly called mixture rule. Thus m/r for any chemical compound/material is given by $\mu/\rho=\Sigma_i (\mu/\rho)_i$, where $(\mu/\rho)_i$ is the mass attenuation coefficient of the $i^{th}$ element and $w_i$ is the fraction by weight of the $i^{th}$ element. For a compound/material with chemical formula $(Z_1)_{a1}$, $(Z_2)_{a2}$, ......$(Z_n)_{an}$ the weight factor for the $i^{th}$ element is given by $w_i =\{( a_i A_i) /(S a_i A_i)\}$, Where $A_i$ is the atomic weight of the $i^{th}$ element. In the literature the effective atomic numbers ($Z_{eff}$) of the dosimetric materials data are not available in low energy X-rays. Hence an attempt has been in this regard to determine the mass attenuation coefficient of X-rays for the dosimetric materials and then determined the $Z_{eff}$ of these materials by LSF method from ln ($\mu/\rho$) Vs. lnZ graph.

**Materials and Methods:** In one of our earlier papers a method is described of determination of mass attenuation coefficient $\mu/\rho$ using a NaI(Tl) detector (Nagabhushan *et al* 2004). g-rays emitted from the radioactive isotope of $^{55}$Fe and $^{65}$Zn. In the transmission experiments a rigid good geometry, with an arrangement, to place the sample normal to the beam and at midway between the source and detector and the transmitted intensity is measured by gating the channels at FWHM position of the photo peak, has been adopted. In the present method the "best value" of mass attenuation coefficient is obtained in the transmission range from 50% to 2%. This is tested for the aluminum elemental foil for the whole transmission range. The attenuation coefficient ($\mu/\rho$) is calculated using the least squares fit method and the results are presented in Table 1. This method gives very good indication that the method can be utilized to determine mass attenuation coefficient for any element or for any sample. Hence we have adopted this method to determine $\mu/\rho$ for dosimetric materials.

**Results and Discussion:** The error involved in over all experimental values is about less than 1% for the elemental foils and about 2 to 3% for the other samples since the thickness of the foils are determined by taking the weight of the sample and determined the area by a traveling microscope. The determined values of $\mu/\rho$ in this transmission range is agreeing well with the theoretical values[1] and the agreement between them is within 2% for the aluminum foil at this energy and result is presented in the Table 1. The determined m/r even for dosimetric materials and the calculated mass attenuation coefficient are agreeing well within 2 to 3%. The extrapolated effective atomic number values of the dosimetric materials are shown in the column 4 of Table 1. These values are determined by extrapolating $\mu/\rho$ values on the [ln ($\mu/\rho$)] Vs. [ln(Z)] graph. The theoretical values of the $Z_{eff}$ are calculated using the formula given[2,3] are presented in 5 and 6 column of Table 1. The experimental values are agreeing with the theoretical values within 5-7%. It is important to mention that the theoretical/calculated values have not considered the edge effects and hence the effective atomic numbers are underestimated when any element falls below the absorption edge. Hence there is a good indication that even in the low photon energy region say at 15 keV the effective atomic number can be determined with greater accuracy but one should take into account of edge effects. In conclusion, the determined $Z_{eff}$ value is agreeing with the theoretical values within 7% at this energy.

**References**

1. Hubbell JH, Seltzer SM. NISTIR 1995;95:5632.
2. Jackson DF, Hawkes JD. Physics Reports 1981;70:169.
3. Wantanabe Yoichi, 1999, Phys. Med. Biol, 48, 222.

**P-40**

**Radiation hormesis: an update of the present position**

CM Pathak, PK Avti, KL Khanduja. *Department of Biophysics, Postgraduate Institute of Medical Education and Research, Chandigarh - 160 012, India*
E-mail: chander_pathak@rediffmail.com

All the living organisms exist in an ocean of ionizing radiations from

Table 1: Mass attenuation coefficient and effective atomic number of the dosimetric materials

| *Name of the sample* | *Mass attenuation coefficient ($m^2/kg$)* | | *Effective atomic number($Z_{eff}$)* | | |
|---|---|---|---|---|---|
| | *Experimental value* | *Theory value* | *Expt.-graph* | *Jackson1981* | *Wantan-abe 1999* |
| 5.947 keV | | | | | |
| Al | 117.6±0.6 | 117.7 | | | |
| $MgSO_4.7H_2O$ | 57.7±1.7 | 57.8 | 10.25 | 9.88 | 9.82 |
| $CaSO_4.2H_2O$ | 145.9±4.3 | 146.7 | 14.01 | 15.08 | 14.73 |
| $MnSO_4.H_2O$ | 74.6±0.4 | 77.0 | 11.18 | 19.33 | 18.98 |
| $FeSO_4.7H_2O$ | 61.6±1.1 | 60.1 | 10.48 | 16.26 | 16.03 |
| $ZnSO_4.7H_2O$ | 73.6±2.4 | 71.1 | 11.13 | 20.98 | 20.11 |
| 8.112 keV | | | | | |
| Al | 49.5±0.6 | 49.1 | | | |
| $MgSO_4.7H_2O$ | 24.1±0.7 | 23.1 | 10.07 | 9.88 | 9.82 |
| $CaSO_4.2H_2O$ | 61.3±2.3 | 60.8 | 13.76 | 15.08 | 14.73 |
| $MnSO_4.H_2O$ | 96.2±0.8 | 107.1 | 15.99 | 19.33 | 18.98 |
| $FeSO_4.7H_2O$ | 70.5±0.4 | 76.5 | 14.42 | 16.26 | 16.03 |
| $ZnSO_4.7H_2O$ | 30.2±0.4 | 29.7 | 10.87 | 20.98 | 20.11 |

This PDF is available for free download from a site hosted by Medknow Publications (www.medknow.com)

[Downloaded free from http://www.jmp.org.in on Friday, July 29, 2011, IP: 130.14.254.25] || Click here to download free Android application for t cosmic, telluric (earth's crust) of the internal origin. Additional radiation exposure is also obtained from medical exposures, industrial accidents etc. It has been generally considered that any dose of ionizing radiation is detrimental to the living organisms, however low the radiation dose may be. Even a single hit to the target cellular DNA can cause cancer. The much relied upon 'Linear No Threshold' (LNT) hypothesis does not have convincing experimental evidence regarding deleterious effects at very low-dose and low-dose rates. The deleterious effects have been inferred theoretically by extrapolating the known effects of high dose radiation to low-dose range to the biological systems. There is no doubt that ionizing radiations at absorbed doses of around 0.5 Sv or more given on a single occasion, are carcinogenic. Radiological protection regulations and practices are based on the recommendations of the International Commission on Radiological Protection (ICRP) which assumes that at low-doses there is a linear relationship between dose and the risk (cancer incidence), all the way down to zero (LNT model).[1] However, recent reports suggest that low-radiation dose (<50cGy) is not only harmful but is beneficial to the living system.[2,3] The epidemiological data available from a vast population living in high-dose background area (US, China, India, Brazil), the occupational workers engaged in the nuclear submarines, radiation associated industries, the hospital radiation clinics, the surviving Japanese population from the atomic bomb exposure to low-dose ionizing radiations indicate the reduction in the cancer mortality rate as compared to the control population. The beneficial effects of exposure to low-dose ionizing radiation are also obtained from the animal studies.[4,5] This phenomenon of beneficial effects of radiation at low-dose is known as 'radiation hormesis'. Radiological evidence for hormesis is based on radio-adaptive responses. The LNT hypothesis ignores the fundamentals of normal cellular and DNA repair processes, immune reactions, antioxidant defense and the role of apoptosis in the biological systems. Generally, damaging or lethal cellular effects are observed at high radiation doses, while cellular stimulating effects are observed at low-dose short-term exposures in the range of 0.01-0.50 Gy. Recent reports, both *in vitro* and *in vivo*, suggest that antioxidant defense system [reduced glutathione, superoxide dismutase, glutathione peroxidase, glutathione reductase, catalase[6] and RNA expression of g-GCS (glutamyl cysteine synthetase)] and the levels of immune cells ($CD_4$, $CD_8$ cells and natural killer) are enhanced. The increase in free radical generation including lipid peroxidation due to oxidative stress is known to regulate cellular redox-status mainly through phosphorylation of various serine/threonine Mitogen Activated Protein Kinases (MAPK). These kinases further activate different redox-sensitive transcription factors like Nuclear Factor Kappa-B (NF-κB) and Activating Protein-1 (AP-1). The activation of these transcription factors further results in expression of various proteins involved in antioxidant defense system, immune system, DNA repair and apoptosis. Recently, differential gene expression have been reported to be triggered by g-ray doses of 10 cGy and less in human myeloid leukemic cells in p53 regulated genes such as cyclin dependent kinase (CDKNIA), growth arrest and DNA damage response protein (GADD 45A) and murine double minute-2 protein (MDM-2).[7,8] The authors have recently observed that whole body-irradiation of mice in a narrow dose range of 10-50 cGy stimulates some of the above parameters, to variable extents in different organs. Low level ionizing radiations (LLIR) thus may be essential trace energy of life, analogous to essential trace elements like Se, Zn, I, Fe etc. Thus, low-dose whole body exposure to ionizing radiation may prove to be helpful in curing/preventing some of the cancers like chronic lymphocytic leukemia, Non-Hodgkin lymphoma and reactive oxygen species (ROS)-related diseases like Alzheimer and Parkinson diseases. Clinical trials for treating the aforesaid diseases have been initiated by different groups in USA and France. This is a step forward towards a better understanding of how low-dose ionizing radiation modulates the living cells and the organisms. Such studies and data will provide a more reliable judgement on how to interpret the present radiological evidence on the basis of radiation hormesis. An update on the above mentioned low-dose biological mechanisms will be presented.

**References**

1. International Commission on Radiological Protection. 1990 Recommendations of the International Commission on Radiological Protection. ICRP publication 60. Pergamon Press: Oxford; 1991.
2. Ina Y, Tanooka H, Yamada T, *et al.* Suppression of thymic lymphoma induction by life-long-low-dose-rate irradiation accompanied by immune activation in C57BL/6 mice. Radiat Res 2005;163:153-8.
3. Kojima S, Nakayama K, Ishida H. Low-dose gamma-rays activate immune functions via induction of glutathione and delay tumor growth. J Radiat Res 2004;45:33-9.
4. Feinendegen LE. Evidence for beneficial low level radiation effects and radiation hormesis. Br J Radiol 2005;78:3-7.
5. Tiku AB, Kale RK. Adaptive response and split-dose effect of radiation on the survival of mice. J Biosci 2004;29:111-7.
6. Avti PK, Pathak CM, Kumar S, *et al.* Low dose gamma-irradiation differentially modulates antioxidant defense in liver and lungs of Balb/c mice. Int J Radiat Biol 2005;81:901-10.
7. Amundson SA, Lee RA, Koch-Paiz CA, *et al.* Differential responses of stress genes to low-dose rate γ-irradiation. Mol Cancer Res 2003;1:445-52.
8. Lee WJ, Majumder ZR, Jeoung D 3rd, *et al.* Organ-specific gene expression in C57BL/6 mice after exposure to low-dose radiation. Radiat Res 2006;165:562-9.

This PDF is available for free download from a site hosted by Medknow Publications (www.medknow.com).

**P-41**

**Calibration of Ir-192 HDR brachytherapy source using well type and 0.6 CC farmer ionization chambers: A comparative study**

Atanu Kumar, DK Ray. *Department of Radiotherapy, Chittaranjan National Cancer Institute, 37, S.P. Mukherjee Road, Kolkata - 700 026, India*

**Introduction:** High Dose Rate (HDR) remote after loading brachythrapy for intracavitary, interstitial and surface mould applications has gained much popularity due to certain advantage over Low Dose Rate brachytherapy. Field calibration of HDR Ir-192 sources are necessary since the manufacturer's source specifications can not be accepted without verification and to ensure traceability to national standards. Calibration of HDR sources are fundamentally based on In Air measurements of exposure or air kerma. In the present study the Reference Air Kerma Rate (RAKR) of Ir-192 HDR source was measured by well chamber supplied with RAKR of Ir-192 source calibration factor and 0.6 cc Farmer ionization chamber having C0-60 source air kerma calibration factor and their intercomparision has been presented.

**Materials and Methods:** 0.6 cc Farmer ionization chamber with PTW UNIDOS Dosimeter was used for RAKR measurement in a specially made Perspex Jig for in - air measurement. It has arrangement to keep the chamber at 10 cm source to chamber distance in between two parallel source channels. The chamber has air kerma calibration factor ($N_{k,air}$) for Co-60 and buildup cap for Co-60. Therefore average value of with and without buildup cap measurement was taken to derive RAKR of Ir-192. The measurement was taken at maximum response position and RAKR at 1 meter was calculated using following formula:

$$RAKR\ (0.6\ cc\ chamber) = M_R \times K_{TP} \times N_{k,air} \times I_{invcorrec}$$

Where, $I_{invcorrec}$ = Inverse square correction.

In next set of measurement we used well chamber with PTW UNIDOS E dosimeter supplied by Nucletron with proper insert specifically designed for it. The measurement was taken at the position of maximum sensitivity in the chamber at 6.1 cm from chamber bottom. This chamber has RAKR calibration factor for Ir-192 supplied by BARC against 1000cc

Table 1: RAKR measured using well chamber and 0.6 cc chamber

| *Chamber* | *RAKRmGy h⁻¹ at 1 m* | | *% deviation* |
|---|---|---|---|
| | *measured* | *Source certificate* | *from source certificate* |
| Well chamber | 39.995 | 40.59 | 1.47 |
| 0.6 cc chamber | 39.83 | 40.59 | 1.87 |

[Downloaded free from http://www.jmp.org.in on Friday, July 29, 2011, IP: 130.14.254.25] || Click here to download free Android application for t graphite reference chamber. RAKR was calculated by using following formula:

RAKR (well chamber) = $M_R$ X $K_{TP}$ X $N_{RAKR}$

Both set of measurements were done in scatter free medium.

**Result:** The measured values of RAKR of the Ir-192 source by well chamber and 0.6 cc ionization chamber are shown in Table 1. The % deviation of measured RAKR from source certificate for well chamber and 0.6 cc ionization chamber were 1.47% and 1.87% respectively and the % difference between measured RKAR from well chamber and 0.6 cc chambers was 0.4% only.

**Conclusion:** Good agreement was found between the measured RAKR of Ir-192 HDR brachytherapy source by well chamber having RAKR calibration factor for Ir-192 and 0.6 cc Farmer Ionization chamber having air kerma calibration factor for Co-60. Therefore 0.6 cc ionization chamber may also be used for Ir-192 source calibration using proper positioning device to fix channels and chamber with proper source to chamber distance by those centres that do not have well chamber. However the complexity and time involved for source calibration can be significantly reduced with the use of well ionization chamber specifically designed for this purpose.

### Reference

1. High Dose Rate Brachytherapy: A Text Book by Subir Nag.

### P-42

### Small field output factors of indigenously developed Bhabhatron and Theratron equinox Telecobalt unit

Rajesh Kumar, Mahua Basu, SD Sharma, DC Kar**, RA Kinhikar*, Rituraj Upreti*, Reena PH***, S Kannan. *Radiological Physics and Advisory Division, Bhabha Atomic Research Centre, CT and CRS Building Anushaktinagar, Mumbai - 400 094, *Department of Medical Physics, Tata Memorial Hospital, Mumbai - 400 012, **Division of Remote Handling and Robotics, Bhabha Atomic Research Centre, Mumbai - 400 094, ***Department of Radiation Oncology ACTREC, Kharghar, Navi Mumbai, India*

E-mail: raj_resh@rediffmail.com

**Introduction:** Output factor is defined as the ratio of dose in water at a depth of maximum dose ($d_m$) in central axis for a specific field size with respect to reference field size of 10x10 cm$^2$. Output factor measured at a point on central axis for a high energy photon beam varies with the collimator setting due to various reasons; one of them being scattering of photon beam by structures in the head, especially in the collimator and also due to variation in the collimator head design when two different units are considered. Bhabhatron is an indigenously developed telecobalt unit by BARC and Theratron Equonox-80 is a new model of telecobalt unit from the MDS Nordion, Canada. These units can produce small field size less than 1x1 cm$^2$, which was not possible in other available model of telecobalt units. In this study, small field out factor of both Bhabhatron and Theratron Equinox-80 telecobalt machines has been compared with changes in field size using TLD micro cube, miniature ion chamber and Gafchromic EBT film.

**Materials and Methods:** All the measurements were performed on indigenously developed Bhabhatron and Theratron Equinox-80. Secondary collimator of Equinox is multivane type made up of the tungsten. Two pair of trimmer bar along with a motorized wedge is provided beneath the lower end of secondary collimator. Secondary collimator of Bhabhatron contains two pairs of collimating jaws made of natural uranium. Two pair of trimmer bars made of the natural uranium is also provided beneath the lower jaws. Measurements of output factor were carried out for field sizes in the range of 1x1 to 20x20 cm$^2$. EBT film samples of size 2.5x2.5 cm$^2$ were irradiated for different field sizes for equal durations. The duration of irradiation was selected to deliver 4 Gy from 10x10 cm$^2$ field at $d_m$ in SSD setup (SSD= 80 cm) in a full scatter solid phantom. Exposed EBT film was analysed using flatbed document scanner (Epson, UK) with ImageJ (National Institute of Health, USA) software. Similar measurements were carried out using TLD micro cubes of size 1x1x1 mm$^3$ and miniature ion chamber CC01 (Scandritronix Wellhofer, Sweden). TLD measurements were carried out using a locally fabricated perspex phantom plate of size 30x30x0.1 cm$^3$ with a groove of 2x2 mm$^2$ at the center, to hold the four TLD cubes together. The duration of irradiation was selected to deliver 1 Gy from 10x10 cm$^2$ field at $d_m$ in SSD setup (SSD= 80 cm) in a full scatter solid phantom. Results of all four TLD cubes were averaged for better statistical result. Individual calibration factor was measured for each TLD cube in the same perspex plate, used for the output factor measurements. Exposed TLDs were evaluated using UL-230 TLD reader (Rexon USA) under nitrogen flushing.

**Results and Discussions:** Output factor measured using EBT films for both bhabhatron and equinox is given in Figure 1. It is observed from this curve that output factor of bhabhatron is significantly lower in comparison to equinox's output factor for 1x1 to 3x3 cm$^2$ field size. The variation in the output factor may be attributed to the design difference of the collimator assembly of these two units. The suitability of different detector for measuring small field output factor has been studied for theratron equinox unit and the results is shown in Figure 2. The variation observed between EBT film with respect to ion chamber and TLD reading for 1x1 cm$^2$ is of the order of 5.4% and 3.0% respectively. As the field size increases, the difference in output factor measured using these detectors reduces to 2-3%, which is within measurement uncertainty. At field size 1x1 cm$^2$, higher variation

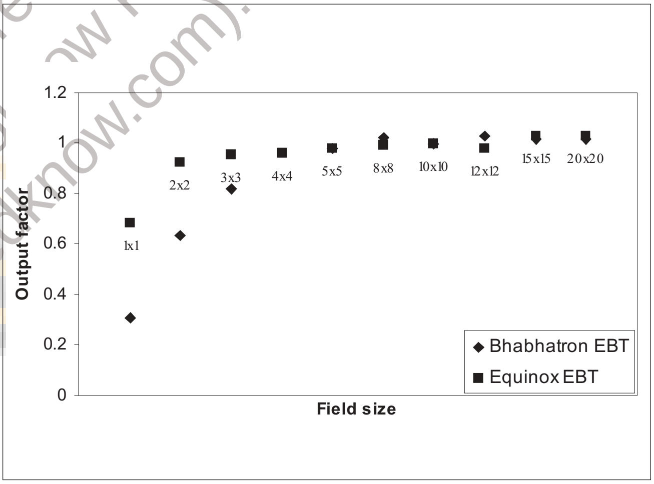

**Figure 1:** Output factor measurement using EBT film for bhabhtron and theratron equinox telecobalt unit

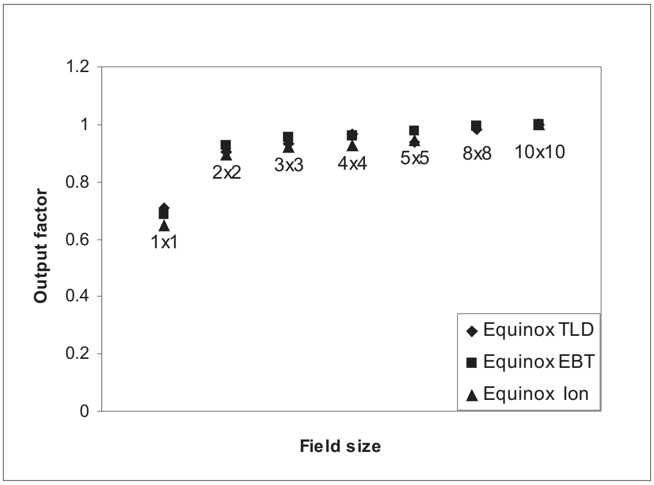

**Figure 2:** Output factor measured using TLD micro cubes, EBT films and ion chamber for theratron equinox telecobalt unit

This PDF is available for free download from a site hosted by Medknow Publications (www.medknow.com).

[Downloaded free from http://www.jmp.org.in on Friday, July 29, 2011, IP: 130.14.254.25] || Click here to download free Android application for t between EBT and ion chamber, might be due to the lack of lateral disequilibrium in cc01 ion chamber measurement, while this is not the issue in case of EBT film. TLD micro cube reading was not very consistent during our study. This study reveals that EBT film is one of the suitable detectors for small field output factor measurement. Conclusions: Small field output factor of bhabhatron and theratron equinox - 80 was compared. EBT film with flatbed document scanner is one of the suitable detectors for small field output factor measurement.

**P-43**

**Performance characteristics of an indigenously developed 6 MV Siddharth medical linear accelerator**

PK Dash Sharma, G Sahani, Rajesh Kumar*, RK Chaturvedi, Sarad T Chavan**, Amol Bhagwat**, DN Sharma***, SP Agarwal. *Radiological Safety Division, Atomic Energy Regulatory Board, Niyamak Bhavan, Anushaktinagar, Mumbai - 400 094, *Radiological Physics and Advisory Division, Bhabha Atomic Research Centre, CT and CRS Building, Anushaktinagar, Mumbai - 400 094, **SAMEER, IIT Campus, Powai, Mumbai - 400 076, ***Radiation Safety Systems Division, Bhabha Atomic Research Centre, Trombay, Mumbai - 400 085, India*

Introduction: The increase in number of Medical Linear Accelerators (Linac) in the country during last few years in comparison to Telecobalt units shows that the Linac has become the first unit of choice due to various advantages such as reduced penumbra, better skin sparing effect, dose rate, percentage depth dose (PDD) etc. over telegamma therapy unit. However, almost all the Linacs installed in the country are imported ones. Considering the large number of requirement of teletherapy unit in India, a 6 MV Medical Linac named SIDDHARTH has been developed indigenously by Society for Applied Microwave Engineering and Electronics Research (SAMEER), Mumbai. The first unit of SIDDHARTH is already installed at one of the Radiotherapy Centres in the country. To evaluate its suitability for clinical use comprehensive performance tests were carried out. This paper describes the performance characteristics of SIDDHARTH. Materials and Methods: SIDDHARTH is a 6MV single photon beam energy, computer controlled Linac with a provision to set the dose rate between 50 MU/min to 240 MU/min. This is an rotational teletherapy machine with target to isocentre distance 100 cm. Field size (FS) can vary from 0x0 cm$^2$ to 35x35 cm$^2$ (40x40 cm$^2$ with clipped corner). Electrical, mechanical, dosimetric and radiation safety related evaluations of SIDDHARTH unit were carried out as per International Electrotechnical Commission standards (IEC 60601-2-1:1998) and Quality Assurance protocol of RP and AD, BARC (RPAD/BARC/ACC/97). 3D Radiation Field Analyzer (Scanditronix Wellhofer, Sweden) was used for measurement of flatness, symmetry, penumbra, PDD etc. for various field sizes. Therapy Verification Films (Kodak, USA) were used for optical and radiation field congruence, radiation isocentre with respect to collimator, gantry and couch rotation. The radiograms of the head were taken using diagnostic X-ray films to find out the hotspots. Radiation leakage through the head and collimator transmission measurements were carried out using pocket dosimeters (Digirad, BARC), 30cc square ionization chamber (Nuromax) and TLDs (CaSO4: Dy). Monitor chamber characteristics of SHIDDHARTH were measured using 0.65cc farmer type ion chamber (Scanditronix Wellhofer, Seweden). Results and Discussion: All the electrical safety interlocks, which includes emergency switches, door interlock, end course cut-off, wedge filter interlock etc. were checked and found satisfactory. Unlike the imported units, it gives aural indication in the treatment room for ready state and during treatment with different tones as specified in IEC standard. The mechanical parameters, which include mechanical isocentre, field size accuracy, accuracy of various scales etc. were checked and found satisfactory. The results of dosimetric and safety parameters including some of the mechanical parameters are shown in the Table 1. Conclusion: A thorough performance evaluation of the SIDDHARTH Medical Linear Accelerator was carried out and all the test parameters were found satisfactory. The test reports were scrutinized by the Regulatory Body and the unit was type approved for clinical use.

Table 1: Some of the results pertaining to mechanical, dosimetric and safety parameters

| *Test parameters specified* | *Tolerance* | *Maximum deviation Observed* |
|---|---|---|
| Shift in mechanical Isocentre | ≤ 2 mm | 2 mm |
| Optical field size accuracy (FS ≤ 10x10 cm$^2$) | ≤ 1 mm | 1 mm |
| Optical field size accuracy (FS > 10x10 cm$^2$) | ≤ 2 mm | 1 mm |
| Quality index of Photon energy | 0.676 ± 0.009 | 0.672 |
| Depth of dose maximum (dm) | 1.5 ± 0.2 cm | 1.46 cm |
| Percentage depth dose at 10 cm depth (FS = 10x10 cm$^2$) | 67.1% ± 1.5% | 66.3% |
| Optical and radiation field congruence | ≤ 2 mm | 2 mm |
| Photon Beam Flatness (FS = 5x5 cm$^2$) | ≤ 3% | 1.1% |
| Photon Beam Flatness (FS = 10x10 cm$^2$) | ≤ 3% | 2.0% |
| Photon Beam Flatness (FS = 30x30 cm$^2$) | ≤ 3% | 3.3% |
| Photon Beam Symmetry (FS = 5x5 cm$^2$) | ≤ 3% | 1.1% |
| Photon Beam Symmetry (FS = 10x10 cm$^2$) | ≤ 3% | 0.3% |
| Photon Beam Symmetry (FS = 30x30 cm$^2$) | ≤ 3% | 0.2% |
| Photon Beam Penumbra (FS = 10x10 cm$^2$) | | – 7.6 mm |
| Reproducibility of Dose Monitors | ≤ 0. 5% | 0.38% |
| Linearity of Dose Monitors | ≤ 1% | 0.53% |
| Leakage radiation outside the useful beam in patient plane | ≤ 0.2% | 0.2% |
| Leakage radiation at 1 meter from the electron path other than patient plane | ≤ 0.5% | 0.07% |
| Leakage through Beam Limiting Devices | ≤ 0.5% | 0.5% |

**P-44**

**Comparative study on performace of an indigenously developed medical linearaccelerator with imported medical linearaccelerators**

G Sahani, PK Dash Sharma, Rajesh Kumar*, RK Chaturvedi, Kanta Chhokra, DN Sharma**, SP Agarwal. *Radiological Safety Division, Atomic Energy Regulatory Board, Niyamak Bhavan, Anushaktinagar Mumbai- 400 094, *Radiological Physics and Advisory Division, Bhabha Atomic Research Centre, CT and CRS Building, Anushaktinagar Mumbai- 400 094, **Radiation Safety Systems Division, Bhabha Atomic Research Centre, Trombay, Mumbai - 400 085, India*

**Introduction:** The photon beam energy most often used for external beam radiation therapy is 6 MV because of its suitable beam characteristics for majority of the tumour sites. 6 MV photon beam is also considered as suitable energy for the special techniques such as Stereotactic Radiosurgery (SRS), Stereotactic Radiotherapy (SRT) etc. Apart from the known advantages to compete with the cost of telecobalt unit and the potential market in the developing countries, the manufacturers of Medical Linear Accelerators (Linac) have designed 6 MV single photon energy Linacs with and without electron beams, which is quite affordable for many of the user institutions in India. In view of the above mentioned advantages and the requirements of large number of teletherapy units in the country, SIDDHARTH, a 6 MV Medical Linear Accelerator has been developed indigenously by Society for Applied Microwave Engineering and Electronics Research (SAMEER), Mumbai. This Paper describes the comparative study of indigenously developed S IDDHARTH 6 MV Linac with imported 6 MV Medical Linear Accelerators.

**Materials and Methods:** Comprehensive performance evaluation, which includes electrical, mechanical, dosimetric and radiation safety parameters, of Clinac DBX (Varian), Primus Essential (Siemens) and

This PDF is available for free download from a site hosted by Medknow Publications (www.medknow.com).

[Downloaded free from http://www.jmp.org.in on Friday, July 29, 2011, IP: 130.14.254.25] || Click here to download free Android application for t

Table 1: Comparison of dosimetric parameters of Clinac DBX, Primus Essential and Siddharth

| Parameters | Specified tolerance | Clinac DBX | Primus essential | Siddharth |
|---|---|---|---|---|
| Photon Beam Flatness (FS = 10 x 10 $cm^2$) | ≤ 3% | 0.9% | 1.06% | 2.0% |
| Photon Beam Symmetry (FS = 10 x 10 $cm^2$) | ≤ 3% | 0.9% | 0.7% | 0.3% |
| Photon Beam Penumbra (FS = 10 x 10 $cm^2$ at 100 cm TSD and at a depth of 10 cm) | – | 9.9 mm | 8 mm | 7.6 mm |
| Quality Index of Photon Energy (6 MV) | 0.676 ± 0.009 | 0.671 | 0.672 | 0.672 |
| Depth of dose maximum ($d_m$) | 1.5 ± 0.2 cm | 1.5 cm | 1.5 cm | 1.46 cm |
| Percentage depth dose at 10 cm depth (FS 10 x 10 $cm^2$ at 100 cm TSD) | 67.1% ± 1.5% | 66.6% | 65.9% | 66.3% |
| Reproducibility of Dose Monitors | ≤0.5% | 0.037% | 0.039% | 0.38% |
| Linearity of dose Monitors | ≤1% | 0.75% | 0.044% | 0.53% |
| Leakage radiation at 1 meter in patient plane | ≤0.2% | 0.008% | 0.035% | 0.2% |
| Leakage radiation at 1 meter in other than patient plane | ≤0.5% | 0.037% | 0.048% | 0.07% |

SIDDHARTH (SAMEER) was carried out, as all of them are Linacs of single energy 6 MV photon beam. Performance evaluations of these units were carried out as per Quality Assurance protocol of RPandAD, BARC (RPAD/BARC/ACC/97). For measurement of flatness, symmetry, penumbra, PDD etc. for various field sizes 3D Radiation Field Analyzer was used. Therapy Verification Films were used for optical and radiation field congruence, radiation isocentre with respect to collimator, gantry and couch rotation. Radiation leakage through the head and collimator transmission measurements were carried out using pocket dosimeters. Monitor chamber characteristics of these units were measured using farmer type ion chamber.

**Results and Discussion:** The electrical and mechanical test results of Clinac DBX, Primus Essential and SIDDHARTH were found in conformity with the national standards. The dosimetric and radiation safety parameters of these units are shown in Table 1. The values shown in the table are the maximum deviations observed during the measurements. From the results shown the table, it is evident that the dosimetric and radiation safety related parameters of the indigenously developed Linac are comparable with the imported accelerators except the beam flatness and leakage radiation in the patient plane. However, all the dosimetric and safety related parameters are well within the prescribed tolerance limits. As per the information provided by SAMEER, like imported units the indigenous treatment planning system is going to be networked with SIDDHARTH in future.

**Conclusion:** The performance test results of the indigenously developed Linac SIDDHARTH are quite encouraging in comparison to imported ones and can be used for clinical purposes. However, there is further scope of improvements such as provision for 3DCRT, SRS, SRT, IMRT etc. like imported units.

This PDF is available for free download from a site hosted by Medknow Publications (www.medknow.com).

**P-45**

**BMD: Corrections in T and Z scores for Indian subjects**

B Santhanaraj, Emmanuel*, Venkatasai**, AV Lakshmanan***. *Sahrdaya Engg. College-Trichur, *Bharat Scans, **SRMC and RI, ***Cancer Institute, Chennai, India*
Correspondence: Dr. B. Santhanaraj, 114 Somasundara Layout, Udumalpet Road, Pollachi - 641002, Tamilnadu, India
E-mail: bsrindia@yahoo.com

Bone mineral density (BMD) plays an important role in the prediction and assessment of osteoporesis and osteopenia. The bone fracture risk can also be determined by BMD. Dual Energy X-ray absorptiometry (DeXA) provides complete information of the BMD at regional as well as wholebody level. T scores and the age matched Z-scores are the two indices used for the estimation of fracture risk as well as the diagnosis of osteopenia and osteoporesis. The two indices are variable with resopect to grace, ethinicity, life style etc. Hence the values are to be set at the regional (geographical) level. The peak bone mass for Indian subjects are not yet seems to be estimated. The T and Z scores are not available for Indian Subjects in any of the commercially available softwares with the vendors of DEXA or QUS Densitometers. The standard practice in our country is to refer to the US female value for Indian female subjects and German values for Male subjects as they are assumed to be closely correlated to Indian Subjects. However our studies on both DeXA and Quantitative Ultrasonometry (QUS) shows a significant deviation from these values. Nearly 150 cases were studeied which contains almost equal number of both sexes in the age group of 20 to 70 with maximum sunbjects at the age range of 30-50 years. The selection of maximum subjects were according to Indian reference Man. Our result shows that the peak bone mass was not achieved at early 20s as with western population in almost all cases. The T scores and Z scores show significant deviation from the western values for assessment of osteoporesis. Corrections were made to the T and Z scores and new set of data for Indian subjects are suggested. The BMD value measured by QUS at oscalsis is comparable with whole body BMD value measured with DeXA among significant population.

**P-46**

**Radiological safety guidelines in diagnostic X-ray examinations**

RK Chaturvedi, VK Shirva, SP Agarwal. *Radiological Safety Division Atomic Energy Regulatory Board*, Mumbai, India
E-mail: chaturvedirk2003@yahoo.co.in

**Introduction:** In the past 100 years, diagnostic radiology has evolved from the original crude practice to advanced technique that form an essential tool for all branches and specialties of medicine. Benefit to patients from medical diagnostic X-ray examinations has been established beyond doubt. Modern diagnostic radiology assures faster, more precise diagnosis and enables monitoring of a large proportion of diseases. It has been estimated that in about one half of cases, radiological procedures (plain film radiography, fluoroscopy, computed tomography) have a substantial impact on the speed of diagnosis and in a large fraction of cases they are of decisive importance. This paper is designed to provide basic information on the doses from various diagnostic X-ray examinations, the magnitude and type of risk, as well as answers to commonly asked questions (e.g., radiation and pregnancy).

**Typical doses from various medical diagnostic procedures:** All living organisms on this planet, including humans, are exposed to natural background to an average yearly effective dose of about 2.5 mSv. Various diagnostic radiology procedures cover a wide dose range based upon the procedure. Typical values of effective doses for some procedures are presented in Table 1.

**Materials and Methods:** Managing radiation doses in diagnostic X-ray examinations: There are several ways to reduce the risks to very, very low levels while obtaining very beneficial health effects of radiological procedures. So high ratio of benefit vs. radiological risk

[Downloaded free from http://www.jmp.org.in on Friday, July 29, 2011, IP: 130.14.254.25] || Click here to download free Android application for t

Table 1: Doses from medical diagnostic procedures:

| *Diagnostic X-ray examinations* | *Typical effective dose (mSv)* |
|---|---|
| Limbs and joints (except hip) | < 0.01 |
| Chest (single PA film) | 0.02 |
| Skull | 0.07 |
| Thoracic spine | 0.7 |
| Lumber spine | 1.3 |
| Hip | 0.3 |
| Pelvis | 0.7 |
| Abdomen | 1.0 |
| Barium swallow | 1.5 |
| Barium Meal | 3 |
| Barium enema | 7 |
| CT Head | 2.3 |
| CT chest | 8 |
| CT abdomen or pelvis | 10 |

depends to large extent on a good methodology of procedures and high quality of their performances. Therefore quality assurance and quality control in diagnostic radiology play a fundamental role in the provision of appropriate, sound radiological protection of the patient. Special consideration of children and pregnant women: Since both the fetus and children are thought to be more radiosensitive than adults. So, before a diagnostic procedure is performed it should be determined whether a patient is pregnant, whether the fetus is in the primary radiation area and whether the procedure is relatively high dose (e.g., barium enema or pelvic CT scan). If an examination is typically at the high end of the diagnostic dose range and the fetus is in or near the radiation beam, care should be taken to minimize the dose to the fetus while still making the diagnosis. This can be done by tailoring the examination and examining each radiograph as it is taken until the diagnosis is achieved and then terminating the procedure.

**Regulatory Control for Medical X-ray Units:** The Atomic Energy Regulatory Board (AERB) has been constituted on November 15, 1983 by the Government of India to carry out certain regulatory and safety functions envisaged under section 16, 17 and 23 of the Atomic Energy Act of 1962. The regulatory control over medical X-ray units is exercised by AERB based on the following:

1. The Atomic Energy (Radiation Protection) Rules, G.S.R. 303,2004.
2. The Radiation Surveillance procedures for Medical Applications of Radiation, (GSR 388), 1989.
3. The safety code for Medical Diagnostic X-ray equipment and Installations, AERB/SC/MED-2 (Rev.1), 2001.
4. AERB safety manual: Atlas of reference plans for Medical, Diagnostic X-ray Installations, (1986).

**Regulatory Control:** Atomic Energy Regulatory Board (AERB) permits only type-approved devices marketed in India. AERB insists manufacturers of medical diagnostic X-ray units to installation their X- ray units at AERB approved layout plans and procure tools and accessories including quality assurance kit to carry out quality assurance of the diagnostic X-ray units at the user's premises and encourage the users for registration of diagnostic X-ray units with AERB.

**Conclusions:** The objective of minimizing the radiation risk from diagnostic procedures is achieved by avoiding unnecessary (unjustified) examinations and optimizing the applied procedures both from the standpoint of diagnostic quality and of reduction of the excessive doses to patients.

**P-47**

### Calculation of energy distribution and mean energy of $^{169}Y_b$ high dose rate brachytherapy source

S Vandana, S Sahoo, A Philomina, T Palani Selvam, SD Sharma, S Kannan. *Radiological Physics and Advisory Division, Bhabha Atomic Research Centre, Mumbai, India*

E-mail: bikaiv_barc@rediffmail.com

**Introduction:** Due to the high specific activity (370 GBq mm$^{-3}$) of $^{169}$Yb, this radionuclide is a possible replacement of the $^{192}$Ir source in high dose-rate (HDR) applications. There is published dosimetry data in accordance with AAPM TG-43U1 formalism using MCNP5 code involving detailed transport of electrons and photons. In the present study, we have used MCNP version 3.1 (which ignores transport of electrons) to calculate energy distribution and mean energy of HDR $^{169}$Yb brachytherapy source (model 4140). Before doing this we verified MCNP5-based dose parameters.

**Materials and Methods:** Monte Carlo calculations: The dose parameters such as air-kerma strength per mCi, $S_k$/mCi, dose rate constant, $\Lambda$, radial dose function, g (r) and anisotropy function, F(r, $\theta$) parameters have been calculated and compared with the published data. The air-kerma weighted and fluence-weighted mean energies, $E_{ak}$ and $E_{fl}$, respectively, were calculated for both in free space and in water medium using the following relations:

$$E_{ak} = \frac{\int E^2.\phi(E).[\mu_{en}(E)/\rho].dE}{\int E.\phi(E).[\mu_{en}(E)/\rho].dE} \quad (1)$$

$$E_{fl} = \frac{\int E\phi(E).dE}{\int \phi(E).dE} \quad (2)$$

Table 1: Fluence and air-kerma weighted mean energies calculated in water for point and real $^{169}$Yb sources

| *Distance in water (cm)* | *Mean energy (keV)* | | | |
|---|---|---|---|---|
| | *Point source* | | *Real source* | |
| | *Fluence-weighted,* Ç$_{fl}$ | *Air-kerma weighted,* Ç$_{ak}$ | *Fluence-weighted,* Ç$_{fl}$ | *Air-kerma weighted,* Ç$_{ak}$ |
| 1 | 85 | 115 | 101 | 137 |
| 2 | 80.5 | 105 | 95.8 | 127.8 |
| 3 | 77.3 | 98.3 | 91.8 | 120.4 |
| 4 | 75.4 | 93.8 | 89.1 | 115 |
| 5 | 73.1 | 89.2 | 86.7 | 111 |
| 6 | 72.8 | 87.8 | 85.1 | 107 |
| 7 | 72.0 | 85.7 | 83.5 | 105 |
| 8 | 71.2 | 84.8 | 83.1 | 104 |
| 9 | 72.0 | 84.6 | 81.5 | 102 |
| 10 | 71.5 | 83.1 | 81.7 | 102 |

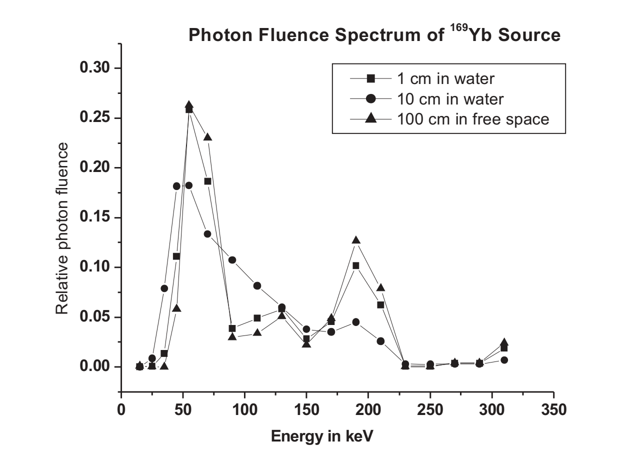

**Figure 1:** Normalized fluence spectra obtained in water phantom for radial distances 1 and 10 cm along the transverse axis of the $^{169}$Yb HDR source. Also shown is the spectrum obtained in free space for comparison purpose

This PDF is available for free download from a site hosted by Medknow Publications (www.medknow.com).

[Downloaded free from http://www.jmp.org.in on Friday, July 29, 2011, IP: 130.14.254.25] || Click here to download free Android application for t where E is the kinetic energy of photon in MeV, Φ (E) is the differential photon fluence spectrum at E about dE and $\mu_{en}/\rho$ is mass energy absorption coefficient of air. In water simulation, point and line $^{169}$Yb source were centered at the origin in a spherical water phantom of radius 15 cm to determine $E_{fl}$ and $E_{ak}$. The fluence spectrum was scored at every 10 keV interval.

**Results and Conclusion:** The value of $S_k$/mCi, was calculated to be 1.09 x $10^{-4}$ ± 0.05 cGy $h^{-1}$ $cm^2$ $mCi^{-1}$. The absolute dose rate in water at 1 cm on the transverse axis was determined to be 1.30 ± 0.09 cGy $h^{-1}$ $mCi^{-1}$ and the dose rate constant, L, was calculated to be 1.20 ± 0.10 cGy $h^{-1}$ $U^{-1}$. The calculated values of $E_{fl}$ and $E_{ak}$ in free space for the actual source are 109 ± 0.06% keV and 150 ± 0.08% keV, respectively. The corresponding values for point source are 92.7 ± 0.02% keV and 130 ± 0.05% keV. Table 1 presents the calculated mean energies in water for both point and line source geometry of $^{169}$Yb model HDR4140 brachytherapy source along the transverse axis for distances of 1 - 10 cm. The statistical uncertainty associated with the mean energies is within 1%. The line source mean energy values are higher than the point source due to absorption of low energy photons by the source and encapsulation materials. Fig 1 presents normalized photon fluence spectra scored in water for distances 1 and 10 cm and the spectrum obtained in free space at 100 cm along transverse axis of the source. The spectral details may be useful for evaluating detector response function. This study indicates that, MCNP radiation transport code version 3.1 can safely be adopted for the calculation of $^{169}$Yb dosimetric parameters.

### P-48

### Fabrication and dosimetric evaluation of customized electron beam cutouts

A Srinivasan, Finlay Godson Henry, John Subhashini, Ravindran B Paul. *Department of Radiotherapy, Christian Medical College, Vellore - 632 004, India*

**Introduction:** High-energy electrons have been used in radiation therapy since the early 1950s. Shaping the electron beam according to the area to be treated is necessary since only square shaped applicators are available and this is mostly carried out using lead strips. These strips are either placed on the patient's body or at the distal end of the electron applicator. The disadvantages are the difficulty in reproducing the beam shape during the treatment and in obtaining irregular beam shapes. Hence customized beam-shaping blocks for electron treatment using low melting alloy (LMA) were fabricated and their beam characteristics such as variation in output and beam transmission through the block were investigated.

**Materials and Methods:** LMA, known as cerrobend consists of Bismuth-50%, Lead-26.7%, Tin-13.3% and Cadmium-10%. Advantage of LMA is its low melting point of 70°C against 327°C of lead and is harder at room temperature. A few centimeters thickness (1-2 cm) is sufficient for most of the electron beam energies. The field shape obtained from the treatment planning system (TPS) was traced on a Styrofoam and was cut using a Styrofoam cutter. The resulting block of Styrofoam was placed over the in-house LMA mould tray with proper immobilization and the melted LMA was poured into the tray. On cooling, the LMA block was removed from the tray. The shape obtained was checked with the template of field shape from the TPS.

**Dosimetric evaluation of the customized block:** The percentage of transmission was measured for a 15x15 $cm^2$ LMA block of 1.5 cm thick using Capintec parallel plate chamber in a perspex phantom for the maximum electron energy available. Also, output for field size shaped with both the customized block and the lead strips were measured in a perspex phantom using the Capintec 0.6cc Farmer chamber at 1 cm depth for all available electron energies. For this a cutout of field size of 9x8 $cm^2$ was fabricated and fitted to the 15x15 $cm^2$ cone. The same field size was created using lead strips. The increase in output due to the insertion of the customized block as well as lead strips was studied.

**Result and Discussion:** The percentage of transmission through the customized block for the maximum energy was 4.7% and is within the permissible level of 5%. The output was found to increase by 3.03% for 9MEV and 3.7% for 12MEV respectively when the fabricated block was inserted to shape the field. The increase in output was found to be 2.23% for 9MEV and 3.03MEV for 12MEV when lead strips were used. The increase in output was found to be less for the lead strips but is unaccounted when treatment time is calculated thus resulting in an overdose by 2.23 to 3.03% depending on the energy used. But when using a customized block, this increase in output is taken into account. The added advantage is that the field shape is reproducible during the course of treatment. Moreover while using the customized block, the field center matches with the isocenter of the machine irrespective of the gantry angle. But with the lead strips, this is not possible.

**Conclusion:** A customized cerrobend shielding block for shaping the electron beam was successfully fabricated and its transmission characteristics as well the change in output was investigated. Customized field shaping is found to be superior to the lead strips in terms of reproducibility during treatment.

### P-49

### Segmental IMRT for nasopharyngeal cancer in clinical setting

Girgesh Yadav, Alok Kumar, Vinod Pandey, Gautam Mukherjee, Kalyan Bhattacharya. *AMRI Hospitals, Kolkata, India*

**Introduction:** Due to complex geometry of various target volumes and organs at risk (OAR), it is useful to apply intensity-modulated radiotherapy (IMRT) for the treatment of nasopharyngeal cancers. The number of beams, the beam orientations and the number of segments, when using a segmental MLC technique, are important parameters for the optimization process of such an IMRT plan and result in a vast amount of degrees of freedom for the optimization process. Several groups have studied optimization of beam orientation for IMRT. In most of these studies, beam orientation optimization resulted in improved dose distributions, when a small number of beams were applied. IMRT is often clinically introduced using fixed beam geometry. Seven equiangular coplanar beams are used for the IMRT Planning nasopharyngeal cancers.

**Materials and Methods:** In our institute segmental IMRT, we have step and shoot IMRT. For the segmental IMRT, the optimal fluence maps obtained by an inverse planning process are divided into a discrete number of intensity levels. By increasing the number of intensity levels, a better approximation of the optimal fluence map is obtained. For practical implementation, the total number of segments is an important parameter, Since the treatment time is closely related to the number of segments, the use of many segments will not only result in long treatment times but also in many segments with few monitor units. This might lead to unacceptable uncertainties in the treatment delivery. Potter *et al.* reported the treatment efficiency, represented total number of MLC segments, first depends on number of intensity levels and then segmentation technique used. the number of intensity levels used to obtain results close to the optimal intensity maps was 5-10. The relation between the quality of treatment plan and number of beams in combination with number of segments is important for the clinical introduction of segmented IMRT**.** We have the Treatment planning software (PLATO sunrise, Nucletron India Pvt. Ltd.) and Linear Accelerator (Elekta Medical System). Four Patients, Previously treated with conventional radiotherapy for nasopharyngeal carcinoma (T1-3) at our institute were re-planned for this study. All patients underwent for the CT simulation. Scan was taken at 3 mm slice intervals. The clinical target volume (CTV) of the lymph node containing regions, the CTV of primary target and OAR (spinal cord, brain, brainstem, optic chiasm, Parotid) were delineated by a physician. A margin of 7 mm was applied to each CTV to take into account the set-up uncertainties and organ motion, resulting in the planning target volume (PTV) of the CTV of the primary tumor and the lymph node. No margins were applied to the OAR. All the extensions were limited in the region containing the skin, where the PTV was adjusted by the hand when necessary to assure a minimal distance of 5 mm between the PTV and the skin.The prescribed dose for the PTV node region

This PDF is available for free download from a site hosted by Medknow Publications (www.medknow.com)

[Downloaded free from http://www.jmp.org.in on Friday, July 29, 2011, IP: 130.14.254.25] || Click here to download free Android application for t is 50 Gy (2Gy /fraction, five fraction in a week) and for the primary tumor it is 70 Gy (2Gy/fraction, five fraction in a week). IMRT plans were generated using the inverse treatment-planning module (ITP) of Plato. The ITP module optimizes the fluence for a fixed beam geometry to obtain a dose distribution that best fits a series of dose constraints for target maximum and minimum dose specified and for OARs only maximum dose is specified. Standard set of dose constant was determined by systematically varying the penalties, minimum and maximum dose values for this purpose we used a patient including all PTV and Oars. The starting point was to irradiate only the PTV without considering the OARs. When acceptable dose homogeneity was achieved with in the PTVs, then OARs were added to the optimization process. Increasing the penalty was stopped when the volume of the PTVs receiving 95% of the prescribed dose reduced significantly. After obtaining the standard set, it was tested on two other patients using three different beam geometries. The standard ITP sequencer for the Elekta MLC was used for the sequencing procedure**.** The optimized intensity values of each fluence map were therefore subdivided into a number of equidistant levels.In order to minimize the number of segments, 1D median filter is applied to the theoretical fluence with a width of three bixel. For each patient, the dose distribution was optimized using a different number of equiangular beams with the gantry angle of the first beam at 0. The difference between the dose received by 95% of the PTV and dose received by 5% of the PTV (D 95-5) was used as a parameter indicating the homogeneity of the dose distribution. The volume receiving less than 95% of the prescribed dose and more than 105% of the prescribed dose were used as parameters indicating underdose and overdose respectively. For the PTV of the lymph nodes the volume receiving less than 90% of the prescribed dose was also calculated.

**Result:** The Segmental IMRT technique the dose homogeneity within the target volumes improved when the total number of segments increased. Seven beams were sufficient to achieve the saturation level for dose homogeneity. The mean dose to the parotid glands depended on the beam geometry and tumor location and did not depend of the number of segments.

**Conclusion:** Seven beams are sufficient to achieve acceptable dose homogeneity within the target volumes and significant parotid sparing.

**P-50**

**Reduction of person-Sv for packing and dispatch group of RPL, BRIT**

N Swaminathan, Lalit Kumawat, SS Sachdev*. *RSSD, BARC, Trombay, Mumbai - 400 094, *RPL, BRIT, Vashi Complex, Navi Mumbai - 400 705, India*

**Introduction:** Radiopharmaceuticals Laboratory (RPL), Board of Radiation and Isotope Technology (BRIT) supplies radiopharmaceuticals, mainly ≈400 GBq of $^{99}$Mo and ≈300 GBq of $^{131}$I, are numbering about 250 packages per week. These Type-A packages were monitored for the surface dose-rate and dispatched to various hospitals for diagnosis and treatment. The procedure adopted for packing was manual, laborious and time consuming, thus results in considerable person-Sv expenditure. Modified procedure in packing, introduction of new package has resulted in the reduction personnel exposure of the staff engaged in this dispatch group. The paper discusses the details of the procedures modified and the new package inducted.

**Materials and Methods:** The vial containing dispensed radioactivity and housed in a lead container was brought to the dispatch room for packing. In the earlier practice, majority of the operations such as sealing of the lead pot (body with its lid) with monsoon tape, transfer of the same into a tin container and sealing and final packing in a cubical wooden box was carried out manually. These packages were temporarily stored in a shielded storage facility and later retrieved and loaded into the dispatch vehicle manually. On detailed study, the contributing factors for the increase in person-Sv expenditure for the dispatch staff were identified. Except sealing of the tin container, all the operations are manual, time consuming and requires presence of the staff in proximity to the source. The temporary storage after packing and monitoring and later retrieval of the same delivers undesirable dose. The maximum dose recorded at the bottom of the package, delivers unnecessary wrist dose to personnel handling the packages. Modification Incorporated: The modifications incorporated and the impacts on the exposures of the staff are:

1. Quick-fit clipping arrangement for the lid and the lead pot. This eliminated the dose due to sealing the assembly with monsoon tape.
2. Introduction of handle strip enables to carry the lead pot easily, after transfer of vial containing activity. This has reduced both chest and wrist dose considerably.
3. Transfer of the lead pot containing activity directly into moulded high-density "thermocol", housed inside a cylindrical standardized package bucket shaped package. This has eliminated intermediate tin container packing step.
4. The activity in the liquid form present at the bottom of the vial, has been increased by introducing 6 cms. thick thermocol packing. This has resulted in the reduction of wrist exposure, considerably.
5. Pneumatic lid sealing of the package. This has eliminated wrist dose received due to nailing of wooden package considerably.
6. Direct transfer of the packages into the dispatch vehicle with the help of the handle attached to the bucked shaped package. This has resulted in large reduction of dose to the staff.

**Result:** The collective dose of the staff against the number of the packages dispatched and also the collective dose of the staff against total activity dispatched, annually for the period from 1991 to 2005

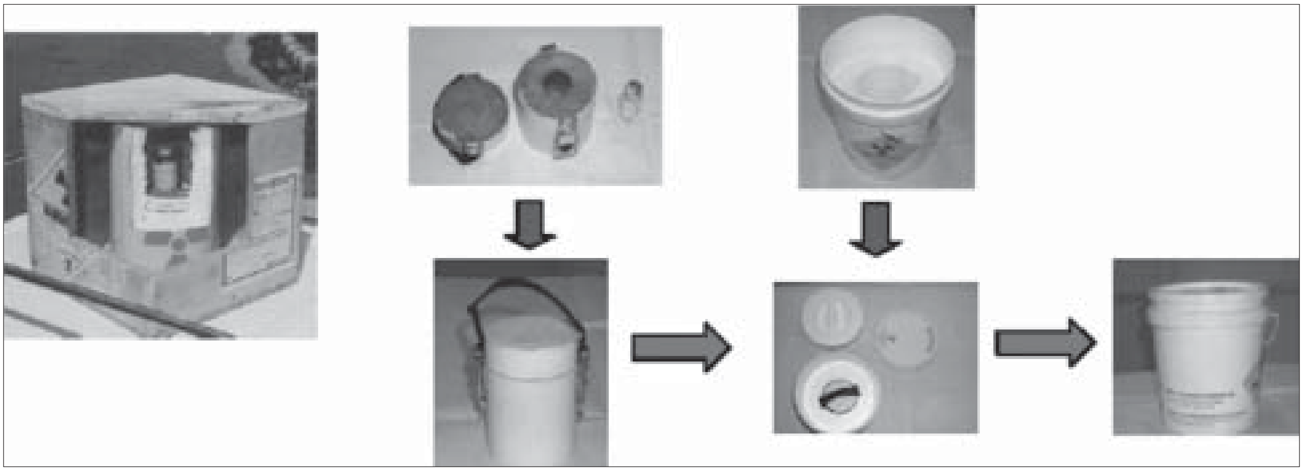

**Figure 1:** Old conical wooden package (Type-A) and new bucket shape package (Type-A) with modified lead pot

[Downloaded free from http://www.jmp.org.in on Friday, July 29, 2011, IP: 130.14.254.25] || Click here to download free Android application for t

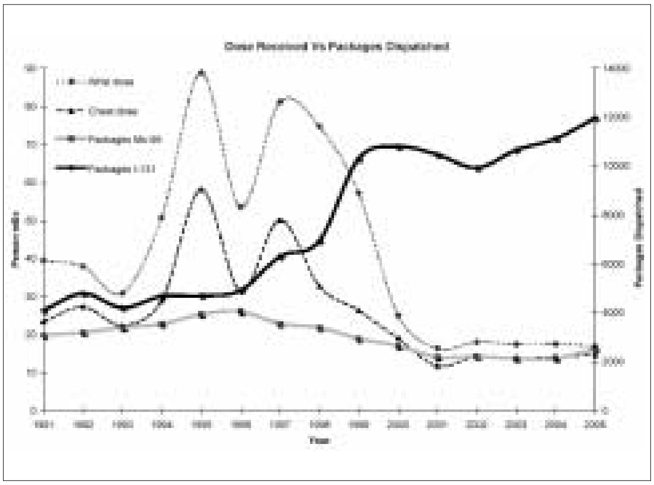

**Figure 2:** Collective dose received Vs number of packages dispatched, annually

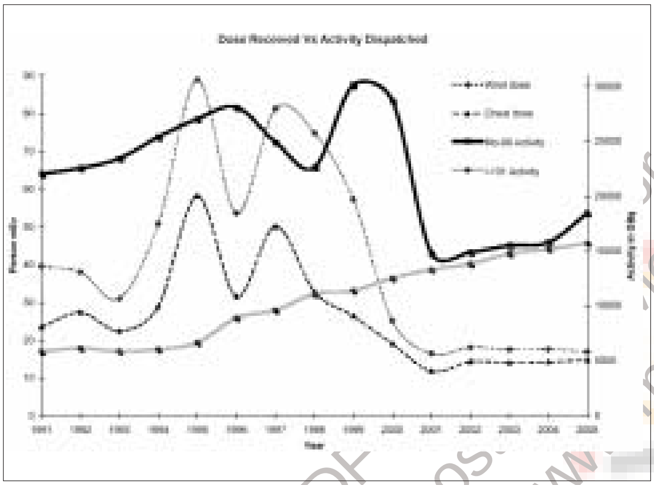

**Figure 3:** Collective dose received Vs total activity dispatched, annually.

was brought in the Figures 2 and 3 respectively. From 1991 till 1995 the total activity ($^{99}$Mo and $^{131}$I) and also number of packages dispatched increased considerably, thus also the person-Sv consumed (both chest and wrist dose). Modified procedure was introduced and the introduction of new package for the dispatch of $^{99}$Mo from June 1995 [Figure 1]. This has resulted person-Sv reduction by a factor 1.8 (chest) and 1.65 (wrist) in 1996. There was a reduction of $^{99}$Mo packages and activity (except in mid 1999) dispatched since 1996 onwards. But there was a continuous increase (≈10%) in $^{131}$I number of packages and activity from 1996 onwards. Due to this increase, there is an increase in person-Sv for the year 1997 also. With the introduction of new packages and modified procedure for $^{131}$I activity was commenced from 1998, which has resulted in continuous reduction of person-Sv since then [Figures 2 and 3]. The new package and modified procedure introduced for the packing of radiopharmaceutical packages resulted in the over-all reduction of 5 times in the wrist dose and ≈4 times in the chest dose from 1995 to 2005 for the staff engaged in the packing. Also, manual labour was reduced considerably. Further reduction in person-Sv can be achieved by introduction of full automation in the packing and monitoring work.

### P-51

### Radiation safety in a cyclotron: PET/CT facility

GS Pant, S Senthamizhchelvan. *Dept of Nuclear Medicine, AIIMS, New Delhi - 110029, India*

**Introduction:** Positron emission tomography (PET) imaging is used to study the *in vivo* physiological and metabolic processes by tagging biologically active chemicals with positron emitting radionuclides. The ultra short half-lives of the radionuclides used in pet require the onsite cyclotron to produce them. the presence of a cyclotron in a medical facility creates unique radiation safety issues. This paper addresses issues related to radiation safety in a self-shielded cyclotron and PET/CT facility.

**Materials and Methods:** We performed radiation survey for gamma rays and neutrons in and around an 11MEV self shielded pet cyclotron before, during and after radioisotope production. We also did the radiation survey of the radiochemistry laboratory during synthesis of positron emitting radiopharmaceuticals. Radiation dose levels in and around imaging room were also measured. The exposure rate from patients administered with $^{18}$f-fdg was also recorded during uptake period and before PET/CT imaging. The radiation dose to the staff handling the patients was also measured. the cyclotron is completely self-shielded during bombardment and the chemistry laboratory has heavily shielded mini cell for synthesis and hot cell for dose dispending and calibration.

**Results and Conclusion:** The gamma ray dose levels at 1 meter from the outer surface of cyclotron shields in the cyclotron room varied from 0.3 to 0.5 msv/hr, 1.5 to 50 msv/hr and 0.4 to 5 msv/hr before, during and after bombardment respectively. The neutron dose rate was much less, which varied from 0.01 to 14.2 msv/hr during bombardment. The dose levels around the chemistry laboratory during synthesis varied from 0.3 to 4.0 msv/hr. Around the PET/CT room the exposure level were found well within acceptable limits. The exposure rate from patients was reasonably high at close distances. It is therefore necessary for the staff to keep distance during injection, transportation and positioning of patients till they leave the pet facility after completion of imaging. However the radiation dose to staff handling the injection and scanning of the patients were well within annual dose limits.

### P-52

### Evaluation of HVL for lead alloy - Measurements and Monte Carlo calculations

Neeraj Dixit, T Palani Selvam, K Biju, S Kannan. *Radiological Physics and Advisory Division, Bhabha Atomic Research Centre, Mumbai - 400 094, India*
E-mail: ndixit1@yahoo.com

**Introduction:** Lead is not a recommended material for structural shielding for x and gamma rays because it is soft, highly malleable and ductile toxic metal. Although it is resistant to corrosion it tarnishes upon exposure to air. Lead fusible alloy (density, $\rho$ = 9.67 gcm$^{-3}$ and effective atomic number, $Z_{eff}$ = 77.6), indigenously developed, may be used as a shielding material as it can be mould in the form of tiles. It's strength is better than lead. The aim of present study is evaluation of value of HVL for the lead alloy at $^{60}$Co energy based on measurements in a telecobalt radiation field using a 0.6 cc ionization chamber. In addition, Monte Carlo calculations were also performed using MCNP code.

**Materials and Methods:** HVL measurements are generally carried out in air or in phantom under good geometry conditions. In our study, central axis transmission measurements using the 0.6 cc ionization chamber in a 30x30x30 cm water phantom were carried out as a function of thickness of lead alloy against a telecobalt beam (Thetratron-780 E telecobalt unit). In the measurements source-to-phantom distance was 80 cm and the filed size at this distance was 10x10 cm$^2$. The alloy was positioned at 55 cm from the source and the chamber was at 15 cm depth in the water phantom. The measured ionization currents with and without the alloy were used for calculating HVL value (HVL = 0.693/m, where m is the linear attenuation coefficient of lead alloy).

**Monte Carlo computations:** We carried out Monte Carlo simulations using the MCNP code by two approaches. In the first approach (approach 1) air-kerma in air was calculated. In the second

[Downloaded free from http://www.jmp.org.in on Friday, July 29, 2011, IP: 130.14.254.25] || Click here to download free Android application for t approach (approach 2) we simulated the experimental set up. In this approach air-kerma at 15 cm depth in water phantom was scored. In both the approaches, the incident beam at 80 cm was circular. The radius of the beam was chosen to give 100 $cm^2$ area. The Monte Carlo calculations were carried out using a realistic photon spectrum from a typical telecobalt unit (Rogers *et al* 1987) as well as using two principal gamma lines (1.117 and 1.33 MeV) $^{60}$Co.

**Results and Conclusion:** The measured HVL value obtained for the lead alloy is 1.30 as opposed to Monte Carlo-calculated values of 1.22 and 1.20 obtained based on approaches 1 and 2, respectively, when $^{60}$Co spectrum (fluence-weighted mean energy is 1.05 MeV) was used. This suggests that HVL measurements may be done in water. Auxiliary calculations suggest that the Monte Carlo-calculated HVL value obtained for lead alloy using 1.17 and 1.33 MeV gamma lines is 1.26 cm. The HVL value obtained for pure lead (density 11.34 $gcm^{-3}$) using latest value of linear attenuation coefficient at 1.25 MeV (0.666 $cm^{-1}$) is 1.04 cm. Using density scaling, the value of HVL for lead alloy is 1.22 cm [= (11.34/9.67) x 1.04 cm]. This suggests that approach of mass density scaling is valid for HVL calculation. As Monte Carlo method is known for accurate modeling of particle transport, for practical purposes, we recommend a HVL value of 1.26 cm for the lead alloy.

### P-53

### Use of fluoro-glass for dosimetry evaluation of $^{60}$Co gamma chamber

V Raja, SG Mhatre, AK Mahant, VV Shaha. *Radiation Standards Section, Radiation Safety Systems Division, Bhabha Atomic Research Centre, Mumbai, India*

**Introduction:** Radiophotoluminescent (RPL) glass dosimeters provide a convenient method to measure cumulative dose over a wide dose rate range. Experiments were carried out to measure transit dose and dose rate from a $^{60}$Co gamma chamber (GC900) available in our laboratory using FD-P8 fluoro-glasses developed by Toshiba, Japan. A comparison of dose to water value of $^{60}$Co gamma chamber obtained by fluoro-glass dosimeter and Fricke chemical dosimeter showed an agreement within ±2%.

**Materials and Methods:** FD-P8 silver activated phosphate glass (8x8x4.7 mm) was used for these studies in conjunction with the Toshiba model FGD-6 fluoro glass reader manufactured by Toshiba, Japan. Glass dosimeters were thoroughly cleaned and annealed at 400°C for 1 hour. Beam output of $^{60}$Co teletherapy machine (Model Th780E) in terms of exposure rate was measured at 80 cm from the source for a field size of 10x10 cm using the reference ion chamber NE2571/1692 of this laboratory. FD-P8 glasses encased in Teflon and in a 0.9 mm tin filter were exposed to different exposures ranging from 200R to 2500R. After predose correction, linearity of the glasses was found to be within ±3% in 200R to 2500R range. The Gamma Chamber (GC900) used for these studies as a source of high intensity gamma radiation field has sixteen $^{60}$Co pencil sources in annular cylindrical geometry. A high dose rate of 4000-5000 R/min is obtained at the centre of irradiation volume. During its movement to the final irradiation position a transit dose to the sample results due to leakage radiation from the source pencils in the gamma chamber. The evaluation of the transit dose is important for the correct measurement of the dose received by the sample. Measurement of transit dose was done by irradiating one FD-P8 glass for 20 seconds and another FD-P8 glass also for 20 seconds, but in two intervals of 10 sec each. The transit dose (450 R) was calculated as follows.

$D_1 = D_{20} + D_x$ [$D_1$ =total dose to first glass, $D_{20}$ = dose in 20 s in stable irradiation condition, Dx = transit dose]

$D_2 = D_{10} + D_x + D_{10} + D_x$ [$D_2$ = total dose to second glass, $D_{10}$ = dose in 10 s in stable irradiation condition] Also $D_{20} = 2D_{10,}$ hence $Dx = D_2 - D_1$

FD-P8 glasses were irradiated for different exposure times viz. 10 sec, 15 sec, 20 sec and 25 sec at the centre position of GC900. Exposure rate of gamma chamber was determined by measurement of signal obtained from these glasses in the reader and was found to be 4500 R/min.

Under conditions of electronic equilibrium, the dose in air is related to the dose to the water by the following relationship:

$$D_{water} / D_{air} = [(\bar{\mu}_{en}/\rho)_{water} / (\bar{\mu}_{en}/\rho)_{air}].\ A_{eq} \quad (1)$$

$A_{eq} = 0.99$ and $[(\bar{\mu}_{en}/\rho)_{water} / (\bar{\mu}_{en}/\rho)_{air}] = 1.112$ at $^{60}$Co energy.

$$D_{water} = X.\ W/e.\ [(\bar{\mu}_{en}/\rho)_{water} / (\bar{\mu}_{en}/\rho)_{air}].\ A_{eq} \quad (2)$$

For exposure (X) in roentgens and $D_{water}$ in rad;

$$D_{water} = 0.8764.\ [(\bar{\mu}_{en}/\rho)_{water} / (\bar{\mu}_{en}/\rho)_{air}].\ X.\ A_{eq} \quad (3)$$

Dose rate to water and transit dose as calculated using the formula (3) are presented in Table 1.

Table 1: Dose rate to water and transit dose

| *Dosimeter* | *Transit dose (Gy)* | *Dose rate to water ($D_W$) Gy/min* |
|---|---|---|
| Fluoro-glass (FD-P8) | 4.34 | 43.4 |
| Fricke | 4.30 | 42.9 |

**Conclusion:** A comparison of dose rate to water and the transit dose value for $^{60}$Co gamma chamber obtained by fluoro-glass dosimeter with that of Fricke dosimeter showed an agreement better than ±2%. These experiments have demonstrated the potential of fluoro glass dosimeters for high dose rate measurements

### P-54

### Radiation source reloading in gamma knife: First of its kind in India

RK Bisht, N Gopishankar, EB Rajmohan, SS Kale, GK Rath, AK Mahapatra. *All India Institute of Medical Sciences, New Delhi, India.*
E-mail: raaj_bisht@rediffmail.com

**Introduction:** Gamma knife (GK) radiosurgery destroys small intracranial targets using Gamma rays. The idea behind source reloading in GK machine is similar to the source reloading in other radiotherapy units containing the radio-isotopes, but some specific procedures and mechanisms involved in GK source reloading makes it different from conventional radiotherapy machines.

**Materials and Methods:** There are 201 Co-60 sources in the hemisphere array of the GK unit head. Each source is composed of about 20 Co-60 metal pellets of 1 mm diameter and 1 mm in length. The pellets are contained in double stainless steel capsules with fission welded closures with specific activity approximately 250 Ci/gm. Each source is labeled with special coding, which decides the position of the source in the five-ringed hemispherical surface array. The activity of each sealed Co-60 source pellet is nearly 0.981±3% TBq. All sealed sources are packed in a lead container and kept inside an iron container, which have hooks and base designed for the easy lifting and transportation. Source package containing fresh sources [Type B (U), Yellow III] surveyed and found the maximum level as 40 mSv/hr at the surface and 4 mSv/hr at 1 m distance. Before placing the source container in the treatment room, loading machine was placed near the head of the GK unit. It provides the facility to transfer and place all old and new sources inside the container simultaneously. Minimum three persons are required to perform the procedure. One person rotates the source holding hemispheric body using hand wheel and aligns beam channels individually with the five openings in the radiation shielding. The second person positioned near the first chamber inserts a long tong into the source hemisphere removes the old source and passes it to another chamber. The third person positioned near the second chamber receives the old source from the first chamber and places them inside the source container. Once all the old sources are removed from the machine head, a survey is made to check the radiation level around the container. Maximum level of 57.8 mSv at the surface and 6.3 mSv at 1 m distance were observed, when both the old and fresh radiation Co-60 sources were placed inside the container, it was ensured that none of the source were left behind in the machine head. Procedures for loading fresh sources starts from the second chamber, the third person lifts the new sources in second chamber

This PDF is available for free download from a site hosted by Medknow Publications (www.medknow.com).

[Downloaded free from http://www.jmp.org.in on Friday, July 29, 2011, IP: 130.14.254.25] || Click here to download free Android application for t from the source container lying beneath the loading machine and transfers it to the first chamber with long tongs. Second person receives the new sources from second chamber and inserts in to the machine head with bush mechanism. Meanwhile the first person aligns the source position in the source hemisphere.

**Quality assurance, performance and comissioning:** Stereotactic radiosurgery with GK systems represents the dedicated technique of precision radiotherapy. It requires the different set of quality assurance parameters that requires minimum QA tests.

a) Checking of several interlocks, dummy treatment, treatment stop button and emergency interrupts.
b) The microswitch in each of the collimator helmet with in 0.1 mm accuracy at the treatment positioning.
c) Checking of coincidence of mechanical isocenter (UCP) and radiation Isocenter.
d) Checking of the integrity of trunnions by using cross bar.
e) The wipe test of the surface of the helmet to check if there is any leakage of the source encapsulation.

**Dosimetric Evaluation**: Radiation output measurement was measured at the isocenter (40.3 cm) with 0.125cc PTW ionization chamber coupled with Janus electrometer (at - 400 Volts. polarisation voltage to chamber) using solid spherical polystyrene phantom 16 cm in diameter aligned at X=100 in the unplugged 18 mm collimator. Gamma angle was set at 90° and sensitive volume was centered at X=Y=Z=100. Based on dosimetric evaluation, dose rate at focus, collimator scatter factor and decay factor for cobalt source were fed to the beam library files of LGP. The calibration of planning system involved, beam library corrections, printing of physics protocol and comparing the physics parameters such as dose rate, attenuation coefficient and collimator factors of different collimator helmets.

**Conclusion:** Throughout the procedure, members involved received doses which were under safe recommended limits of the radiation exposure. Gammaknife source reloading is a safe procedure.

### P-55

### Modulation of radiation induced biochemical changes in brain of Swiss albino mice by *Grewia asiatica*

Muktika Ahaskar, KV Sharma, Rashmi Sisodia*. *Radiation Biology Laboratory, Department of Zoology, University of Rajasthan, Jaipur - 302 004, India*

Grewia asiatica fruit found in abundance in western Rajasthan is known for its medicinal properties since ages. Although, it is rich in anthocyanin and vitamin C etc., which have emerged as powerful antioxidants, its possible role in radioprotection has not been explored. The present study is therefore an attempt to evaluate its possible radioprotective role in brain of Swiss albino. The effect of oral administration of methanolic extract of *Grewia asiatica* (Phalsa) (GAE) fruit against gamma irradiation was studied in mice. Healthy Swiss albino mice from an inbred colony were divided into four groups viz.1. Normal 2. Only Drug treated 3. Control (Irradiated) 4. Drug + irradiation (experimental). Groups 2 and 4 were treated with GAE for 15 days, at 700 mg/kg of b. w. /day. After treatment group 3 and 4 were exposed to 5 Gy gamma radiations. Mice in all the groups were examined and autopsied at intervals of 1, 3, 7, 15 and 30 days post irradiation. Brain was removed and estimated for various biochemical changes. The mice of experimental group exhibited significant modulation of radiation induced decreases of reduced glutathione (GSH) and radiation induced increase in lipid peroxidation (LPO) in the whole brain at all autopsy interval. Maximum modulation in GSH (16.03%) and LPO (24.22%) was noticed at 1st day post irradiation. At day 30 the values reached near normalcy. The findings support antioxidative properties of GAE against the gamma irradiation. This may be due to scavenging of free radicals by anthocyanin and vitamin C present in *Grewia asiatica* extract. However, further investigations are needed.

### P-56

### Radioprotection of Swiss albino mice against whole body gamma irradiation by *Grewia asiatica*

Smita Singh, Muktika Ahaskar, Rashmi Sisodia. *Radiation Biology Laboratory, Department of Zoology, University of Rajasthan, Jaipur-302 004, India*
E-mail: rashsisodia@yahoo.co.in

**Introduction:** The radioprotective effects of *Grewia asiatica* fruit against radiation induced hematological and biochemical alterations in peripheral blood and the survival of Swiss albino mice were studied. *Grewia asiatica* (Phalsa), indigenous plant of Indian subcontinent also found in abundance in western Rajasthan, is known to have ethno medicinal properties. It is rich in antioxidants like vitamin C, anthocyanin and folate that play an important role in radioprotection.

**Materials and Methods:** A regression analysis of survival data obtained after 5,7, 10 and 12 Gy irradiation to mice yielded $LD_{50/30}$ as 6.21 and 9.53 Gy for the control and experimental group respectively. It and produced a dose reduction factor (DRF) of 1.53. For evaluating haematological parameters, healthy Swiss albino mice from an inbred colony were divided into four groups viz.1. Normal 2. Only Drug treated 3. Control (Irradiated) 4. Experimental (Drug + irradiation). Groups 2 and 4 were orally supplemented with GAE for 15 days, at 700 mg/kg of b. wt./day. After treatment group 3 and 4 were exposed to 5 Gy gamma irradiation. Significant increases in total erythrocyte (20%) and leucocytes counts (22%), hemoglobin concentration (22.33%) and hematocrit values (12.12%) were observed in the animals of experimental group in comparison to the hematological values observed in the control group at 24 hour post irradiation. A decrease in reduced glutathione (GSH) content and an increase in lipid peroxidation (LPO) were observed in control animals. However, the animals of the experimental group exhibited a significant ($P<0.001$) increase (31.80%) in GSH content and a significant ($P<0.01$) decrease (28.76%) in LPO level.

**Results and Conclusion:** The findings support the antioxidative properties of GAE against the gamma irradiation. This may be due to scavenging of free radicals by anthocyanin and vitamin c present in *Grewia asiatica* extract. However, further investigations are needed.

### P-57

### Radiomodulation by rosemary (*Rosemarinus officinalis*) leaves extract against intestinal lesions in mice

Archana Jindal, PK Goyal. *Radiation and Cancer Biology Laboratory, Department of Zoology, University of Rajasthan, Jaipur, India*

**Introduction:** The twentieth centaury has seen an increasing use of nuclear energy in medical, engineering, industrial and scientific research and for generation of electricity. Such uses have raised the problem of radiation hazards to living beings. The deleterious effects of radiation can now be strategically counterpoised by the use of many drugs and chemicals. A large number of chemicals have been investigated during last five decades to overcome radiation damage. However, the inherent toxicity of these agents at the useful concentration has warranted further exploration. The herbs have been commonly used to treat various disorders in man since the advent of human history. Humans are dependent on herbs not only for medicinal use but also consume herbs and fruits for their sustenance. Therefore, use of products from natural sources could be a better choice to reduce the deleterious effects of radiation. Intestine is one of the most rapidly renewing tissues in the body and hence it responds rapidly to injury. Intestine has valuable quantitative parameters as well as serially arranged qualitative cellular configuration. *Rosemarinus officinalis* (Rosemary), belonging to family labiatae, is an evergreen branched bushy shrub, attaining a height of about one meter with upright stems, whitish blue flowers and dark green leaves. Rosemary extract (ROE) relaxes smooth muscles of trachea and intestine and has choleretic, hepatoprotective and antimutagenic activity. The present study is an attempt to evaluate its possible protective effects on intestine in mice against radiation exposure. Materials and Methods Male Swiss albino mice selected from inbred colony were divided into four groups. Animals of Group-1 were administered orally with DDW (volume equal to

This PDF is available for free download from a site hosted by Medknow Publications (www.medknow.com).

[Downloaded free from http://www.jmp.org.in on Friday, July 29, 2011, IP: 130.14.254.25] || Click here to download free Android application for t

ROE) to serve as normal while animals in Group

**P-58**

**Patient specific IMRT QA with gafchromic films and ionization chamber**

Gautam Mukherjee, Alok Kumar, Girigesh Yadav, Vinod Pandey, Arundhati Chakrawarti, Kalyan Bhattacharya. *AMRI Hospitals, Kolkata, India*

**Introduction:** IMRT is one form of conformal therapy where radiation beams have non-uniform radiation intensities delivered across the two-dimensional field. But the dose delivery in IMRT is very complex and if misapplied may result in excess tumour recurrences or complications that will negate the potential benefits of this new technology. Film dosimetry is one of the most powerful tools for verification/validation of such complex treatment. Measurement of isodose curves and depth dose distributions in high-gradient regions of beams using conventional measuring systems such as TLDs and radiographic films involve numerous practical problems. Measurement of dose with the help of TLDs is both cumbersome and time consuming while Radiographic films suffer from high degree of variances in sensitivity in the photon energy range 10-200 keV. Energy absorption properties of such films do not match those of biological tissues. Moreover, radiographic films also have the disadvantages of sensitive to room light and requiring wet chemical processing. This led to the development of Gafchromic EBT films for IMRT plan dosimetry. Radiochromic films have double-layer radiochromic sensors coated on both sides of a polyester base of 97 micron thickness. In the following procedure we have measured response of Gafchromic film using VIDAR film scanner (Nucletron make) and flat bed scanner using VERISOFT and FILM QA softwares respectively. IMRT point dose measurement using 0.125cc chamber was also done as a QA procedure.

**Materials and Methods:**

1) CT scan of IMRT phantom is done having center markings with three ROBs (Radio Opaque Balls). The scans are taken both in coronal as well as sagittal plane.
2) The scans are transferred to virtual simulation software for contouring.
3) The contoured images are exported via DICOM RT to PLATO treatment planning software.
4) Patient plan is created in the imported images of the IMRT phantom.
5) The TPS dose grid file is exported via DICOM RT to both VERISOFT and FILM QA film scanning softwares in axial and coronal planes. The patient plan is also exported to the LINAC machine.
6) Expose small pieces of Gafchromic film to various dose levels- 25 cGy, up to 300cGy in 25 cGy interval. These exposed films are scanned in the VIDAR film scanner and flat bed scanner to generate a look-up-table or calibration curve for the film.
7) Expose the Gafchromic film by executing patient plan in the machine by putting the film inside the IMRT phantom both in coronal and axial planes. Sufficient time is given for exposed films for post irradiation colour stability (around 1 hour) before scanning the films.
8) The Dose Grid file from PLATO and the exposed film is compared and analyzed in VERISOFT and FILM QA software on the basis of gamma index (The minimum radial distance between the measurement point and the calculation points expressed as a surface in the dose-distance space is termed the Gamma ($\gamma$) Index), DTA (the distance-to-agreement is the distance between a measured data point and the nearest point in the calculated dose distribution that exhibits the same dose), dose difference, 2D and 3D isodose check, Profile match etc.
9) For point dose measurement, patient plan is executed in the IMRT phantom using 0.125cc ionization chamber (PTW make) kept at the isocentre of the phantom. This is compared with the TPS calculated dose. In any discrepancy in the dose measurement, the measurement was done in the flat region of profile of the particular field.

**Result and Conclusion:** The difference between calculated and measurement data is within 3% and 3 mm. The Gafchromic films has high spatial resolution, low spectral sensitivity variation, insensitive to room light, do not require chemical processing, near Tissue equivalent, low energy dependence, independent of dose rate effect between 2-4 Gy/min and linearity over the wide clinical range. Both the scanners Vidar and flat bed scanner give equally good result with Gafchromic film, however the flat bed scanner is less expensive than Vidar film scanner. Any normal phantom can give the point dose measurement with thimble chamber with acceptable level.

**P-59**

**Role of $^{18}$F-FDG PET/CT scan in the evaluation of esophageal carcinoma**

Praveen Kumar, CS Bal. *Department of Nuclear Medicine, AIIMS, New Delhi, India*

**Introduction:** Esophageal carcinoma (EC) is one of the most fatal cancers primarily because of its late presentation. It is a common cancer in Asian countries, particularly countries like Iran, China and Japan. In India also the incidence is high (>10 per 100,000 men) in some parts such as Kashmir, Assam and Mumbai. A prospective study of preoperative and postoperative tumor-node-metastasis staging of patients with esophageal carcinoma (EC) was designed to compare the accuracy of $^{18}$F-fluorodeoxy-glucose (FDG) PET/CT scan with conventional modalities like endoscopic ultrasound (EUS), CT scan and histopathology findings.

**Materials and Methods:** Patients with potentially resectable esophageal carcinoma were included. A whole-body PET/CT scan was acquired after injection of 18F-FDG and was evaluated for areas of increased focal uptake. Accuracy was determined by comparing PET with surgical staging.

**Results:** Potentially resectable esophageal cancer was identified in 5 patients (till now). PET detected three sites of distant metastases, which were missed by conventional scanning. FDG-PET had a higher accuracy for diagnosing stage IV diseases compared with the combination of CT and EUS (82% v 64% respectively). PET improved diagnostic specificity for LN staging.

**Conclusion:** Positron emission tomography improved our ability to detect distant metastases missed by conventional non-invasive staging of esophageal carcinoma. Early use of PET in the staging of patients with esophageal cancer could facilitate treatment planning and identifying unsuspected distant metastasis with a negative metastasis survey by conventional staging.

**P-60**

**Role of I-131 MIBG scintigraphy in the diagnosis and management of neuroendocrine tumours**

Sarita Kumari, R Kumar, GP Bandopadhayaya, CS Bal, GS Pant, A Malhotra. *Department of nuclear medicine, All India Institute of Medical Sciences, New Delhi, India*

**Introduction:** The purpose of this study was to assess the role and efficacy of Iodine-131 metaiodobenzylguanidine (MIBG) scintigraphy in diagnosis and management of neuroendocrine tumours.

**Materials and Methods:** During the year 2004 to 2005, forty-one patients (31 males and 10 females) ranging in age from 7 months to 62 years (mean age 21.83 years) with confirmed or suspected neuroendocrine tumour that underwent I-131 MIBG scans were included in this study. All patients underwent I-131 MIBG scintigraphy for diagnosis or disease evaluation on clinical follow up. A variety of neuroendocrine tumours were analyzed: 16 had pheochromocytoma, 15 had neuroblastoma, 2 had medullary thyroid carcinoma and 1 each had pancreatic head mass, pulmonary tumour, parathyroid adenoma, parathyroid carcinoma, paraspinal tumour, neurogenic tumour, bilateral adrenal gland hyperplasia and pelvic primitive

This PDF is available for free download from a site hosted by Medknow Publications (www.medknow.com)

[Downloaded free from http://www.jmp.org.in on Friday, July 29, 2011, IP: 130.14.254.25] || Click here to download free Android application for t neuroendocrine tumour. The images were interpreted and analyzed with respect to uptake in non- physiological areas, intensity and features of the tracer uptake. These findings were then compared to any previous information or results from other anatomical imaging modalities such as ultrasonography, CT and MR imaging.

**Results:** I-131 MIBG findings were interpreted as positive in 20 patients. Of these all were eventually diagnosed with neuroendocrine tumours. I-131 MIBG scans were interpreted as negative in 21 patients. There was no false positive lesion in this study. Five false negative patients were encountered during the study; among these 2 were adrenal lesions with size less than 1.5 cm on CT/MR imaging; other 3 false negative results were seen in medullary thyroid carcinoma with bone metastases, paraspinal tumour and pelvic primitive neuroendocrine tumours. The sensitivity, specificity, positive predictive value, negative predictive value, false negative rate, false positive rate and overall accuracy of I-131 MIBG in diagnosing neuroendocrine tumour were 80%, 100%, 100%, 76%, 20%, 0% and 87%, respectively.

**Conclusion:** I-131 MIBG scintigraphy is a sensitive and highly specific non-invasive technique for diagnosis and disease evaluation in neuroendocrine tumors.

### P-61

### Recovery coefficient method for the correction of partial volume effect in PET images

Manjesh Pandey, Rakesh Kumar. *Dept. of Nuclear Medicine, AIIMS, New Delhi, India*

**Introduction:** Due to the limited spatial resolution of the PET scanners, the structures (hot spots) which are smaller than twice the FWHM show partial loss of intensity and the activity around the structure appears to be smeared over a larger area. This underestimates the activity in the lesion or may overestimate the size of the lesion. The aim of this study was to make necessary correction where ever possible in PET images.

**Materials and Methods:** Hollow spherical phantoms of different volumes were filled with $^{18}$F-FDG and submerged in a cylindrical phantom filled with tap water containing much smaller activity as compared with the spherical phantoms such that the ratio of the activity in the spheres and background water was 8: 1. This is repeated with the sphere to background activity ratio as 6: 1 and 4: 1. The total activity used in each experiment was 1.8 mCi, The phantoms were scanned with scan duration of 20 minutes. Using the recovery coefficient formulae the ratio of measured and known activity was obtained for all the spheres.

**Results:** The recovery coefficient data was used for calculating the partial volume effect. This data can be used for partial volume correction of PET images.

**Conclusion:** The study could provide a correction table for partial volume correction of PET images. Our method of using hot spheres in a warm background most closely simulates the imaging environment in a living subject. This method could facilitate generation of equipment specific recovery coefficients for partial volume correction in PET/CT imaging.

### P-62

### Radiation exposure to staff during routine monitoring of thyroid Cancer patients in isolation ward

Amarjeet Kumar, GS Pant, A Malhotra. *Dept. of Nuclear Medicine, AIIMS, New Delhi, India*

**Introduction:** Estimation of radiation dose to the technologist during monitoring of thyroid cancer patients in isolation ward.

**Materials and Methods:** The dose estimation to the technologist has been done for monitoring 100 thyroid cancer patients. Dose administered to the patients ranged from 40 mCi to 200 mCi (1.1G Bq to 7.4 G Bq) of I-131 with a mean dose of 85.88 ± 8.5 mCi. There were 39 male and 61 female patients treated (mean age 39 ± 3.9). Average distance of technologist from the patient was estimated during therapy, monitoring and post therapy scanning. The duration of stay was also recorded at these distances. The dose rate (DR) was measured at a distance of 1.0 m and 2.0 m with a calibrated survey meter available in the department.

**Result:** Exposure to technologist was estimated based on monitoring once a day for total duration of patients' stay in the hospital. The duration of stay was taken as 15 seconds each at 1.0 m and 2.0 m distance. The values are given in the table.

**Conclusion:** The estimated dose to the staff involved in routine monitoring of thyroid cancer patients appears to be much less than prescribed annual dose limits by the competent authority in India. Thus the associated risk to the staff/technologists is minimal provided the laid down radiation safety guidelines are strictly followed.

| *Day 1* | | *Day 2* | | *Day 3* | *Day 4* |
|---|---|---|---|---|---|
| 1.0 m | 2.0 m | 1.0 m | 2.0 m | 1.0 m | 1.0 m |
| 57.44 μSv/TD | 21.22 μSv/TD | 14.42 μSv/TD | 7.4 μSv/TD | 5.05 μSv/TD | 0.7 mSv |

Total exposure in 65 days was estimated as 106.23 μSv which may result in an annual dose of 0.59 mSv.

### P-63

### Analysis of factors involved in patient tolerance limit during the radiation treatment in hyper fraction versus conventional fraction of CO-60 EBRT in head and neck cancers

A Balraj, PB Vidyasagar*, N Chacravarty**, PK Thakur, S Bhatnagar, SS Supe***. *Dept of Radiation Oncology, Command Hospital (SC) Pune - 411 040, *Dept of Physics, University of Pune, Pune - 411 007, **Dept of Radiation Oncology, Army Hospital (R and R), Delhi cantt - 110 010, ***Dept of Radiation Physics, Kidwai Memorial Institute of Oncology, Bangalore, India*

**Introduction:** Most patients with head and neck cancer are treated with EBRT (External Beam Radiotherapy). Radiation therapy planning ideally sets up fields to cover the tumour adequately while treating as little normal tissue as possible. Unfortunately, head and neck cancer is a regional disease and typically requires treating regional lymph nodes as well as the primary tumour. This approach requires wide radiation fields. During the treatment there is a predictable incidence of xerostomia, dysgeusia, anorexia, chewing and eating difficulties, mucositis and infection. These factors decide the patient tolerance limit of radiotherapy. These factors may be reduced by using cytoprotective drug, where as in this study we have analysed factors involving in patient tolerance limit without using cytoprotective drug. Objective: The cytoprotective drugs are expensive and there is no much of relief for patients treated with and without cytoprotective drug during Radiotherapy. Due to the toxicity the patient has to break the treatment, which results in incomplete or inferior treatment. Therefore improving the factors involving these side effects and risk will give more relief to patients and better tolerance of EBRT. This will improve the over all outcome of the treatment.

**Materials and Methods:** Between March 2000 to April 2004, The head and neck cancer patients were treated with hyper fractionated radiotherapy (HFRT) and Conventional fractionated radiotherapy (CFRT) with concurrent chemotherapy, HFRT alone and CFRT alone. Among these patients, 20 patients of HFRT alone and 20 patients of CFRT alone have been selected for this comparative study. A medical history and physical examination such as complete blood count, chest X-ray and in certain cases CT and MRI of the head and neck, a diagram of the primary tumour and neck nodes and a dental evaluation were obtained. The disease was staged and managed according to NCCN protocols. The patients those who were on conventional fractionation (arm 1) were treated with parallel opposing fields in the head and neck region with 2.0 Gy, 5 days in a week to receive a total dose of 60

This PDF is available for free download from a site hosted by Medknow Publications (www.medknow.com)

[Downloaded free from http://www.jmp.org.in on Friday, July 29, 2011, IP: 130.14.254.25] || Click here to download free Android application for t

Gy in 6 weeks. All patients in Hyper fractionated RT (arm 2), were treated with 2 daily fractions of 1.1 Gy, (minimum 6 hours apart), 5 days in a week for 6 to 7 weeks to receive a total dose of 66 to 77 Gy. Treatment technique: All patients were treated on Theratron 780E Cobalt - 60 teletherapy unit with isocentre parallel opposed pair of lateral fields. At 40 Gy, the field reduction was done to spare spinal cord. Second field reduction was done at 60 Gy to boost the tumour alone. The patients were examined at least once a week for acute toxicities (mucositis, skin reaction, nausea and vomiting, marrow depression and renal impairment). In addition to the tumour status, acute and late normal tissue effects were graded with Karnosfsky performance test score.

**Results:** The over all study shows that the patient tolerance limit depends upon various factors like the field size, fractional size, dose per fraction total dose, tumour staging, treatment technique, patients general condition and nourishment, hygiene, type of organs treated and depth of the tumour. The main factor involved here was the size of the field. More the field size the more is toxicity in both the arms. All larynx patients of stage I and II, due to smaller treatment field tolerated very well in both the arms. All Patients treated with HFRT arm had better tolerance than the patients treated with CFRT arm. Younger age groups tolerated better than the older age groups in both the arms. IMRT may play a major role in reducing the toxicity during the treatment, but the same can not be implemented in Co-60 EBRT.

**Discussion:** Efforts to enhance the therapeutic index in the treatment of head and neck cancer by improving efficacy and limiting toxicity involve both physical and cytoprotective approaches. Although cytoprotective drugs improve saliva protection after the completion of the treatment, there is no much of benefit during the treatment period. Physical approaches like smaller field size, treatment techniques, HFRT will reduce the toxicity in Co-60 EBRT. IMRT may be the better choice to reduce the toxicity since IMRT uses the computer analysis, inverse planning and algorithms with novel beam arrangements to "paint" the radiation dose in desired anatomic regions. It permits dose distributions that avoid certain normal structures while treating others. It is used to focus radiation on the tumour and nodes while reducing the amount of normal oral cavity and salivary tissue included in the high dose volume.

### P-64

### Effect of gamma radiation on some biological important substances

Gurpreet Kaur Sandhu, Kulwant Singh Thind. *Department of Physics, Guru Nanak Dev University, Amritsar - 143 005, India.*
E-mail: sandhu_75@yahoo.com

**Introduction:** Accurate data of transmission and absorption of X-rays and gamma rays in biological, dosimetric and shielding materials are needed in medical physics, radiation biology and other scientific areas. Mass attenuation coefficients for elements, having Z = 1 to 92 and some additional substances of dosimetric and biological importance have been compiled Hubbell and Seltzer.[1] A computer program, XCOM for calculating cross-sections and attenuation coefficients for any element, compound or mixture, at energies from 1 keV to 100 GeV has been developed by Berger and Hubbell.[2] From mass attenuation coefficients, a number of related parameters, such as mass energy-absorption coefficient, total interaction cross-section, molar extinction coefficient, effective atomic number and electron density of the absorber, can be derived. Since radioactive sources are increasingly used in biological studies, radiation sterilization and industry,[3] a thorough knowledge of the interaction of gamma radiations with materials of common use and biological importance, like amino acids, carbohydrates and fatty acids have been an important subject in the field of radiation physics. These substances play an important role in the lives of plants and animals, both as structural elements and maintenance of functional activity. Interaction cross-sections of some sugars and amino acids have been published by Nair *et al.*[4] In present study we report the mass attenuation coefficients ($\mu/\rho$) and effective atomic number ($Z_{eff}$) of some biological important substances in aqueous solutions as well as in pure form in the energy range, in which the influence of all photon interaction processes can be seen.

**Materials and Methods:** Various substances, viz. Glycine, l-Serine, l-Theronine, D-Glucose, D-Fructose, Maltose, Acetic Acid, Propionic Acid, Butyric Acid have been studied by using the narrow beam transmission experiment set-up,[5] using $\gamma$-ray energies 81 keV, 356 keV, 511 keV, 662 keV, 1173 keV and 1332 keV. The signal from the 1.5" x 1.5" NaI (Tl) crystal having energy resolution of 12% at 662-keV after suitable amplification was recorded in EG and G ORTEC 4K plug-in-card coupled to a PC/AT. The intensity of the transmitted radiation was measured by gating the channels at FWHM position of the photo peak to minimize the contributions of both small angle and multiple scattering events. ($\mu/\rho$) are calculated using precisely measured densities ($\rho$) and values of $I_0$ (transmitted intensity without absorber) and $I$ (transmitted intensity with absorber) in the following equations:

$$I = I_o e^{\frac{\mu}{p} px}$$

and $$\frac{\mu}{\rho} = \left(\frac{\mu}{\rho_w}\right) + \left[\left(\frac{\mu}{\rho_s}\right) - \left(\frac{\mu}{\rho_w}\right)\right] w_s$$

where $(\mu/\rho)_s$ and $(\mu/\rho)_w$ are the mass attenuation coefficients of solute and water respectively and $w_s$ is weight fraction of the solute. These calculated values agree well with corresponding theoretical values[3] and difference lies within experimental uncertainty and found to decrease systematically with the increase in energy as well as concentration (g/cm$^3$), for all these biological samples. Further by using the experimental data of ($\mu/\rho$) for H, C and O at different energies,[6] $Z_{eff}$ of these samples were determined, which lies in the range 3-11-4.32 and were found to be independent to the concentration of the solution and energy of the photons.

**Conclusion:** This interaction study and calculated values of parameters (($\mu/\rho$) and $Z_{eff}$) expected to yield valuable information in radiation dosimetric, radiation biology and other health oriented applications.

### References

1. Hubbell JH, Seltzer SM. Tables of X-ray Mass Attenuation Coefficients and Mass Energy- Absorption Coefficients 1 keV -20 MeV for Elements Z=1 to 92 and 48 Additional Substances of Dosimetric Interest. NISTIR: 1995. p. 5632.
2. Berger MJ, Hubble J.H. XCOM: Photon Cross sections Database, Web version 1.2, available at http://physics.nist.gov/xcom. National Institute of standards and Technology. Originally published as NBSIR: Gaithersburg, MD 20899, USA; 1987. p. 87.
3. Hall EJ. Radiation and life. Pergamon press: New york; 1978. p. 55.
4. Gopinathan Nair KP, Gowda C, Kumari JS, Anasuya SJ, Umesh TK, Gowda R. Total Interaction Cross-sections of several sugars for $^{133}$Ba. Nucl Sci Eng 1993;115:300.
5. Singh K, Gagandeep, Lark BS, Sahota HS. Study of interaction of 662 keV photons for some solutions of urea. Nucl Sci Eng 1999;32:1.
6. El-Kateb AH, Abdul Hamid AS. Photon attenuation coefficient study of some materials containing hydrogen, carbon and oxygen. Appl Radiat Isot 1991;42:303.

### P-65

### A phantom study to compare 2D electronic portal imaging with 3D megavoltage cone beam for treatment position offset calculation

Shrikant Deshpande, VK Sathiyanarayanan, Janhavi Bhangle. *Department of Radiation Oncology, Ruby Hall clinic, Pune - 01, India*

**Introduction:** To compare patient positioning with 2D electronic portal imaging (EPID) and DRR with 3D registration technique that uses MV cone beam images and planning image along with RT structures from TPS for a human skull.

**Materials and Methods:** Recently our hospital has started Image guided radiotherapy with Siemens Oncor Plus linear accelerator and coherence therapist workstation. Siemens Oncor Impression plus uses

This PDF is available for free download from a site hosted by Medknow Publications (www.medknow.com)

[Downloaded free from http://www.jmp.org.in on Friday, July 29, 2011, IP: 130.14.254.25] || Click here to download free Android application for t

MVision which is a Image guided radiotherapy platform and is capable of generating on line mega voltage cone beam imaging and auto co-registration with planning images along with RT structure used for computerised treatment planning with megavoltage images and calculates the offset in patient's treatment position. The existing system is capable of calculating the same offset calculation using 2D image data i.e., Digitally reconstructed radiographs obtained from treatment planning system with portal images obtained from a-Si flat panel (EPID). The phantom based study is reported here compares the MVCBCT imaging capability with conventional EPID 2D imaging and registration. OminoIMRT phantom and skull phantom with six fiducial marker are kept inside judiciously so that it will accounts all six dimensions degree of freedom. Inside skull phantom various objects are created using wax and fiducial are embedded inside it. Both phantoms are scanned in our Shimadzu CT simulator of slice thickness 1 mm. These images are exported to coherence Dosimetrist workstation for drawing CT structures and isocenter placement. These images along with RT structures and isocenter position were sent to Coherence Therapy workstation. Both phantoms are positioned in the treatment room and verify the treatment position using MVCBCT. On the treatment table known positional shifts are introduced in the longitudinal, lateral and vertical directions and treatment position offset calculations obtained based on 2D EPID imaging and 3D cone beam images from fiducial are noted for a few known positional shifts and compared.

**Results and Conclusion:** The MVCBCT and EPID shifts were well correlated as determined by linear regression analysis. On the shift by shift basis the 3D MVCBCT was more accurate than 2D EPID.However EPID has advantage over MVCBCT as it shows treatment portal overlays on DRR (along with RT structures) where Field edges and isocenter position can be seen and scale is also available to measure. At the present analysis level we are able to conclude that for known shifts below 1.0 cm, the offsets obtained with 2D and 3D match within 1.0 mm. When the shifts are above 1.0 cm and below 2.0 cm, on one occasion the offset matches within 2.0 mm. Investigations are done to evaluate whether it is subjective or otherwise.

This PDF is available for free download from a site hosted by Medknow Publications (www.medknow.com)

### P-66

### Evaluation of dose distribution in tangential breast planning at different planes

Rajeshri H Pai, Reena Phurailatpam, Anusheel Munshi[#], Tejpal Gupta, DD Deshpande* Shyam K Shrivastav[#]. *Department of Radiation of Oncology, ACTREC, TMC, Kharghar, Navi Mumbai, India, *Department of Medical Physics, [#]Radiation Oncology, TMH, Mumbai, India*

**Introduction:** The treatment planning for whole breast irradiation employing tangential portal is conventionally performed on a single two-dimensional (2D) contour taken at the central plane of the breast. Two dimensional dose distributions are interactively optimized using various combination of wedge angle to achieve dose homogeneity within the recommended limit (95 to 107%) of ICRU report no 50. However, varying nature of contours at different planes in the breast volume may result Inhomogeneous dose distribution to other parts of the breast. the aim of this study is to investigate dose inhomogeneity in the superior and inferior part of the breast of different sizes and present a novel approach for breast planning.

**Materials and Methods:** Twelve patients were randomly selected for this study. These patients were grouped in three different categories based on their respective chest wall separation (CWS) and the thickness of breast along the line perpendicular to the center of chest wall. Two patients were labeled as "small" (CWS <18 cm and thickness <6 cm), while five patients each were labeled as "medium" (CWS < 20 cm and thickness <7 cm) and "large" (CWS >20 cm and thickness > 7 cm) respectively. The patients were positioned supine on conventional simulator using an inclined breast board. Isocentric simulation was performed and manual contour was taken at the Isocenter plane. The involvement of lung within the tangential ports as seen in the fluoroscopic image was incorporated in the manual contours. two more contours along with the section of lung were taken at locations 5 cm superior and 5 cm inferior to the isocenter plane. Treatment planning was carried out using 6 MV X-rays and simulation parameters on plato sunrise treatment planning system (TPS). as we do conventionally, in plan 1 (p1) was generated in the single Isocenter contour and dose distribution was optimized using various wedge angle to cover the whole breast with 95% of the prescribe dose. A second plan (p2) was made on the 3d volume generated using the three contours and the same beam parameters including wedges used in p1. The dose uniformity through out the breast was evaluated at four points defined at the center, apex, medial and lateral portion of each contour. The dose at these points and the maximum point dose on the superior and inferior contours were compared with the dose at the corresponding points of p1.

**Result:** Qualitative evaluation of the dose distribution in p1 and p2 for small and medium size breast shows similar dose distribution. For small and medium size breast, dose measured at the representative points in p1 and p2 was also found to be similar. The dose measured at the representative points in the inferior and superior slices of p2 was 5 and 8% respectively. This suggests that 2D dosimetry can be adequately used for planning of small and medium size breast. In majority of the patients with larger breast, dose distribution on the superior slice was found to be more as compared to the isocenter slice of p1. The dose measured at the representative points in the inferior and superior slices of p2 was as high as 9% and 15% respectively. This implies that the optimization of dose distribution based on the 2d single isocentric contour is not adequate to decide the goodness of the plan.

**Conclusion:** Based on our dosimetric study, breast planning only on the single isocentric contour is not sufficient to evaluate dose distribution especially for larger breast. At least three breast outlines (5 cm superior, central and 5 cm inferior) should be used for dose evaluation. CT based planning particularly for larger breast is suggested for more accurate information for dose distribution through out the breast.

### P-67

### Reference air kerma rate determination for brachytherapy sources: Experimental and theoretical approach

S Vandana, A Philomina, SD Sharma, S Sahoo, A Pendse*, S Kannan. *Radiological Physics and Advisory Division, CT and CRS Building, BARC, Mumbai, India, *Department of Radiation Oncology, Hinduja Hospital, Mumbai, India. E-mail: sridhar_sahoo@indiatimes.com, bikaiv_barc@rediffmail.com*

**Introduction:** Brachytherapy is one of the unique approaches for the management of the cancer. BRIT supplies $^{137}$Cs and BARC supplies $^{125}$I sources to the different centers as per the their requirements. For brachytherapy sources, the internationally recommended unit for specifying the source strength is Reference Air Kerma Rate (RAKR), which is defined as "the air kerma rate at 1 meter along the perpendicular bisector of the long axis of the source, corrected for air attenuation and scattering". The present study reports reference air kerma rate determination of various models of $^{137}$Cs, $^{125}$I and $^{192}$Ir sources experimentally and its validation by Monte Carlo simulation.

**Materials and Methods:** In the current study a well type ionization

Table 1: Reference air kerma rate of brachytherapy sources

| *Radionuclides* | *Types* | *Activity* | *Measured RAKR (μGy/h)* | *MCNPRAKR (μGy/h)* | *Difference (%)* |
|---|---|---|---|---|---|
| $^{137}$Cs | Amersham | 60 mCi | 176.9 | 178.30 | 0.79 |
| $^{137}$Cs | CSA-1 | 120 mCi | 348 | 346.33 | 0.48 |
| $^{137}$Cs | CSA-2 | 80 mCi | 232 | 231.34 | 0.28 |
| $^{192}$Ir | HDR | 9.30 Ci | 37484 | 37510 | 0.07 |
| $^{125}$I | Ocuprosta R | 1 mCi | 1.28 | - | |

[Downloaded free from http://www.jmp.org.in on Friday, July 29, 2011, IP: 130.14.254.25] || Click here to download free Android application for t

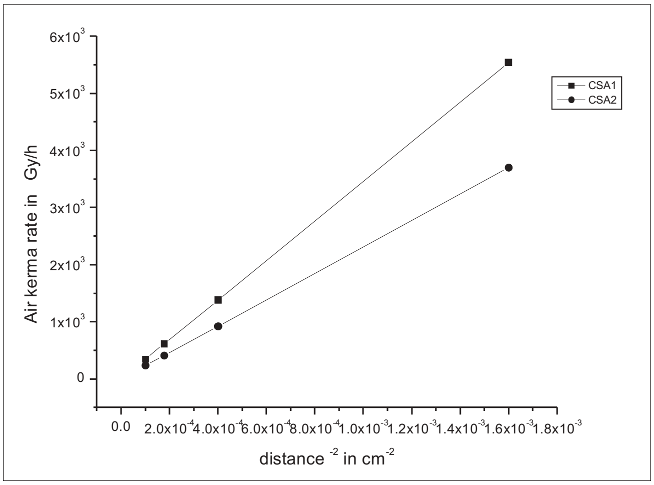

**Figure 1:** Reference Air-kerma rate against $d^2$ of BRIT$^{137}$ Cs sources chamber, model HDR-1000 plus was used to carry out reference air kerma rate determination for all the sources. This well type chamber is having calibration factors for $^{137}$Cs (CDCS-J, Amersham), high dose rate (HDR) $^{192}$Ir and $^{125}$I seed (6711, Amersham), provided by the supplier of the chamber and are traceable to University of Wisconsin, USA. The diameter of the chamber is 10.2 cm and height 15.6 cm, with an active volume of 245 cm$^3$. The HDR-1000 plus well chamber was placed at the centre of a large room, at least 1.2 meters away from all the walls and about a meter above floor levels. Response of the chamber with varying source position was obtained for each individual radionuclide. The peak position was at about 50 mm from the bottom of the well. Stability of the response was verified using reference CDCS-J type $^{137}$Cs source, which has an active length 13.5 mm, total length 20 mm and stainless steel filtration 0.5 mm. Presently, BRIT supplies two types of $^{137}$Cs sources, CSA-2, of active length 10 mm, total length 18 mm and CSA-2, of active length 15 mm and total length 21 mm. These sources are doubly encapsulated with 0.1 mm Pt followed by 0.5 mm stainless steel and having diameter of 3 mm. $^{125}$I seed source have active length 3 mm, total length 4.75 mm and diameter of 0.8 mm. This source is titanium encapsulated with a thickness of 0.05 mm. HDR brachytherapy $^{192}$Ir seed sources, have active length of 5 mm and outer diameter of 0.59 mm. The CSA-1, CSA-2 and HDR sources were simulated in 5 meter diameter free space sphere. Air-kerma rate was calculated along transverse axis at 25, 50, 75 and 100 cm using point detector tally for $10^7$ photon histories. Values of reference air kerma rate as a function of inverse square of distances were plotted. The slope of the plot gives reference air kerma rate [Figure 1].

**Results and Conclusion:** Figure 1 shows monte carlo calculated RAKR for two $^{137}$Cs sources. The numerical value of RAKR determined by Monte Carlo is also tabulated in Table 1 along with measured values for comparison. The Monte Carlo calculated value of Amersham $^{137}$Cs source is taken from published paper (Palani *et al.*) The mesurement and Monte Carlo uncertainties are within ± 3% and ± 0.05% respectively. The deviations in Monte Carlo calculated and experimentally determined RAKR for these sources are in good agreement, within 1%. Reference air kerma rate should also be used to specify the strength of the brachytherapy sources as it reduces the uncertainties involved in the correction factors. HDR 1000 plus well type ionization chamber is suitable for the calibration of brachytherapy sources both for LDR and HDR. Comparison of Monte Carlo calculated and HDR 1000 well type chamber measured RAKR for LDR$^{137}$Cs, $^{125}$I and HDR$^{192}$Ir sources suggest that well type chamber is an appropriate choice for routine calibration of brachytherapy sources.

### P-68

### Dosimetric comparison of electron arc and intensity modulated radiotherapy for PNET of chest wall

SV Jamema, PK Sharma, S Laskar*, DD Deshpande. *Department of Medical Physics, *Radiation Oncology, Tata Memorial Hospital, Parel, Mumbai - 400 012, India*

**Introduction:** Chest wall tumors of peripheral neuro-ecto dermal tumors and Ewing's sarcoma are rare and have poor prognosis. Multi-modality therapy has improved survival results. Nearly 87% of the patients require radiotherapy either in combination with surgery or with radiotherapy. In-homogeneity of chest wall thickness, large curved PTV's and proximity to critical structures makes treatment planning for chest wall tumors a complex process. The aim of this study is to compare electron arc therapy and photon intensity-modulated radiation therapy for treatment of chest wall tumors of peripheral neuro-ecto dermal tumors and Ewing's sarcoma in-terms of dosimetric accuracy.

**Materials and Methods:** Five patients of PNET of the chest wall treated with EA were included in this study. Patients were immobilized using thermoplastic moulds followed by treatment planning CT scans using the CT simulator (Somatom Emotion: Siemens). Target volumes and Organs at Risk (OAR) were drawn in accordance with ICRU 50. Both EA and IMRT plans were generated using the PLATO planning system (The Nucletron, Netherlands). For EA plans the aim was to cover the entire PTV with the 85% isodose line. For IMRT, 5-7 equally spaced coplanar isocentric beams were used. Constraints were set depending on the location of the tumor and OARs (Heart, Lung and Spine). Dose Volume Histograms (DVH) were calculated for PTV and OARs and compared between EA and IMRT. For PTV the following parameters were evaluated: $V_{95}$, $V_{107}$ i.e., percentage volume covering 95% and 107% of the prescription isodose respectively. $D_{max,}$ $D_{min\ and}$ $D_{mean}$ (Maximum, minimum and mean dose). $D_{min}$ and $D_{max}$ being the volume receiving 99% and 1% of the dose respectively. For OARs the following parameters were evaluated; $D_{max}$, $D_{min}$, $D_{mean}$, $D_{50,}$ $D_{30,}$ i.e., Maximum, minimum and mean dose, dose to 50% and 30% of the volume of the organ are evaluated. For normal tissue irradiation, low dose regions $D_{10}$ and $D_{30,}$ dose receiving 10% and 30% of the volume are evaluated.

**Results:** IMRT resulted in superior PTV coverage and homogeneous dose distribution compared to EA. The dose to spinal cord (SC) is more in EA compared to IMRT for PTVs that are posteriorly placed and close to the SC, whereas the dose to SC is high in IMRT compared to EA in the case where PTV is in the anterior chest wall. DVH of ipsilatral lung (ILL) can be divided into three parts viz low dose region ≤ 5Gy, medium dose region 5-30Gy and high dose region ≥ 30Gy. Dose received in EA is less compared to IMRT in the low dose region and in the high dose region. However in the medium dose region the dose in IMRT is less compared to EA. Dose received by the contralateral lung is less in EA compared to IMRT. Cardiac dose profile was similar to spinal cord. Normal tissues receive more dose in IMRT compared to EA.

**Conclusion:** IMRT has the potential to offer superior target homogeneity, conformity and reduction of high doses to critical structures compared to EA. However increased volumes of contralateral lung, spinal cord and ipsilateral lung and heart receive low doses. The effects of these low doses have to be studied systematically. Hence we have to use clinical judgment to choose the modality of the treatment and weigh the risks of irradiating the unknown against the benefits of delivering homogeneous dose to PTV and reducing the exposure to known critical structures.

### P-69

### Calculation of reference air-kerma rate calibration factors for $^{192}$Ir HDR sources using realistic energy spectrum

T Palani Selvam, KN Govinda Rajan, Reena Sharma, S Kannan. *Radiological Physics and Advisory Division, Bhabha Atomic Research Centre, Mumbai - 400 094, India. E-mail: pselvam66@yahoo.co.in*

**Introduction:** There is no primary standard existing for $^{192}$Ir sources. Reference air-kerma rate calibration factor of a given chamber at $^{192}$Ir energy, $N_{K,Ir}$ is derived based on measured response of the chamber ($N_k^{-1}$) as a function of photon energy (250 KV X-rays - 1.25 MeV). In

This PDF is available for free download from a site hosted by Medknow Publications (www.medknow.com)

[Downloaded free from http://www.jmp.org.in on Friday, July 29, 2011, IP: 130.14.254.25] || Click here to download free Android application for t

Table 1: Values of reference air-kerma rate calibration factor, $N_{k,Ir}$ for NE2561 and NE2571 chambers for different $^{192}$Ir HDR source models

| *Source type* | *Reference air-kerma rate calibration factor, $N_{k,Ir}$ (mGy/unit reading)* | |
|---|---|---|
| | NE2561 | NE2571 |
| Micro selectron PDR (old) | 9.432 | 41.171 |
| Micro selectron HDR (old) | 9.432 | 41.171 |
| Vari soure (new) | 9.432 | 41.166 |
| Vari source (old) | 9.432 | 41.166 |

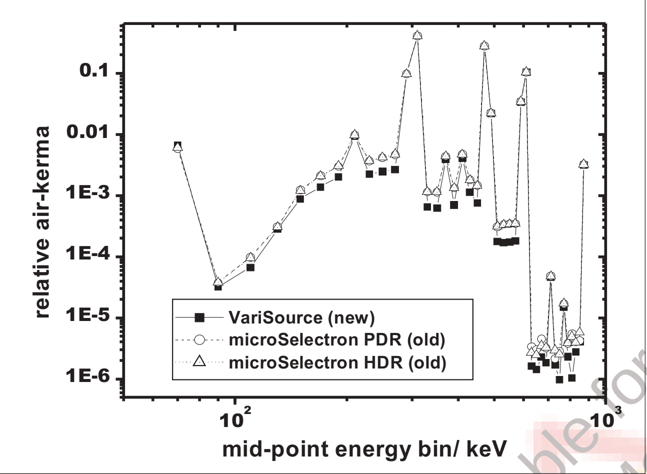

**Figure 1:** Relative air-kerma spectra of $^{192}$Ir HDR sources plotted as a function of mid-point of energy bins. The bin width is 20 keV

the present study we use realistic air-kerma spectra exiting from different models of $^{192}$Ir HDR brachytherapy sources while evaluating $N_{k,Ir}$ for NE2561 and NE2571 ionization chambers. For air-kerma spectra calculations, we used Monte Carlo-based MCNP code.

**Materials and Methods:** Air-kerma rate at 1 m in free space referred to as reference air-kerma rate, $K_R$ of an $^{192}$Ir HDR source using a reference ionization chamber can be determined as follows:

$$K_R = \frac{M(d_R)N_{k,ir}}{\Delta t} \quad (1)$$

where $M(d_R)$ is the electrometer reading due to source photons that reach the chamber at the reference distance, $d_R$ = 1 m along the transverse axis of the source and $\Delta t$ is the charge integration time. $M(d_R)$ is corrected for exponential attenuation of source photons in air, room-scatter and finite size of the chamber. The expression for determining $N_{k,Ir}$ is as follows:

$$N_{k,ir} = \sum_{i=1}^{n} K_i \left[\sum_{i=1}^{n} K_i (N_{k,i})^{-1}\right] \quad (2)$$

$$K_i = \varphi(E_i)\, E_i\, (\mu_{tr} / \rho)_{air,\, i} \quad (3)$$

where $N_{k,i}$ is the interpolated calibration factor for the $i^{th}$ spectral line (energy $E_i$) of the investigated $^{192}$Ir sources, $K_i$ is air-kerma in free space for photon for $i^{th}$ energy $E_i$, $\varphi(E_i)$ is the normalized fluence of photons of energy $E_i$ and $(\mu_{tr}/\rho)_{air,i}$ is mass energy transfer coefficient of air for photons with energy $E_i$.

**Monte Carlo calculation of air-kerma spectra:** Air-kerma spectra (at every 20 keV energy bin) needed for deriving $N_{k,Ir}$ were calculated at 100 cm along the transverse axis of the sources using the MCNP Monte Carlo code. These spectra are calculated for microSelectron HDR and PDR and new and old models of HDR VariSource $^{192}$Ir sources. The source designs of these sources are significantly different from each other. For a given source, the air-kerma values calculated in air medium or in free space are found to be statistically identical. This is because the exponential attenuation by air almost cancelled air scatter. Calculation of $N_{k,Ir}$: The $N_k$ factors of the NE2561 and NE2571 chambers as a function of photon energy were given $4^{th}$ order logarithmic polynomial and third order exponential fits, respectively. Using these functional fits, $N_{k,i}$ was calculated for a desired energy, $E_i$ ($E_i$ represents mid-point of the energy bin). Value of $N_{k,Ir}$ for each source and chamber was derived by using the values of $N_{k,i}$ and the Monte Carlo-calculated $K_i$ in equation (2). We also used bare $^{192}$Ir spectrum while evaluating $N_{k,Ir}$.

**Results and Conclusion:** Table 1 summarizes the $N_{k,Ir}$ factors obtained for NE2561 and NE2571 chambers for the investigated $^{192}$Ir sources. The value of $N_{k,Ir}$ obtained for NE2571 chamber when using bare spectrum is 0.14% higher in comparison to the values obtained using realistic spectra. Considering the uncertainties associated with the $N_k$ factors and fitting of these factors, this difference is insignificant. Figure 1 presents the air-kerma spectra obtained for the sources investigated. The shape of the spectrum is independent of the source model although the total air-kerma of each source was different from each other. It may be noted that the gamma lines 296, 308 and 316 keV of the $^{192}$Ir spectrum together constitute about 52% and the line at 468 keV constitutes about 21% of the total emission. Hence, the peaks are observed at energy bins 300-320 keV and 460-480 keV in the air-kerma spectrum. Due to dominance of these gamma lines the $N_{k,Ir}$ is insensitive to source model. For practical purposes we recommend $N_{k,Ir}$ = 9.432 and 41.170 mGy/unit reading for NE2561 and NE2571 chambers, respectively. These values may be used for any source type.

This PDF is available for free download from a site hosted by Medknow Publications (www.medknow.com)

### P-70

### Protective efficacy of rosemary extract against radiation-induced hepatic glycogen changes in mice

Dhanraj Soyal, Inder Singh, PK Goyal. *Radiation and Cancer Biology Laboratory, Department of Zoology, University of Rajasthan, Jaipur - 302 004, India.* E-mail: pkgoyal2002@rediffmail.com

**Introduction:** Ever since the human is born on this earth, he is

Table 1: Variations (mean ± S.E.) in glycogen content in liver of mice after exposure to different doses of radiation in the presence (experimental) or absence (control) of *Rosemarinus officinalis* extract (ROE)

| *Dose of radiation* | *Group* | *Post-treatment autopsy intervals.* 12 hrs. | 24 hrs. | $3^{rd}$ day | $5^{th}$ day | $10^{th}$ day | $20^{th}$ day | $30^{th}$ day |
|---|---|---|---|---|---|---|---|---|
| 3 Gy | C | 5.40 ± 0.595 | 5.87 ± 0.219$^c$ | 6.34 ± 0.211$^c$ | 7.00 ± 0.228$^c$ | 6.60 ± 0.213$^c$ | 4.67± 0.100 | 4.28 ± 0.110 |
| | E | 4.70 ± 0.317 | 4.94 ± 0.282$^a$ | 5.17 ± 0.599$^b$ | 6.18 ± 0.385$^c$ | 5.21 ± 0.280$^c$ | 4.53 ± 0.269 | 4.21 ± 0.054 |
| 6 Gy | C | 6.11 ± 0.351$^c$ | 7.05 ± 0.132$^c$ | 7.96 ± 0.381$^c$ | 8.65 ± 0.302$^c$ | 7.82 ± 0.263$^c$ | 4.92 ± 0.086$^b$ | 4.46 ± 0.078 |
| | E | 5.87 ± 0.878 | 6.58 ± 0.519 | 6.81 ± 0.710 | 7.83 ± 0.317$^a$ | 6.26 ± 0.221$^c$ | 4.64 ± 0.348 | 4.28 ± 0.127 |
| 9 Gy | C | 6.41 ± 0.515$^b$ | 6.62 ± 0.976$^a$ | 8.09 ± 0.209$^c$ | 9.40 ± 0.046$^c$ | 11.53 ± 0.057$^c$ | N.S. | N.S. |
| | E | 5.26 ± o.266 | 5.89 ± 0.075 | 7.08 ± 0.148$^b$ | 8.74 ± 0.241$^a$ | 10.99 ± 0.101$^c$ | 9.95 ± 0.129 | 8.70 ± 0.105 |

C= Control (DDW + radiation), Statistical comparison, significance level, E= Experimental (ROE + radiation) Normal v/s Control $^aP < 0.05$, N.S.= No survival of animals. Control v/s Experimental $^bP < 0.01$, $^cP < 0.001$

[Downloaded free from http://www.jmp.org.in on Friday, July 29, 2011, IP: 130.14.254.25] || Click here to download free Android application for t bombarded by ionizing radiation. Everybody on this earth irrespective of his place of stay and occupation is getting some dose of ionizing radiation which exists all around us. Protection from radiation effects through chemicals is in practice for past several years. Many chemical compounds have been and are being tested for their radioprotective effects in mammals (2-MPG, WR-2721, deoxyspergualin etc.). However their success has always been limited owing to their toxicity and side effects at their optimum dose. Recently, interest has been generated to search the potential drug of plant origin that are capable of modifying radiation response with minimum or no side effects. *Rosemarinus officinalis*, commonly called as rosemary belonging to family labiateae, is known for its different medicinal uses. Aim of present study is to evaluate the radioprotective effects of *Rosemarinus officinalis* leaves extract (ROE) against radiation-induced hepatic injury in mice.

**Materials and Methods**: Healthy Swiss albino mice (6-8 weeks old) selected from an inbred colony maintained under standard conditions of temperature and light (light: dark, 10h: 14h) were divided into 2 groups. Animals of Group-I were given double distilled water (DDW equal volume of ROE) to serve as control; whereas animals of Group-II were orally administered ROE at the dose of 1000 mg/ kg b. wt./ day for 5 consecutive days to serve as experimental. 30 minutes after last administration of DDW or ROE, animals of both the groups were whole-body exposed to 3, 6 and 9 Gy gamma radiation. These animals were autopsied between 12h to 30 days post-treatment and homogenate of liver was used to determine the glycogen content. Lipid peroxidation (LPx) and glutathione (GSH) were also estimated adopting standard procedures.

**Results:** Mice exhibited the symptoms of radiation sickness in both the groups but their magnitude was observed lesser in experimental group. The morphometric or pathological symptoms were rare in experimental group and such animals were found to be quite healthy and active than control animals. Biochemical estimation revealed that an increase in glycogen content occurred at all the doses of radiation, both in the drug treated irradiated and non drug treated irradiated animals. However, this increase was significantly lesser in the drug treated irradiated animals. An early return to normal level of glycogen was recorded in ROE pretreated irradiated mice of 3 and 6 Gy groups as compared to control. After exposure to 9 Gy, non-drug treated animals could not survive more than 10 days; whereas drug treated irradiated animals survived till 30 days, however, normal value of glycogen could not be restored by the end of experimentation. A significant increase in LPx level and decrease in GSH level were noted in blood and liver of irradiated animals; whereas ROE pretreated irradiated mice exhibited a significant fall in LPx and rise in GSH level.

**Conclusion:** The present study suggests radioprotective effects of leaves extract of *Rosemarinus officinalis* (ROE) against radiation-induced glycogen changes in liver of Swiss albino mice. ROE treatment also inhibits LPx and increases GSH level after irradiation.

### P-71
### Objective of personnel monitoring to medical radiation workers

P Padma Savitri, MK Kamble, Madhumita Roy, KS Reddy, MM Adtani.
*TLD Unit, PM and DRS, R.P.A.D, Hyderabad, India*

**Introduction:** Personnel monitoring service for radiation workers is the continuous measurement of an individual exposure dose. This is accomplished via the use of TLD badge.

**A TLD badge based on $CaSO_4$:** Dy Teflon discs has been designed and is in regular Personnel monitoring use since 1975. The TLD badge has shown satisfactory performance for monitoring beta, gamma and X-ray doses of medical radiation workers. The complete Personnel Monitoring TLD badge consists of a TLD card and a plastic cassette with multiple filters for holding the TLD card. The badge is affixed to the clothing of a person with the help of a crocodile clip attached to the badge. This paper discusses the objective of providing monitoring services to medical radiation workers. Also the paper elaborates the service procedure to get registered under personnel monitoring services and discusses the responsibilities of workers and employers in smooth functioning of services.

**Service Procedure:** In general, all occupationally exposed personnel whose radiation exposure may exceed 10 percent of the annual allowable dose shall participate in monitoring program. On receipt of a request from an Institution desirous of availing of the TLD Personnel Monitoring Service, TLD section will send a copy of the application form (PMS-TLD/2) and a format of the Personal Data form. The TLD/2 application form and Personal Data Form for all the persons to be monitored should be filled in and returned to Dose Registry Group, BARC. Depending upon the number of radiation workers, radiation work and the sources being handled, the institution will be informed about the total number of TLD badges. Institution and all radiation workers to be monitored will be allotted an Institution number and Personal numbers respectively which will be intimated to the user institution. A TLD card and holder to each person will initially be issued. For medical institutions the service is provided on quarterly basis. Thereafter a new card will be issued for next service period. Used Cards should be forwarded to the TLD Units for processing and evaluating dose. Dose reports based on doses evaluated by TLD units are prepared and sent to institutions periodically. Dose reports contain current period doses and upto date dose of the current year. An individual centralized lifetime dose data of all radiation workers in the country is maintained in RPAD, BARC. Our goal is to ensure safe working conditions for radiation workload of occupational exposure well within the limits stipulated by AERB. Atomic Energy Regulatory Board (AERB) has laid down dose limits to be followed in our country. The Effective Dose (whole body dose) constraint for the five years period is 100 mSv i.e., average 20 mSv per year in this 5 years block. In any one-calendar year in this period the individual effective dose (whole body dose) shall not exceed 30 mSv. Annual equivalent dose limit for the skin, the hands and feet is 500 mSv and for the lens of eye 150 mSv. However it is strongly recommended to control and minimize the individual doses to as low as possible by following good work practices. The present practice is that all the individual whole body doses exceeding 10 mSv in a service period is considered as over exposure and such cases are investigated by committee designated for this purpose. No special consideration for women radiation workers, when pregnancy has been diagnosed dose should not exceed 2 mSv during the remaining period of pregnancy. The institution should arrange investigate the causes of the overexposure and report the findings to TLD Unit immediately in the prescribed format and should take preventive action to avoid such exposures in the future. Persons receiving more than 100 mSv have to be subjected to hematological examinations including differential blood counts and are likely to be called for chromosomes aberration test at BARC. After receiving the investigation reports from the institutions the overexposure cases are reviewed by over exposure committee in BARC and advice on necessary follow-up action to the concerned institution.

### P-72
### Study of overexposure cases among radiation workers in medical institutions of Andhra Pradesh

P Padma Savitri, MK Kamble, Madhumita Roy, KS Reddy, Geetha V*.
*TLD Unit, PM and DRS, R.P.A.D, Hyderabad, *PM and DRS, R.P.A.D, B.A.R.C, Mumbai, India*

**Introduction:** The Personnel monitoring technique for external radiation exposure strictly follows the International Commission on Radiation Protection (ICRP). Based on that Atomic Energy Regulatory Board (AERB) has laid down dose limits to be followed in our country. The cumulative effective dose constraint for any five-year block is 100mSv, i.e., average 20mSv per year. However during any single year, a person should not exceed 30mSv. If the dose recorded by TLD badge in a monitoring period is 10mSv or above, it is considered as over exposure. TLD unit at Nuclear Fuel Complex, Hyderabad is providing Personnel Monitoring for External radiation to about 4000 radiation workers from DAE, Medical, Research and Industrial institutions of Andhra Pradesh using TLD badges. Out of these some industries are served on monthly basis and others are served quarterly. There are

This PDF is available for free download from a site hosted by Medknow Publications (www.medknow.com).

[Downloaded free from http://www.jmp.org.in on Friday, July 29, 2011, IP: 130.14.254.25] || Click here to download free Android application for t

**Table 1: Number of overexposure cases in medical institutions**

| Year | No. of persons monitored | No. of over exposure cases | Genuine cases | Non-genuine cases |
|---|---|---|---|---|
| 1999 | 922 | 8 | 7 | 1 |
| 2000 | 1042 | 9 | 5 | 4 |
| 2001 | 1019 | 6 | 3 | 3 |
| 2002 | 1303 | 18 | 2 | 16 |
| 2003 | 1377 | 9 | 4 | 5 |
| 2004 | 1640 | 24 | 10 | 14 |

**Table 2: Number of overexposure cases in various departments of medical institutions during 1999-2004**

| Department | Genuine cases | Non-Genuine cases |
|---|---|---|
| Diagnostic X-ray | 28 | 38 |
| Radiotherapy | 3 | 4 |
| Nuclear medicine | - | - |
| CC ray | - | 1 |

about 200 registered medical institutions in this state, out of which 70% have diagnostic X-ray facility whereas remaining 30% includes other facilities like Nuclear medicine, brachytheraphy, beamtherapy, cath lab etc. About 1600-registered medical radiation workers in the above mentioned facilities are being monitored on quarterly basis. This paper presents the cases of overexposure in Medical institutions for the five year block 1999-2003 and also for the year 2004. The objective of the follow-up of overexposure is to have effective control over the doses received by the radiation workers, minimizes the instances of causing non-genuine exposure and also to avoid recurrence of such cases and ensure the safe working conditions.

**Materials and Methods:** The exposure cases where equivalent dose of 10 mSv and above has been recorded by the dosimeter during a monitoring period are considered overexposure cases and are intimated to the Head of the institution by fax for prompt investigation. It is also intimated to Member Secretary, Overexposure Investigation Committee and Head, Personnel Monitoring Division with details like pattern of card readings, control reading and glow curves. The local Institutional safety committee consisting of head of the institution, medical physicist or radiological safety officer investigates the reason for the overexposure and report their finding to Member Secretary, Overexposure investigation committee for receiving advice regarding the remedial and follow-up measures. A committee constituted by Chairman, Atomic Energy Regulatory Board, which has experts from AERB and BARC, analyses the circumstances under which overexposure has occurred, taking into consideration the following data: 1) investigation report received from the institution as well as the statement of the individual. 2) Chromosome aberration test report if any (normally CA test is carried out when a person is reported to have recorded a dose in excess of 100 mSv) and 3) the pattern of the readings of the discs of TLD card. The committee decides whether case is genuine or non-genuine and recommends necessary steps to be taken to minimize future exposures including restrictions on the radiation work carried out in the institution, any lay off of worker from radiation work or to carry out radiological protection survey to assess the work practices and working conditions.

**Study of Overexposure cases:** Table 1 gives the overexposure cases during the 5 years block 1999-2003 and the year 2004 for medical institutions in Andhra Pradesh. It indicates 41% of the total 74 cases are genuine and 59% are non-genuine cases. Table 2 indicates the number of genuine and non-genuine overexposure cases in various medical practices. It can be seen from the table that 90% of the cases are found in X-ray diagnostic departments. Table 3 represents the number of genuine and non-genuine over exposure cases in different dose levels. Table also gives comparison of Cases in Medical institution with total cases from Industrial, Medical and Research institutes. It can be seen that 75% of the total cases are from Medical Institutions. Out of which 87% of genuine cases are below 30 mSv where as 55% of non-genuine cases are below 30 mSv in any single year. This indicates more non-genuine cases are in higher range of dose mainly due to negligence in the usage of dosimeter.

**Conclusion:** The reporting, investigation and follow-up of overexposure cases helps in effective control of doses to the radiation workers. It is also emphasized that in case of any unusual incident the TLD cards should be sent for urgent processing to Personnel Monitoring Section to know the dose to the worker at the earliest for effective control of individual exposure and safe working conditions.

**Acknowledgements:** Authors wish to thank Dr. R.K. Kher, Head, PMDRS and Shri S. Kannan, Head, RPAD for their necessary guidance and encouragement to carry out this work.

**P-73**

**Forward IMRT planning of conservative breast**

Vinod Pandey, Alok Kumar, Girigesh Yadav, Gautam Mukherjee, Arundhati Chakraborty, Kalyan Bhattacharya. *AMRI Hospitals, Kolkata, India*

**Introduction:** Breast cancer is the most commonly diagnosed cancer in women and majority of the cases are treated with breast-conserving surgery followed by Radiotherapy. Radiotherapy is an effective

**Table 3: Number of radiation overexposure cases (≥10 MSV) in medical and other institutions of Andhra Pradesh during 1999-2004**

| Year | Dose levels→ | 10-19.99 (msv) | 20-29.99 (msv) | 30-39.99 (msv) | 40-49.99 (msv) | 50-99.99 (msv) | >100 (msv) |
|---|---|---|---|---|---|---|---|
| | Institutions | G ng | G ng | G ng | G ng | G ng | G ng |
| 1999 | Med | 5 (1) | 1 | 1 | | | |
| | Total | 8 (3) | 2 | 3 | | | |
| 2000 | Med | 4 | 1 (1) | - | 0 (1) | | 0 (2) |
| | Total | 6 (2) | 1 (1) | | 1 (1) | | 0 (2) |
| 2001 | Med | 1 (2) | 0 (1) | - | - | 1 | 1 |
| | Total | 2 (2) | 0 (1) | | | 1 (1) | 1 |
| 2002 | Med | 2 (7) | 0 (2) | 0 (1) | - | 0 (1) | 0 (5) |
| | Total | 3 (7) | 2 (2) | 0 (1) | | 0 (1) | 0 (5) |
| 2003 | Med | 1 | 2 | | | 1 | 0 (5) |
| | Total | 4 | 2 | 0 (1) | | 1 | 0 (5) |
| 2004 | Med | 10 (5) | 0 (4) | | | | 0 (5) |
| | Total | 13 (5) | 0 (4) | | | | 0 (5) |

G: Genuine cases. Ng: Non genuine cases. Total indicates industrial, medical and research institutions

This PDF is available for free download from a site hosted by Medknow Publications (www.medknow.com).

[Downloaded free from http://www.jmp.org.in on Friday, July 29, 2011, IP: 130.14.254.25] || Click here to download free Android application for t treatment modality for the treatment of breast cancer and widely practiced all over the world. The conventional two tangential wedged fields can result in a heterogeneous distribution which can result poor cosmetic result in many cases of breast conservative radiotherapy after irradiation. In an attempt to improve the dose distribution homogeneity in case of breast conservative radiation therapy, many techniques has been developed. In this article we will focus on static multi-segment forward IMRT planning for conservative breast for achieving the more homogeneous dose distribution in the breast and minimize the doses to the heart, lung and contra lateral breast.

**Materials and Methods:** 15 patients were selected for this study all of them has been treated previously in our institute. Patient lied down supine on a breast board with their arms placed above the head in an armrest. All necessary landmarks have been noted on the positioning sheets. A 5 mm thick and 5 mm spacing CT slice were taken by our spiral GE CT machine and the images are sent to the Oncentra contouring and virtual simulation system.

**Volume:** First, using auto countouring tool of oncentra contouring system delineated the skin surface and both the lung. Entire breast tissue of the interest is delineated as a PTV. left lung,right lung and heart and a part contra lateral breast has been delineated as organ at risk.

**Treatment Planning:** The appropriate gantry angle, collimator angle and field border virtually simulated using the CT-data and the given volume and a 3D plan was developed for each patient, by using 6MV beam of our Elekta Linac. Saved plan is exported to Nucletron PLATO Sunrise 3D Planning system for dose calculation and to see the dose distribution. Following steps were taken to do the forward IMRT planning. Same plan is imported at Plato Sunrise 3D planning system. At first 2 tangential open beam with equal weight, calculation was done for a dose of 50.4Gy in 28 fraction (five fraction per week) then isodose lines are seen in traverse coronal and sagittal slices, area of higher isodose and lower isodose lines are noted in all the sections, then a number of segmented tangent field beams with different weight were iteratively created to reduce the "hot spots" produced by 2 tangential field.

**Plan evaluation:** each patient plan was evaluated by calculating differential DVH and compared with the conventional wedge plan by the following parameters. 1. V105-Volume receiving more than 105% of the prescribed dose. 2. V110-Volume receiving more than 110% of the prescribed dose. 3. Max-dose or hot spot.

**Conclusion:** The forward IMRT technique offers improvement in target dose homogeneity by reducing the volume getting higher doses and minimizes doses to the critical structure (up to little extent) as compared with conventional wedge planning.

This PDF is available for free download from a site hosted by Medknow Publications (www.medknow.com)

**P-74**

**Design and characterization of total skin electron beam irradiation facility with high energy linear accelerator**

KV Rakesh, NS Shine, Mathew Joby, Prasad P Satheesh, Bhaskaran K Pillai. *Department of Radiation Oncology, Amrita Institute of Medical Sciences, Kochi, India*

**Introduction:** Electron energy ranging from 2-6 MeV from linear accelerators are used for treating superficial lesions covering large areas of the body, such as Mycosis Fungoides. At these energies electron beams are characterized by a rapid falloff in dose beyond a shallow depth and minimal X-ray background (1% or less). The physical principle underlying these techniques is to use a Perspex beam spoiler of 8 mm to 10 mm thickness at large distance from the isocentre and degrade a 6-8 MeV (HDRE Mode) electron beam to obtain a low energy, less penetrating beam of large field size. Low Z scattering material, Perspex produces a shower of degraded energy electrons at the patient's skin level, resulting from collision of primary electrons. We have standardized the technique of irradiation for TSET at Amrita Institute of Medical Sciences at Kochi, the technical and physical aspects of the setup are highlighted.

**Materials and Methods:** 6 MeV electron beam from Elekta Precise LINAC in high dose rate mode (3000 MU/min at isocentre), along with the special electron applicator was used. Acrylic sheets of total thickness 10 mm were mounted on a frame of 2.00 m x 1.00 m size. The frame is kept at 2.75 m from isocentre with a clearance of 1.0 m from the distal wall. A distance of 25 cm from the Perspex degrader is planned to provide a Focus Skin Distance (FSD) of 3m. The single field beam flatness was measured using PPC 40, the detector being moved in the vertical and horizontal planes at 1 mm intervals with the help of PTW RFA motor assembly. Electron beam energy was assessed using a solid water phantom, size 30x30x15 cm, plane parallel chamber (PPC 40) embedded in this and sheets of varying thickness 1mm to 10 mm were added. From the depth dose curve, the range values Rp, R50 were measured and electron energies Ep,o and Eo were calculated. The absorbed dose in Gy/MU was measured by keeping the front wall of parallel plate chamber at 1 mm depth in solid water phantom. Correction factors 1) $K_Q$, $K_{ecal}$ (TG-51) 2) $K_{TP}$ 3) polarity 4) recombination 5) leakage were applied for the ionization readings to arrive at the absorbed dose. The absorbed dose/ MU is also calculated by doubling the corrected output of 25x25 cm at 100 FSD of 6 MeV beam 1.053 cGy/ MU applying inverse squarer law 1 / (3.0x3.0), assuming that the electron intensity gets doubled, when one primary electron produces another additional secondary electron by collision in the Perspex beam spoiler material.

**Results:** The full width at half maximum (FWHM) of the beam flatness curve was 140 cm, which indicates 70 cm useful field on either side of the central axis. From calculations it was found that two electron beams with a tilt of $18^o$ on either side of $270^o$ position of the gantry, gives uniformity at the overlap edges of the useful beam. This facilitates a flat degraded electron field of 140x100 cm, sufficient for TSET. The Ep, o and Eo of the degraded beam were 3.39 MeV and 2.32 MeV respectively. The Bremsstrahlung background was found to be less than 0.4% for the horizontal beam. The measured absorbed dose/MU for the horizontal beam was 0.1092 cGy/MU. This is designated as $D_H$, the dose/MU from single horizontal beam. The calculated dose rate at the position of the patient by the proposed hypothesis in this study was 0.1147 cGy/MU agreeing within 5%. Further work has to be done to apply this $D_H$ value for dose delivery with Stanford technique using 6 pairs of electron fields. This arrangement is used for the clinical application of TSET at our center.

**Discussion:** Techniques for TSET vary in different radiotherapy centres depending on the local irradiation geometries. Therefore standardization of technique and dosimetry becomes essential according to individual circumstances. 6 field Standford Technique is followed in most of the radiotherapy clinics over the last 4 decades, but even now detailed outlook on the dosimetry technique followed at each centre becomes worthwhile and informative for a new centre to introduce the technique. Therefore we felt that the above presentation is necessary. Anacak *et al* have demonstrated from *in vivo* dosimetric data of 67 TSEI treatments that overall dose homogeneity throughout the skin surface of the body is around + 15% compared to the + 5% variations in the idealised situation with humanoid phantom measurements. They suggested that deviations in the extratrunk parts (moving extremeties) are relatively high compared to the trunk regions. In our earlier work (Ravikumar and Ravichandran, 1999) the treatment distance suggested was 4.6M with $8^o$ angulation of tilted pair of beams. The Ep, o and Eo measured for the electron beam were 3.09 MeV and 2.18 MeV respectively for the Clinac 1800 linear accelerator. The observed dose uniformity with humanoid phantom was within 10%. Present work has shown $12.2^o$ to achieve uniformity in the beam intensity due to pair of fields.

**References**

1. AAPM Report No.23. TSET technique and dosimetry. AIP Pubs: NY; 1988.
2. Khan FM. Phys Radiat Ther 1999. p. 411-2.
3. Ravikumar M, Ravichandran R. J Med Phys 1999;24:11-4.
4. Anacak Y, Arican Z, Bar-Deroma R, Tamir A, Kuten A. Total skin electron irradiation: Evaluation of dose uniformity throughout the skin surface. Med Dosim 2003;28:31-4.
5. Gossman MS, Sharma SC. Total skin high-dose-rate electron

[Downloaded free from http://www.jmp.org.in on Friday, July 29, 2011, IP: 130.14.254.25] || Click here to download free Android application for t therapy dosimetry using TG-51. Med Dosim 2004;29:285-7.

**P-75**

**Texture analysis of the digitized conventional radiographs in the evaluation of osteoporosis compared with quantitative ultrasound bone densitometer**

R Karunanithi, S Ganesan*, TMR Panicker, A Govindan, M Paul Korath, K Jagadeesan. *K J Hospital Research and Postgraduate Centre, Chennai - 600 084, *Anna University, Chennai - 600 025, India*

**Introduction:** Osteoporosis is a widespread medical condition that affects millions people in India and globally and accounts for about 1.3 million new fractures per year in people older than 45 years. This condition is more prevalent in women and in the elderly. Osteoporosis manifests as a loss in bone mass, a tendency toward fracture and as a structural alteration of bone. The trabecular bone represents 20% of the skeletal mass and 80% is cortical bone. Trabecular bone is less dense, more elastic and has a higher turnover rate than cortical bone. Normal trabecular bone resembles a honeycomb, consisting of a highly connected network of vertical and horizontal plates, called trabeculae. The osteoporotic trabecular bone shows apparent replacement of plates by rods and obvious trabecular disruption, giving rise to the view that loss of connectivity is an important component of skeletal fragility in osteoporosis. Application of textural parameters in the evaluation of osteoporosis using digitized conventional radiographs of the calcaneum and comparison with the broadband ultrasound attenuation (BUA), determined by quantitative ultrasound bone densitometer at the calcaneum.

**Materials and Methods:** 104 subjects (Female 65 and Males 39) of age group 35 and above were included in the study undergo and the Broad band Ultrasound Attenuation (BUA) by Quantitative Ultrasound Bone Densitometer (QUS) (CUBA PLUS) The QUS basically measures how much sound is attenuated by the bone in dB $MHz^{-1}$. Radiographs of the calcaneum (Lateral View) for all the subjects were taken at fixed KV and mAs. The radiographs are digitized with the help of a 16-bit digitizer available at the Indira Gandhi Centre for Atomic Research (IGCAR) Kalpakkam and stored as TIFF images for analysis. A clinically significant Region of Interest (ROI of 256 x 256 Pixels) was selected for analysis. An algorithm developed by us in Matlab7 ($14^{th}$ Release) was used for extracting 12 textural parameters from co-occurrence matrix. The elements of the co-occurrence matrix represents how often pairs of pixels occurs with values of the same gray level. The subjects were divided into three groups based on QUS Bone Densitometer, Group-I Normal, who's T-Score greater than -1, Group-II (physiological loss of calcium) Osteopenic who's T-Score between -1 and -2.5 and Group-III (abnormal loss of calcium) Osteoporotic who's T-Score below than -2.5. From the twelve parameters, three parameters namely Entropy, Triangular Symmetry and Diagonal Moment gave a significant difference in their mean values, which is found to be statistically significant. The Results obtained are shown in the table.

**Conclusion:** The results evaluated from the textural measurement shows that the above values increases steadily with the T-Score evaluated by Ultrasound Bone Densitometer in the sizable population study. This may have a clinical application in the evaluation of osteoporosis from a simple calcaneal radiograph digitized with a film scanner, which can also be used as a screening tool for osteoporosis. These texture parameters are to be standardized with the Dual Energy X-ray Absorptiometry (DEXA) that has been considered as 'Gold Standard' in the measurement of osteoporosis and the work is undergoing. The method was developed for the classification of bone images involving trabecular structures present in the radiographs. There are still other textural analysis techniques, which may be applied to the experiment where image thresholding and application of the second order statistical parameters are involved.

**Table 1: Entropy, triangular symmetry and diagonal moment for three different groups**

| Groups | Mean age + STD | Qus T-score | Entropy | Triangular symmetry | Diagonal moment |
|---|---|---|---|---|---|
| Group-I n = 47 | 52 + 12 | Sd > -1 | 9.723 | 0.672831 | 1089 |
| Group-II n = 28 | 60 + 8 | -1 > sd > -2.5 | 9.797 | 0.6996375 | 1094 |
| Group-III n = 29 | 56 + 11 | Sd < -2.5 | 9.824 | 0.707479 | 1183 |

**P-76**

**Absolute output variation with QI and PDD measurements: An attempt to achieve the optimize dosimetry condition for clinical use**

VP Choube, B Sarkar, HV Ghare, MA Laviraj, A Deshmukh, S Choudhari. *Dept. of Medical Physics, R.S.T Regional Cancer Hospital, Manewada Road, Nagpur - 27, Maharastra, India. E-mail: biplabphy@yahoo.com*

**Introduction:** Measurement of the absolute output is one of the most crucial factors in radiotherapy. The recent TRS 398 recommended method of determination of absolute dose in water for clinical beam, at user end, in reference condition is at reference depth ($Z_{ref}$). But this method requires the percentage depth dose (PDD) value in the reference depth for output determination. As a recommended practice, in hospital level, the output measurement is performed in 30x30x30 $cm^3$ fixed grooved water phantom. It is very difficult to measure the PDD on such phantom accurately, hence standard set of PDD values are evaluated by measuring in radiation field analiser (RFA) (48x48x48 $cm^3$ water phantom). In this present study we have tried to find out the variation in output for standard 10x10 $cm^2$ Field size for 6MV (QI = 0.676) and 15MV (QI = 0.756) X-ray beam of Elekta Precise Linear Accelerator for following conditions: 1) Due to variation in beam quality index (QI). 2) When measured in standard phantom 30x30x30 $cm^3$

| Beam energy | Calculated QI (from PDD) | Output (cGy/mu) due to calculated QI (a) | Measured QI (from TMR) | Output (cGy/mu) due to measured QI (b) | % variation {(a-b)/a} x 100 |
|---|---|---|---|---|---|
| 6MV | 0.6765 | 0.9924 | 0.6768 | 1.0023 | -0.01% |
| 15MV | 0.7577 | 1.0024 | 0.7577 | 1.0019 | -0.05% |

| No. of measurement | Output measured in standard phantom (30x30x30 $cm^3$) | | Output measured in RFA | |
|---|---|---|---|---|
| | 6Mv O/P (cGy/mu) | 15Mv O/P (cGy/mu) | 6Mv O/P (cGy/mu) | 15Mv O/P (cGy/mu) |
| 1 | 0.9854 | 0.9484 | 0.9986 | 0.9899 |
| 2 | 0.9752 | 0.9523 | 1.004 | 1.0061 |
| 3 | 0.9705 | 0.9466 | 0.99696 | 1.0018 |
| 4 | 0.9856 | 0.9499 | 0.9942 | 1.0030 |
| 5 | 0.9764 | 0.967 | 1.0076 | 0.9957 |
| 6 | 0.9966 | 0.962 | 0.9976 | 0.9927 |
| 7 | 0.9924 | 0.9634 | 0.9993 | - |
| 8 | 0.9736 | 0.9532 | 0.9928 | - |
| 9 | 0.9814 | - | - | - |
| % Variation ={[(max-min)/max] x 100%} | 2.6% | 2.1% | 1.5% | 1.6% |
| Standard deviation (s) | 0.00295 | 0.00306 | 0.00173 | 0.00258 |

[Downloaded free from http://www.jmp.org.in on Friday, July 29, 2011, IP: 130.14.254.25] || Click here to download free Android application for t and a standard PDD set is used. 3) When output measured in RFA and PDD is also evaluated in the same setup.

**Materials and Methods:** The standard set of PDD is measured during commissioning or after replacement of any relevant items. The QI is being obtained either from PDD, by the formula given in TRS 398 or can be evaluated from measured TMR values. The PTW semiflex (active vol 0.125 cc) and RFA is used for depth dose (PDD/TMR) measurement and PTW Farmer (active vol 0.6 cc) chamber is used for Absolute dose evaluation in standard phantom and RFA.

**Results:** Table 1.

**Conclusion:** The variation on absolute output due to variation in QI, if calculated from measured PDD or evaluated from measured TMR is negligible. Either mode of determining QI may be preferred in routine dosimetry practice. It has been found that the absolute output varies of the order of ~1.5% when evaluated in RFA and ~2% to 2.5% when evaluated in recommended phantom by using standard set of PDD value. In order to achieve the better accuracy in dose measurement at user end measurement in BARC recommended 30x30x30 $cm^3$ backed by measured PDD in same setup in RFA should be preferred. The recommended water phantom is unable to provide accurate dose for all field size for clinical need, hence the relative as well as absolute dose measurement are needed to be performed in RFA. Thus to match the recommended conditions and clinical need measurement done in RFA backed by Dose to water measurement done in recommended phantom is suggested. Normalization of RFA absolute and relative data with that of absolute dose measurement in recommended condition where $N_{DW}$ is provided may lead to achieve the accuracy in overall data of clinical use.

### P-77

### Ushering in eGovernance in radiation safety surveillance in India with RSDIS

A Balasubramanian, PK Dash Sharma*, SP Agarwal*. *Softmasters, Off. No. 29, Plot 17, Sector-8, Nerul, Navi Mumbai - 400 706, *Radiological Safety Division, Atomic Energy Regulatory Board, Niyamak Bhavan, Anushaktinagar, Mumbai - 400 094, India*

**Introduction:** A software RSDIS has been developed by M/S Softmasters, Mumbai to enable the radiation user institutions submit their application forms for various regulatory clearances, periodic safety status reports etc. to Atomic Energy Regulatory Board (AERB) online using the Internet and to help the AERB officials to perform their task with regards to issuance of regulatory clearances, surveillance activities etc. faster.

**Design Considerations:** As per the statutory requirements the user institution has to provide various details about their infrastructure, radiation facilities, staff and equipments etc. at the time of procuring new equipments/radiation sources and also provide working details on a periodical basis to Radiological Safety Division (RSD), AERB. The forms containing these details have to be completed diligently and in time for periodic reports. This form filling procedure can take away their precious time. Similarly AERB officials, while issuing regulatory clearances, need to refer the latest details of the user institution, for verifying their compliance with the rules on several aspects e.g., the status of their radiation equipments, measuring equipments, presence of qualified staff, radiation protection aspects, radiation incidents etc. With the enhanced growth of radiation-based services in the recent years in the fields of medicine, industry and research in India, there has been manifold increase in the workload of RSD, AERB in checking the compliance with respect to various stipulations and in secretarial work in preparing, sending and retrieving the required documents. This results in the reduction of their precious time for development work, training, inspection etc. Hence, a proposal to use the services of ubiquitous IT was contemplated to redress the problems of both the Radiation user Institutions in their data submission and the AERB officials in utilizing these data. The main goals of this computerization are 1. To maintain a comprehensive national database of all Radiation Equipment and Instrumentation data and Radiation users' permanent and temporal data. 2. Capture the relevant portion of this database pertaining to the users directly through the Internet from the online submissions of various statutory forms. 3. Automate the procedures needed by the AERB officials in verifying the compliance of rules by the user institutions to avoid any delay. 4. Deploy the database to analyze the trends of radiation usage so that AERB can help the radiation user community on a long-term basis.

**Database Considerations:** The RSD Database typically consists on-the-one-hand common, less volatile details on various type-approved makes and models of radiation equipments, measuring equipments, suppliers, techniques and the dosages used in studies, radiation pharmaceuticals etc. say (RSD Master Data). On the other hand RSD Database also consists another set of information which are dependent on the user institutions like the actual equipment deployed, the staff members, their qualifications, number of sources in their possession, equipment maintenance dates, instrument calibration dates, patient workload, inspection dates etc. And there are partitions in the database as per the application area e.g., Radiotherapy, Nuclear Medicine, Diagnostic X-ray and other industrial areas. Apart from the standard features of data integrity, reduction of redundancy the RSD database is designed to obviate obsolescence since the radiation data collected is of national, long-term significance.

**Associated Software Considerations:** The software has been designed in two parts namely Webhosting and In-house Application. Webhosting part of the software is meant for capturing the data from the institutional users after security verification. The facilities provided for the form submission, include: 1. Downloading the previously submitted relevant data and edit and reuse them 2. Drop-down lists of the instruments and manufacturers details etc. from the RSD master data 3. Provision to upload figures and documents 4. Inbuilt help for filling each item 5. Tracking the status of submission at RSD end 6. Forms can be completed in parts as per the convenience of user institution In-house application part of the software helps the AERB officials to 1. Generate letters, emails, mass mails to the radiation user institutions 2. Generate summary and trend reports using current and historical data.

**Conclusion:** With all these IT facilities provided by the first version of RSD Database and Associated software system called RSDIS, the authors feel that the era of eGovernance has been ushered in the area of Radiation Safety surveillance. The In-house application part RSDIS is already installed in AERB and are being used by AERB Officials and the Webhosting part is being tested.

### P-78

### Computer based screening test

Mitali Das, Tiwari TN. *Department of Physics, National Institute of Technology, Rourkela - 769 008, India. E-mail: das.mitali@rediffmail.com*

**Introduction:** Noise may be considered as any unpleasant or disturbing sound. It is well known that factories and workshops tend to be quite noisy due to the noises and vibrations arising from the operations of various machines. Noise levels in the work areas of even production industries may be as high as those found in typical workshops. However, there may be quiter areas at locations far away from the noise sources in the case of production industries. But even at such relatively quiter sites, the noise levels may still be above speech interference levels or even potentially harmful levels. Long term exposure may cause temporary or permanent hearing loss. Most early noise-induced hearing losses pass unnoticed unless they are detected by appropriate hearing tests. This type of test is generally referred to as an audiometric examination and it tests the hearing acuity. It is performed with an instrument known as an "audiometer". An audiometer is essentially an oscillator driving a pair of headphones and is calibrated noise sources and bone-conductor vibrator. Audiometers may be divided into two mail groups on the basis of the type of stimulus they provide to elicit auditory response: pure-tone audiometers and speech audiometers. Pure-tone audiometer is used primarily to obtain air-conduction and bone- conduction thresholds of hearing. These thresholds are helpful in diagnosis of hearing loss. Pure tone screening tests are employed extensively in industrial and

This PDF is available for free download from a site hosted by Medknow Publications (www.medknow.com).

[Downloaded free from http://www.jmp.org.in on Friday, July 29, 2011, IP: 130.14.254.25] || Click here to download free Android application for t school hearing conservation programs. Speech audiometers are normally used to determine speech reception thresholds for diagnostic purposes and to assess and evaluate the performance of hearing aids. Existing audiometers can be used to test the hearing loss of only one person at a time. If the tests are to be conducted on a large number of people, the time consumed is very large. To overcome this difficulty, a microprocessor-based mass-screening audiometer can be used. The objective of the audiometric measurements using computer-based system is to conduct a quick and at the same time credible assessment of the hearing sensitivity of individuals. The system solves the problem of making audiometric measurements easily accessible for the public by providing cost effective solutions based on the software for testing hearing installed in local personal computers or in network servers.

**Materials and Methods:** The concept of screening examination presented in this paper belongs to both questionnaires and measurement test. Due to the growing epidemiology of hearing impairments and the resulting demand for hearing loss prevention, audiometric testing needs to be done in schools, in local health centers and other healthcare and medical institutions as well as in work places exposed to noise risk. As the personal computer is becoming more and more common and given the fact that it has the capacity to read data recorded on optical, magnetic or magneto-optical media, it seems possible to increase the availability of audiometric tests using a computer-based hearing screening system. The computer-based hearing screening system consists of a personal computer, a disk drive, coupled through its acoustic output with the acoustic amplifier, with the gain calibrated through the control and with an optional voltmeter. Headphones or a bone vibrator are plugged in to the amplifier output. The three modes of operation are envisioned in the system. First a questionnaire form is filled in by the system operator. The next step is audiometric tests and the last phase is speech-in-noise tests.

**Conclusion:** Audiometry will be an ongoing task in almost all occupational health departments. There is a need for sophisticated audiometers to measure accurately the hearing losses within the workforce. There is also a need to analyze the information produced and to store that information for future use. So far it has been possible to measure the hearing losses reasonably well and to analyze that information in part within the audiometer. It has not been possible to store this information except as a paper copy. With the help of computer-based hearing screening system, people who are suffering or may suffer from hearing problem may be detected in a very cost effective process. Based on the result of the test, those who may have the hearing problems may refered to the hospital, so that they can be cure easily.

**P-79**

**Dosimetric evaluation of half beam blocked fields**

Lalit M Aggarwal, A Mandal, S Pradhan, UP Shahi, AK Asthana. *Department of Radiotherapy and Radiation Medicine, Institute of Medical Sciences, Banaras Hindu University, Varanasi (UP) - 221 005, India*

**Introduction:** Half-beam block technique is a widely used technique in which the collimator is opened two times the treatment field and half of the beam is then shielded. As asymmetric collimator facility is not available in majority of the Telecobalt units, Breast cone which is basically a half beam shielding block is used for the treatment of intact breast or chest wall following surgery. Half beam blocked field results in cutting off divergence of the beam leading to minimization of the radiation dose to the underlying lung tissue. It is also useful during abutting fields to avoid overdose at junction due to the diverging nature of radiation beams. It could be expected that dosimetric parameters of the beam would change while one is blocking half of the useful beam. The present study has been aimed at investigating the effect of beam blocking on output, Percentage Depth dose (PDD), depth dose profile, etc.

**Materials and Methods:** Output measurements were carried out on Theratron 780-E using 0.6 cc ionization chamber in water at 5 cm depth. These measurements were carried out for varying field sizes i.e., 20x20 cm, 15x15 cm, 10x10 cm with and without breast cone. To simulate the useful beam, measurements were also carried out for the fields 20x10 cm, 15x7.5 cm, 10x5 cm without any shielding. A 0.125 cc ionization chamber (PTW) was used to obtain PDD data in water phantom (MT-150-T, Med Tec) having vertical drive mechanical resolution of 0.1 mm. Kodak X-Omat V film (Kodak, Rochester, NY) was also exposed in a solid acrylic phantom to see the depth dose beam profiles. Exposed films were scanned and the beam profiles were obtained using software developed by TSG Integrations Pvt. Ltd., India.

**Results:** Evaluation of the resulting data showed that output of useful beam increased by 2% while using breast cone (half beam block) as compared to the open field having same area by perimeter (beam size equivalent to useful beam with breast cone). The change in percentage depth dose was less as compared to open field. Depth dose profile at 5 cm depth showed perfect elimination of divergence of the beam under blocked area. Measurement of penumbra showed that in comparison to the open field, it reduced to one third with half beam block. Therefore, it could be concluded that with breast cone, measurement of percentage depth dose, output and other dosimetric parameters needs to be carried out separately.

**Acknowledgement:** Authors are thankful to Er SL Kapoor, Director, TSG Integrations for providing the software in carrying out this study.

**P-80**

**Practical consideration of task group 51 protocol**

K Senthil Nathan, S Habeeb Rahman*, M Florence. *Dept. of Oncology, Kamineni Hospitals, Hyderabad - 500 068, *Radiation Dosimetry, Hyderabad - 500 033, India*

**Introduction:** The AAPM's Task Group 51 (TG 51) is a protocol for clinical reference dosimetry of mega voltage radiation therapy. Explicit applications of TG- 51 to high energy accelerator with electron capability require to have a 1 mm thick sheet of lead for photon energy above 10 MV to avoid the electron contamination and for a calibration of electron beam having energy less than or equal to 6MeV parallel plate chamber are recommended. The purpose of the present work is to examine if these requirements are absolutely necessary. We also examined the differences in absolute dose determined using an ADCL calibration combined with the published $K_{ecal}$ factor (TG-51 Method-B) with that determined by acquiring $K_{ecal}$, $N_{D,\,w}$ from Cross Calibration of Parallel Plate chamber against cylindrical chamber in a high energy electron beam (TG-51 Method A) and using this value for other energies.

**Materials and Methods:** Measurements were made for 15 MV photon energy on Siemens linac using 0.6 cc ion chamber. The PDD were measured with and without 1mm thickness of lead foil. The dose rates at $d_{ref}$ were determined with cylindrical and parallel plate chambers. Method A and Method B for electron calibration was also compared.

**Results and Discussion:** Lead vs no lead: For photon energy 15 MV to determine $K_{Q,}$ the PDD (10x) (depth dose with electron contamination

$K_q$ value for comparing lead and alebric expression

| Machine Siemens | Energy (MV) | % dd (10) x (Pb/No Pb) | $K_Q$ (Pb/alg.exp) |
|---|---|---|---|
| | 15 | 1.008 | 0.999 |

| Energy (MeV) | Cylindrical chamber | Parallel plate chamber | Parallel plate/ cylindrical plate |
|---|---|---|---|
| 6 | 95.56 | 106.08 | 1.110 |
| 9 | 99.77 | 103.96 | 1.041 |
| 12 | 100.28 | 102.38 | 1.020 |
| 15 | 99.05 | 100.78 | 1.017 |
| 18 | 97.70 | 99.55 | 1.018 |

| Energy (MeV) | Method A/Method B |
|---|---|
| 6 | 1.108 |
| 9 | 1.037 |
| 12 | 1.016 |
| 18 | 1.012 |

This PDF is available for free download from a site hosted by Medknow Publications (www.medknow.com)

[Downloaded free from http://www.jmp.org.in on Friday, July 29, 2011, IP: 130.14.254.25] || Click here to download free Android application for t

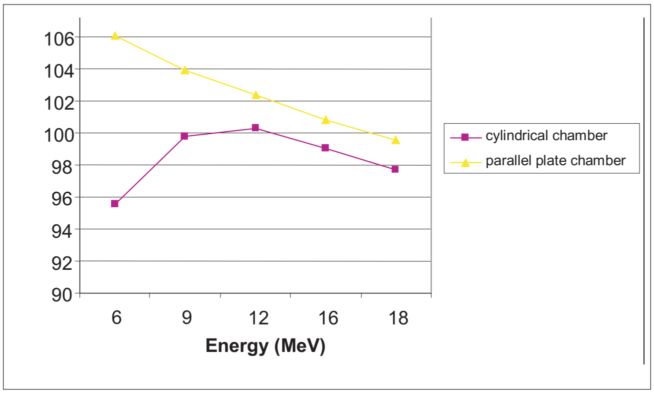

**Figure 1:** Comparison of parallel plate and cylindrical chambers removed) is measured. $K_Q$.

**Parallel plate vs Cylindrical chamber:** Dose is determined for parallel plate and cylindrical chambers for energy ranges from 6-18 MeV and the values are tabulated below.

**Electron beam calibration (Method-A, Method-B):** Finding the product of $k_{ecal}$ and $N_{D,W}$ $co^{60}$ by cross calibrating the plane parallel chamber against cylindrical chamber (Method-A). Product of the supplied $K_{ecal}$ value with an ADCL supplied $N_{D,W}$ (Method - B) and the values are tabulated below.

**Conclusion:** We placed 1 mm lead foil in the treatment head to remove the electron contamination, but the $K_Q$ value varied only by 0.2%, it is insignificant. The comparison between parallel plate and cylindrical chamber showed no measurable difference in the calibration factor across the range of electron energies. For cross comparison of parallel plate chamber against cylindrical chamber method A is preferable to Method B.

### P-81

### Comprehensive dosimetry: A clinical physicist perspective

VP Choube, S Choudhuri*, H Ghare, B Sarkar, M Laviraj, A Deshmukh. *Dept. of Medical Physics, *Radiation Oncology, R.S.T. Regional Cancer Hospital, Research and Teaching Institute, Nagpur, India*

**Introduction:** Aim of the study is to introduce a terminology for Clinical Physicist practice and advice in relation to dosimetry aspects of radiation physics in Radiotherapy clinics by analyzing and assessing the actual process of dosimetry to achieve the goal of Radiotherapy (RT). The conceptual procedure and practice specific methods are used. Dose measurement, determination, distribution, analysis is recognized as dosimetry. Dose is the main interest of quantity in the management of malignant and benign tumors by radiation therapy. 'Dose Standard': (Primary, Secondary and Field-tertiary) is a measure, norm or model in comparative evaluations of absolute absorbed dose in water. In clinics, one should be able to measure or convert dose at any depth, source to skin distance, source to prescription point distance, field size or at any angle for specific type and energy of radiation. The Dosimetry process performed at PSDL, SSDL and field (Clinics) are recognized as PRIMARY, SECONDARY and FIELD DOSIMETRY respectively and are shown in formulas as follows:

$(\text{Actual dose})_{\text{at PSDL}}$ = Direct measurement as per principle formula ................. (1).

$(\text{Actual dose})_{\text{at SSDL}} = (\text{Observed value})_{\text{at SSDL}} \times [\,(N_{DW})_{PSDL}]_{\text{for SSDL}}$ ...................(2).

The field user receives C.F. (Calibration Factor) from SSDL $CF_{\text{for user}} = [(N_{DW})_{SSDL}]_{\text{to User}}$ = Actual Dose / $[(\text{Observed value})_{\text{for user}}]_{\text{at SSDL}}$

At clinic site i.e., at field user,

$(\text{Actual Dose})_{\text{at User}} = (\text{Observed value})_{\text{at User}} \times [\,(N_{DW})_{SSDL}]_{\text{to User}}$ ........ (3).

Field user can also cross calibrate other chambers, dosimeters available in hospital and generate cross calibration factor (CCF) as

$CCF_{\text{for user}} = [(N_{DW})_{\text{User calibrated}}]_{\text{to User other}}$

= Actual Dose / $[(\text{Observed value})_{\text{for other}}]_{\text{at User Calibrated ch.}}$

The calibration of field chamber and dosimeter has been mandatory to make dose traceable to PSDL and recommended to repeat after every 3 years or any repairs. Dosimetry, Treatment Planning, Radiobiological effect predictions, assessment and/or design isoeffective RT protocols are the prime responsibilities of Clinical Physicist. The Standard Protocols for Absolute Dose Rate determination recommended by IAEA (TRS 398), AAPM (TG-51), RPAD-BARC designed commissioning protocols, BJR-25 (CADD data) with relative dose data measurement for checking consistency of all clinically relevant physical parameters (such as field size, PDD, TPR, penumbra flatness, symmetry, etc.) are routinely used for Dosimetry. This should be globally nomenclated as PHYSICAL DOSIMETRY of Radiotherapy and should be routinely audited under TLD postal dose inter-comparison procedure for every individual technology. The Dose Volume relation based norms of ICRU 38, 50 and 62 are used in treatment planning. The volumetric anatomical and field technique and/or source loading based beam arrangements are clubbed to achieve desired dose distribution(s) backed up by differential and cumulative DVH assessment for each phase and/or fraction and thereafter combined effect of overall schedule based dosimetry practice should be nomenclated as CLINICAL DOSIMETRY. The Dose-volume relationships along with TIME FACTOR" (treatment time, OTT, inter-intra fraction gap) and "RADIOSENSITIVITY" of tumor and normal tissue are the parameters in use for response predictions or assessment and such dosimetry practice should be considered as RADIOBIOLOGICAL DOSIMETRY. Dosimetry practice in radiotherapy for top three ranking cancers in our hospital will be discussed. With our experience, we are hereby suggesting 1) "COMPREHENSIVE DOSIMETRY" as a terminology for specifying the Physical, Clinical and Radiobiological Dosimetry component of Clinical Physicist responsibilities and 2) segregation of Physical, Clinical and Radiobiological Dosimetry as sub-branches of Clinical Physics applications with dose quality audits even for Clinical and Radiobiological Dosimetry at national level so as to effectively apply the Clinical Physics policies, ease the national and international inter-disciplinary transaction and bridge the gap of radiotherapy delivery quality between the RT centers.

### P-83

### Monte Carlo model for estimating fetal doses to average Indian female for abdominal examination

K Biju, T Palani Selvam, S Kannan. *Radiological Physics and Advisory Division, Bhabha Atomic Research Centre, Anushaktinagar, Mumbai - 400 094, India. E-mail: biju_keshav@indiatimes.com*

**Introduction:** Occasionally, it is clinically necessary to perform a radiological examination(s) on a woman who is known to be pregnant or an examination is performed on a woman who subsequently discovers that she was pregnant at that time. In radiological examinations, especially of the lower abdomen and pelvis area, the fetus is directly irradiated. Fetus is assumed to have the same risk for potential radio carcinogenic effects as in children. It is therefore important to be able to determine the absorbed dose to the fetus in diagnostic radiology for pregnant patients more accurate. This work presents Monte Carlo modeling of various stages of the pregnancy and the fetal dose normalized to entrance skin dose to an average Indian female patient for a typical abdominal AP examination.

**Materials and Methods:** The geometry and X-ray beam characteristics of the abdominal X-ray examination was simulated using Monte Carlo code, MCNP. The mathematical model for the average Indian adult phantom was modified to incorporate the different stages of prenatal life, end of first, second and third trimesters of pregnancy (at 90 days, 180 days and 266 days after conception) as determined by ICRP. Uterus is modeled with regions specifying uterine wall, contents and the fetus separately. At different stages of pregnancy, the structure has different size and locates differently. Uterus is a pear shaped structure modeled as the frustum of a right circular cone capped at both ends by hemispheres. Fetus is the inner region in the uterus and

This PDF is available for free download from a site hosted by Medknow Publications (www.medknow.com).

[Downloaded free from http://www.jmp.org.in on Friday, July 29, 2011, IP: 130.14.254.25] || Click here to download free Android application for t

Table 1: Fetal doses normalized to entrance skin dose in mSv/mGy for abdominal X-ray examination

| Name of the trimester | Fetal dose (mSv/mGy) |
|---|---|
| First | 0.225 |
| Second | 0.115 |
| Third | 0.0529 |

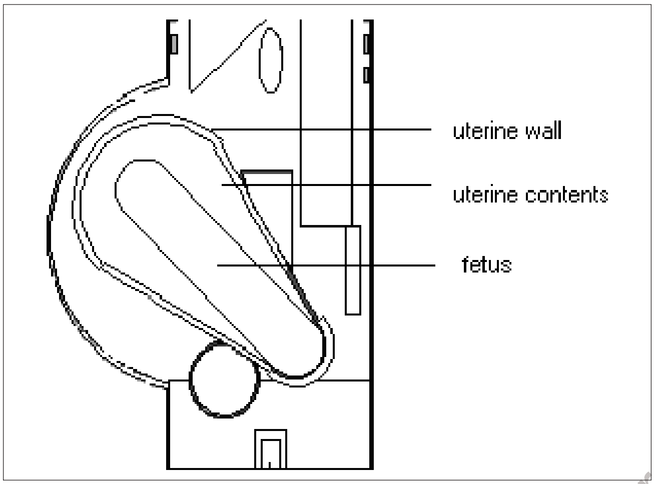

**Figure 1:** The cross sectional view of phantom model of the second trimester is modeled as a right circular cylinder capped with hemispheres. The growth of the uterus and the fetus were incorporated by changing the model parameters. The fetal doses are the as the average dose to the fetal region. At first trimester, the uterus has not been sufficiently enlarged to cause any changes in the exterior of the adult phantom. In the second and 3rd stage of pregnancy, uterus cannot be contained within the confines of the adult phantom. The trunk was therefore enlarged to accommodate the enlarged uterus and other abdominal organs are modified accordingly. The trunk was represented by the elliptical cylinder of the non-pregnant phantom plus the part of an ellipsoid that extends outside the cylinder to the front of the phantom. The volumes of the uterine regions were scaled down to suit the average dimensions of the Indian adult from the standard dimensions quoted in the literature. The cross sectional view of the phantom of the 2nd trimester is shown in figure 1. The X-ray source was made to fall on the phantom for a typical field size of 26x36 cm, generally used for abdominal AP examination. 5 Million histories were run to estimate the dose to the fetus and the entrance skin dose (ESE) for the X-ray beam 80 kV, 2.5 mm Al filtration. Air kerma rate was estimated in the fetus and at the surface of the phantom (entrance skin dose).

**Results and Conclusion:** The normalized fetal doses for abdominal AP examination or the three trimesters are presented in the Table 1. The standard deviation of the values is less than 2%. The normalized dose is less for the last stages of pregnancy in this particular exposure situation. This is due to higher shielding offered by the abdomen, uterine contents etc. and the extended size of the fetus.

**P-84**

**PC based TL reader for medical and research applications**

P Ratna, Probal Chaudhury, G Nilshree, DN Sharma. *Radiation Safety Systems Division, Bhabha Atomic Research Center, Mumbai - 400 085, India*

**Introduction:** Thermo luminescence dosimeters (TLD) are widely used for dose estimation in medical field. TL technique is also used in many other fields like archaeology, geology and forensic science and quality control industry. For such different applications, selection of proper TLDs is very essential for the accuracy of the dose estimation. Basic requirement, for selecting a dosimeter and studying their characteristics is a proper TL Reader System. A new PC based TLD Reader has been developed. In this new PC Based TL Reader, where the parameters like heating rate, end point temperature, cycle time, EHT and start temperature can be entered through the PC and can be permanently stored in the memory of the Reader. Software has been developed for windows 2000/ XP platform using Microsoft Visual Basic for the Reader control, data acquisition, data processing, on-line plotting of glow curves and heater profiles and data analysis. For exclusive study of TL characteristics of TL materials, heating rate should be reproducible. In this paper study of the comparison of the software generated heating rate with the actual heating rate has been done. Study has also been done on the variations in the pre-set heating rates with respect to the clamping temperature from 100°C to 450°C.

**Materials and Methods:** A linear temperature heating system has been developed using microcontroller at89s8252. System hardware consists of AK-type thermocouple, photo multiplier tube, current to frequency converter, microcontroller card, linear temperature controller card, EHT module and a low voltage, high current transformer. in this new pc based TL reader, clamping temperature can be varied between 100°c to 450°c and heating rate from 0.5°c /sec to 60°c /sec and cycle time from 1 sec to 999 seconds. This reader is tested for different clamping temperature up to 450°c for a pre-set heating rate of 10°c /sec. a graph [Figure 2] between time versus temperature shows that the heating rate is constant up to the maximum clamping temperature. (Within the uncertainty of ± 0.4%, see Table 1) there is a difference between the pre-set and measured heating rate, which ranges from 4.2-5.5%. Moreover this is not a limitation for its use, as the actual heating rates are reproducible with s = 0.4%. Table 1 shows the heating rates calculated for different clamping temperatures for a set heating rate of 10°c /sec. Study for the various heating rates from 0.5°C/sec to 60°C /sec has been done with this new Reader. Table 2 shows, calculated heating rates for different set values of heating rates. To study the performance of the Reader, glow curves for the TLD discs exposed to 500 mSv of $\gamma$, ($CaSO_4$:Dy) have been take, for different heating rates and observed that glow curve shifts left (in time scale) as the heating rate increases [Figure 1].

**Software:** The software of the reader consists of two parts. 1) Assembly language software written for AT89S8252 microcontroller for controlling the entire operation of the TLD reader. 2) An interactive user-friendly software package by using microsoft visual basic has

Table 1: Actual temp and heating rates for different clamping temp and heating rate = 10°C/sec

| Temp set°C | Actual temp°C | Actual heating rate°C/sec |
|---|---|---|
| 100 | 99 | 9.45 |
| 150 | 153 | 9.58 |
| 200 | 199 | 9.53 |
| 250 | 249 | 9.5 |
| 300 | 299 | 9.5 |
| 350 | 349 | 9.49 |
| 400 | 400 | 9.5 |
| 450 | 452 | 9.5 |

s = 0.4%

Table 2: Actual heating rates for different set heating rates and clamping temp of 290°C

| Heating rate set°C/sec | Actual heating rate°C/sec |
|---|---|
| 1 | 0.95 |
| 5 | 4.75 |
| 10 | 9.55 |
| 20 | 19.07 |
| 30 | 28.72 |
| 40 | 38.44 |
| 50 | 48.82 |
| 60 | 58.85 |

This PDF is available for free download from a site hosted by Medknow Publications (www.medknow.com)

[Downloaded free from http://www.jmp.org.in on Friday, July 29, 2011, IP: 130.14.254.25] || Click here to download free Android application for t

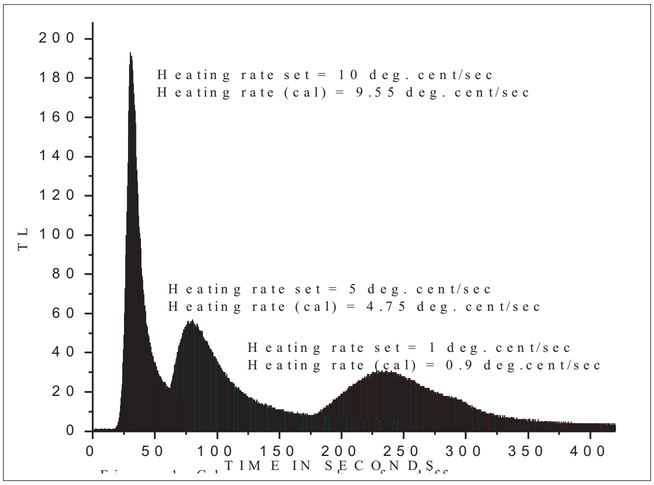

**Figure 1:** Glow curve taken for different heating rates for CaSO4: Dy discs for a clamping temperature of 290 deg. centigrade

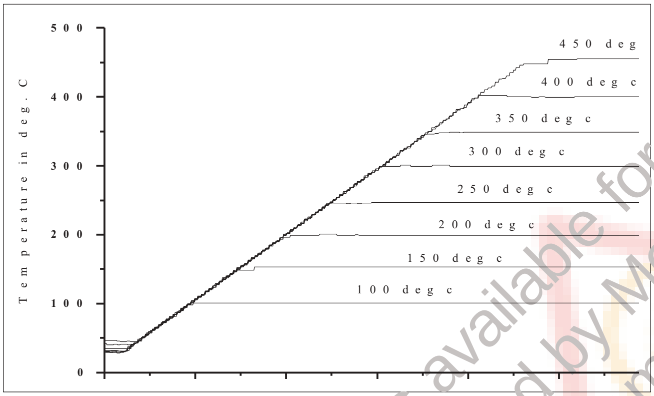

**Figure 2:** Temperature profile for different clamping temperaturesang heating rate set = 10 degc/sec been developed. on initiation of data acquisition, real time data is received from the reader at an interval of 100 ms for preset duration. data includes time, temperature and tl values. Real time plots representing temperature profile and glow curve are generated for visual presentation and the data is stored in a file for offline processing.
**Results:** A new PC based TL reader is configured to operate over the temperature range 100°C to 450°C and a linear heating rate which can be varied from 0.5°C /sec to 60°C /sec. Performance of the Reader has been evaluated using Gamma irradiated $CaSO_4$: Dy Teflon discs.
**Conclusion:** The PC based TL Reader described above has improved performance for TL measurements, as it provides permanent storage of the glow curves along with all the parameters. And the glow curve data in a user defined file for the further analysis. These features are very much useful for dose analysis in clinical dosimetry and basic research of TL phosphors.

### P-85

### Effect of radiation on salivary PH in head and neck cancer patients during and after radiotherapy treatment

Rimpu Kumari, *Nidhi Patni, PK Goyal. *Department of Zoology, University of Rajasthan, Jaipur - 302 004, *Department of Radiotherapy, Bhagwan Mahaveer Cancer Hospital and Research Center, Jaipur, India.*
E-mail: pkgoyal200@rediffmail.com

**Introduction:** Tumors of the head and neck comprise an important group of neoplasia, the incidence of which is increasing in many parts of the world. These constitute a major health problem in developing countries like India, representing a leading cause of death. Since, the oral cavity is more accessible to complete examination, it could be used in early detection of precancerous and cancerous lesions. But either due to ignorance or inaccessibility of medical care, the disease gets detected in the later stages. Thus, there is a need for improvement in early detection of oral carcinomas, because in the initial stages, treatment is more effective and the morbidity is minimal. Normal salivary function is considered to be critical for the maintenance for healthy oral mucosa. Salivary pH reflects cellular pH and the state of health. Cancer needs an acid and low oxygen environment to survive and flourish within. Terminal cancer victims are around 1000 times more acidic than normal healthy people. Oral fluids provide an easily available non-invasive for the diagnosis of a wide range of diseases and clinical situations. So test of salivary acidity or alkalinity can be used to measure the susceptibility to cancer. The present study is an attempt to evaluate the variations in pH of saliva in oral cancer patients during and after radiotherapy treatment.
**Materials and Methods:** The study was conducted on 50 patients with histopathologically proven epidermoid carcinoma of head and neck (stage I, II, III and IV) attending the Department of Radiotherapy, Bhagwan Mahaveer Cancer Hospital and Research Center, Jaipur. The selected group of patients was subjected to history taking and routine laboratory necessary examination. Patients were classified according to the TNM staging system of the American Joint Committee for cancer staging and end results reporting. Patients were treated by external telecobalt beam radiotherapy and were advised to give the saliva after rinsing their mouth and to hold the water in mouth for at least two minutes, moving it with tongue. The sample was collected in a beaker and pH was analyzed with the help of pH INDIKROM papers (Qualigens Fine Chemicals, a division of GlaxoSmithKline Pharmaceuticals Limited, Dr. Annie Besant Road, Mumbai - 400 025). The pH values were estimated before therapy, midtherapy (25-30 Gy), after completion of radiotherapy (50-70 Gy) and four times during follow-up after every three months.
**Results and Conclusion:** In the present study, the pH level before therapy in head and neck cancers were found to be lower (acidic) initially but later it increased (alkaline) during radiotherapy treatment and remained alkaline in maximum subjects after completion of therapy as well as during follow ups, while in other patients values become normal (i.e., neutral). Radiotherapy damages the mucosal epithelium and causes subsequent changes in oral fluids by reducing the tumor burden. Due to regression in tumor growth, the environment surrounding around tumor cells become alkaline, as a result pH of saliva gets affected and shows alkalinity. It has been found that cancer cells go into a dormant state if their pH is around 7.4. (7.0 is neutral pH and 7.4 is slightly alkaline). Acidity prevents the production of A, C, G and T nucleotides, the steps on the spiral-shaped DNA ladder, that allow for normal DNA synthesis. Thus, it can be concluded that the state of salivary pH changes favorably with the advancement of radiotherapy treatment, which may controls tumor growth and induce regression in patients with residual tumor. Also, change in salivary pH after completion of radiotherapy treatment may be an indicator of such tumors.

### P-86

### Dosimetry quality audit programme for high energy X-rays from medical linear accelerator

SD Sharma, S Vandana, A Philomina, SS Deshpande*, S Chaudhary*, S Kannan, BC Bhatt**. *Radiological Physics and Advisory Division, Bhabha Atomic Research Centre, CT and CRS Building, Anushaktinagar, Mumbai - 400 094, *Department of Radiotherapy, P. D. Hinduja National Hospital, Mahim, Mumbai, **CSIR/BARC, Trombay, Mumbai - 400 085, India*

**Introduction:** A quality audit (QAu) is an independent examination and evaluation of the quality assurance activities and results of an institution. As per IAEA recommendations (IAEA TECDOC 1040, 1997), QAu should be conducted by personnel within the institution as well as those from outside the institution. However, a complete QAu by personnel from outside the institution is expensive and time consuming. QAu by mailed dosimeter is therefore an alternative to fulfil partially

This PDF is available for free download from a site hosted by Medknow Publications (www.medknow.com)

[Downloaded free from http://www.jmp.org.in on Friday, July 29, 2011, IP: 130.14.254.25] || Click here to download free Android application for t

Table 1: Measured dosimetry parameters for 10x10 cm² field

| Parameter | Measured data for 10x10 cm² field | | | | | |
|---|---|---|---|---|---|---|
| | 6 MV | | | 15 MV | | |
| | IC | TLD | RCF | IC | TLD | RCF |
| Output at $d_m$ (cGy/MU) | 1.0561 | 1.0531 | 1.0512 | 1.0245 | 1.0246 | 1.0315 |
| PDD at 3 cm | 95.16 | 93.42 | 93.56 | 100.00 | 100.00 | 100.00 |
| PDD at 5 cm | 86.37 | 85.87 | 87.28 | 94.13 | 95.70 | 94.35 |
| PDD at 10 cm | 67.19 | 66.42 | 69.50 | 76.54 | 75.01 | 75.78 |
| PDD at 15 cm | 51.34 | 50.01 | 50.72 | 61.63 | 61.35 | 61.37 |
| PDD at 20 cm | 39.07 | 38.28 | 38.61 | 49.60 | 48.17 | 48.35 |
| Quality Index | 0.676 | 0.665 | 0.650 | 0.756 | 0.755 | 0.755 |

the requirements stipulated under audit programme by national/ international agencies. IAEA through the networks of Secondary Standard Dosimetry Laboratories (SSDL) is conducting the postal audit from last four decades. However, IAEA based audit conducted by SSDL is limited to audit of dose output in reference conditions only. A QAu method for audit of important dosimetry parameters, both in reference and non-reference conditions, of high energy X-rays from medical linear accelerator (linac) was developed and tested. This paper describes briefly the method developed and its validation results.

**Materials and Methods:** Two different specialised PMMA jigs, one for holding thermoluminescent (TL) capsules and other for radiochromic film (Gafchromic EBT) samples, were developed and fabricated locally to carry out the dosimetry audit measurements. These two jigs were having provision to hold TL capsules/ radiochromic film (RCF) samples at the depth of dose maximum ($d_m$), 5, 10, 15 and 20 cm along central axis of the beam so that the dosimetry parameters such as absolute output, percentage depth dose (PDD) at different depths and quality index (QI) of the beam can be calculated from readouts of different TL capsules/ RCF samples irradiated simultaneously. The RCF jig was also having provision to hold a long RCF strip to evaluate the beam uniformity and symmetry at 10 cm depth. TL powder and the polyethylene capsules used in this work were those which are used by IAEA for dose intercomparison in beam therapy. Standard procedures of TL and RCF dosimetry were followed. The calibration for TL and RCF dosimeters were carried out in $^{60}$Co gamma rays and 6 and 15 MV X-rays at 5 cm depth in a water phantom. Freshly prepared TL capsules/ RCF samples were placed in their respective PMMA jigs and were irradiated for field sizes 5x5, 10x10 and 20x20 cm² in 6 and 15 MV X-rays from the medical linac (Clinac 2300 CD, Varian Medical Systems). The absolute output of the accelerator for 6 and 15 MV X-rays were measured using calibrated thimble ionization chamber (IC) following the methodology of IAEA TRS 398. The PDD, QI, output factor and beam profile at 10 cm depth of the beam were measured using a 3-D radiation field analyzer (Blue Phantom, Scanditronix) and miniature diode detectors. The absolute output, PDD, QI and output factors thus measured by these three different methods were compared. Further the beam uniformity and symmetry at 10 cm depth determined from the RFA measurements and RCF strip were also compared.

**Results and Discussion:** Measured dosimetry data using TL capsules and RCF for audit purposes along with ion chamber (IC) measured data as reference data set are shown in Table 1 for 10x10 cm² filed and in Table 2 for 5x5 and 20x20 cm² fields. It is observed from these two tables that reference values obtained using IC and TL/RCF measured values of dosimetry parameters for 6 and 15 MV beams are in very good agreement to one another. Beam uniformity and beam symmetry measured using RCF and IC were also in good agreement to each other. Though TLD obtained values were highly reproducible, RCF values lack expected reproducibility.

**P-87**

**Performance evaluation of kVp meter/timer developed by RSSD, BARC**

MD Lalsare, UP Gaonkar, AR Kulkarni*. *Radiation and *Radiological Safety Systems Division, Bhabha Atomic Research Centre, Mumbai-85, India.* E-mail: artik@aerb.gov.in

**Introduction:** Measurement of X-ray beam quality is an important part of Quality Assurance of Diagnostic X-ray machines. The accepted accuracy of applied kVp is within + 5 kV and for timer it is + 10%. This instrument measures the peak kilo voltage applied to X-ray tube and X-ray exposure time. It uses non-invasive method of kVp measurement. Operating potential is one of the important factor which affects the image quality. Development of kVp meter/timer was presented in National Symposium on Nuclear Instrumentation in 2004.[1] The instrument is further upgraded to two separate units as the detector unit and readout unit. Readout unit can be placed at control panel (10 m of cable is provided between detector unit and readout unit).

**Materials and Methods:** The kVp Meter/Timer is based on PIN semiconductor diode detectors. The kVp Meter /Timer comprises of two parts namely, the detector unit and the readout unit both of which are based on Atmel micro controller 89S8252. The detector unit is interfaced to the readout unit with a RS-232 connection. This instrument covers the range from 50 kVp to 125kVp with an accuracy of + 3 kVp and exposure time ranges from 1 ms to 9999 ms with an accuracy of + 5 ms. The detector unit consists of diodes, which are placed under copper filters of 0.5 mm, 1.0 mm and 2.0 mm thickness to cover a wide range of kVp measurement from 50 kVp to 125 kVp. When detector unit is exposed, response of detector under lowest thickness (0.5 mm)

Table 1: Comparison study of kVp meter

| Applied | 1 $\phi$ Full wave machine | | | 3 $\phi$ Full wave machine | | | High freqency constant potential machine | | | |
|---|---|---|---|---|---|---|---|---|---|---|
| kV | mAs | Unfors | BARC | mAs | Unfors | BARC | mAs | Unfors | BARC | Multi-o-Meter |
| 50 | 60 | 56.9 | 54 | 24 | 73.6 | 71 | - | - | 50 | - |
| 60 | 20 | 62.7 | 59 | 10 | 75 | 72 | 20 | 59.7 | 60 | 59.5 |
| 70 | 20 | 70 | 69 | 10 | 83 | 81 | 20 | 69 | 71 | 67.6 |
| 81 | 20 | 74.8 | 76 | 6 | 87 | 89 | 10 | 78 | 79 | 77.2 |
| 90 | 20 | 82.4 | 81 | 5 | 91.6 | 89 | 5 | 86.6 | 86 | 85.7 |
| 100 | 20 | 91.2 | 92 | 5 | 100.2 | 101 | 5 | 97.5 | 98 | 96.7 |
| 109 | - | - | - | - | - | - | 5 | 106.6 | 107 | 106 |
| 121 | - | - | - | - | - | - | 2.5 | 119 | 118 | 118 |
| 125 | - | - | - | - | - | - | 1 | 121 | 119 | 121 |

This PDF is available for free download from a site hosted by Medknow Publications (www.medknow.com)

[Downloaded free from http://www.jmp.org.in on Friday, July 29, 2011, IP: 130.14.254.25] || Click here to download free Android application for t of copper filter interrupts the micro-controller and starts the measurement cycle. At the end of the exposure, the software timer is stopped and measurement cycle terminates. The ratio of response of the two appropriate detectors is computed and the kVp is derived from the stored look-up table and displayed.

**Discussion:** Performance of kVp Meter/Timer developed by RSSD, BARC was compared with kVp meter and Multi-O-Meter manufactured by M/s. Unfors, Sweden. It is observed that for single phase and three phase machines, set kVp in the control panel was not matching with the observed kVp in the kVp meters, however the kVp meter inter comparison is found within the limit. This shows the machines were not calibrated. For high frequency constant potential the set kVp and observed kVp in all kVp meters were well matching. For single applied kV measurements were repeated with various source to detector distance. Results were consistent and within acceptable limit. The X-ray machines from manufacturers like Siemens Ltd., Wipro GE were used for measurements with these kVp meters. The performance of kVp meter is found within + 3kVp in comparison with Unfors kVp meters and exposure time was found within + 3% of the set time in the X-ray machine. The detector unit works on re-chargeable Ni-Cd battery of 6V/1300 mAh capacity, which has longer life and the readout unit is provided with 4 x 1.5 V pencil cells. The same instrument can be used to measure the kVp of dental X-ray machine.

**Acknowledgement:** The authors are thankful to Shri Kannan, Head RPandAD for his encouragement and valuable guidance and Shri V. K. Shirva, Shri M. L. Bhutani and Shri N. Dixit for their support.

### Reference

1. Lalsare MD, Gaonkar UP, Kannan S. A new kVp Meter/Timer based on a micro-controller. Proceedings, DAE-BRNS. Nat Symp Nucl Instr 2004:204-5.

### P-88

### Intensity map extention and dose conformity to target volume in an IMRT procedure

Pramod Kumar Dixit, KR Muralidhar, Rajeensh Kumar, Madhusudhan Sresty, S Shiyama, Thirumalai Swamy, AK Raju. *Indo American Cancer Institute and Research Center, Banjara Hills, Road No 14, Hyderabad - 300 034, India, E-mail: dixit.pk @gmail.com / pkd-dixit@yahoo.co.in*

**Introduction:** The purpose of this paper is about the need of intensity map extension and its effect on segment number and MU in an IMRT plan. IMRT is a well known procedure in Radiation Therapy to achieve best conformity to tumor. The non uniform fluence of photons delivers non uniform dose to the irradiated region with so many pixels of varying intensities. This pixels of varying intensities is known as intensity map. The non uniform flunce map is generated by the motion of MLC leafs. Due to the the inverse planning dose prescription the intensity map on the PTV and OARs are different for which dose to OARs and PTV are different. Within the field if there is any organ motion or swelling occurs to PTV, then some part of the PTV may miss the estimated dose coverage. So the ideal intensity map can be extended to account for the region where organ motion or swelling is expected to occur prior to generating MLC segments. The intensity map extension is controlled by following parameters. (i) Distance to extend the intensity map away from the skin i.e., out side body. (ii) Distance to average the intensity along a given row in the intensity map starting from the body out line.

**Materials and Methods:** Patient CT slices are obtained for IMRT planning. Then body, all OARs and PTV (Breast) are contoured. The breast IMRT plan was done on CMS XIO planning system using convolution algorithm. PTV to MLC margin was 1 cm. With different intensity level the plan was done. The discrete intensity level varied from 5-10 and plan was obtained with the change in MU and total segments with same optimizing parameters.

**Result:** Table 1.

**Conclusion:** With increase in discrete intensity level no. of segment size increases. It is because for higher discrete intensity level, system has to form intensity map beyond PTV volume. For which MU variation for each beam and MU per segment also varies.

| *Discrete intensity level* | *Number of segments* | *Monitor units for different beams* | | | | |
|---|---|---|---|---|---|---|
| | | *RAO300 deg* | *RAO1320 deg* | *RAO2340 deg* | *LPO130 deg* | *LPO1110 deg* |
| 6 | 67 | 192 | 194 | 157 | 251 | 175 |
| 7 | 83 | 248 | 211 | 171 | 262 | 214 |
| 8 | 87 | 200 | 196 | 171 | 257 | 191 |
| 9 | 102 | 213 | 207 | 150 | 291 | 205 |
| 10 | 115 | 237 | 193 | 147 | 275 | 197 |

### P-89

### Study of in house immobilization Device's accuracy for the treatment on pelvic and abdomen regions using 2D-portal and volumetric analysis (IGRT- mega voltage cone beam)

NK BABU, VK Sathiyanarayan, Shrikant Deshpande, Kumaraswamy, Sumit Basu, Vikram Maiya, Surender Pawar, Preethamol Joseph. *Department of Radiation Oncology, Ruby Hall Clinic, Pune, India*

**Introduction:** The aim of radiotherapy is to deliver maximum dose to tumour and to spare normal organs as much as possible. In 3D-conformal radiotherapy (3DCRT) and Intensity Modulated radiotherapy (IMRT) the margin to the target is very tight. Small variation in the daily set up error may lead to under dose to the tumour and over dose to the critical organs. Various immobilization devices help us to achieve the consistency of the patient positioning.

**Materials and Methods:** The immobilization accuracy is one of the important factors on radiotherapy practices. Particularly for the treatment of 3D-conformal and Intensity Modulated Radiotherapy treatment (IMRT), the reproducibility is very important as the margin to PTV is less and the critical organs are close to the PTV. It is really challenging to radiotherapy technologist for minimizing daily set up errors particularly for the abdomen and pelvic regions. Immobilization devices from various vendors are costly as that all centers cannot afford. We have translated some of our ideas concerning immobilization in to prototype ones so that after testing and using them they can be prepared on commercial scale. Carbon fiber base plates and thermoplastic materials are found to be useful in reproducing and positioning the patient. They are found to be adequate in the head and neck regions perfectly. In the abdomen and pelvis they do not contain the longitudinal errors positional errors. Hence, we designed a few Vacuum cushion holders from the available material in the department for the treatment of abdomen and pelvic regions for IMRT and 3D CRT. Low-density thermocol material was used along with Vacuum cushions to serve this purpose. For daily setup, first the cranio-caudal matching of the immobilization device with the treatment couch and the Vacuum cushion is positioned on the immobilization device and finally patient is set on the Vacuum cushion with the help of scale on the immobilization devices and the patient's tattoo markers. We found the over all set-up time is less and the accuracy is within acceptable limit.

**Result and Discussion:** 80 Abdomen and Pelvic set up have been studied. Almost 80% of the results, the set up error was found within 2-3 mm and 20% with 3-5 mm, but not exceeding 5 mm.

**Conclusion:** The study shows the immobilization devices not only cost effective also give good reproducibility for the IMRT and 3D CRT treatment.

### P-90

### Estimation of dose to other organs due to screen film mammography: On the basis of Indian data

Kanta Chhokra, V Jayalakshmi*, Reena Sharma*. *RSD, Atomic Energy*

This PDF is available for free download from a site hosted by Medknow Publications (www.medknow.com)

[Downloaded free from http://www.jmp.org.in on Friday, July 29, 2011, IP: 130.14.254.25] || Click here to download free Android application for t

*Regulatory Board, *RP & AD, Bhabha Atomic Research Centre, Mumbai, India*

**Introduction:** This study was aimed at estimation of dose to some of the critical organs such as thyroid, lungs, eyes, abdomen and contra breast during mammography procedures making use of suitable phantoms and thermoluminescent dosimeters.

**Materials and Methods:** The data regarding the distances of critical organs such as thyroid, lungs, eyes, abdomen and contra breast and chest from the breast was collected for a small group of women in the two age groups 25-35 and 40 and above. For this purpose the center between the breasts were taken as the reference point of measurements, except for the of the contra breast, the distance was taken between the nipples. From the studies it was observed that these distances were 10 cm for lung, contra breast 21 cm, thyroid 24.5 cm, eyes 36 cm, abdomen 21 cm and 14 cm chest just below the breast. For this work, the thermoluminescent dosimeters used were LiF: Mg, Cu, P pellets of 4.5 mm diameter, which is tissue equivalent and having an energy independent response within ± 5% for diagnostic X-rays. The detection threshold of LiF:Mg,Cu,P is 50 μSv. The LiF:Mg,Cu.P pellets were cleaned using laboratory grade acetone, annealed at 240° C for 10 min and packed in polythene pouches for the convenience of handling. Four dosimeters were placed in each pack. The packs were fixed at different locations on the phantom and one pack was kept at reference point to estimate the entrance dose. All the packs were exposed simultaneously and the doses recorded by TLDs were evaluated using a calibrated TLD reader.

**Results and Conclusions:** It is estimated that the percentage dose to organs under consideration varied from 4 to 1.4% of the entrance breast dose as indicated in the table below. It is evident that doses received by various organs are dependent on the reference entrance doses. Some of the units are found to give high entrance breast dose of the order of 26 mSv per film. Since X-ray mammography is one of the most reliable imaging modality for the early detection of breast cancer and the radiation induced cancer risk from mammography is estimated to be 1 new case created for every five cases detected, strict quality control programme and regulatory control measures including inspections are essential at regular intervals.

Table 1: Percentage organ doses normalized to breast entrance dose during screen-film mammography

| *Location* | *% organ dose* |
|---|---|
| Entrance dose (breast) | 100 |
| Thyroid | 2.39 |
| Lung | 4.2 |
| Eye | 1.38 |
| Abdomen | 2.89 |
| Contra breast | 1.52 |
| Chest | 2.47 |

### P-91
### Relative dose measurements at different depths in a breast equivalent phantom

Kanta Chhokra, V Jayalakshmi*, Reena Sharma*. *RSD, Atomic Energy Regulatory Board, *RP & AD, Bhabha Atomic Research Centre, Mumbai - 400 094, India*

**Introduction:** This study was aimed at estimation of doses at various depths with in a breast phantom which includes entrance, mid plane and exit dose. The mid plane dose is representative of the mean glandular dose which is the quantity recommended by ICRP-1987 for calculation of risk from X-ray mammography.

**Materials and Methods:** For the study, a dedicated indigenous mammography unit was used along with a locally fabricated PMMA phantom consisting of semi circular discs of varying diameter 9 cm to 21 cm and increasing in thickness by 5 mm at every step.[1] The total 4 cm thicknesses of the phantom with 8 discs in place were used to represent 4.5 cm equivalent breast thickness of composition 50% adipose and 50% glandular tissues.[2] A quality control check performed on the indigenous mammography unit indicated that the system conformed to the acceptable limits. For estimation of doses, LiF:Mg Cu.P pellets of 4.5 mm diameter, which is tissue equivalent and having an energy independent response within ± 5% for diagnostic X-rays was used. The detection threshold of LiF:Mg, Cu, P is 50 μSv. The measurements were carried at different depths of the phantom for three different KVp normally used for clinical purposes i.e., 26 kVp, 28 kVp, 32 kVp. The mammography unit was set in the semi automatic mode where the kV was manually selected and the mAs was automatically selected by the unit. The LiF:Mg,Cu.P pellets were cleaned using laboratory grade acetone, annealed at 240° C for 10 min and packed in polythene pouches for the convenience of handling. Four dosimeters were placed in each pack. The packs were fixed at different locations on the phantom at the surface representing entrance skin dose, at 5 mm depth under skin representing adipose tissues, at the mid plane, representing glandular tissue and at the exit. All the packs were exposed simultaneously and the doses recorded by TLDs were evaluated using a calibrated TLD reader. A calibrated external mammography ionization chamber (Victoreen Model 6000-529) having volume 3.3 cc, diameter 4 cm and thickness 2.5 cm and energy response with in ± 5% for half value thickness in Al of 0.2 to 0.5 mm (16 to 90 kVp) was used with calibrated Victoreen dose calibrator model 8000 Nero ™ mAx for the purpose. The calibration of the measuring system is traceable to National Institute of Standards and Testing (NIST). A comparison and checking of the calibration of the system was done at Radiation Standards and Instrumentation Division, BARC for energy range from 22 kV to 40 kVp and the system performance was found to be with in ± 2%.

**Results and Conclusions:** The method of calculating mean glandular dose by Dance *et al*[3] is not a true representation of the breast dose, as glandular tissues are not concentrated at any one location in the breast. The glandular tissues include the acinar and ductal epithelium and associated stroma.[4] Therefore the dose distribution with in the breast will help in estimating the relative risk in mammography procedures. The results of entrance doses at various depths and at different kVs are indicated in Tables below. It is recommended that this data should be generated at every mammography centre for the

Table 1: Doses at various depths of the breast phantom at 26kVp

| *Location* | *Dose (mSv)* | *% of the entrance dose* |
|---|---|---|
| Entrance (skin) | 3.41 | 100% |
| 5mm depth (under skin) | 2.19 | 69.75 |
| Mid breast | 0.56 | 16.42 |
| Exit | 0.17 | 4.99 |

Table 2: Doses at various depths of the breast phantom at 28kVp

| *Location* | *Dose (mSv)* | *% of the entrance dose* |
|---|---|---|
| Entrance (skin) | 3.16 | 100 |
| 5mm depth (under skin) | 1.81 | 57.28 |
| Mid breast | 0.63 | 11.39 |
| Exit | 0.17 | 5.38 |

Table 3: Doses at various depths of the breast phantom at 32 kVp

| *Location* | *Dose (mSv)* | *% of the entrance dose* |
|---|---|---|
| Entrance (skin) | 4.36 | 100 |
| 5mm depth (under skin) | 2.54 | 58.26 |
| Mid breast | 0.88 | 20.18 |
| Exit | 0.28 | 6.42 |

This PDF is available for free download from a site hosted by Medknow Publications (www.medknow.com)

[Downloaded free from http://www.jmp.org.in on Friday, July 29, 2011, IP: 130.14.254.25] || Click here to download free Android application for t mammography unit being used and the mid- plane dose to glandular element of breast should not exceed 50% of the entrance skin dose for the highest kVp used.

### References

1. Chhokra K, *et al*. An indigenous mammography phantom. J Med Phys 1997;22:3.
2. Thilander A, *et al*. Special problem of patient dosimetry in mammography. Radiat Protect Dosim 1992;43:1.
3. Dance DR, Skinner CL, Carlsson GA. Breast Dosimetry. Appl Radiat Isot 1999;50:185-203.
4. National Commission on Radiological Protection Report 149," A Guide to Mammography and other breast Imaging Procedures", NCRP December 31, 2004.

**P-92**

### Contrast enhancement techniques for mammographic images using computer aided functions

Gurpreet Singh, Pratik Kumar, Sanjay Thulkar*. *Medical Physics Unit, *Dept. of Radio diagnosis, IRCH, All India Institute of Medical Sciences, New Delhi - 110 029, India*

**Introduction:** When simple objects are detected on uniform background, the human observer is limited only by noise in the image and this problem is scarier in low contrast situations. The possibility of improving detection by use of computer techniques in such situations can be much helpful. Other deficiencies in human performance are more obvious targets for improvement through the introduction of computer aided diagnostic techniques. Simple oversight of abnormalities contributes significantly to false negative mammography interpretations. Computer analysis of images can begin only in digital format. This has involved digitization of existing film. Direct digital acquisition of images, now possible in certain clinical situations, will most likely form an eventual input for future practical aided diagnostic systems. Whole breast direct acquisition systems under development include storage phosphor/charge coupled device area detectors as well as scanned beam detectors. But in those cases where simple film screen pattern is used one can scan the film and digitize the image. Using the computer codes contrast can be enhanced and which can help the radiologist by enhancing the micro calcification.

**Materials and Methods:** Computer codes have been developed for enhancing the contrast of images in matrix laboratory language. Various functions which are inbuilt in MATLAB language, particularly suitable for contrast enhancement e.g., imadjust, histeq, adapthisteq etc. But in the present case the adapthisteq function is more effective. Imadjust increases the contrast of the image by mapping the values of the input intensity image to new value. Adapthisteq performs contrast-limited adaptive histogram equalization. Adapthisteq function operates on small data regions rather than the entire image. Image processing is the first step in most algorithms based on computer vision. Such processing allows signal to noise characteristics of certain findings in the image (such as micro calcifications in particular size range) to be enhanced, while unwanted detail is suppressed.

**Discussion and Conclusion:** Each part of mammogram contrast is enhanced so that the histogram of each output region approximately matches the specified histogram (uniform distribution by default). The differences between the images are striking. Figure 1 is mammogram on which codes is applied and the Figure 2 is the image after processing. The contrast enhancement can be limited in order to avoid amplifying the noise which might be present in the image. The values are already spread out between the minimum of 0 and maximum of 255. Histogram equalization substantially changes image. Many of the previously hidden features are exposed, especially the vessels inside the mammogram. It is be noted that the part near the tumor has became clearly visible after the processing. This brief discussion about contrast enhancement techniques indicates that a collection of filters can be created to look for different types of image characteristics. This has obvious application in computer aided detection. It is an important algorithmic component

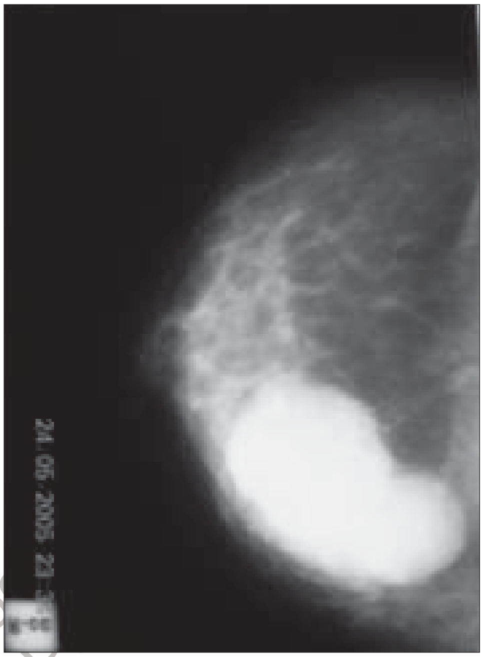

**Figure 1:** Preprocessed image

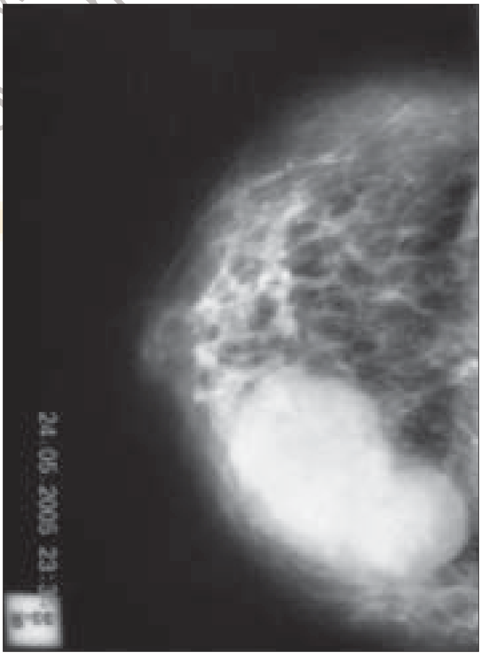

**Figure 2:** After processing in a variety of imaging applications, which are designed to optimize the workflow around measuring things (such as accurately seeing the micro calcification and vessels). The field of medical image analysis is extremely broad and enhancing the contrast of the details is a fundamental aspect of virtual implementation of an image analysis and visualization solution. Finally, an important consideration is the fact that although imaging algorithms in computer aided radiology considered to be part of the post processing domain, a tremendous amount of work has to be done before even to come at definite and concrete conclusions. Greater awareness of how an image is processed prior to display is increasingly important as radiology becomes digital. By extension, an awareness, of the capabilities of image analysis techniques may derive a greater

[Downloaded free from http://www.jmp.org.in on Friday, July 29, 2011, IP: 130.14.254.25] || Click here to download free Android application for t acceptance of the role of computer aided radiology.

**P-93**

**Dose reduction approaches in digital radiography using phantoms: A preliminary study**

Gurpreet Singh, Pratik Kumar, Ashu Seith*, Sanjay Thulkar*, Sanjay Sharma*, Pawan Popli*, S Vashisht*. *Medical Physics Unit (IRCH), *Dept. of Radiodiagnosis, All India Institute of Medical Sciences, New Delhi - 110 029, India*

**Introduction:** The one of the main focus of research in radiology is to get best diagnostic image quality without increasing the dose to the patients. The present study outlines how to improve the contrast, Density etc. of radiographs and simultaneous using the parameters which can reduce the effective dose in digital radiography. There exists appropriate photon energy and photon flux (mAs) for which best suitable match may be obtained between contrast and dose. The main objective is to find appropriate parameters which can help in dose reduction. In digital radiography there is no appreciable deterioration of image even at higher dose. Therefore optimization is needed. AEC should be set according to local needs so that minimum dose will be delivered to patient. Digital radiography provides large dynamic range as compared to conventional radiographic system.
**Materials and Methods:** The study was carried out by using the chest and Pelvis phantoms which are used for checking the quality of images. The radiographic images showed all important anatomical structures and bony land marks and looked fairly similar to patient's routine radiographs. Radiologists were unaware of the parameters used for radiography. The radiologists rated the visual impression for diagnostic capabilities of heart, diaphragm, CP/cardiophrenic angles, lung fields, bones and in pelvis radiography the various parts the various parts are bone density, hip joints, SI joints, sacral spine, public symphasis. The radiologists also give overall expression on the scale of Best/Good/Average/Poor. The field size during the experiment was not changed as all experiments were done on same phantom without disturbing the experimental setup.
**Results and Discussion:** The parameters normally used for chest radiograph at our institute are 150 kV, 0.74 mAs and 1.45 ms which gives Dose Area Product (DAP) meter reading 0.36 dGycm$^2$. But after experiment was performed using phantom the chest radiograph rated as best by the radiologists was having the parameters 150 kV and 0.31 mAs. The value of Dose area product was 0.28 dGycm$^2$ so that reduction in dose is nearly 21%. Similarly in Pelvis radiography standard parameters used are 77kV, 12.89 mAs and 12.28 ms. The DAP value corresponding to these parameters was 8.75 dGycm$^2$. But the radiograph which was rated best by radiologists were having the exposure factors 66kV, 12.42 mAs and 11.36 ms. The DAP value corresponding to these parameters was 6.3 dGycm$^2$. After calculation dose reduction was 28% with respect to factors used for standard pelvis radiograph.
**Conclusion:** The present study suggests that about 21% and 28% dose reduction is feasible in case of chest and pelvis radiography respectively. So as a result of this sharper images with better contrast and fewer artifacts in case of digital radiography with giving lesser dose to the patients. These procedures are possible in case of digital radiography. More or less its the advantage of flat panel detectors which are giving large dynamic range and more X-ray absorption. Conventional film systems are constrained by the dynamic range, so the decreased contrast can be hindrance. The relatively lower SNR of Conventional radiography system conceals the advantage of lowering the kV and mAs. Clearly another advantage of reducing the mAs is lowering the tube loading which is helpful in doing the more radiographs. This lowers the risk of overheating the X-ray tube.

**P-94**

**Experimental dosimetry of the gm plus HDR $^{192}$IR source using 0.1 CC chamber**

NP Patel, V Vijayan*, B Majumdar**. *Dept. of Medical Physics, A. H. Regional Cancer Centre, Cuttack, India, *Institute of Physics, Bhubaneswar, **Dept. of Physics, Govt. College of Science, Raipur, India*

**Introduction:** High dose rate $^{192}$Ir sources are widely used for the brachytherapy. The aim of the therapy is achieved by delivering prescribed dose as accurate as possible to the defined target volume. It is well known that the dosimetric data of each type of brachytherapy source is required as input data or to verify the dose calculations done by the treatment planning system. The recommended dosimetric data are dose rate constant, radial dose function, anisotropy function and the geometry factor. We have used 0.1 cc ionization chamber for the measurement of the radial dose function and anisotropy function. As the ionization chamber is used, we have applied correction factors for the non-uniformity photon fluence and displacement of water with air.
**Materials and Methods:** The 0.1 cc ionization chamber was used in the dosimetric study of the GammaMed Plus $^{192}$Ir HDR source. The chamber and electrometer (UNIDOS dosimeter) was supplied by PTW - Freiburg, Germany. A special jig was designed for short distance measurements, which makes possible for accurate and reproducible positioning of the chamber and as well as source. The chamber's holder was fixed mechanically at one position. The holder with source applicator (steel needle of inner and outer diameter of 1.35 mm and 1.65 mm respectively) was shifted linearly along the transverse axis, which can be placed at scale pointer with an accuracy of about ± 0.01 cm. The jig was designed such that the setup can also be placed inside the water phantom. The chamber response vs. source dwell positions were studied to know the transverse axis. For tissue attenuator factor, the measurements were performed in air and then in water for same distance consecutively, for source to chamber distances from 0.8 cm to 20 cm. The ratio of measured value from water to air was taken as tissue attenuator factor. The depth-dose measurement was performed for chamber to source distance from 0.8 to 20 cm in water medium. The measured values were corrected for non-uniformity photon fluence and displacement of water with air. The anisotropy function was measured for radial distance of 5 cm with polar angles from 10$^o$ to 170$^o$. In this process, the Polar coordinate was converted into Cartesian coordinate ($X$, $Y$). The chamber was fixed at one position assumed as the origin. The source applicator to chamber distance was taken as first coordinate $X$. The second coordinate $Y$ was set by stepping the source at specified position.
**Result and Discussion:** The tissue attenuator factor measured in this study was independent of uncertainties due to positioning and non-uniform photon fluence. The tissue attenuator factor measured in this study was found in good agreement with the famous data derived by Meisberger *et al.* The radial dose functions calculated from tissue attenuator factor at distance of 2, 5, 10 and 20 cm were 1.004, 0.950, 0.890 and 0.634 respectively. The radial dose function from depth-dose was derived from two different ways. The measured depth-doses were normalized with the dose at 1 and 5 cm respectively. Small variation was observed in radial dose functions at shorter distance where as good agreement was observed at larger distances. The mean radial dose functions calculated from two methods at distance of 2, 5, 10 and 20 cm were 0.997, 0.982, 0.906 and 0.632 respectively. The anisotropy factor was measured only for radial distance of 5 cm because of the limitation of the ionization chamber. The anisotropy function measured for angles of 10$^o$, 30$^o$, 90$^o$, 150$^o$ and 170$^o$ were 0.809, 0.958, 1.00, 0.938 and 0.774 respectively. Our measured data was in very good agreement with the published data derived using Monte Carlo calculation for the GM Plus HDR source.

**P-95**

**Radical treatment by surface mould or interstitial brachytherapy with HDR $^{192}$Ir source: An experience**

S Padhi, NP Patel*, L Pattanayak, S Banerjee, DN Singh. *Dept. of Radiotherapy and *Medical Physics, A. H. Regional Cancer Center, Cuttack, India*

Brachytherapy is a form of intensity modulated radiotherapy where very high dose is delivered to the tumor by sparing the surrounding normal tissues. Brachytherapy is used in combination with external radiotherapy or alone for recurrent lesions. Small lesion without distant

This PDF is available for free download from a site hosted by Medknow Publications (www.medknow.com).

[Downloaded free from http://www.jmp.org.in on Friday, July 29, 2011, IP: 130.14.254.25] || Click here to download free Android application for t metastasis and nodal involvement is preferred for radical treatment by brachytherapy alone. The high dose rate GM Plus afterloading unit was installed in 2001 in our center. This paper deals with our experience with radical treatment of small lesions by brachytherapy. Six patients with skin cancer were treated by surface mould brachtherapy and two with ca buccal mucosa were treated by interstitial implant. The patients with skin cancer over face and scalp after excision for biopsy were chosen for surface mould brachytherapy. For the surface mould, the plastic tubes were placed parallel in equidistance of about 1.2 cm over the wax sheets of total thickness of about 4 mm. Additional sheets were placed over the plastic tubes. Generally, the planes were in curvature shape due to the surface. Orthogonal films were taken for planning to generate the dose distribution in the treatment planning system. The dose was prescribed at depth of 1.0 to 1.5 cm from the skin. The treatment areas at prescription depth vary from 16.0 to 25.0 $cm^2$. The LQ-model was used for calculation of equivalent fractionation schemes for high dose rate. Two cases of Ca. buccal mucosa of stage $T_1$-$T_2$, $N_0$ $M_0$ were planned for interstitial brachytherapy. The sizes of lesion area were within 2.0 $cm^2$. The thickness of the lesion was within 0.8 cm. The implant was done in single plane using flexible plastic tubes with separation of 1.2 cm. The tubes were fixed by buttons. Orthogonal radiographs were taken and dose distribution was carried out in Abacus treatment planning system. The dose was prescribed at reference points at 0.5 cm from the source positions. The treatment volumes covered by prescribed dose were 12 and 16 cc respectively. For the radical treatment, total EBRT equivalent dose of 60 Gy (2 Gy x 30 fractions) was delivered. The LQ- model was used for calculation of fractionation scheme for high dose rate. The treatment was scheduled for two fractions per day with minimum 6 hours of interval. The total dose delivered by high dose rate for surface mould and interstitial implant was 42-45 Gy in 9-14 fractions. In the surface mould, skin dose was found to be very high which resulted in severe reaction. The reaction observed for the skin cancers were Grade II to Grade III. Two patients with skin cancer have two years disease free survival. Increased fractionation is advised to decrease the skin reaction. Two cases have been treated recently and other two are lost to follow up. Mild mucositis was observed after seven days of treatment which recovered very soon. The patients of carcinoma buccal mucosa treated with interstitial implant were developed Grade II mucositis in one week after treatment. Successive observation revealed healthy mucosa and regression of growth. The complete assessment of the treatment protocol needs prolonged follow up.

**P-96**

**Patients' satisfaction for imaging services in a cancer hospital: A survey based study**

Pratik Kumar, Geetanjali Arora*, Sanjay Thulkar*, Anil Pandey. *Medical Physics Unit and *Dept. of Radiodiagnosis, Institute Rotary Cancer Hospital, All India Institute of Medical Sciences, New Delhi - 110 029, India.* E-mail: drpratikkumar@hotmail.com

**Introduction:** Patient care and services in medical imaging department involves the proper response to patients' needs and expectations. Proper attention to the patients' need does not only improve the standard of the medical care but also enhances the satisfaction of the patients attending the Radiology Department. As radiology is extensively used for diagnosis and also during treatment for evaluation of efficacy of the treatment strategy, the patient satisfaction with radiology services becomes an important part of the over-all medical care in an hospital. Keeping this in mind, patients' satisfaction survey was carried out in Radiology Department of Cancer Hospital (Institute Rotary Cancer Hospital) at All India Institute of Medical Sciences, New Delhi. This study attempts to find the parameters that are important for patients in a Radiology Department and attempts to identify the scope to meet patients' expectations from radiology.

**Materials and Methods:** A survey was carried out on hundred patients who attended Radiology Department for various investigations. The survey was conducted with the help of a questionnaire with 17 questions designed to assess patients' view on the level of satisfaction with the present radiology services. The questions were related with waiting time both for examination and appointment date, environmental conditions in waiting area and investigation rooms, courtesy and help extended during investigations, the way an investigation was carried out, availability of the report etc. The patients (n=100) who attended CT, Ultrasound and X-ray radiography examinations were administered the survey and their views were recorded only after the investigations. This ensured that the patients interacted with the system and they could from the opinion based upon their experiences. Patients were also assured about the confidentiality of the data or views shared by them even from radiology staff so that they can give free and fair opinion.

**Results:** The mean age for the patients surveyed was 36.6 years for male (n=60; 60%) and 38.7 years for females (n=40, 40%). 70% of the patients were married and rest were singles. As expectations maybe linked with patients' background in terms of education, occupation and financial status these were also probed. 76% of respondents had educational qualifications as matriculation or above. Most of the patients were engaged in some sort of service (24%) followed by students (21%) and self employed (20%). The next biggest group was of house-wives (18%). About half (45%) of the patients belonged to family with monthly family income of Rs. 15,000 to 25,000. However, the lower family income of less than Rs. 15,000 and higher one of Rs. 25,000 to Rs. 40,000 were 26% and 25% respectively. Majority of the patients (69%) surveyed had visited the hospital earlier as well. Therefore, their views were based upon their experiences with previous visits as well and may be taken as more near to reality. 70% (14 out of 20) patients for ultrasound and 61% (11 out of 18) patients for CT scan had to take prior appointment as against 16% (10 out of 62) patients took appointment for X-ray radiography. It showed that appointment list for US scan was slightly longer than CT scan and probably showed that there is a need to reschedule the priority in ultrasonography to bring the appointment time down. Survey indicated that, in general, patients and attendant were satisfied with the hygienic condition prevailing in the scan rooms. The attendants of 57% of the patients were not allowed inside the examination room yet the majority (61%) believed that the presence of attendants is conditional depending upon the state of the patient. It showed that patients' attendants are responsive to the need of the hospital as well and, therefore, are quite receptive to the any education hospital wish to provide them (e.g., regarding radiation safety, crowding etc.). About 90% patients were informed about the further course of action after the investigations. 18% patients had to undergo repeat examinations due to various reasons which included clinical requirements as well. Out of these repeated examinations, about half (56%) were informed about the cause of repeat examination. Probably this is the area there is scope to improve the communication with the patient. The survey revealed that majority (64%) were satisfied with the imaging services while next 27% were neutral. 7% patients were categorized dissatisfied while 2% expressed their opinion towards very satisfied group.

**Conclusion:** In this way, the study provides an opportunity to improve our radiological imaging services to patients which can raise the patients' satisfaction level.

**P-97**

**Quality assessment of bedside portable chest radiography: A comparison with OPD based fixed X-ray unit chest imaging**

Pratik Kumar, Nidhi Gupta*, SR Manwar**, TC Chavan**, Anil Pandey, S Thulkar*, S Vashisht*. *Medical Physics Unit, Dr. BRA IRCH, *Department of Radiodiagnosis, **WHO Trainee at Medical Physics Unit, Dr. BRA IRCH, All India Institute of Medical Sciences, New Delhi, India.* E-mail: drpratikkumar@hotmail.com

**Introduction:** The chest radiography is the most common radiological examination accounting for the maximum volume of the radiological imaging processes in any hospital. Portable or bedside radiography is substantial among chest radiographs and this is especially true for emergency and critical care situations. It is futile to say that bedside radiography plays a pivotal role for determination of diagnosis and management of critical care patients. Yet, the quality of bed-side radiography in general and chest radiography in particular is seldom

This PDF is available for free download from a site hosted by Medknow Publications (www.medknow.com)

[Downloaded free from http://www.jmp.org.in on Friday, July 29, 2011, IP: 130.14.254.25] || Click here to download free Android application for t as satisfactory as its counterpart from fixed radiographic machines. Unfortunately, it has been accepted unofficially in imaging community that there is little to be done to improve the quality of bedside radiography. The study was conducted in order to alter this mindset that bedside radiography can't be improved. It is the first step towards the identification of weaker areas of bedside chest radiography. The study attempts to quantify image quality of bedside chest radiography with respect to fixed machine chest radiography and analyses the areas which needs further augmentation.

**Materials and Methods:** For comparison of image quality between bedside chest radiographs and fixed machine chest radiographs we developed a quality scale containing seven parameters. They were film density, patient compliance of deep respiration, quality of reproduction of thorax, rib cage, vascular pattern in lungs, heart and aorta, diaphragm and castro-phrenic angles, retro-cardiac lung and mediastinum. The quality of reproduction of these physical and anatomical features were graded by the radiologists for a full marks of 100. The quality assessment was carried out by two radiologists in 300 bedside chest radiographs performed by the portable X-ray units and 300 routine OPD chest radiographs performed by fixed X-ray units. It was ensured that films' processing and reading conditions were same for the radiographs taken by portable and fixed X-ray units. Prior to the study portable and fixed X-ray units were subjected to quality assurance tests so that their performance may be comparable.

The films with overall zero grade (marks) for all parameters or with a dominating factor disturbing the image quality to such an extent that the images have no appreciable diagnostic values were rejected.

**Results and Discussion**: The study showed that only 3 (1%) OPD chest radiographs performed by the fixed X-ray unit were rejected as compared to 89 (29.7%) of radiographs by portable units. This buttressed the common belief that majority of portable radiographs are of lower quality. The major causes for rejection of portable X-rays were inappropriate exposure (19.3% cases of over and 21% cases of under exposure), respiration blur (6%) and positioning error (11.7%). Apart from rejected films, 36 portable films had grades below the marks 30 (poor quality) while this figure for OPD fixed unit radiographs was only 4. Here again, inappropriate exposure (over and under) was the culprit. It is obvious that in screen-film radiography where radiographer has to select the parameters on his wisdom there is a need for re-assessment of the situation in the case of bedside radiography. Probably development of an exposure-chart dedicated to bedside portable radiography and orientation of radiographers to the specific and specialized need of this is warranted. Again, it was analyzed from the data that 70 portable radiographs against 13 OPD radiographs had objectionable respiration blur. Probably it is due to the fact that patients undergoing bedside radiography are generally more compromised than the OPD patients. There were 11.7% rejection due to gross positioning faults in bedside chest radiographs as opposed to 0.33% in OPD fixed X-ray radiographs. Again, 27 bedside films got marks below 30 and another 50 films achieved marks between 40 to 50. The number of OPD fixed X-ray units films falling in these two marks categories were 4 and 6 respectively. This is the arena where we may make the radiographers aware of our study and probably the situation may be ameliorated despite our realization that the positioning of ambulatory patients may not be improved more than a limit. However, the increased awareness on the part of radiographers may reduce the bedside films with poor markes (grades) in positioning. Visualization of retro-cardiac lung and mediastinum affected the quality in most of the OPD films. This parameter scored upto 70 marks in 64% films (190 out of 297 accepted OPD films) and rest 36% (107 films) scored 80 and above signifying good quality. Another parameter which was the next frequent degrader of quality in OPD radiographs was the vascular pattern in lung and peripheral vessels which scored upto 70 marks in 56.2% films. But in case of bedside films the retro-cardiac lung and mediastinum (which was the most frequent poor performer in OPD films) was the second frequent degrader of bedside films' quality with 75.8% (160 out of 211) films scoring upto 70 marks. The border of heart and aorta was the most frequent degrader of quality in bedside films. This parameter could get upto 70 marks in 80.6% films (170 out of 211 accepted films) making this criteria the most frequent poor performer for bedside films.

**Conclusion:** This study identifies the weakness in bedside chest radiography as this portable radiography has long been neglected for quality images. Digital radiography may also be explored for improving quantity in bedside radiography.

### P-98

### Analysis of radiation dose from exposed radiochromic film using flatbed document scanner and the software image

MR Barman, EB Rajmohan, Pratik Kumar. *Medical Physics Unit, IRCH, All India Institute of Medical Sciences, New Delhi - 110 029, India*

**Introduction:** Radiochromic dosimetry films are self-developing film media that have been developed for the measurement of absorbed dose and the mapping of radiation fields. The films develop a distinctive and characteristic color upon exposure to ionizing radiation and become progressively darker in proportion to absorbed dose. These media can be handled in normal room light, thereby eliminating the cost of darkroom space. Since they are self-developing, the costs and inconvenience of post-exposure development are eliminated and there are no hazardous chemicals or waste to dispose of. Radiochromic dosimetry films are available optimized to high or low energy photons as well as particle radiation. The films are tissue equivalent, light and flexible and may be cut to any desired shapes or sizes. The construction of radiochromic dosimetry films even permit them to be immersed in water for short periods of time. Radiochromic dosimetry films have responses that are not only dose-rate independent, but they do not exhibit fractionation effects. Their response is also energy-independent down to about 0.2 MeV. The color change in radiochromic dosimetry media may be measured with any densitometer or scanner. Several types of instruments (single point densitometers and one or two dimensional position sensitive light detectors) are used for the two dimensional film densitometry. The study utilizing the above mentioned types of densitometers are reported in our earlier abstracts. The densitometers required for reading these films are expensive reflective densitometer equipped with red-light as these films have major absorbance peak at about 674 nm and a minor peak at about 615 nm wavelengths.

**Scanner and Imagej Software:** The commonly used flat bed document scanner may also be used for reading density on the radiochromic film although it has not been designed for this purpose. It employs a fluorescent light source with a broad band spectrum and linear charge coupled device array detectors. The exposed images are scanned with proper care so as to avoid the uncertainty in the final measurement outcome. ImageJ software or image processing and analysis in Java is a domain freely available from the internet. Due to its users' friendly interface and compatibility with all types of image formats it is preferred in all fields of image processing and analyzing. Image J runs on Linux, Mac OS 9, Mac OS X and Windows. ImageJ and its Java source code are freely available and in the public domain.

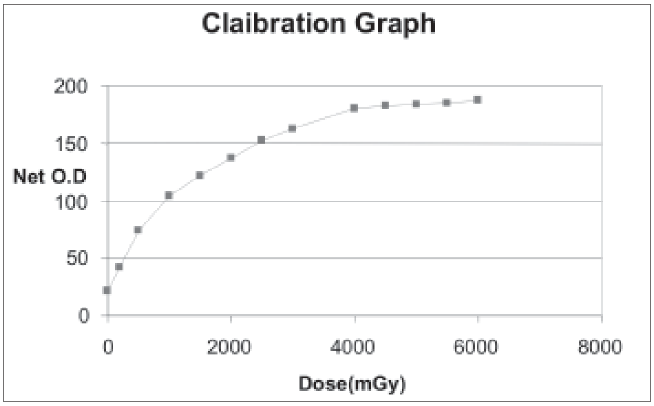

**Figure 1:** Claibration graph

This PDF is available for free download from a site hosted by Medknow Publications (www.medknow.com)

[Downloaded free from http://www.jmp.org.in on Friday, July 29, 2011, IP: 130.14.254.25] || Click here to download free Android application for t

It opens and saves all supported data types as TIFF (uncompressed) or as raw data, GIF, JPEG, BMP, ASCII, DICOM, FITS, PNG and PGM. It can also open TIFFs, GIFs, JPEGs, DICOMs and raw data using a URL and many other formats using plugins. Image J is the world's fastest pure Java image processing program. It can filter a 2048 x 2048 image in 0.1 seconds, i.e., 40 million pixels per second. It supports smoothing, sharpening, edge detection, median filtering and thresholding on both 8-bit grayscale and RGB color images. It interactively adjusts brightness and contrast of 8, 16 and 32-bit images. It measures area, mean, standard deviation, lengths, angles, pixel value, min and max of selection or entire image. It uses real world measurement units like millimeters and calibrates using density standards and can generate histograms and profile plots.

**Materials and Methods:** First of all, for calibration procedure 5 x 5 $cm^2$ Gafchromic film pieces were cut and exposed to known doses (200, 500, 1000, 1500, 2000, 2500, 3000, 4000, 4500, 5000, 6000 mGy) from cardiac angiography machine at mostly used but fixed kVp and measured by calibrated Radcal ionization chamber so that the uncertainty of energy is minimized. The resulted optical densities on films were scanned by ordinary flat bed document scanner. Each film was scanned five times to minimize the variation (scanner noise). Flat bed document scanners designed for high quality photographic scanning with an option to operate in transmission mode usually employ a fluorescent light source with a broad band emission spectrum and linear charge coupled device array detectors (CCD). The exposed images were scanned with proper care so as to avoid the uncertainty and variation in subsequent scanning. The scanned images were analyzed as pixel value or net O.D. (Optical Density) using ImageJ software. Color management is the essential part of the image acquisition, image processing and image output (pixel vale or net OD). Most image input/output devices processes color in RGB region of images. The color management is usually done in image processing tool box of ImageJ, which includes color management module. The images were imported in the toolbox window and the pixel values in the region of interest (ROI) were measured. As we noticed that the true images have variation in the RGB pixel values, a complement of the true scan image (gray scale image) was obtained. The pixel values for all the five scans for one set is made. The net O.D. is obtained by subtracting the averaged pixel value of the back ground film from the averaged pixel value of the exposed film pieces. Net O.D. = Pixel Value (Exposed) - Pixel Value (Background).

**Results and Discussion:** [Figure 1]. A calibration graph for the gafchromic film using document scanner with Image J software was plotted. The patient films used in the Percutaneous Transluminal Coronary Angioplasty (PTCA) cardiac procedure were read from this calibration curve and the results were compared with the other international studies. They were found to be correlating. In this study Gafchromic XR type R film dosimetry protocol has been standardized for its use with a document scanner and ImageJ software. In our past experience with other softwares like Ulead Photoimpact-7 and MATLAB 6.5, the Image J software was found to be simpler and users' friendly. This software can be used even by those users who are not familiar with programming languages. In this study both JPEG and TIFF formats of images were used and found that the Net O.D. in TIFF images was more closer to the international study than JPEG since TIFF formats applies lossless compression. Unlike JPEG, TIFF files can have a of 16-bits per channel in addition to the standard 8-bits per channel and can store multiple layered images. This is the main reason for the lesser variation in the pixel value in Tiff images.

**Conclusion:** ImageJ software alongwith a flatbed document scanner can be utilized to assess the doses from Gafchromic XR type R film dosimetry media. It was found that TIFF images are more reliable and the values were nearer to the standard one than JPEG format.

**P-99**

**Commissioning of an infrared marker-based frameless patient positioning and localization system**

Reena Phurailatpam, Rajeshri H Pai, Tejpal Gupta, Deepak Despande[#], Shyam K Shrivastav*. *Department of Radiation Oncology, ACTREC, Kharghar, Navi Mumbai, [#]Medical Physics, *Radiation Oncology, TMH, Mumbai, India.* E-mail: rphurailatpam@actrec.gov.in

**Introduction:** An infrared marker-based frameless patient positioning and localization system for extra-cranial radiotherapy (ExacTrac; BrainLab AG, Germany) was installed at our hospital. This paper addresses the commissioning and evaluation of the ability of ExacTrac for accurate isocenter positioning using electronic portal imaging device (EPID).

**Materials and Methods:** The Exactrac System consists of ceiling mounted two Infrared Cameras (IRC) and one Video Camera (VC), Infrared Reflective markers (IRRM) and supporting software.

**The calibration of ExacTrac includes:** IRC Calibration, VC Calibration and Isocenter Calibration. IRC Calibration: IRC calibration phantom having 25 IRR markers arranged in a fixed geometry was positioned within the camera's field of view and near the isocenter such that the camera detects all the markers. The analyzing software compares the detected 3D co-ordinate of these markers with an internal reference look-up table and displays the variation. The position of the phantom was adjusted till the variation is within the specified tolerance limit and calibration is accepted.

**VC Calibration:** After successful calibration of IRC, the VC calibration was carried out following the same procedure as describe before. Once the VC detected all the markers within the specified tolerance limit, calibration is accepted and is ready for the last step of isocenter calibration.

**Isocenter Calibration:** The isocenter calibration aligns the Exactrac system with the isocenter position of the Linear accelerator (LA). An isocenter calibration phantom (10 cm x 10 cm x 2 cm box with five IRR markers) was positioned on the couch within the field of view of the IRCs. The engraved lines on top face and sides of the phantom were aligned precisely with the collimator cross wire projection with gantry at $0^o$, $90^o$ and $270^o$. Then the positions of the five IRR markers were captured on the IRC and a video image of the phantom was acquired on the VC. Accuracy in Isocenter localization by the ExacTrac system was evaluated on a solid phantom simulating a patient's study. Five IRR markers were placed on five randomly selected positions on the phantom surface and another one radio opaque marker of 2 mm diameter was kept at 5 cm depth representing the tentative isocenter. CT scan of 3 mm slice thickness was taken covering the whole phantom and virtual simulation was performed keeping the isocenter at the center of the radio-opaque marker. The isocenter was marked on the phantom surface. The plan was imported at the ExacTrac workstation, which automatically localizes the IRR markers from the CT images and pre-defined isocenter position and store as a reference look-up table. The phantom along with the markers in the same orientation as in planning scan was randomly placed on the LA couch. The ExacTrac system identifies the positions of the markers and compared with the reference look-up table. The difference in isocenter position was evaluated in six degree of freedom including three transnational and three rotational angles. Phantom and table position was adjusted till the variation is within the set tolerance limits. Orthogonal electronic portals were taken and source-to-surface distance was checked.

**Result:** A precise match between the wire-frame model grid of the isocenter calibration phantom generated by the IR cameras based on the captured co-ordinates of the IRR markers and video image verifies that both infrared and video camera system are successfully calibrated and synchronized to the same coordinate system. The ExacTrac system could position the isocenter within ± 1 mm and ± $1^o$ accuracy in transnational and rotational coordinates respectively. The set up SSD as measured over the surface of the phantom was matching well with the planning system measurement. After removal of upper slab of phantom, the optical crosswire and laser projections were found to be exactly on the center of radio opaque marker placed at the center of chamber holder.

**Conclusion:** The calibrations of cameras are very important and it should be done very accurately. This study performed on the flat and static phantom shows good accuracy achieved by the ExacTrac system. It should be further evaluated with reference to laser-based systems

This PDF is available for free download from a site hosted by Medknow Publications (www.medknow.com)

[Downloaded free from http://www.jmp.org.in on Friday, July 29, 2011, IP: 130.14.254.25] || Click here to download free Android application for t on patients before being adopted in clinical practice.

### P-100

### 3D Planning and dosimetry for customized mould in treatment of carcinoma of penis by mHDR

AK Srivastava, SP Mishra, B Paul*, RR Ghosh*, BK Mishra*. *Department of Medical physics and *Radiation Oncology, Regional Cancer Centre, Kamala Nehru memorial Hospital, Allahabad - 211 002, (UP), India*

**Introduction:** Wax and y-dent moulds have been extensively used in positioning the teletherapy treatment of Carcinoma of penis. However, brachytherapy could be a preferred choice of treatment in Ca penis as it provides uncomplicated reproducibility, delivery of high dose in a well defined volume without exceeding the tolerance of critical structures and non-invasive modality for catheter insertion. The locally invasive Ca-Penis holds the promise of complete cure if the target volume is adequately covered using a cylindrical mould for brachytherapy. In this study the mHDR brachytherapy has been utilized for the treatment of Ca Penis by customized Cylindrical mould for complete coverage of the treatment volume. The cylindrical mould brachytherapy technique is devoid of large scatters to neighboring areas and shielding requirements of critical structure viz testis etc. that are necessary in teletherapy delivery.
**Materials and Methods:** In this study customized cylindrical mould were prepared by obtaining the impression of penis for delivering the mHDR Brachytherapy to the target volume. The catheters were placed on the surface of cylindrical mould by drilling a hole on the circumference, which were kept at 1.00 cm apart. An innovative approach for 3D dose distribution was made by obtaining coordinates of each catheter on the circumference of cylinder cross-section with respect to the center of the circle being at (0,0). The complete 3D cylinder was thus reconstructed using 3D Plato TPS for obtaining the desired dose distribution. The length of the cylinder was the target length of the penis to be treated. The customized cylindrical mould thus prepared and reference dose selected for treatment are detailed in Figures 1 and 2. The Perish system of dosimetry was utilized for distribution and dosimetry. The reproducibility of cylinder was insured by using markers at the root of the penis and a reference dose of 300cGy BD for 6-7 fractions were delivered. Phantom dosimetry was used for verification of the dosimetry. The 3D TPS optimized the dose distribution by arranging the dwell time along the length of the implant tube in the cylindrical mould. In this presentation the details of preparation of cylindrical mould, methodology for identifications of coordinates for area reconstruction, planning and target volume assessment, selection of reference isodose will be demonstrated.
**Results and Conclusion:** It has been found that customized cylindrical mould provides a noninvasive modality for the treatment of Ca Penis using mHDR. The reproducibility of the mould position in each sitting has been ensured for reproducibility of reference volume. The result obtained has better cosmetic results, no disconfiguration of penile anatomy were noted and doses to the critical structures were within their limit of tolerance. This modality provides an effective method for treatment of such lesions, which is innovative and cost effective using mHDR.

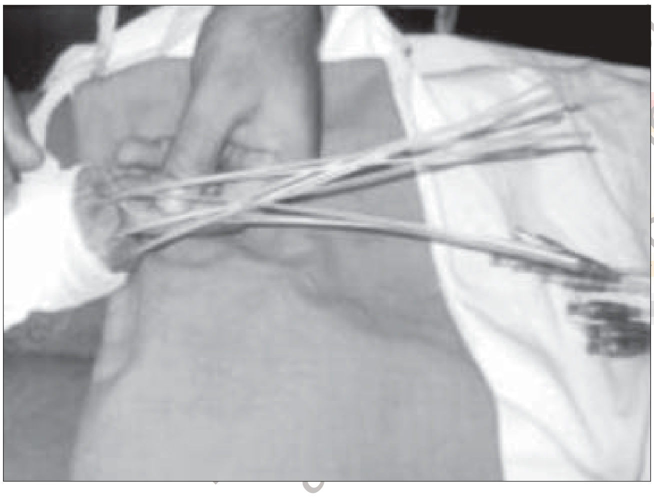

**Figure 1:** Customized cylindrical mould in position

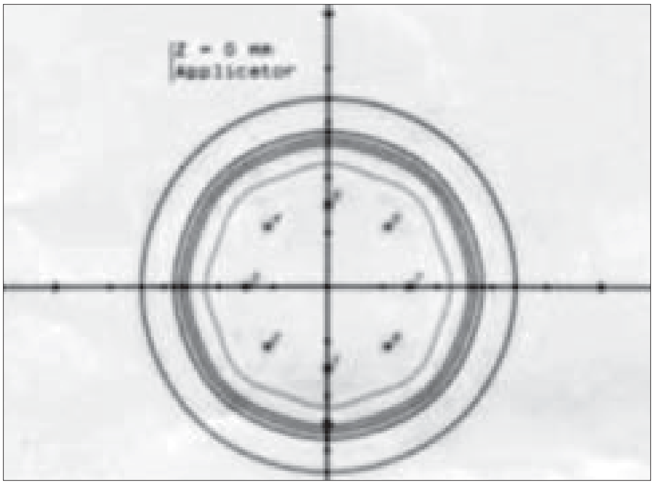

**Figure 2:** Cross section of dose distribution of cylindrical mould implant (green line depicting 90% isodose)

### P-101

### Use of an amorphous silicon electronic portal imaging device for relative dosimetry and quality assurance of linear accelerator

M Karthick Raj, KJ Maria Das, SK Senthil Kumar, AK Maurya, S Kumar, NR Datta. *Department of Radiotherapy, Sanjay Gandhi Postgraduate Institute of Medical Sciences, Lucknow, India*

**Introduction:** Quality assurances are playing a vital role in precision radiotherapy. In this study we intend to use the electronic portal imaging device (EPID) for relative dosimetry and routine quality assurance procedures on a linear accelerator.
**Materials and Methods:** EPID was mainly developed for the purpose of patient treatment position verification and replaced the radiographic films, which was originally used for the beam portal verification of a patient. In the mid of 1990'S liquid filled ionization chamber and camera based EPID were commercially available, but the image quality was not satisfactory. The recent developments using amorphous Silicon (a-Si) EPID system considerably improved the image quality and have been accepted as the device of choice for radiotherapy treatment verification imaging. These images acquired using a-Si EPID system has now been explored for the purpose of relative dosimetry and routine quality assurance procedures on a linear accelerator with devices for beam modulation. This study was carried out using a-Si 500-II EPID system on a Clinac 600C linear accelerator with an 80 leaf millennium multi-leaf collimator (MLC) of Varian Medical Systems, USA and a BrainLab micro multi-leaf collimator (mMLC). This EPID System includes an image detection unit (IDU-20) with image acquisition system (IAS3) enabling a maximum of 30 frames per second and has an active area of 40 x 30 $cm^2$ with a pixel matrix of 512 x 384. Each pixel corresponds to 0.784 $mm^2$ at isocenter. Images were acquired keeping the detector position at isocenter. The dark field and flood field were obtained to correct the detector response as well as to keep the absolute pixel values proportional to the incident fluence. The following procedures were carried out to illustrate the use of EPID.
**Open beam and Wedge Profiles:** The images are acquired for open and wedge beams in the integral mode for 100 MU and all the frames were averaged to obtain the final image. The EPID images were calibrated using commercially available Dicom software to obtain the pixel values, normalized to the central axis pixel and obtained the off-axis profile. The profiles generated from radiation field analyzer (RFA)

[Downloaded free from http://www.jmp.org.in on Friday, July 29, 2011, IP: 130.14.254.25] || Click here to download free Android application for t

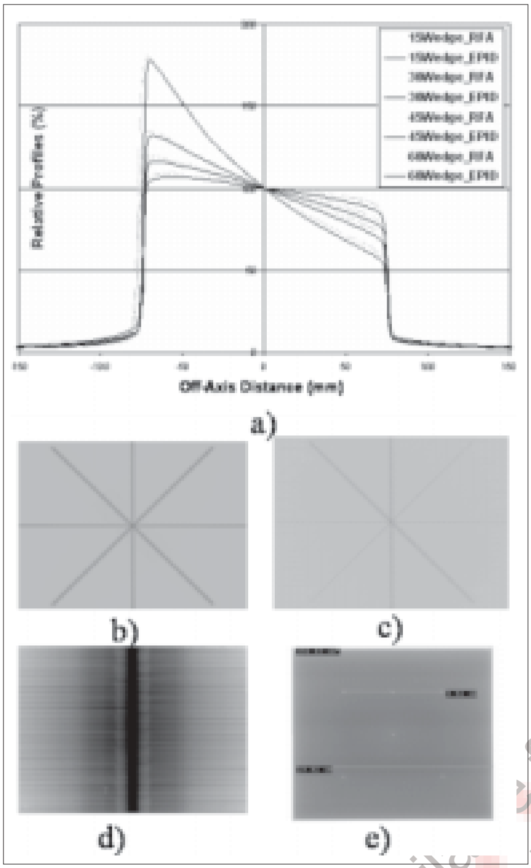

Figure 1: a) Profile comparison of RFA and EPID, b) Collimator spoke shoot, c) MLC spoke shoot, d) MLC leaf positional accuracy and e) Optical and radiation field coherence were transferred on ASCI data. A graph is plotted between the RFA and EPID data for open and 15, 30, 45 60 degree wedge beams [Figure 1a]. The profiles were also analyzed for the penumbra characteristics of RFA and EPID data.

**Spoke shoot test:** In order to study the accuracy of the collimator, MLC and mMLC rotation axis, images were obtained for various collimator angles of 0°, 45°, 90° and 315° with a field size of 0.5 x 40 $cm^2$. Each collimator angle image is recorded in the high quality mode (0.74 MU / frame, averaged over 4 frames and doserate of 240 MU/ min) with the detector positioned at the isocenter. These Dicom RT images were converted into a Tiff format images with the same pixel value. Using a standard imaging software (Paint shop pro) all the pixel values were arithmetically summated to obtain the spoke shoot pattern. The same procedure was repeated with the MLC and mMLC [Figures 1b and c].

**MLC leaf positional accuracy:** Various MLC leaf positions were designed in the shaper software module and transferred to the 4D treatment console. The EPID images were acquired in the high quality mode and analyzed using ImageJ software. The images were calibrated and the obtained MLC leaf positions were compared with that designed in the shaper module [Figure 1d].

**Inter and intra leaf leakage test**: The images were acquired in the integral mode for a 100 MU for the intra and inter leaf leakage. The calibrated images for the pixel value were compared with that obtained using ionization chamber and film measurements.

**Optical and radiation field coherence:** To verify the optical and radiation field coherence, images were acquired in the integral image mode for 100 MU with a field size of 20 x 20 $cm^2$ and positioning the EPID at the isocenter. The field edges were marked for field sizes of 10 x 10 and 20 x 20 $cm^2$ with the lead markers to identify the optical field edges. The image obtained was calibrated and analyzed for coherence of the optical field and radiation field [Figure 1e].

**Results and Discussion:** The profiles obtained for open and wedge beams were compared with the RFA and observed to be within a maximum 2.4% in the non dose gradient region and variation of 2 to 5 mm in the penumbral region. This deviation could be due to that, the EPID does not exactly simulate the water equivalent material for builup. The rotational axis accuracy of the collimator, MLC and mMLC using spoke shoot were in agreement with the film measurements. All the routine quality assurance procedures carried out using EPID was found to be in agreement with the film and ionization chamber measurements. The EPID is independent of energy and has a pixel resolution of 0.78 mm and so found to be adequate in using it for routine quality assurance procedures and proves to be a useful tool for the physics.

### P-102

### Changes in physical characteristics of tissue/ tissue environmental may be the cause of cancer: A theoretical approach: A new theoretical concept of mathematical zoology

RM Chandola. *Medical Physics Research Laboratory, Department of Radiotherapy, Pt. J.N.M. Medical College and Dr. B.R. Ambedkar Memorial Hospital, Raipur - 492001 Chhattisgarh (India).*
*Correspondence Author:
House No 40, Near Bal Udyan, Sector - 01, Shankar Nagar, Raipur Chhattisgarh, India

This new concept is being made on the basic concept of work energy principle of physics. Work and energy are vice versa to each other. If the tissue works whether we define if in terms of it's metabolic activity or any activity, for this surely a energy is required. This energy may be either electrostatic potential or the potential due to magnetic field across the tissue or the group of tissue. The change of this any kind of potential may be the cause of tissue converting into the cancerous tissue. Therefore this potential is needed to be defined in mathematical form.

### P-103

### Automation of low energy electron irradiation system for medical applications

SK Mahapatra, BJ Patil, ST Chavan*, SD Dhole, VN Bhoraskar. *Department of Physics, University of Pune, Pune, India, *Sameer, I. I. T, Powai, Mumbai, India*

Introduction and Objective: With the advent of various electron sources, the low and high energy electrons have become a viable option for studying the basic research as well as for the medical applications. The low energy electrons (eV to keV) do not penetrate deep, because of its low Linear Energy Transfer (LET ~ 0.03 keV/μm) along the path. These low energy electrons have two major effects; first, the negative charge particle travel in contorted path and has range from nm to ¼m in water. Second, multiple ionization (1 keV to 100 keV) occurs in the immediate vicinity (few nm) of the track. It also generate cluster of high ionization density along an irregular path. Therefore, the short range of these low energy electrons is important to the radiosensitive target such as DNA for radiotherapic effectiveness. This is because of the energy density drops in as a function of distance in nm. The electron depth dose characteristics produce a high skin dose, but exhibit a fall off after only a few nm to μm. This aspect of low energy electron is used for treating skin lesions, post surgery, mastectomy scar in breast cancer patient, nodal areas etc. In addition, depending on the dose of an irradiation, the various results are possible such as genetic changes, sterilization, delay of growth, chemical decomposition etc. However, the last few decades, the keV energy electron sources have gained importance because of their requirements in generation of Grenz rays, which is used in Therapy for Skin Disorders. In the present paper, to peruse the medical applications, an automation of low energy electron source has been made for the accurate measurements of beam parameters such as energy, current, dose, voltage etc. This system can provide electrons of different energy over the range of 50 eV to 20

This PDF is available for free download from a site hosted by Medknow Publications (www.medknow.com)

[Downloaded free from http://www.jmp.org.in on Friday, July 29, 2011, IP: 130.14.254.25] || Click here to download free Android application for t keV and beam current upto 50 μA at all electron energies.

**Materials and Methods:** The electron source is pierce type electron gun and it consists of four electrodes namely cathode, focusing electrode, control electrode and anode. They are made up from stainless steel material. These four electrodes are separated through ceramic rods with the desired distance between them. The oxide coated tungsten is mounted on the focusing cathode, mainly used as electron emitting element through the thermionic process. The electrons emitted from the filament can be focused and accelerated by the potentials between anode, cathode and grid respectively. This assembly of the electron gun mounted in the S.S chamber, where the actual experiments can be performed. The automation of beam parameters such as filament voltage (VF), beam energy (EB), beam current (IB) and sample current (IS) of the electron source have been introduced to measure the precise dose and to control the parameters. The signal conditioning circuit is specially designed for buffering, scaling of data and control the parameters. The measurement parameter such as beam current (IB), beam energy (EB), filament voltage (VF) are controlled by analog outputs and the data is acquired by analog inputs through serial interface, whereas the sample information has serial communication interface. The system is provided with Windows based software for easy setting and acquisition of data. Finally one can set the desired conditions with precession and accuracy for the measurement of dose received by the sample. The emission current (IE) with filament voltage (VF) at different energy electrons indicates that the threshold of VF decreases as increases in the energy and can be obtained the maximum current 200¼A in the range 17 to 18 volts. It is observed that the beam current increases with increasing electron energy and appreciable change was also found in increase in VF. This particular property of electron gun is advantageous in the medical drugs and therapy where the precise dose can be controlled as per the requirements.

**Results and Discussion:** The feasible experiment was performed using this system by irradiating few polyimide samples. The polyimide was chosen because it contains H, C, N, O and which are also the constituents of the human body. So the effect of low energy electron in polyimide shows the equivalent biological effects.

**Conclusion:** Automated low energy electron irradiation system can successfully implemented to change the properties of the polyimide and can also be used as a treatment for medical drugs, food processing and superficial therapy.

**P-104**

**Angular distribution of Bremsstrahlung radiation from 6 and 15 MeV pulsed electron beam incident on Tungsten target**

BJ Patil, SK Mahapatra, ST Chavan*, SN Pethe*, VN Bhoraskar, SD Dhole. *Department of Physics, University of Pune, Pune - 411 007, India, *Sameer, IIT Pawai Campus, Mumbai - 400 076, India*

**Introduction:** In recent years, there has been rapid growth in the use of low and medium energy electron accelerators for medical and industrial applications. Electron accelerators are very useful in generating continuous energy gamma rays called bremsstrahlung radiation. The bremsstrahlung yield1 is defined as the number of photons of energy E per unit energy from the target which would reach a given point in a vacuum per unit solid angle per electron incident on the target.

$$\frac{dS}{dE} = \frac{1}{N_e}\frac{d^2 N_\gamma}{dEd\Omega}$$

where $\left(d^2 N_\gamma / dEd\Omega\right)$ dE is the number of photons with energy between E and E + dE which exit the target and reach a given point per unit solid angle and Ne is the number of electrons incident on the target. The spectral distribution of bremsstrahlung is of general interest in applications such as radioisotopes production, oncological hadrons therapy, conventional radiotherapy and gamma ray radiography. However, the angular distribution of bremsstrahlung radiation is also equally important for the production of radionuclide through the photo nuclear reactions. Thus, the dimension of the target should be optimized to get the maximum photon fluence rate in a small volume of vessel. This kind of targets can generate neutrons in the thermal, epithermal and fast range and can be used effectively for the neutron therapy and boron neutron capture therapy programme for cancer treatment. Because neutron are more effective at killing tumor than the conventional therapy. Therefore, in the present paper, keeping the importance of the bremsstrahlung radiation, neutron targets, neutron therapy and BNCT programme, the angular distribution of bremsstrahlung at various angles for different Z of the material have been studied. This will certainly help to control the radiation dose at 1/2 and 1/10$^{th}$ intensity of the bremsstrahlung yield.

**Materials and Methods:** The Fluka computer code was used in the present work 2 and is now found to be a most accurate method available to calculate bremsstrahlung spectrum for various targets as well as radiation effects in body tissue and determine doses. In general, when high energy particles, in the form of electrons/photons hit matters, they travel through the material, interacting with atoms and their nuclei in various ways. The path of each particle can be modeled as a random walk as collisions with atoms occur with well defined probability. Each individual interaction generates many more electrons/photon due to collisions as it travels through matter. The same interaction process is simulated by Monte Carlo technique in Fluka 2. Fluka is a general purpose tool for calculations of particle transport and interactions with matter, covering an extended range of applications. Fluka can simulate with high accuracy, the interaction and propagation in matter of about 60 different particles, including photons, electrons and neutrons. Therefore, it was used to decide the geometry of the target to generate bremsstrahlung spectra from high energy electrons. The present simulation work is carried out for SAMEER's linear accelerator and Pune University's circular accelerator by using their standard parameters. The electron energy, repetition rate, average and pulsed beam current of SAMEER has 15 MeV, 200 PPS, 80 μA and 100 mA, whereas Pune university has 6 MeV, 50 PPS, 1 μA and 10 mA. Using these inputs, the simulation was carried out to generate bremsstrahlung spectrum for tungsten target having thickness 0.42 cm for 15 MeV energy and 0.22 cm for 6MeV electron.

**Results and Discussion:** It is observed that as the angle increases right from 0° to 180°, the bremsstrahlung yield found to be decreases exponentially. However, it is also found that at 15 MeV energy of the electron beam, the bremsstrahlung yield increased by 6 times than the 6 MeV electron beam. The ratio of the bremsstrahlung yield at 00 to 1800 is lower in the case of 6 MeV electron beam as compared to 15 MeV electron beam. This result attributes that as the energy of the incident electron increases, the bremsstrahlung ratio increases. It is also observed that the average energy of bremsstrahlung spectra decreases with increase in angle for 6 and 15 MeV electron beam. It is also found that the bremsstrahlung peak is slightly shifted towards the lower energy as increase in angle from 0° to 180°.

**Conclusion:** The results obtained by Fluka code, the angular distribution of bremsstrahlung spectra for tungsten target at 6 and 15 MeV pulsed electron beam are useful in controlling the precise and accurate level of dose for therapies. This angular data of bremsstrahlung radiation will provide the information about the desired radiation dose to be given to the patient as per their requirement.

**P-105**

**Development of correction technique for transmission contamination in $^{67}$Ga/$^{153}$Gd simultaneous emission and transmission tomography**

SC Kheruka*#$, BF Hutton*#, AZ Kyme*, RL Hatton*, S Ganbhir$, AK Shukla$. **Departments of Medical Physics and Nuclear Medicine and Ultrasound, Westmead Hospital, Sydney, NSW, Australia, #Centre for Medical Radiation Physics, University of Wollongong, Wollongong, NSW, Australia, $Department of Nuclear Medicine, SGPGIMS, Lucknow, India*

**Introduction:** Single photon emission computed tomography (SPECT) is a tomographic technique used for imaging and measurement of radio active tracer distribution within the body. The effect of attenuation and compton scatter severely regulates the quantitative accuracy of

This PDF is available for free download from a site hosted by Medknow Publications (www.medknow.com)

[Downloaded free from http://www.jmp.org.in on Friday, July 29, 2011, IP: 130.14.254.25] || Click here to download free Android application for t

SPECT images. In order to obtain post-injection transmission data, the transmission and emission data are acquired simultaneously using $^{153}$Gd and $^{67}$Ga respectively. Spillover from the 93.5 keV photopeak and downscatter from the higher energy photopeaks (184 keV and 300 keV) of 67 Ga contaminate the transmission data. The aim of this work is to develop a correction method to produce a corrected attenuation map that can be used for attenuation correction in the emission reconstruction.
**Materials and Methods:** Proportion of total events of scatter in the upper energy window (184 keV) was determined by convolving the upper energy window with a bi-exponential scatter function of the form S=k/N (A*exp (-br) + exp (-cr)), where r is radial distance, N normalizes the bi-exponential function to a unit integral and k is scatter fraction. The relationship between scatter fraction, spillover fraction and measured attenuation path length (md) was established. The corrected transmission projections were reconstructed and the corrected attenuation map was scaled to take into account the difference in photon energies of the two radionuclides. This was then deployed to reconstruct the combined emission images (90 + 184 keV) of a thorax phantom and human volunteer using the OS-EM algorithm.
**Results:** It has been found that the bi-exponential scatter function is independent of material and depth of source. It is necessary to use an iterative process for correction of the transmission map in practice. The quantitative errors observed in the emission images reconstructed with the corrected transmission map were +7% and -8% in the lung and liver respectively compare to -35% and -54% reconstructed with an uncorrected transmission map. Similarly in the clinical study corrected transmission data improved the contrast and visualisation.
**Conclusion:** Transmission based correction method can be effectively applied to correct the attenuation map deployed for attenuation correction for emission images of $^{67}$Ga SPECT.

**P-106**
**Incidence of micronuclei in thyroid cancer patients treated with therapeutic dose of radioiodine (I-131)**
GS Pant, Omana Nair*, S Senthamizhchelvan, CS Bal, PK Julka*, GK Rath*. *Departments of Nuclear Medicine and *Radiotherapy, AIIMS, New Delhi, India*

**Introduction:** Ablation of any thyroid remnants with Iodine-131 is the mainstay of the post-surgical management of patients with differentiated thyroid cancer. The radiation induced chromosomal abnormalities can reliably be assessed by evaluating the frequency of micronuclei (MN). Therefore, the yield of micronuclei in peripheral blood lymphocytes can be considered as a "biological dosimeter". The micronuclei (MN) assay in peripheral blood lymphocytes was used to assess the genotoxic potential of a single dose of $^{131}$I given to 50 patients for ablation of thyroid remnants after total thyroidectomy.
**Materials and Methods:** Peripheral blood samples were collected before and after $^{131}$I therapy and evaluated for the incidence of MN. Cultures were set up in duplicate for each blood sample. Whole blood (0.5 ml) was cultured in a complete media, consisting of 4.5 ml RPMI-1640 supplemented with 0.5 ml fetal bovine serum and 1.5% phytohemoagglutinin (PHA). After 44 h of incubation, cytochalasin B (Cyt-B) was added at a final concentration of 6 μg/ml. Cells were harvested at 72 h and slides were prepared according to standard procedures. A minimum of 1000 binucleated cells was scored for the presence of micronuclei per sample.
**Results:** A significantly increased MN frequency was observed in lymphocytes of patients two days after $^{131}$I therapy, when it was almost two fold the spontaneous frequency observed in the pre-therapy samples. MN frequency slowly declined thereafter, reaching the baseline levels after about 6 months when the patient came for follow up. A relationship was found between the MN frequency observed in lymphocytes of patients and activity of $^{131}$I administered for therapy.
**Conclusion:** Increased incidence of MN frequency by radioiodine therapy has previously been reported for differentiated thyroid cancer (DTC) patients. Our results also showed increased incidence of MN in these patients. We also observed that when these patients came for follow up after 6 months, the MN frequency in their blood lymphocytes comes down to the spontaneous incidence level. Complete disappearance of MN frequency after 6 months suggests that all the damaged cells have selectively been removed from the blood pool.
**Acknowledgement:** Indian council of medical research (ICMR) partially supported this work through its research contract No.5/13/16/2001-NCD-III.

**P-107**
**Use of non-isocentric CARM for intracavitary brachytherapy treatment: An experience of N.R.S. Medical College and Hospitals, Kolkata**
Jayanta Kumar Pal, Amitava Roy. *N.R.S. Medical College and Hospitals, Kolkata, India*

**Introduction:** In India the most common found cancer among woman is the Cancer Cervix. Conventional External Beam can not along provide the required dose to the site due to the dose limiting structures of bladder and rectum. To escalate dose local radiation is given by brachytherapy. Individual plan by the TPS can be done very easily when images are taken in an non-isocentric CARM and the images are transferred to TPS in the DICOM format. Locally made CARM s are non isocentric, but isocentric mode of TPS can be used by giving rotational and translational motion of the CARM with correct value.
**Materials and Methods:** AP and Lateral images of the pelvis of the inserted patient are taken by performing rotational and translational movement of the CARM. Images are then fed to PLATO Sunrise TPS system. Magnification and source to axis distance are given properly and the catheters are reconstructed on TPS. Reconstructed catheters are checked. Treatment calculation is performed and Treatment Control Unit executes plans.
**Result and Conclusion:** Catheter reconstructed in this way is very fast, the whole planning procedure can be completed very fast. Hence each day 3 to 4 patient plans can be performed very easily, so that patients may receive the correct dose every time.

**P-108**
**Calibration of well type ionization chambers used for brachy therapy sources**
AK Mahant, SS Tambe, Sudhir Kumar. *Radiation Safety Systems Division, Bhabha Atomic Research Centre, Mumbai - 400085, India*

**Introduction:** Well type ionization chamber provides a convenient means for the independent verification of brachytherapy high dose rate (HDR) sources in view of its sufficiently large signal and reproducible measurement geometry, though it has to be first calibrated in terms of reference air kerma rate (RAKR) by free in air measurements with the help of reference standard. SSDL, BARC recently has carried out on-site calibration of well type chambers for more than twenty hospitals using a 1000 cc graphite ion chamber specially designed for this purpose. The paper gives the details of calibration procedure and the results of the measurements.
**Materials and Methods:** A 1000 cc graphite ion chamber has been designed and fabricated for the measurement of Reference Air Kerma Rate (RAKR) of brachytherapy LDR and HDR $^{192}$Ir sources used in radiotherapy centres in India. RAKR value thus obtained is used to calibrate the Well type chambers of the hospitals. The ion chamber is cylindrical in shape and is made of high-density, high purity graphite. The chamber sensitivity has been computed theoretically for $^{192}$Ir ($\bar{E}$ =397 keV) and $^{137}$Cs (662 keV) the two commonly used brachytherapy sources in India. Sensitivity calculations are based on Burlin's general cavity theory[1] in view of the large size of the chamber cavity. The ion chamber is being used in conjunction with an electrometer NE 2670 (U.K.) The experimental set up for the measurement of RAKR consists of a jig for mounting the ion chamber and the source at three distances viz. 50 cm, 75 cm and 100 cm. The use of jig ensures the precise positioning of the chamber and the source. The measurements at three distances are used to set up three equations of which any two equations are solved to obtain the room scatter. The mean scatter value obtained

[Downloaded free from http://www.jmp.org.in on Friday, July 29, 2011, IP: 130.14.254.25] || Click here to download free Android application for t

**Table 1: Results of $^{192}$Ir HDR source measurements with the 1000 cc graphite chamber**

| S. No. | Hospital | Source | $RAKR_{Measured}$ $cGy.m^2h^{-1}$ | $RAKR_{Vendor}$ $cGy.m^2h^{-1}$ | % Deviation |
|---|---|---|---|---|---|
| 1. | H1 | Nucletron MS | 2.1590 | 2.187 | -1.28 |
| 2. | H2 | Nucletron MS | 1.0946 | 1.096 | -0.13 |
| 3. | H3 | Nucletron MS | 0.1265 | 0.1311 | -3.51 |
| 4. | H4 | Nucletron MS | 3.441 | 3.432 | +0.26 |
| 5. | H5 | Alpha-Omega | 3.3377 | 3.3675 | -0.88 |

**Table 2: Calibration factors of well type chambers**

| S. No. | Hospital | Source | Well Chamber Type/Electrometer | $(N_{RAKR})^{192}Ir$ $Gy.m^2h^{-1}A^{-1}$ |
|---|---|---|---|---|
| 1. | H1 | Nucletron MS | Nucletron SDS/PTW Unidos | 9.519 x10$^5$ |
| 2 | H2 | Nucletron MS | SI HDR-1000/ Scanditronics wellhoffer | 4.570 x 10$^5$ |
| 3 | H3 | Nucletron MS | Nucletron SDS/ PTW Unidos | 9.0239 x 10$^5$ |
| 4 | H4 | Nucletron MS | Nucletron SDS/ PTW Unidos | 9.559 x 10$^5$ |
| 5 | H5 | Alpha-Omega | SI HDR-1000/ CDX-200A | 4.686 x 10$^5$ |

by solving the three sets of equations is subtracted from the chamber output for three distances. The corrected chamber outputs are used to compute the RAKR from which, the average RAKR value is calculated. The uncertainty in RAKR has been estimated to be ± 3% at 1σ level for LDR sources and 2% at 1σ level for $^{192}$Ir HDR sources. Calibration of Well type chambers was done at the position of maximum sensitivity as determined by positioning the source at various distances from the chamber bottom in steps of 2.5 mm. Table 1 gives the results of some recent measurements and for comparison purpose the value of RAKR quoted by the source manufacturer/vendor has also been given. Table 2 shows the calibration factors of the Well type chambers determined with the help of the 1000 cc graphite chamber.

**Reference**

1. Attix FH. Introduction to radiological physics and radiation dosimetry. Wiley-VCH: p. 248.

**P-109**

**A study on dose reduction methods during image guidance with KV-CBCT in radiation therapy**

Paul Ravindran, Mohammad Islam*, David Jaffray*. *Christian Medical College, Vellore, India, *Princess Margaret Hospital, Toronto, ON, Canada*

**Introduction:** Cone beam CT using megavoltage and kilo Voltage beams are increasingly being used for image guidance in Radiotherapy (IGRT). Of this kilo Voltage Cone beam CT is preferred because of better image quality and relatively less dose to patient during imaging. Though the patient dose during the kV CBCT could be small compared to the patient therapy dose, repeated use of this for daily treatment verification could deliver considerable dose to the normal tissues. In this study we have measured the dose during a kV CBCT using phantoms at different depths, to eye and contralateral breast and also for various image acquisition methods to arrive at methods of dose reduction during kV CBCT.

**Materials and Methods:** A Bench top kV CB CT setup that can simulate the geometries of kV CBCT attachment of a Linear accelerator has been used in this study. The doses were measured with cylindrical water phantom of diameters 16 and 30 cms and on humanoid phantoms. A set of high sensitivity MOSFET, with high bias, was utilized to make the dose measurements. The MOSFET calibration factors, in terms of cGy/mV, were determined by measuring the response at a depth of 2.0 cm in water and comparing the corresponding dose measured by an ionization chamber Dose to eye was measured using a head phantom for 360-degree full rotation, 270-degree and 195-degree (half rotation plus fan angle) arc scans. The eye dose was also measured for 195-degree scan simulating X-ray tube rotation anterior as well as posterior to the head. Dose to contra-lateral breast was also evaluated with a Rando phantom. The dose measurements were performed for 120 kV beams with mAs values of 0.5, 1, 2 and 3.2 per projection. The images of the head and breast phantoms obtained during these measurements were analyzed for image quality.

**Results:** Excellent image quality was obtained with 0.5 mAs/ projection and 320 projections over a complete rotation scan, however, acceptable image quality also resulted with 195 degree arc scan data for a 75% reduction in eye dose. The image quality was excellent for tube current of 0.5 mAs/ projection and as the current was increased the detector saturated resulting in reduced contrast. The dose measured on the surface of the eye was less by 50% for 270-degree scan and by 75% for 195- degree posterior scan compared to 360 scan. Performing 195-degree scan with tube rotation posterior to the head had significant effect on dose compared to a 195-degree scan with tube rotation anterior to the head. The dose to the eye and the image quality are compared for 195-degree posterior and anterior scans. The dose to contra-lateral breast was less by 30% and 40% for 270 and 195-degree scans. The image quality was analyzed by reducing the number of projection for 360-degree, 270-degree and 195-degree scans though these are expected to reduce the dose to critical organs, the reconstructed images had streak artifacts

**Conclusion:** Reducing the scanning arc resulted in significant reduction in dose without much loss of image quality. Increasing the mAs resulted in increased dose to critical organs and reduction in image contrast. Performing a 195-degree posterior arc scan reduced the dose to eye considerably compared to an anterior arc scan without any change in the image quality. Thus this work demonstrates that there are opportunities to minimize dose to critical organs without compromising the quality of image guidance.

This PDF is available for free download from a site hosted by Medknow Publications (www.medknow.com).

**P-110**

**Comparision of rectal dosimetry with 0.1CC ionization chamber measured and TPS calculated for HDR intracavitary applications in carcinoma of cervix**

C Ramakrishna Rao, P Krishna Reddy, Rajib L Sha, B Mishra, BN Rao, SD Sharma*. *Department of Radiation Physics, MNJ Institute of Oncology and RCC, Red Hills, Hyderabad, *RP. and A.D, BARC, Mumbai, India.* E-mail: rajibsha@gmail.com

**Introduction:** Intracavitary Brachytherapy (ICBT) in the management of carcinoma of cervix is an established technique which is gaining popularity. Rectum is one of the most important critical organ in the treatment of carcinoma of cervix. Conventionally, dosimetry of Intracavitary application is carried out using variable/orthogonal angled radiographs where point doses to critical structures such as rectum and bladder were calculated according to the specification of ICRU 38. In this study, 20 HDR intracavitary applications were analysed and the dose received by the rectum from 0.1 cc (1 cm active length) Ionisation Chamber were compared with the ICRU 38 point doses.

**Materials and Methods:** 20 Patients with cancer cervix who were treated with radical radiotherapy and undergoing HDR Brachytherapy were selected for this comparative study. Radio-opaque gauze packing was used in all patients (i) to maintain fix geometry of applicator, (ii) to push bladder and rectum away from the applicator position. A thimble chamber (0.1 cc) of type 23323 which is air - and watertight and flexible enough was inserted in the rectum for measuring rectal dose and subsequently used as reference for marking rectal point. Three lead rings were wound on the active region of the detector which are equally distant to verify the correct positions by the help of simulator

[Downloaded free from http://www.jmp.org.in on Friday, July 29, 2011, IP: 130.14.254.25] || Click here to download free Android application for t in fluoroscopic mode. A contrast (7 cc) filled foley catheter ballon was used to visualize the bladder. All patients were simulated and variable angled radiographs obtained with radio-opaque dummies in the applicator. The images were transferred via DICOM SC to the TPS. Treatment planning carried out on 3D PLATO SUNRISE Brachytherapy V.14.3 (Nucletron, The Netherlands). A dose of 7/9Gy per fraction was given at Manchester pt 'A'. Though smaller than 2-3 cc (3-4% of the min volume of the rectum) could be clinically not significant according to ICRU38, still we measured and compared 0.1cc volume of rectal dose value with average rectal dose values calculated by TPS taking three reference points in rectum. After simulation and planning each patient was taken to HDR treatment room, detector was connected with PTW UNIDOS SSD and treatment delivered using Microselectron HDR (Nucletron). Finally the 0.1 cc volume (1 cm active length) rectal dosimetry was calculated by using the following formula.

Dose 'D' (Gy) = MR (nc) x Attenuation factor x Ktp x NDw (Co60) (Gy/c) x KQ,Q0.

Where attenuation Factor corrected to lead rings and Ktp corrected to temp at rectum and room pressure and KQ,Q0 = 0.97634 was obtained by interpolating the values from the energy response curve given in the manufacture's instruction manual.

**Results and Discussion:** The average rectum dose for the 20 patients ranges from 32.11 to 69.85% obtained from TPS and 28.98 to 64.4% calculated from 0.1 cc rectal chamber. The variation between average rectal dose obtained from TPS and from the measured values by 0.1cc rectal Ion chamber was found to be ± 2% in 2 patients, ± 5% in 10 patients, ± 10% in 4 patients and ± 20% in 4 patients.

### P-111

### Study of different methods for the verification of point co-ordinates in HDR brachytherapy

P Krishna Reddy, C Ramakrishna Rao, B Mishra, RL Sha, BN Rao. *Department of Radiation Physics, MNJ Institute of Oncology and RCC, Hydrebad, India.* E-mail: m.bibek@gmail.com

**Introduction:** In the present scenario of cancer treatment Brachytherapy plays an important role. The accuracy of the treatment in delivering the prescribed dose as executed through treatment planning system needs verification through a simulated point co-ordinate system. In the user facility sometime all the resources are not available. In this paper less expensive materials were taken to study the identification of the point co-ordinates. By the use of wax phantom with grids, slab (paper) phantom with dummy X-ray markers and a ping pong spherical ball with three lead shots of one mm dia, as a part of quality assurance for TPS, one can find its suitability for the verification and its reproducibility. By the use of a paper slab phantom the irregular nature of the catheters with the dummy sources was studied by taking transverse slices in the CT and reproducing it in the graph sheet. Similarly by the use of wax phantom, keeping a grid in a reference plane with a defined axis in that plane and source co-ordinate in the virtual applicators, which can be constructed like Fletcher suit, Henscke, Vaginal cylinder and intra luminal applicators, the point co-ordinates can be well verified. Transferring the co-ordinates form one frame of reference to the other and extrapolating to different angles when there is some problem in identifying the catheters, the projected co-ordinates was obtained.

**Materials and Methods:** A wax phantom was designed with 15x15x15 cm with 3 slabs incorporating a copper wire grid system with a minimum grid size of 1sq.cm in one plane and a co-ordinate axis with a 3D Cartesian coordinate system. The normal to the plane and the Z-axis of the Cartesian system (reference axis) was parallel. A virtual 60 degree fletcher suit applicator was constructed by taking aluminum pins. A lead ball with 1 mm diameter was placed at a different plane. All the coordinates were generated by spherical coordinate system for the particular gantry angles, again converted into Cartesian system. Now from TPS the values were generated taking the reference axis. All the manual measurements are done with a vernier scale. Similarly a phantom is designed with paper slabs (30x20x10 $cm^3$) and with the help of dummy sources, known irregular shape geometry was drawn in different planes by placing the graph sheets. The catheter reconstruction was carried out by taking 3 mm CT cuts. The coordinates of the points were taken from the TPS and that values were plotted in the graph sheet. Then the results were compared with the manual values. By the use of a ping pong ball and three lead balls having diameter 1 mm two images (Ap and Lateral) were taken. In the 2D imaging, this arrangement reflecting the bladders points. Verification of the points was done taking relative sift of the origins.

**Results and Discussion:** From the above study the variation between TPS generated values and manually calculated values were found to be within 3% or 1 mm which is the recommended tolerance limit as per the BARC protocol.

### P-112

### Study of performance of semi-automatic RIA counter

BL Acharya, NC Sharma*. *Department of Radiotherapy and *Radiodiagnosis, RNT Medical College and Associated Group of Hospitals, Udaipur - 313 001, India*

The PC RIA semi-automatic RIA counter consisting of Gamma counter, PC and matrix printer made of Stratec Biomedical System (Germany) was installed in the Institution in March, 2005. Earlier we had single well RIA Counter made of ECIL, Hyderabad. Due to increase of samples and tests, it has been decided to purchase the new counters. The new counter consisting of 6 highly sensitive detectors and also counter can be upgraded for 12 detectors. Each detector is connected to 256-channel spectrometer. The presentation of the spectrum on the screen allows us to control the performance of the system. The various performances test e.g., energy calibration, resolution, sensitivity efficiency, counting precision and linearity of energy and activity will be discussed. The IAEA and Company protocols have been used in order to evaluate the performance parameters. Some typical RIA results will be presented.

### P-113

### Comparative analysis of effective transit time of remote afterloading HDR brachy therapy units

Hema Vaithianathan*, GC Srinidhi, SS Supe*, H Sudahar, B Ramya, JGR Solomon, MS Vidya Sagar. **Department of Radiation Physics, Kidwai Memorial Institute of Oncology, Bangalore, Department of Radiotherapy and Oncology, Kasturba Medical College, Manipal, India*

**Introduction:** In HDR Brachytherapy, the dose delivered to the tumor is very high in short duration. Hence stringent quality assurance should be done at frequent intervals. The stepping single source is made to dwell in different dwell positions for the desired dwell times to deliver the optimum dose to dose reference points. During the time of movement of the source from one dwell position to the other, the source in transit contributes dose to reference points. This dose is termed as transit dose and is significant when the source activity is high, dwell positions are large in number and the dwell times are lesser. Hence it is important to determine the Effective Transit Time for HDR brachytherapy source as a part of Quality Assurance Programme. A study to compare Effective Transit Time of the High intensity Ir-192 sources of two HDR units has been done and results are presented hereunder.

**Materials and Methods:** In this study, a CD well type ionization chamber and a CD electrometer are used for Gammamed Plus HDR brachytherapy unit and a Nucletron Source Dosimetry System and a PTW Unidos electrometer of a MicroSelectron HDR unit are used to measure the Effective Transit Time of the Ir-192 sources. The net charge generated during the source transit from one dwell position (say X 1 to X 2) to the other dwell position and for the source dwelling at X1 in a Well Type ionization Chamber was measured for the inter dwell distances of 1 cm, 5 cm and 10 cm. A linear regression analysis of the net charge measured as a function of dwell time was made. The combined effects of charge generated during the source transit between two dwell positions, any reduction in the set stationary dwell times so as to account for transit time and dwell time error that may exist in the

This PDF is available for free download from a site hosted by Medknow Publications (www.medknow.com)

[Downloaded free from http://www.jmp.org.in on Friday, July 29, 2011, IP: 130.14.254.25] || Click here to download free Android application for t unit can be quantified from effective transit time measurements.

**Results:** Effective Transit Times computed were found to vary from 0.09541 sec to 0.7496 sec for an interdwell distance of 10 cm for GammamedP*lus* HDR unit. The speed of the Ir-192 source of GammaMedP*lus* HDR unit as a function of interdwell distance was studied and found to be 22.971 cm/sec, 35.5 cm/sec and 26.987 cm/sec for 1, 5 and 10 cm respectively. Effective transit times were found to vary from 0.03812 sec for interdwell distance of 1cm to 0.44256 sec for 10 cm. for Micro Selectron HDR unit. The speed of the source as a function of interdwell distance found to be 52.466 cm/sec, 36.6 cm/sec and 45.16 cm/sec for interdwell distances of 1, 5 and 10 cm respectively.

**Conclusions:** Since the Effective Transit Times for small interdwell distances are much less compared to the timer resolution of the unit, the error in the measurement can be large. This procedure includes dwell time error and can be considered as an Effective Transit Time. The values of effective transit time of a HDR Brachytherapy stepping source are essential to compute the transit dose and assess its significance in treatment planning.

**P-114**

**Comparison of the transmission characteristics of nucletron high dose rate brachytherapy applicators**

H Sudahar, GC Srinidhi, BR Amya, JGR Solomon, MS Vidyasagar. *Department of Radiotherapy and Oncology, Shridi Sai Baba Cancer Hospital and Research Centre, Kasturba Medical College, Manipal - 576 104, India*

**Introduction:** High dose rate (HDR) brachytherapy is one of the major treatment modalities in the field of radiotherapy. HDR brachytherapy using Nucletron HDR unit involves applicators of different materials and dimensions. Also, the Ir192 HDR source is calibrated using an insert when the well type chamber is used for calibration. Baltas *et al*,[1] suggest that the applicator material can influence the source calibration. Uniformity of applicator transmission is an important criterion for using the same source strength when different applicators are used. Aim and objective: To compare the transmission characteristics (relative attenuation and source strength with different applicators) of high dose rate brachytherapy applicators.

**Materials and Methods:** In this work the transmission characteristics of different Nucletron HDR applicators (6 Nos) were estimated in terms of relative transmission factors with respect to the calibration insert. For maintaining the geometry, an additional insert was designed and used. Attenuation of this insert is also taken into account. PTW SDS well type chamber with UNIDOS electrometer was used. The maximum response position in the well type chamber with each of the applicator was found out. The readings taken at the maximum response position are used for comparison of relative transmission factor and air kerma strength.

**Results and discussion:** The relative transmission factors of the applicators with respect to the calibration insert are ranges between 0.9898 and 1.0124. The measured value of air kerma strength of the Ir192 Source with different applicators is ranges between 3.020 cGy.m2.h-1 and 3.089 cGy.m2.h-1. Even though the percentage difference with the quoted Air Kerma strength (3.079 cGy.m2.h-1) falls with in the recommended limit[2] of 3%, these factors are not unique.

**Conclusion:** From the observations it is found that the applicator transmission characteristics are not unique, hence for maintaining the uniqueness it is essential to correct the application transmission factor / air kerma strength for dose calculation in HDR brachytherapy.

**References**

1. Baltas D, Geramani K, Ioannidis GT, Hierholz K, Rogge B, Kolotas C, *et al*. Comparison of calibration procedures for 192Ir high-dose-rate brachytherapy sources. Int J Radiat Oncol Biol Phys 1999;43:653-61.
2. Acceptance/ Quality assurance tests of remote after loading brachytherapy unit. BARC: 2001.
3. Goetsch SJ, Attix FH, DeWerd LA, Thomadsen BR. A new re-entrant ionization chamber for the calibration of iridium-192 high dose rate sources. J Radiat Oncol Biol Phys 1992;24:167-70.
4. Nucletron's selectron dosimetry system user manual.

**P-115**

**Quality assurance of brachytherapy: A prospective evaluation**

MA Bilal Hussain, VA Mohd Ansari. *Dept. of Radiotherapy, JN Medical College, AMU, Aligarh, India*

**Introduction:** Quality Assurance is an integral part of pre-commissioning procedures of any equipment. In Brachytherapy the various QA procedures are adopted to find out the acceptance and suitability of the equipment for patient treatment. The QA program is carried out as per BARC/AERB guidelines and description is given in this paper.

**Materials and Methods:** A Micro-Selectron High Dose Rate Brachytherapy equipment supplied by Nucletron was recently installed at our centre. The machine was loaded with a 10Ci Ir-192 radioactive source. QA tests were carried out as per BARC/AERB proforma RP and AD/Remote QA/01. The Plato Brachytherapy Planning system is supplied along with and forms the integral part of the whole setup. The QA tests have to be carried out separately for the HDR equipment and the Planning System. The QA tests for the HDR equipment are broadly classified into Mechnical, Electrical and Radiation Checks. The Mechanical checks of the equipment like functioning of the sensors, couplings are tested and found to be satisfactory. The time taken to drive the source to 'ON' and 'OFF' position are measured using the independent timer and cross matched with the treatment control station display. The electrical checks included the checking of interlocks like treatment door, applicator-guide tube, guide tube-treatment unit. The functioning of emergency stop button, the displays in the treatment console and on the treatment unit and all the Treatment Control Station functions are checked and found to be working satisfactorily. The Leakage and contamination checks were performed by programming the source in to the flexible implant tube and the same is cut and measured using the contamination monitor Nucleonix CM 710E. The auto-radiograph of the source is taken after programming the source for various positions in the auto-radiographic check device. The positioning accuracy and uniformity are obtained from the auto-radiograph. The Timer error, timer linearity and end error are calculated by using the set procedures and the NE farmer dosimeter with 0.6cc chamber is used. For timer error the source is programmed first for 't' secs and then for '2 x (t/2)' secs. In order to find the timer linearity and end error the source is programmed for 60, 120, 180, 240 and 300 secs and the respective metre readings are obtained and a graph is plotted. A computer program is developed as a part of the already existing in-house planning program for finding the timer error, timer linearity, end error and also for calculating the source strength. The source strength measurement is the critical part of the QA program. As we do not have well type ion chamber, we have used the 0.6cc SSD chamber with the source calibration Jig supplied by Nucletron. The source strength measurement is performed at 10cm distance. Before doing the actual measurement the sensitive source position is found out by programming the source for various dwell positions. The source strength was measured and the deviation with the source certificate was found to be 1.69%, well within the acceptable limit. The final part of the QA of HDR involves the Radiation safety features measurements like Gamma zone monitor, unit leakage and Radiation survey. The survey of the installation was carried out using the survey meter Digirad WB88. In the second part, the QA of the planning system is carried out. The input device accuracy, calculation algorithm accuracy for single and multiple source dwell positions, decay correction and the output device accuracy are found to be satisfactory.

**Conclusion:** QA and pre-commissioning checks are all carried out as per BARC/AERB guidelines and found to be very well within limits. The pre-commissioning checks are not the end by itself. A regular QA programme is designed to carry out the QA checks periodically and after every source change.

This PDF is available for free download from a site hosted by Medknow Publications (www.medknow.com).

[Downloaded free from http://www.jmp.org.in on Friday, July 29, 2011, IP: 130.14.254.25] || Click here to download free Android application for t

### P-116

### Generation of LQ-bed lookup table for radiobiological calculation

MA Bilal Hussain, VA Mohd Ansari. *Dept. of Radiotherapy, J.N.Medical College, A.M.U, Aligarh, India*

**Introduction:** Various Radiobiological Models have been developed to help the therapists to equate and find the new dose equivalent when changing from one regime to other. NSD,TDF, CRE and LQ are among the few. Though Linear Quadratic Model was most promising but it still has many uncertainties and tissue specific parameters which are very less known. To help design day-to-day fractionation schemes, tables of TDF and CRE were made available. The use of tissue specific parameters in the LQ Model restricts such a table for the BED values. In this paper BED tables for both teletherapy and HDR brachytherapy for the daily calculations are described.

Materials and Methods:

The LQ Model BED equation for teletherapy is

$$BED = nd\left(1 + \frac{d}{(\alpha/\beta)}\right) - k\,(T - T_k)$$

This contains tissue specific parameters $(\alpha/\beta)$, k and $T_k$ which need to be ascertained to each type of tissue. For practical calculations $\alpha/\beta$ is assumed a value of 10 for tumour and early responding tissues, k for rapidly proliferating tumour is 0.6 Gy/day, 0.1 Gy/day for slowly proliferating tumour and 0 for late responding tissues. For teletherapy, like TDF table, using above values, a bed table is generated for various dose/FR and for various no. of fractions. The table gives the bed values for the tumour by assuming the parameters of tumour. The fractions ranges from 2 to 40 and the dose/fr ranges from 150 cgy to 1000 cgy. From this lookup table, it becomes easy to find the equivalent dose/fr for variable no. of fractions. A separate table is also made available for finding the factor k $(T-T_k)$ separately. This gives the factors for the values of T and $T_k$. This table along with the BED table helps us in finding out the compensation dose for gap correction. A sample lookup table is given here in Table 1. A software program is developed to generate the tables for various values of the tissue specific parameters, which helps us in finding the tolerance dose for the critical organs as well. The tissue specific parameters were collected from various literatures and researchers. Like Teletherapy, yet another routine problem is finding the equivalent HDR Brachytherapy dose various fractionation schemes. A lookup table for the HDR fractionation is generated using the equation:

$$BED = n\,d\left(1 + \frac{2R}{\mu(\alpha/\beta)\,\mu T}\left\{1 - \frac{1}{\quad}[1 - \exp(-\mu T)]\right\}\right)$$

The $\mu$ values for Tumour and early responding tissues are assumed 1.4 and for late responding tissues 0.46. The table or a graph (sample Chart-1) of BED values can be generated for any value of tissue specific parameters using the software mentioned above. Various tissue specific parameters are already available in the software and user has the provision to input his/her own parameters.

**Conclusion:** These BED lookup tables ease the workload for routine equivalent dose, gap correction calculations and finding the equivalent HDR fractionation doses.

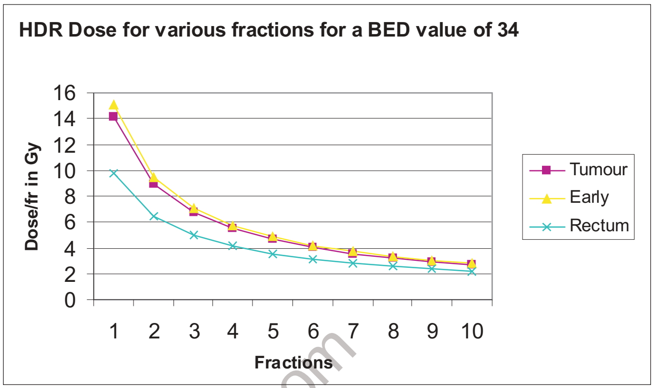

**Figure 1:** Sample chart for 3000cGy LDR equivalent HDR

### P-117

### Expert systems and radiological, clinical and imaging equipment management

SS Arora, PS Saini*. *Departments of Biophysics and *Hospital Engineering, Postgraduate Institute of Medical Education and Research, Chandigarh, India*

**Introduction:** The present healthcare delivery system exhibits a very complex scenario where facilities, materials, equipment and full range of human involvement are in continuous interaction. Various medical, scientific and technical innovations are being deployed for the benefit of human beings. Large hospitals and medical institutes are increasing their financial resources in that direction and allocating special budgets for the procurement of high technology patient care equipment- be it diagnostic, therapeutic or rehabilitative. The correct management of the various technological resources in health care institutions in terms of efficiency, effectiveness and safety is of utmost importance in view of the criteria on which modern medical care is based. Almost all patient care activities carried out in a modern hospital are based on widespread use of equipment and technical procedures, the efficiency and precision of which can directly influence the quality of service offered to the

This PDF is available for free download from a site hosted by Medknow Publications (www.medknow.com).

**Table 1: Teletherapy BED values for 5 Fr/wk: $(\alpha/\beta)=10$**

| *Dose/Fr.* | *Fractions* | | | | | | | | | | | | | | |
|---|---|---|---|---|---|---|---|---|---|---|---|---|---|---|---|
| cGy | 4 | 5 | 10 | 11 | 12 | 13 | 14 | 15 | 16 | 17 | 18 | 19 | 20 | 25 | 30 |
| 150 | 6.9 | 8.6 | 17 | 19 | 21 | 22 | 24 | 25.9 | 27.6 | 29.3 | 31.1 | 32.8 | 34.5 | 40.1 | 44.6 |
| 160 | 7.4 | 9.3 | 19 | 20 | 22 | 24 | 26 | 27.8 | 29.7 | 31.6 | 33.4 | 35.3 | 37.1 | 43.4 | 48.5 |
| 170 | 8 | 9.9 | 20 | 22 | 24 | 26 | 28 | 29.8 | 31.8 | 33.8 | 35.8 | 37.8 | 39.8 | 46.7 | 52.5 |
| 180 | 8.5 | 10.6 | 21.2 | 23.4 | 25.5 | 27.6 | 29.7 | 31.9 | 34 | 36.1 | 38.2 | 40.4 | 42.5 | 50.1 | 56.5 |
| 190 | 9 | 11.3 | 22.6 | 24.9 | 27.1 | 29.4 | 31.7 | 33.9 | 36.2 | 38.4 | 40.7 | 43 | 45.2 | 53.5 | 60.6 |
| 200 | 9.6 | 12 | 24 | 26.4 | 28.8 | 31.2 | 33.6 | 36 | 38.4 | 40.8 | 43.2 | 45.6 | 48 | 57 | 64.8 |
| 210 | 10.2 | 12.7 | 25.4 | 28 | 30.5 | 33 | 35.6 | 38.1 | 40.7 | 43.2 | 45.7 | 48.3 | 50.8 | 60.5 | 69 |
| 220 | 10.7 | 13.4 | 26.8 | 29.5 | 32.2 | 34.9 | 37.6 | 40.3 | 42.9 | 45.6 | 48.3 | 51 | 53.7 | 64.1 | 73.3 |
| 230 | 11.3 | 14.1 | 28.3 | 31.1 | 33.9 | 36.8 | 39.6 | 42.4 | 45.3 | 48.1 | 50.9 | 53.8 | 56.6 | 67.7 | 77.7 |
| 240 | 11.9 | 14.9 | 29.8 | 32.7 | 35.7 | 38.7 | 41.7 | 44.6 | 47.6 | 50.6 | 53.6 | 56.5 | 59.5 | 71.4 | 82.1 |
| 250 | 12.5 | 15.6 | 31.3 | 34.4 | 37.5 | 40.6 | 43.8 | 46.9 | 50 | 53.1 | 56.3 | 59.4 | 62.5 | 75.1 | 86.6 |
| 300 | 15.6 | 19.5 | 39 | 42.9 | 46.8 | 50.7 | 54.6 | 58.5 | 62.4 | 66.3 | 70.2 | 74.1 | 78 | 94.5 | 109.8 |
| 350 | 18.9 | 23.6 | 47.3 | 52 | 56.7 | 61.4 | 66.2 | 70.9 | 75.6 | 80.3 | 85 | 90 | 94.5 | 115 | 135 |
| 400 | 22.4 | 28 | 56 | 61.6 | 67.2 | 72.8 | 78.4 | 84 | 89.6 | 95.2 | 101 | 106 | 112 | 137 | 161 |
| 450 | 26.1 | 32.6 | 65.3 | 71.8 | 78.3 | 84.8 | 91.4 | 97.9 | 104.4 | 110.9 | 117.5 | 124 | 131 | 160 | 189 |
| 500 | 30 | 37.5 | 75 | 82.5 | 90 | 97.5 | 105 | 113 | 120 | 128 | 135 | 143 | 150 | 185 | 218 |

[Downloaded free from http://www.jmp.org.in on Friday, July 29, 2011, IP: 130.14.254.25] || Click here to download free Android application for t

**Table 1: Status of in-house repair and maintenance of clinical, X-ray and medical equipments for the year 2005-06.**

| | | | |
|---|---|---|---|
| Total number of complaints | 1477 | At site | 563 |
| Number of Instruments repaired | 1204 | At site | 440 |
| Percentage of repaired Instruments | 81.51% | | |

| *S.No.* | *Classification of equipments* | *Number of complaints* | *Percentage* |
|---|---|---|---|
| 1. | Class I B | | |
| | i) Minor repair | 491 | 40.78 |
| | ii) Major repair | 93 | 7.72 |
| 2. | Class I BF | | |
| | i) Minor repair | 295 | 24.50 |
| | ii) Major repair | 66 | 5.48 |
| 3. | Class I CF | | |
| | i) Minor repair | 75 | 6.23 |
| | ii) Major repair | 14 | 1.16 |
| 4. | Class II B | | |
| | i) Minor repair | 76 | 6.31 |
| | ii) Major repair | 12 | 1.00 |
| 5. | Class II BF | | |
| | i) Minor repair | 49 | 4.07 |
| | ii) Major repair | 9 | 0.75 |
| 6. | Class II CF | | |
| | i) **Minor repair** | 24 | 2.00 |
| | ii) Major repair | Nil | 0 |

patient. In modern hospitals, radiological services and human organ imaging are the vital areas for providing non-invasive diagnostic tests for detecting various ailments and deep-seated diseases. The importance of these specialties has assumed greater importance primarily because of the multi pronged approach in imaging of various human organs with speed and better resolution. Various sophisticated equipments which are now-a-days in use are MRI Scanners, CAT Scanners, Ultrasound Doppler, Linear and Phased array Scanners, DSA imaging, dedicated mammography along with conventional radiographic and fluoroscopic machines.

**Expert Systems and Medical Equipment Repair:** Advances in the field of remote diagnostics and artificial intelligence in the field service industry by using "expert" oriented decision techniques has opened up new vistas of knowledge. These are based on either rule or "heuristic" expert models and processes. These knowledge based approaches can be used for equipment repair and management by using 'a-priori' and 'post-priori' decision trees. This process culminates into general fault models, trouble shooting models and deep causal models for preventive, predictive and emergency breakdown repair and maintenance of medical equipment.

**Preventive and Breakdown Repair:** Preventive strategies in maintenance of medical equipment are the most economic approaches. Hospitals need continuous presence of technical staff for repair and maintenance of medical equipment in various areas of the health facilities. It is extremely costly to pay a manufacturer/vendor to do the same. Apart from doing the repair jobs, the senior technical staff also looks after the pre-purchase evaluation and monitoring of service contracts. AT P.G.I.: The repair and maintenance of the Biomedical equipment is carried out by the In-house Biomedical engineering department [Table 1] but the very high technological equipment is also given on Annual Maintenance Contracts with the manufacturers/ suppliers. These contracts are monitored by the departmental staff in terms of cost and quality. The equipments which are repaired and maintained by the clinical engineers and technicians are Diathermy machines, Ventilators, Dialysis equipments, Incubators, Defibrillators, ECG machines, Ultrasound and X-ray machines, processors etc.

**Minor Repair:** It includes replacement of fuses, fixing of plugs and electrical mains cord, wires and providing accessories.

**Major Repair:** It includes the repair of any printed card board (PCB) by replacing electronic components or even the replacement of whole PCB.

**Conclusion:** Increasing use of sophisticated medical electronic equipments in hospitals necessitates a strong Biomedical engineering department in the Medical Institutions. Newer approaches like expert and intelligent systems in this area will add another arm in medical equipment management program and will reduce the mean time to repair (MTTR) and increase the mean time between failures (MTBF). This will improve quality of service that includes quick fault diagnoses and more robust and ingenious handling of the equipment faults. An In-house medical equipment management facility is a vital asset for any health care institution.

This PDF is available for free download from a site hosted by Medknow Publications (www.medknow.com)

### P-119

### Occupational over exposures from nuclear medicine practices

Manisha V Inamdar, B Nagalakshmi, SP Agarwal. *Radiological Safety Division, Atomic Energy Regulatory Board*

**Introduction:** Recorded Occupational over exposures after investigation by a committee constituted by Atomic Energy Regulatory Board (AERB) to confirm they are genuine are added to the respective personnel dose record. This paper details the actual instances of over exposures in nuclear medicine practice for past three years, avoidable and unavoidable work practices which led to over exposures.

**Methods and Results:** Personnel Monitoring and Dose Registry Section, BARC supplies personnel monitoring badges through accredited laboratories to all radiation workers in India. The Section maintains a national database of exposures received by all monitored radiation workers. The data comprises of doses received by the workers during

**Table 1: Nuclear medicine related over exposure cases reported during 2002-2006**

| *No.* | *Monitoring period* | *Brief description of event leading to overexposure* | *Dose received* | *Status of overexposure* |
|---|---|---|---|---|
| 1. | July-Sept 2002 | Work with I-131, solvent extraction, TLD left in hot lab | 19.10 mSv | |
| 2. | Oct-Dec 2002 | Dispensing small doses ofI-131 from bulk quantity | 11.6 mSv chest badge, 254.2 mSv wrist badge | Genuine |
| 3. | April-June 2003 | Dispensing and patient preparation with F-18 | 10.5 mSv | Genuine |
| 4. | April-June 2003 | Tc-99m aerosols used for ventilation studies, balloon connection carrying the radiopharmaceuticals got detached, releasing activity on person's apron and TLD | TLD showed gamma dose of 170 mSv | CA test dose 65 mSv as genuine |
| 5. | Jan-March 2004 | TLD left radiation area | Gamma dose 16.8 mSv | Non genuine |
| 6. | April-June 2004 | TLD left in hot lab | 13.2 mSv | Non genuine |
| 7. | April-June 2004 | Patient undergoing I-131 MIBG therapy coughed, in presence of nuclear medicine physician, leading to release of activity and exposure of NMP | Thyroid burden for NMP: 1.511 mGy, External gamma dose:33.15 mSv | Genuine Partly genuine, partly non genuine, since the person continued wearing the contaminated labcoat and TLD |
| 8. | Oct-Dec 2004 | TLD left in Linac room | Gamma dose 21.35 mSv | Non genuine |
| 9. | Jan-March 2006 | Apron with TLD left in hot lab area | 34.85 mSv | Non genuine |

[Downloaded free from http://www.jmp.org.in on Friday, July 29, 2011, IP: 130.14.254.25] || Click here to download free Android application for t a particular monitoring period, annual dose, any over exposure received during the monitoring period and life time dose. Periodicity of monitoring period varies with the use of radioisotopes and potential for exposures. In nuclear medicine practice radioisotopes are handled in unsealed form and in close proximity for diagnostic and therapeutic purposes which has a potential for both external and internal exposures. The practice has been on an increase in India since last decade with more centres being commissioned with the state of art imaging facilities. Tc-99m labeled radiopharmaceuticals obtained from in-house generator are the main work horse for diagnostic applications. Diagnostic use with recent introduction of the medical cyclotron produced Positron Emission Tomography (PET) radiopharmaceutical $^{18}$FDG is on the increase. I-131 in form of NaI solution or capsules is used for both diagnosis and treatment. The personnel monitoring period for nuclear medicine departments is quarterly and the effective dose recorded higher than 10 mSv in a monitoring period and higher than 20 mSv in a calendar year is considered as over exposure and investigated. There have been cases reported of skin exposures and extremity doses (both beta and gamma) close to annual limit, received in a monitoring period. Investigation reports are obtained from the exposed individual and the Radiological Safety Officer of the institution and further investigated by the AERB Committee. Doses are assigned to the individual on the basis of Committee's decision. The over exposures received by nuclear medicine workers, the cause and investigation status genuine/non genuine are listed in the following table.

**Conclusion:** Streamlined work culture and documented radiation protection program, employing qualified and trained staff, using self shielded gadgets, long tools, apportionment of stock radioactive materials and rotation of workers are the suggested measures for minimizing radiation exposures and avoiding over exposures. The authors are thankful to Dr. R.K. Kher, Head, Personnel Monitoring and Dose Registry Section, BARC for providing data on personnel over exposures in nuclear medicine practices.

### P-120

### Excel absolute dose (O/P) calculation process in clinics

V Choube*, AA Deshmukh*, AK Meshram*, B Sarkar*, HV Grare*, Laviraj S. Choudhari*. *Dept. of Medical Physics, **Radiation Oncology, RST, RCC, Nagpur, India*

**Introduction:** To measure the absolute dose in water, so called output of the radiotherapy machine, are mandatory physical dosimetry practice in clinics. The recommended setup and series of absolute reading at different voltages and field sizes, for specific energy beam, need to be generated. This raw data undergo different calculations to achieve the final goal "OUTPUT" at particular point. Here we have formulated and tested the TRS 398 recommended worksheet for absolute dose in water (Dw) in M S Excel software sheet. The aim was to maintain the constancy in calculation as well as to speed up the process without compromising accuracy.

**Materials and Methods:** In Ms Excel there are two options to create formulae (i) insert function (ii) formulae bar. Amongst these two options available for formulating the worksheet of calculation of Dw, formula bar option, have preferred. It is very easy and convenient for anybody those working on MsExcel. The steps are: (i) select cell,(ii) click formula bar, (iii) press "=", (iv) start writing formula with appropriate brackets for each mathematical operation. The quantities and formulae, for Dw Excel worksheet are mentioned below. The convention symbols for MsExecl are to be taken care e.g., sum +, divison /, multiplication * etc. Quantity Actual formula Excel sheet formula:

1. Kpol = -| m1 | + | m3 | / 2 * |m1 | (B6 + B14) / (2 * B6)
2. Ks = $a_0 + a_1 (m_1 / m_2) + a_2 (m_1 / m_2)^2$ = (B3+(B4*(B6/B7))+(B5*((B6/B7)*(B6/B7))
3. $K_{TP}$ = [ (273.2 + t) / (273.2 + t) ] x ($p_o$ / p) = ((273.+$B_9$) / (273.2 + $B_{10}$)) / ($B_{12}$ / $B_{11}$)
4. Corrected MR. = MR* $10^{-9}$ * $K_{TP}$ * $K_{POL}$ * $K_S$ * $K_{ELECT}$ = ($B_8$ * $B_{13}$ * $B_{15}$ * $B_{16}$)
5. O / P @ Dmax =(100*$N_{DW}$* $K_{QQO}$*Corrected MR)/PDD = ($B_{20}$*$B_{19}$*$B_{21}$) / ($B_{18}$) Gy / MU

(This is an example for MS Excel sheet. Suffices in XL formula are row no. of that cell and alphabet indicates the clumn no.) The equation 5 is the main formula to calculate Dw for manual and excel sheet method. One can arrange the formlae step by step,as per his convenience, keeping intact the mathematical analogy in original formulae. * Symboles: [ml-MR for +400V, m2- MR +l00V, m3- MR -400V, m- MR for user polarity (calibration polarity) +400V] MR is meter reading.

**Results and Conclusion:** This method of performing calculation is simple, fastest and accurate upto the desired digits. It is verified along with manual calculation method for several times. Manual calculation method takes 5 minutes for calculating output for each field size. While by excel worksheet, with only 7 prerequisite values instead of 20, takes less than 1 minute within which, entered reading and values can be crosschecked too, 5 times faster. This method is capable of calculating Dw instantly making us able to crosscheck the aspects on

| *Photons* | *6 MV* | *15 MV* | | | *Electrons* | *10 MeV* | | |
|---|---|---|---|---|---|---|---|---|
| As per TRS 398 | 10x10 | 10x10 | 5x5 | 30x30 | | 10x10 | 6x6 | 20x20 |
| a0 for | 1.022 | 1.022 | 1.022 | 1.022 | R50, ion | 3.996 | | |
| a1 m1/m2=4 | -0.363 | -0.363 | -0.363 | -0.363 | R50, Dose | 4.051884 | -0.06 | -0.06 |
| a2 | 0.341 | 0.341 | 0.341 | 0.341 | Z ref | 2.33113 | -0.136 | -0.136 |
| m1 +400 | 11.885 | 13.613 | | | Kelect | 1 | 1 | |
| m2 +100 | 11.82 | 13.595 | | | a0 | 1.198 | 1.198 | |
| ks (recombi) | 1.0018 | 1.0004 | #DIV/0! | #DIV/0! | a1 | -0.875 | -0.875 | |
| t | 20.5 | 20.5 | | | a2 | 0.677 | 0.677 | |
| t0 | 20 | 20 | 20 | 20 | M+ 300 | 1.90233 | | |
| p | 962 | 962 | | | M- 100 | 1.8895 | | |
| p0 | 1013 | 1013 | 1013 | 1013 | Ks (reco) | 1.003284 | #DIV/0! | #DIV/0! |
| Ktp (temp pressure) | 1.0548 | 1.0548 | #DIV/0! | #DIV/0! | M -300 | 1.91 | | |
| m3 -400 | 11.87 | 13.595 | | | Kpol | 1.002016 | #DIV/0! | #DIV/0! |
| Kpol | 0.999369 | 0.999339 | #DIV/0! | #DIV/0! | Hpl | 1 | 1 | |
| Kelect | 1 | 1 | 1 | | t | 20.5 | | |
| TPR 20, 10 | 0.6665 | 0.74868 | | | to | 22 | 22 | 22 |
| PDD at 10 cm | 66.97 | 75.76 | | | p | 968 | | |
| Kqo (B.C corre) | 0.9913 | 0.9775 | | | p0 | 1013 | 1013 | 1013 |
| Ndwqo co 60 | 53890000 | 53890000 | 5E+07 | 5E+07 | Ktp | 1.04117 | #DIV/0! | #DIV/0! |
| CorrectedMR | 1.26E-08 | 1.44E-08 | #DIV/0! | #DIV/0! | Kqqo | 0.91 | | |
| O/Pdmax | 0.010011 | 0.009982 | #DIV/0! | #DIV/0! | Ndwq0 | 5.52E+08 | 6E+08 | 6E+08 |
| | Gy/mu | Gy/mu | Gy/mu | Gy/mu | pdd z ref | 99 | | |
| Current O/P(OLD) | 0.010077 | 0.010091 | | | CorrecteMR | 1.99E-09 | | |
| Variation % | 0.6467 | 1.083 | | | O/P @ Zref | 0.010098 | #DIV/0! | #DIV/0! |

This PDF is available for free download from a site hosted by Medknow Publications (www.medknow.com).

[Downloaded free from http://www.jmp.org.in on Friday, July 29, 2011, IP: 130.14.254.25] || Click here to download free Android application for t spot, that too during setup condition. under process. On observing any variation in the result, we can decide, there itself for any changes required for individual or overall measuremlent setup. Which is not possible in case of using manual calculation method, where one have to repeat the complete setup again. This is most crucial aspect of the Dw Excel sheet. The observed variation in Dw by using both methods is maximum of 0.004%. Similarly output at isocenter for SAD setup can be determined by preparing another Dw worksheet with replacing appropriate formula for SAD technique. Because of faster access this Excel sheet can be effectively used for daily morning output check. The instant detail assessment record hardcopy of worksheet printout, is an added beauty of this Dw Excel sheet. Speacially, useful during Energy calibration.

**P-121**

**A Monte Carlo study of dose perturbations due to inhomogeneity in the medium**

Nirmal K Painuly*#, Anatoly Rozenfeld*, George Takacs*, AK Rath#, Suvendu Sahoo#, BK Mohanta#, H Mod#. **Centre for Medical Radiation Physics, University of Wollongong, New South Wales, Australia, #Hemalata Hospital and Research Centre, Bhubaneswar, India*

**Introduction:** The presence of inhomogeneity (air) in and around tumour is a major cause of concern in external beam radiation therapy. Measurements around air cavities embedded in tissue-equivalent material indicate that under dosing in and around an air cavity is significant. The effect is similar to skin sparing effect of high-energy photons and is of obvious clinical interest. The studies have shown a dose reduction at an air-tissue interface, which occur as a result of the lack of electronic equilibrium, both longitudinal and lateral. Accurate interface dosimetry at air/tissue/bone interfaces is vital for the success of the treatment using ionizing radiation, however, dose measurements at interfaces are generally difficult due to the high dose gradient, the electronic nonequilibrium and the detector finite size. The commonly used dose correction methods correct the dose for changes in photon fluence but do not account for changes in charged particle transport and therefore do not accurately predict the dose at interface. Errors up to 50% for electron beams and up to 30% for problems involving photon and electron transport near inhomogeneities have been reported. Monte Carlo provides a more realistic, physics-based approach. It explicitly transports photons and electrons within a material and therefore provides more accurate results at material interfaces and within inhomogeneities.

**Materials and Methods:** The Monte Carlo N-Particle (MCNP) Transport code version 4C was used for the current study. The purpose of the current study was to investigate the impact of the air cavity on dose profile as well as on depth dose for 6 MV X-ray photon beams. A 2 cm diameter air cylinder was selected to represent the air cavity in the head and neck cancer. Two sets of depth dose data and dose profiles were simulated, one each with air cavity and without air cavity. Simulation was also performed to study the variation of dose distribution along the rim of the air cylinder. The source-to surface distance (SSD) was 100 cm and field size was 5x5 $cm^2$ for each simulation. The dimension of the water phantom was 30x30x10 $cm^3$. The simulation was performed for the following setups:

(a) Dose profile along Y axis at 6 cm depth from the top surface of the phantom
(b) Dose profile along Y axis at 6 cm depth from the top surface of the phantom and below an air cylinder of diameter 2 cm and length 30 cm with it's axis parallel to Y axis.
(c) Depth dose in water along central beam
(d) Depth dose in water along central beam having an air cylinder. The rim of the cylinder (2 cm diameter) was positioned 4 cm beneath the surface with its axis parallel to the Y axis

**Conclusion:** It can be concluded that current study has established the utility of MCNP code as a very powerful and accurate tool for determining the dose at interface. It provides much more realistic estimates of interface dose, which can be exploited for the treatment of most head and neck cancers as well as for chest region which encompass heterogeneity in the beam path.

**P-122**

**A dosimetric study to evaluate additional rectal and/or sigmoid mucosal point doses during high dose rate intra cavitary brachytherapy planning**

Mishra Sanjib Kumar*, Mahantshetty Umesh, Tiwana Manpreet*, Jemima SV*, Engineer Reena, Deshpande Deepak*, Shrivastava Shyamkishore. *Departments of Radiation Oncology and *Medical Physics, Tata Memorial Hospital, Mumbai, India*

**Introduction:** Intracavitary brachytherapy plays an important role in the management of cervical cancer. The incidence of late rectal sequelae is dependent on doses to rectal point/s and incidence is higher when the doses delivered are more than 100Gy (BED). Despite rectal doses within tolerance limit, many patients develop grade III/IV rectal proctitis. Sigmoidoscopy in these patients frequently shows telangiectasia or ulceration of rectal / sigmoid mucosae, which are at higher level then the classical rectal points. In order to identify and document these high dose points in recto-sigmoid mucosae, we undertook this study.

**Methods and results:** Conventionally, dosimetry of intracavitary application is carried out using orthogonal radiographs where ICRU 38 rectal and bladder point doses are calculated. In order to identify additional rectal and sigmoid mucosal points, CT based planning of 22 high dose rate intracavitary (HDR-ICA) applications were evaluated. All these patients underwent standard HDR-ICA applications and later were taken for CT based planning. The planning was according to the ICRU 38 dosimetric planning standards. Additional rectal and sigmoid mucosal points were digitized till the axial CT cuts showed central tandem and doses to these points were compiled. The mean dose to ICRU rectal point was 66.2% of the prescribed dose. However, the doses to the additional rectal and sigmoid mucosal points ranged from 50% to 175% with a mean dose of 150% of the prescribed dose. There is a significant increase in doses to these points than the conventional ICRU rectal points. Further analysis, evaluation and discussion will be presented.

**Conclusions:** This dosimetric study suggests that the rectal and sigmoid mucosal points higher than the conventional ICRU rectal points get significantly higher doses. Identification and documentation of doses to these additional points on conventional orthogonal dosimetry needs to be defined. If these additional points are consistently getting higher doses then, it would be imperative to reduce the doses to these points and hence the late sequelae.

**P-123**

**Marching towards modern (Teletherapy 2006) technology**

G Reddy. *State of Art Technology used, Aiming at Curing Cancer and Controlling Benign Tumor.*

Preperation and execution of therapy constantly changed for achieving the precision. Quality Checks of treatment machines and supportive gadgets are crucial to assure precise radiation to be delivered. Appropriate Electron Beam is unique in achieving conformal therapy, Mycosis Fungoids affectively treated by 6 MEV electron beam delivered to skin all over the body, multiple critical structures could to be spared. Superficial tumours (surface to 4 cm deeply seated) receive required doseand spare undering risky tissue like lung while treating chest wall in case of operated breast CA. Stereotactic Techniques for treatments of benign (malignant occasional) lesions are affective in arresting growth of tumor. Late effect of endovascular occlusion by X- Knife Therapy for cerebral AVMs, acoustic neuroma and small post op residual meningeomas, 12 to 20 Gy prescribed. Critical tissues as lens of the eye, brain stem and optic chaisma etc receive minimal and safe doses. Intensty Modulated Radio Therapy revolutionized the technique to deliver precise radiotherapy to tumor. Surrounding criticals effectively spared. Allowed extra large tumor doses of radiation to head and neck tumors (PNS) and pelvic organ such as Prostate to escape dangers of radical surgery. Simple diagnostic X-ray of field is found useless. CT SCAN / SIMULATOR is essential to localise volume to be treated, verified

This PDF is available for free download from a site hosted by Medknow Publications (www.medknow.com)

[Downloaded free from http://www.jmp.org.in on Friday, July 29, 2011, IP: 130.14.254.25] || Click here to download free Android application for t and matched with preplans after making necessary adjustments. Guided RadioTherapy IGRT just started in India, it allows adjustment of the planned fields to match with movement such as lung lesion to fall exactly into field of radiation during delivery of fractionated course of RadioTherapy. Dose guided and volume guided techniques are under serious consideration to account for required tumor Doses prescribed and volumes defined and targeted. Conclusion: Conformal and Precision Techniques to be utilized for curative cancers; benign tumors producing symptoms and non tumourous lesions as Cerebral AVMs etc.

**P-124**

**Response of personnel monitoring dosimeters in different fields of high energy beta rays**

S Chatterjee, G Varadharajan, RN Pujari*, N Kalyanasundaram*, RK Kher. *Calibration and Dose Records Section, RPAD, *Radiation Safety and System Division BARC, Trombay, Mumbai - 400 085, India*

**Introduction:** Personnel monitoring of all radiation workers are carried out using TLD badges based on $CaSO_4$:Dy phosphor which is able to measure Gamma rays, X-rays, Beta rays as well as mixed fields of Gamma and Beta rays. In India, in CANDU type power reactors, beta dose is often encountered along with gamma rays. Personnels working in power reactors are supplied with a quartz fibre based DRDs for instantaneous dose measurements along with TLDs. Due to the presence of beta rays, the response of DRD and TLDs are different and hence leads to TLD-DRD discrepancy cases. This study was under taken to find the response of DRD and TLD to different dose levels of high energy beta rays in laboratory as well as in field conditions.

**Materials and Methods:** TLD badges used for this experiment are the same badges used for personnel monitoring in India described elsewhere (Vohra *et al*). Quartz fibre based Direct Reading Dosimeter is of Stephan make (U.K.) having range from 0 to 200 mR. Exposure of TLDs and DRDs to Beta rays were carried out using Beta Irradiation system. This Beta Irradiation system, also known as Beta Secondary Standard 2 (BSS 2) consists of three different beta sources, namely Sr/Y-90, Kr-85 and Pm-147. The calibration of the three beta sources has been done at PTB and is directly traceable to the primary standard for the dose of Beta radiation in tissue. The irradiation procedure is controlled by a personnel computer, which stores the calibration data for the sources to be used. The user only has to choose the measurement parameters, the irradiation conditions and the desired dose. BSS 2 can irradiate the dosimeters at a choice of fixed distances of 11 cm, 20 cm, 30 cm and 50 cm with dosimeter oriented at different angles. In our experiment, the exposure of the TLDs and the DRDs were carried out at the distance of 30 cm, normal incidence, over Perspex phantom having dimension of 30 x 30 x 5 $cm^3$. Readout of the TL dosimeters was carried out in Semiautomatic TL reader.

**Results and Discussion:** Table 1 shows the exposure of the TLD badge with DRD, both simultaneously exposed to different doses of Sr/Y-90 source. Each reading for DRD and TLD badge is the average of four DRDs and TLD badges. Kr-85 and Pm-147 was not used for the exposure of DRD because in DRD, ion chamber is inside a metal case, which is enough to cutoff low energy beta rays. It is seen that the dose estimation by DRD is roughly 5-6 times less than the actual dose delivered. TLD badge gives a better dose estimation of the beta dose and it can be further improved by using proper factor for dose evaluation. A similar exercise was also carried out in field conditions, where the energy and dose rate due to both beta and gamma were unknown. Two TLD badges and two DRDs were exposed at different positions of Nuclear Reactor Building. One of the DRD was encapsulated under 5.0 mm of aluminium, to remove contribution due to beta ray. Other dosimeter was used without any filter so that it can record dose due to both beta and gamma rays. Both DRD and TLD were simultaneously exposed for few hours at respective positions depending upon the dose rate at that place. It was observed that the DRD dose with filter gives correct estimation of gamma dose. However under field conditions DRD can be used to estimate beta dose when two DRDs are used simultaneously (one with aluminium filter and another without aluminium filter), the difference of the two reading multiplied by a factor of appropriate factor, gives beta dose. But such beta dose estimation can be done only for high energy Beta rays such as Sr/Y-90 source.

Table: 1 Dose estimation by DRD and TLD badge at different dose of Sr/Y-90

| *Delivered dose (mR)* | *Estimated dose by DRD (mR)* | *Estimated dose by TLD badge (mR)* |
|---|---|---|
| 700 | 115 ± 10 | 968.5 ± 53 |
| 500 | 90 ± 11 | 670.5 ± 40 |
| 300 | 57.5 ± 5 | 369.0 ± 12 |

**Conclusion:** This study helps in knowing the response of quartz fibre DRD and TLD badge used for personnel monitoring of radiation workers in power reactors. The response of DRD is nearly 5-6 times less than the actual dose delivered by Sr/Y-90 beta source alone. It does not show any reading for Kr-85 and Pm-147 source. So, radiation areas, where high energy beta rays are expected, such as due to Sr/Y-90, only TLD badge dose estimation should be considered for arriving at the dose received by the personnel.

**References**

1. Vohra KG, Bhatt RC, Chandra B, Pradhan AS, Lakshmanan AR, Shastry SS. A personal dosimeter TLD badge based on CaSO4:Dy teflon TLD discs. Health Phys 1980;38:193-7.
2. Pradhan AS, Adtani MM, Varadharajan G, Bakshi AK, Srivastava K, Bihari RR. Handbook on the use of TLD badge based on CaSO4: Dy Teflon TLD discs for Individual Monitoring: 2002. (BARC/2002/E/025).

This PDF is available for free download from a site hosted by Medknow Publications (www.medknow.com).

**P-125**

**Estimation of kidney volume in pediatric and adult patient undergoing routine abdominal CT examination**

AK Pandey, Rajesh Kumar Ranjan*, Pratik Kumar, S. Thulkar*. *Medical Physics Unit, IRCH and *Dept. of Radiodiagnosis, AIIMS, New Delhi, India*

**Introduction:** An average Indian differs considerably from his ICRP counterpart in respect of the anatomic, metabolic and physiological characteristics. Some attempt has been made to estimate the organ sizes for Indians of various age group obtained from the post mortem records of accident cases of Indian Adults and children.[1] The available data is scanty and lacks to provide information for all organs. One of the best ways of estimating the organ size is by X-ray tomography, which provides the 3D image of the organ. The liver volume has already been estimated by the same technique.[2] In this paper we present the measurement of kidney volume by multislice abdominal CT in Indian adults and children.

**Materials and Methods:** Fifty-seven (33 male and 24 female) patients from the cancer center of our institution, scheduled for routine contrast enhanced abdominal CT study were included in this study. The study was conducted on Somatom Volume Zoom (Siemens AG) multi detector four-slice CT scanner. Patient age, sex, height (cm) and weight (kg) were recorded prior to the scan. The acquisition parameters used for these patients were recorded. Routine abdominal protocol was used to acquire the studies. Individual patient studies were reconstructed for transverse images using B30L3CON as reconstruction filter and at different slice thickness (namely 3 mm, 5 mm, 6 mm and 8 mm) depending on clinical requirement. We used traverse section images for estimating the kidney volume. Kidney was outlined by hand with the help of ROI tools in interactive mode on each CT image. Volume calculation program available with the machine was used for calculation of kidney volume ($cm^3$). The acquisition parameters used for these patients were recorded. All the data were entered into Microsoft Excel spreadsheet for analysis.

**Results and Discussion:** Kidney volume in 57 patients of different age and sex, who underwent abdominal CT, is shown in Table 1. In adult group having more than 18 years of age, there were 35 patients.

[Downloaded free from http://www.jmp.org.in on Friday, July 29, 2011, IP: 130.14.254.25] || Click here to download free Android application for t

**Table 1: Shows the kidney volume of all the fifty seven patients**

| S. No. | Age (Yrs) | Height (cms) | Weight (kgs) | Kidney volume (cubic cm$^3$) | | |
|---|---|---|---|---|---|---|
| | | | | Left | Right | Total |
| 1 | 35 | 147 | 48 | 119.45 | 120.55 | 240 |
| 2 | 60 | 161 | 46 | 106.67 | 124.44 | 231.11 |
| 3 | 45 | 146 | 63 | 145.02 | 151.44 | 296.46 |
| 4 | 25 | 150 | 52 | 117.82 | 143.49 | 261.31 |
| 5 | 25 | 175 | 54 | 163.5 | 147.83 | 311.33 |
| 6 | 41 | 154 | 52 | 128.86 | 115.1 | 243.96 |
| 7 | 50 | 155 | 63 | 123.98 | 76.99 | 200.97 |
| 8 | 18 | 154 | 43 | 113.31 | 89.86 | 203.17 |
| 9 | 74 | 162 | 43 | 118.83 | 40.16 | 158.99 |
| 10 | 60 | 150 | 47 | 98.92 | 94.77 | 193.69 |
| 11 | 56 | 182 | 71 | 119.44 | 134.17 | 253.61 |
| 12 | 60 | 149 | 55 | 101.21 | 139.74 | 240.95 |
| 13 | 56 | 154 | 64 | 113.64 | 107.99 | 221.63 |
| 14 | 51 | 161 | 54 | 96.46 | 101.8 | 198.26 |
| 15 | 36 | 161 | 54 | 104.19 | 128.32 | 232.51 |
| 16 | 26 | 152 | 57 | 147.51 | 141.2 | 288.71 |
| 17 | 46 | 155 | 46 | 211.57 | 204.41 | 415.98 |
| 18 | 80 | 148 | 44 | 118.99 | 109.77 | 228.76 |
| 19 | 35 | 155 | 54 | 181.11 | 145.24 | 326.35 |
| 20 | 53 | 186 | 83 | 142.18 | 123.16 | 265.34 |
| 21 | 30 | 163 | 49 | 150.52 | 149.16 | 299.68 |
| 22 | 50 | 148 | 64 | 119.61 | 116.95 | 236.56 |
| 23 | 50 | 167 | 50 | 105.95 | 104.81 | 210.76 |
| 24 | 58 | 151 | 55 | 82.91 | 93.84 | 176.75 |
| 25 | 22 | 168 | 60 | 132.66 | 112.32 | 244.98 |
| 26 | 18 | 166 | 56 | 159.86 | 147 | 306.86 |
| 27 | 40 | 150 | 40 | 111.42 | 118.47 | 229.89 |
| 28 | 2 | 90 | 10 | 37.77 | 37.48 | 75.25 |
| 29 | 17 | 176 | 52 | 123.28 | 150.4 | 273.68 |
| 30 | 11 | 154 | 48 | 92.42 | 104.8 | 197.22 |
| 31 | 4 | 130 | 13 | 43.89 | 0 | 43.89 |
| 32 | 22 | 167 | 43 | 131.02 | 142.65 | 273.67 |
| 33 | 50 | 151 | 56 | 120.66 | 131.91 | 252.57 |
| 34 | 10 | 146 | 27 | 70.37 | 89.95 | 160.32 |
| 35 | 30 | 142 | 56 | 132.7 | 123.22 | 255.92 |
| 36 | 44 | 154 | 54 | 122.88 | 136.35 | 259.23 |
| 37 | 6 | 121.92 | 35 | 96.58 | 101.79 | 198.37 |
| 38 | 35 | 164 | 60 | 165.57 | 151.22 | 316.79 |
| 39 | 59 | 154 | 45 | 126.01 | 130.82 | 256.83 |
| 40 | 14 | 154 | 45 | 93.74 | 91.6 | 185.34 |
| 41 | 55 | 161 | 53 | 126.05 | 139.11 | 265.16 |
| 42 | 3 | 112 | 14 | 54.76 | 52.9 | 107.66 |
| 43 | 14 | 142 | 32 | 94.25 | 102.07 | 196.32 |
| 44 | 30 | 164 | 44 | 139.67 | 159.61 | 299.28 |
| 45 | 13 | 135 | 30 | 90.28 | 75.87 | 166.15 |
| 46 | 13 | 134 | 20 | 93.6 | 89.3 | 182.9 |
| 47 | 2 | 60.96 | 12 | 0 | 79.25 | 79.25 |
| 48 | 5 | 114 | 12 | 54.14 | 55.72 | 109.86 |
| 49 | 7 | 112 | 19 | 66.1 | 63.7 | 129.8 |
| 50 | 7 | 120 | 18 | 57.86 | 58.43 | 116.29 |
| 51 | 10 | 125 | 21 | 118.38 | 124.99 | 243.37 |
| 52 | 3 | 100 | 10 | 0 | 53.08 | 53.08 |
| 53 | 1 | 84 | 8 | 31.7 | 31.63 | 63.33 |
| 54 | 7 | 111 | 13 | 77.29 | 79.28 | 156.57 |
| 55 | 5 | 114 | 12 | 43.35 | 42.28 | 85.63 |
| 56 | 3 | 100 | 8 | 98.33 | 0 | 98.33 |
| 57 | 7 | 118 | 17 | 67.1 | 60.2 | 127.3 |

The mean kidney volume varies from 72.61 cm$^3$ (pediatrics: age 1-2 years) to 254.22 cm$^3$ (adult: age 18 years and above). Our result of kidney volume in adult group (254 ± 49.25 ml) measured by CT is slightly higher than those published by Jain *et al*, 1995 (male 212±46 g: female 202±45g); from postmortem study of accidents victims. Between 1-2, 3-7, 8-12, 13-17 years of age and adults the estimated mean kidney volumes were 72.61±8.28 ml, 111.52±43.62 ml, 200.30±41.61 ml, 200.87±42.10 ml and 254.22±49.25 ml respectively. The mean, standard deviation and range of the estimated kidney volumes age-wise distribution are given in Table 2. The study can be extended to all the organs of a large number of subjects of different age groups, which will make a good data for Indian reference man for dosimetry and other clinical applications.

**Table 2: Agewise distribution of estimated kidney volume**

| Age-wise (years) | No. of patients | Mean ± SD (cm$^3$) | Range (cm$^3$) | Jain et al. |
|---|---|---|---|---|
| 1-2 | 3 | 72.61±8.28 | 63.33-79.25 | - |
| 3-7 | 11 | 111.52±43.62 | 43.89-198.37 | - |
| 8-12 | 3 | 200.30±41.61 | 160.32-243.37 | - |
| 13-17 | 5 | 200.87±42.10 | 166.15-273.68 | - |
| 18 and above (adult) | 35 | 254.22±49.25 | 158.99-415.98 | male 212±46 g: female 202±45g |

### References

1. Jain SC, Metha SC, Kumar B, Reddy AR, Nagaratnam A. Formulation of the reference Indian adult: Anatomic and physiologic data. Health Phys 1995;68:509-22.
2. Pandey AK, Pant GS, Sarita, Guglani B, Thulkar S, Kumar P. Estimation of kidney volume in pediatric and adult patient undergoing routine abdominal CT examination. Proc Nat Conf Soc Indian Radiograph 2004;60.

### P-126

### Verification of manufacturer's displayed (CTDI$_W$) values in a multislice CT scanner for various abdominal protocol

AK Pandey, Himanshu Pandey*, Pratik Kumar, S Thulkar*. *Medical Physics Unit, IRCH and *Dept. of Radiodiagnosis, AIIMS, New Delhi, India*

**Introduction:** In order to evaluate patients' exposure to radiation in computed tomography, dose parameter called the computed tomography dose index (CTDI) is used. A computed tomography dose index (CTDI) may also be used to characterize the radiation doses associated with CT scanners.[1] CTDI in the standard head or body CT dosimetry phantom provide an indication of the average dose over a single slice for each setting of nominal thickness. On the assumption that dose in a particular phantom decreases linearly with radial position from the surface to the center, then the normalized average dose to the slice is approximated by the (normalized) weighted CTDI (CTDI$_w$). Whenever a CT protocol is selected, the scanner displays the estimated CTDI$_w$ value in the examination going to be conducted. The purpose of this study was to verify the scanner displayed CTDI$_w$ (weighted CTDI) value during selection of acquisition protocol used in abdominal CT examination.

**Materials and Methods:**

$$CTDI_w = \frac{CTDI_{center} + 2 * CTDI_{periphery}}{3}$$

The CTDI$_w$ in body phantom (Radcal Corporation, USA) was measured using the 10 cm pencil ionisation chamber (10x5-3CT, S/N 8811, Radcal Corporation, USA). The phantom was placed on the table and position in scan plane such that the central hole is at isocenter. For positioning the chamber at isocenter, assessment was made by laser lights. The phantom has nine holes, one at center, four at a 160 mm diameter and other four at 320 mm diameter. Ionization chamber was inserted in the central hole to obtain the value of CTDI center and all other eight

This PDF is available for free download from a site hosted by Medknow Publications (www.medknow.com).

[Downloaded free from http://www.jmp.org.in on Friday, July 29, 2011, IP: 130.14.254.25] || Click here to download free Android application for t

**Table 1: CTDIw at different acquisition protocols**

| *KVp* | *MAs* | *Slice thickness* | *CTDIw measured* | *CTDIw displayed* |
|---|---|---|---|---|
| 140 | 90 | 6 | 3.857 | 9.81 |
| 120 | 80 | 10 | 6.285 | 6.08 |
| 120 | 140 | 8 | 4.46 | 04.04 |
| 120 | 126 | 8 | 4.42 | 9.58 |
| 120 | 90 | 8 | 2.80 | 6.84 |
| 120 | 85 | 8 | 2.59 | 6.48 |
| 120 | 32 | 8 | 1.10 | 2.43 |

holes were filled with perspex rod provided with the phantom. Topogram was taken and scan was planned for minimal scan length, which provided four slices (four slice CT scanner). The same procedure was repeated to obtain the value of CTDI periphery by inserting the ionization chamber in peripheral holes. The scanning protocol (kV, mA, sec, slice thickness, standard reconstruction algorithm) was documented. The $CTDI_w$ was calculated using the formula.

**Results and Conclusion:** The $CTDI_w$ (320 mm diameter) at different acquisition protocol is given in Table 1. The analysis of the data shows that there is inconsistent disparity between our measured $CTDI_w$ values and the displayed one on the CT console. Probably it difference may be due to the difference in experimental arrangement. However, the manual of the CT scanner is silent about the measurement technique adopted by manufacturer. Due to this reason it is suggested that user should measure the $CTDI_w$ values.

### Reference

1. Shope TB, Gagne RM, Jhonson GC. A method for describing the doses delivered by transmission X-ray computed tomography. Med Phys 1981;8:488-95.

**P-127**

### Relationship between image noise and patient weight in abdomen CT: A prospective study

AK Pandey, Raju Sharma*, AK Gupta*, Sarita*. *Medical Physics Unit, IRCH and *Department of Radiodiagnosis, All India Institute of Medical Sciences, New Delhi - 29, India*

**Introduction:** Computed tomography (CT) is an extremely valuable diagnostic tool. The rapid technological developments in CT have resulted in an ever-increasing number of CT examinations. CT has become a major source of radiation dose for the population. Although CT accounts for about 2-4% of all radiological examinations it contributes up to 40% of the population dose in some countries. Due to high radiation dose often delivered by CT it is important to ensure that the required diagnostic information is achieved with the minimum possible dose to the patient. The aim of this study was to make a baseline evaluation of scanner parameters used for abdomen CT, assess image noise and patient dose at a tertiary care hospital. The image noise was correlated with anthropometric parameters like patient weight and effective diameter, to confirm the hypothesis that noise increases as a function of weight. This was the first phase of a larger multi-national study being carried out with the ultimate aim of dose reduction in CT.

**Materials and Methods:** Fifty one patients from the cancer center of our institution, scheduled for routine contrast enhanced abdominal CT study were included in this study. To study the effect of weight, patients were recruited under five different weight categories and the image noise data was collected. The study was conducted on Somatom Volume Zoom (Siemens AG, Erlangen Germany) multidetector four slice CT scanner. Patient height (cm) and weight (kg) were recorded prior to the scan. Abdominal size was assessed by measuring AP and lateral diameter (skin to skin distance). ROI (size > 1 $cm^2$) was placed on homogenous areas on liver, spleen and abdominal aorta at three contiguous levels to measure CT number and noise at these levels. The average noise of these three levels was calculated and the mean of these three values was taken as image noise. The acquisition parameters used for these patients were recorded. All the data were entered into Microsoft Excel spreadsheet for analysis. The data was normalized for 140 mAs. Two separate graphs were plotted between effective diameter versus noise and the weight versus noise.

**Table 1: Baseline values of scanner parameters for abdominal CT**

| | *Range* | *Mean* | *Median* | *Mode* |
|---|---|---|---|---|
| Age (years) | 4-78 | 38.88 | 40 | 40 |
| Weight (kg) | 12-85 | 48.8 | 52 | 40 |
| Height (cm) | 90-173 | 148.54 | 152 | 156 |
| CTDI vol (mGy) | 8.46-13.16 | 12.59 | 12.33 | 13.16 |
| DLP | 125-626 | 458.80 | 481 | 563 |
| kvp | 120,140 | | | 120 |
| MAs | 90,130,140 | | | 130 |
| Reconstructed slice thickness | 3,5,6,8 | | 8 | 8 |
| Horizontal distance | 17.33 -33.89 | 26.52 | 27.1 | 27.83 |
| Vertical distance | 13.39-27.18 | 20.11 | 20.82 | - |
| Mean diameter | 15.2-30.0 | 23.07 | 23.48 | - |
| Noise | 5.3-16.0 | 9.09 | 8.95 | - |
| FOV | 196 - 416 | 308.19 | 313 | 296 |

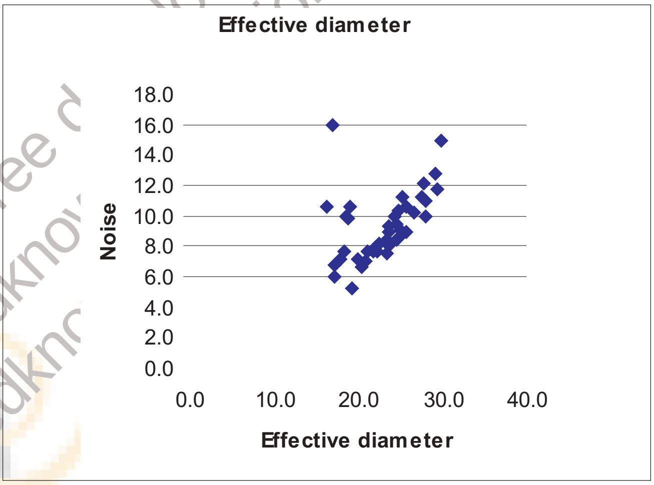

**Figure 1a:** A graph of image noise versus effective diameter (cm)

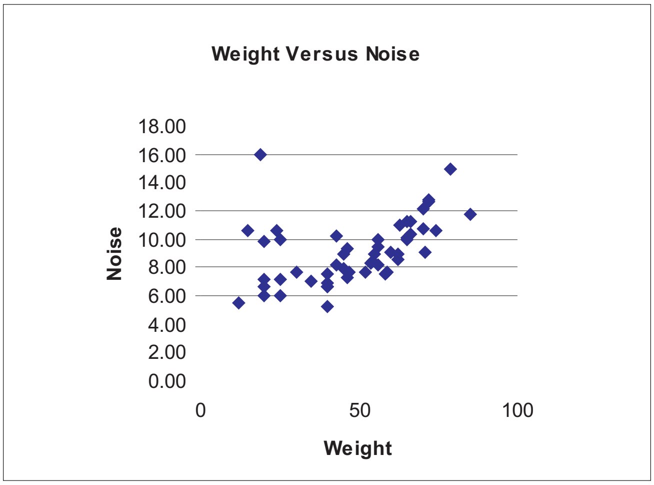

**Figure 1b:** A graph of image noise versus patient weight (kg)

**Results and Discussion:** A total of fifty one patient (male: female = 24:27) were included in the study. The baseline value of the scanner parameters used in abdomen study and the patient's age, weight and height were summarized in the Table 1. Two separate graphs one [Image noise versus patient weight, [Figure 1a] and another [Image

[Downloaded free from http://www.jmp.org.in on Friday, July 29, 2011, IP: 130.14.254.25] || Click here to download free Android application for t noise versus effective diameter, [Figure 1b] were drawn. The trend line of the graph is suggestive of increase in image noise with patient weight and effective diameter. However, a larger study is required to corroborate our findings. No definite relationship was observed between radiological assessment and image noise.

**Acknowledgement:** This work is part of IAEA funded research project (research contract no. 12383/RD)

**P-128**

**Spinal cord response to altered fractionation and reirradiation: Radiobiological considerations and role of bioeffect models**

KM Ganesh, Sanjay S Supe, BN Sankar*. *Kidwai Memorial Institute of Oncology, Bangalore, *Anna University, Chennai, India*

**Introduction:** The aim of radiation oncologist is to implement an uncomplicated loco regional control of cancer by radiation therapy. The bioeffect of a physical dose depends on the nature of the tissue, fractionation scheme, dose rate and treat-ment time. The transformation of absorbed dose into a bioeffect dose is controlled by treatment variables and the radiobiological characteristics of the relevant tissue. Various bioeffect models have been proposed to predict the biological effect of radiotherapy treatments. Spinal cord myelitis limits the dose to tumours in the head and neck, thoracic and upper abdominal regions resulting in reduction of tumour control probability. Radiation myelopathy is one of the most devastating complications of clinical radiothera-py. Treatment techniques that are designed to minimize the risk of spinal cord injury are likely to underdose the tumour consequent failure to control the disease. Since radiation myelopathy results in severe and irreversible morbidity, it is important to establish the tolerance dose of the spinal cord. A number of patients have recently been reported to have developed radiation myelopathy following hyperfractionated accelerated radiotherapy. As the survival rates of patients increase, radiation oncologists are more frequently faced with the problem of treatment of late recurrence or second tumours situated within or close to previously treated site. A rationale for taking a decision in treating in such a condition is even more complex than the original condition and requires knowledge of the kinetics of decay of occult injury of the previous treatment.

**Materials and Methods:** To test the validity of Extrapolated Response Dose (ERD), clinically reported data of altered fractionation to the spinal cord for 7 patients reported by Wong *et al.*, Saunders *et al.* and Bogaert *et al.* were analysed, ERD values were calculated and compared with compiled clinical literature data of 3233 patients for the incidence of spinal cord myelitis reported by Cohen and Creditor, Wara *et al.*, Abbatucci *et al.* and Jeremic *et al.* for conventional fractionation. ERD values were estimated with $\alpha/\beta$ of 2.5 Gy for the conventional and altered fractionation data. To test the validity of TE concept for clinical data of re-irradiation tolerance of the spinal cord, the data of the 22 patients compiled by Nieder *et al.* were used. Clinical data compiled from the literature of Cohen and Creditor,[8] Wara *et al.*, Abbatucci *et al.* and Jeremic *et al.* were used for comparison.

**Results:** The correlation of ERD with probability of spinal cord myelitis was statistically significant ($P$<0.001) for conventional data of 3233 patients. The incidence of spinal cord myelitis in the ERD range of 91-130 Gy is 5.3%. The ERD values for 7 clinically reported patients treated with altered fractionation schedule varied from 101 Gy to 155 Gy. To keep the incidence of spinal cord myelitis below 5% probability the ERD value of less than 91 Gy is preferable in clinical settings. Total Effect (TE) values were estimated with $\alpha/\beta$ of 2.5 Gy for the conventional data. TE values for re-irradiation data were estimated with $\alpha/\beta$ value of 2.5 Gy for first and second course of treatment. TE values were also evaluated with 2.3 Gy for the first course and 1.9 Gy for the second course to account for variable radiosensitivity. Correlation of TE with probability of spinal cord myelitis was statistically significant for the conventional data ($P$<0.001). The probability of myelitis increased significantly beyond TE values of 209. The incidence of spinal cord myelitis occurred for re-irradiation data and TE1(2.5) and TE2(2.5) were beyond 217 for either first course or second course. The occurrence of spinal cord myelitis is found to be more probable when the total TE at 2.5 Gy is above 300. This was in agreement with conventional data where the corresponding probability was above 16.5%. The spinal cord myelitis was observed for the re-irradiation data when TE1(2.3) or TE2(1.9) reached a value of 202.

**Conclusion:** The validity of the ERD equation is proved for the prediction of spinal cord myelitis for altered fractionation schedules. Our study also indicates that the TE formula can be meaningfully used for the prediction of re-irradiation tolerance spinal cord as it is based on strong radiobiological foundation.

**P-129**

**Design and characterization of total skin electron therapy facility with high energy linear accelerator**

KV Rakesh, Mathew Joby, NS Shine, Prasad P Satheesh, Bhaskaran K Pillai. *Department of Radiation Oncology, Amrita Institute of Medical Sciences, Kochi, India*

**Introduction:** Electron energy ranging from 2-6 MeV from linear accelerators are used for treating superficial lesions covering large areas of the body, such as Mycosis Fungoides. At these energies electron beams are characterized by a rapid falloff in dose beyond a shallow depth and minimal X-ray background (1% or less). The physical principle underlying these techniques is to use a Perspex beam spoiler of 8 mm to 10 mm thickness at large distance from the isocentre and degrade a 6-8MeV (HDRE Mode) electron beam to obtain a low energy, less penetrating beam of large field size. Low Z scattering material, Perspex produces a shower of degraded energy electrons at the patient's skin level, resulting from collision of primary electrons. We have commissioned the technique of irradiation for TSET at Amrita Institute of Medical Sciences at Kochi, the technical and physical aspects of the setup are highlighted.

**Materials and Methods:** 6 MeV electron beam from Elekta Precise LINAC in high dose rate mode (3000 MU/min at isocentre), using the special electron applicator was selected for TSET. Acrylic sheet of 200 cm x 100 cm x 1 cm was mounted on a frame as beam degrader. The beam degrader is kept at 2.75 m from isocentre with a clearance of 1.0 m from the distal wall. A distance of 25 cm from the Perspex degrader is planned to provide a Focus Skin Distance (FSD) of 4 m. The combined beam profile was obtained by summing the single field (beam angles 270° ±18°) beam profiles using PPC 40 plane parallel chamber (with mini phantom), the detector being moved in the vertical and horizontal planes at small intervals with the help of PTW RFA motor assembly. Depth dose measurements at treatment SSD in solid phantom at 1mm interval was taken. From the depth dose curve, the range values Rp, R50 were measured and electron energies Ep,o and Eo were calculated. The absorbed dose rate in Gy/MU was measured by keeping the detector at $d_{max}$ (1 mm) in solid water phantom. Correction factors 1) $K_Q$ (Obtained by extrapolating graph between $R_{50}$ and Kq values for this chamber), $K_{ecal}$ (TG-51) 2) $K_{TP}$ 3) polarity 4) recombination 5) leakage were applied for the ionization readings to arrive at the absorbed dose. The geometry of the treatment setup is by 6 dual-fields (Stanford Technique), to reduce self shielding.

**Conclusion:** The AAPM protocol TG 30 (Report 23) recommends field size of the composite electron beam at the patient treatment plane must be approximately 200 cm in height by 80 cm in width to encompass the largest patient. Within this rectangle, a vertical uniformity of ± 8% and a horizontal uniformity of ± 4% over the central 160 cm x 60 cm area of the treatment plane are achievable goals for most techniques. We are able to achieve the recommended dose uniformity over the specified area.
